# Individuals who deviate from polygenic expectation are enriched for damaging variants in genes linked to rare disease

**DOI:** 10.1016/j.ajhg.2026.05.013

**Published:** 2026-06-22

**Authors:** Nikolas A. Baya, Frederik H. Lassen, Barney Hill, Samvida S. Venkatesh, Hannah Currant, Cecilia M. Lindgren, Duncan S. Palmer

**Affiliations:** 1 Big Data Institute, Li Ka Shing Centre for Health Information and Discovery, University of Oxford, Oxford, UK; 2 Centre for Human Genetics, Nuffield Department of Medicine, University of Oxford, Oxford, UK; 3 Department of Statistics, University of Oxford, Oxford, UK; 4 Nuffield Department of Population Health, Medical Sciences Division, University of Oxford, Oxford, UK; 5 Nuffield Department of Women’s and Reproductive Health, Medical Sciences Division, University of Oxford, Oxford, UK; 6 Broad Institute of MIT and Harvard, Cambridge, MA, USA; 7 The Pioneer Centre for SMARTbiomed, Big Data Institute, Li Ka Shing Centre for Health Information and Discovery, University of Oxford, Oxford, UK

**Keywords:** polygenic scores, rare variants, rare disease, complex traits, misaligned, liability threshold, height, cholesterol, diabetes, coronary artery disease

## Abstract

Polygenic scores (PGSs) stratify disease risk but often fail to capture individual variation. “Misaligned” individuals, whose observed phenotypes deviate from their genetically expected values based on PGS, provide a powerful model for identifying factors beyond common-variant effects, including additional genetic factors. Here, we apply misalignment classification and enrichment testing frameworks to seven continuous traits and three diseases, assessing whether misaligned individuals in the UK Biobank are enriched for rare (minor-allele frequency [MAF] <0.1%) damaging genetic variation. We identified significant enrichment of predicted loss-of-function (pLoF) variants in *COPB2* and *GORAB* among individuals with lower-than-expected bone mineral density. Regarding stature, shorter-than-expected individuals were enriched for pLoF variants in *ACAN* and *IGF1*, while taller-than-expected individuals showed enrichment for damaging missense variants in *FBN1*. Transitioning from validation to discovery, we performed an exome-wide scan for genes associated with misalignment and identified 74 significant genes, including *KANK1*, a gene which may have a protective role against primary ovarian insufficiency, and *ACSL6*, a lipid metabolism gene where damaging missense variation was associated with lower-than-expected BMI. For diseases, results supported a liability threshold model involving counteracting common and rare variant effects. Diagnosed type 2 diabetes mellitus patients with rare pathogenic variants in *HNF1A* and *HNF4A* possessed significantly lower polygenic risk than those without. Conversely, coronary artery disease controls harboring protective *ANGPTL3* variants had nominally higher polygenic risk. This study highlights the power of misalignment-based analyses in complex continuous phenotypes and disease with the potential to validate known genetic contributors to traits and identify previously unassociated genes.

## Teaser

Polygenic scores stratify disease risk but often miss individual variation. We show that individuals whose traits deviate from polygenic expectation are enriched for rare, damaging variants. This misalignment framework supports a liability threshold model of counteracting common and rare variant effects, offering a powerful tool to explore complex disease architecture.

## Introduction

**Figure 1:**
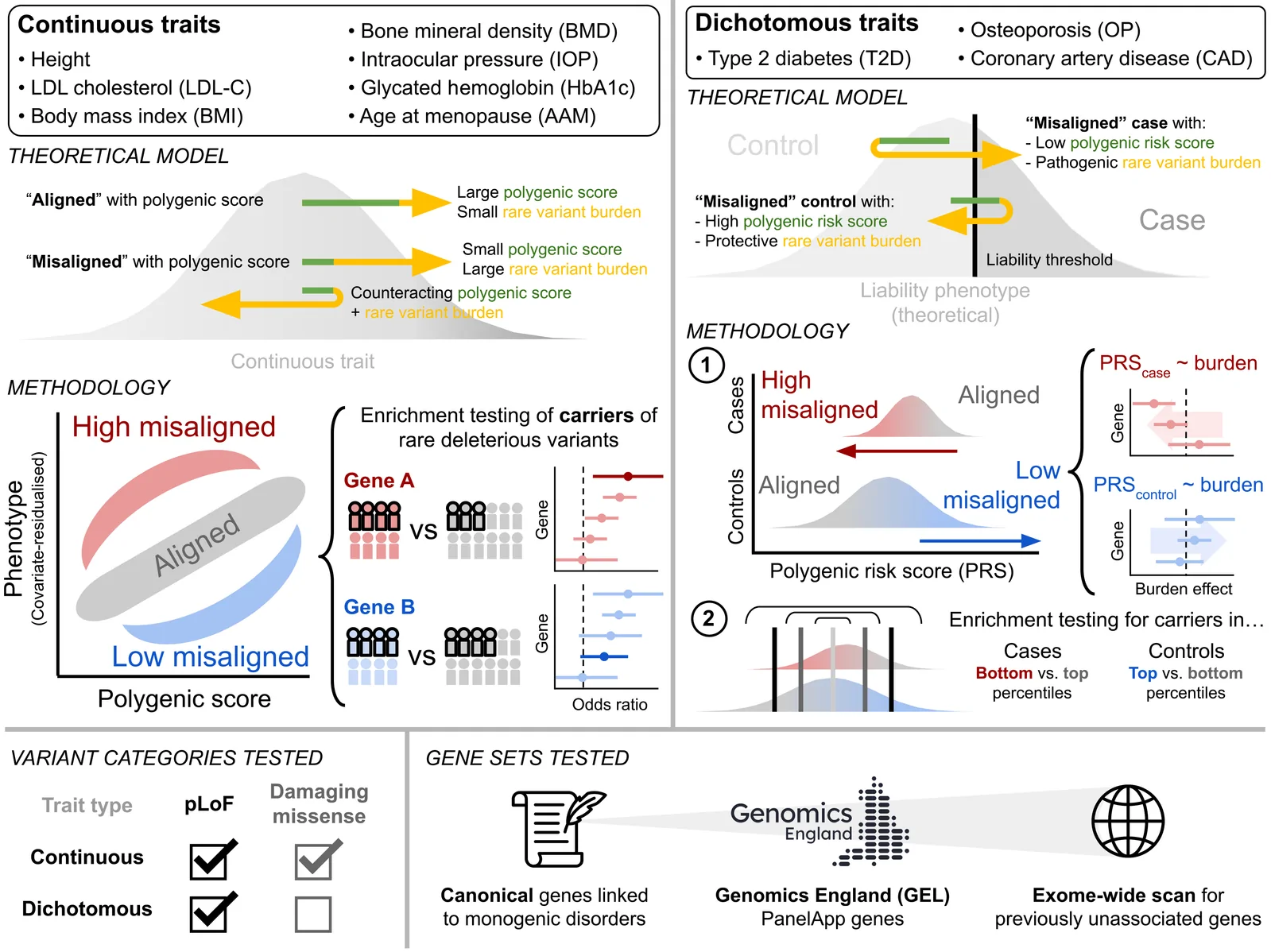
Overview of phenotypic misalignment analysis We investigated seven continuous and three dichotomous traits to determine whether deviation from common-variant genetic prediction is driven by rare damaging variants. Theoretical model: for continuous traits, misaligned individuals are defined by large residuals between observed and expected phenotypes (PGSs). For dichotomous traits, under the liability threshold model, misaligned individuals include cases with low PRSs and controls with high PRSs, with misalignment driven by pathogenic or protective rare variants. Methodology: enrichment in continuous traits was assessed by comparing odds of harboring rare variants in high/low misaligned groups against aligned individuals. For dichotomous traits, we utilized a two-step process: regression of PRS against rare variant burden and enrichment testing based on PRS percentiles. Variant categories tested: we computationally annotated variants and used pLoF or putatively damaging missense variants in our enrichment and burden tests. Only pLoF variants were tested for dichotomous traits. Gene sets tested: tests were applied in stages across canonical genes linked to monogenic disorders, GEL PanelApp gene panel genes, and an exome-wide scan.

Modeling *how* an individual develops disease is key to preventing disease. One approach to modeling genetic disease risk is the liability threshold model.^1^^,^^2^ This model assumes a continuous “liability” for disease, comprised of genetic and environmental contributions.^2^ An individual develops disease if their liability exceeds a certain threshold. The genetic risk portion of this liability can be separated into risk contributed by common variants and rare variants for a heritable trait^3^ (Figure 1).

One way to quantify the common-variant genetic component of a phenotype is with polygenic scores (PGSs).^4^^,^^5^ PGSs have transformed the study of complex traits by enabling the prediction of phenotypes and disease risk based on the aggregate contribution of common genetic variation.^6^^,^^7^^,^^8^^,^^9^ However, given the many other contributors to phenotypic variability in the population, assessing how an individual’s observed phenotype deviates from expectation based on their PGS could refine signal for other components (e.g., rare variants, environmental effects) and improve power in association studies. For example, adjusting for PGS has been demonstrated to improve power in rare-variant association analyses.^10^

There are other applications for this notion of divergence from the genetically expected phenotype measured by aggregating common-variant effects. Under a liability threshold model, individuals diagnosed with disease but with low common-variant risk may be expected to have other independent components of risk causing them to cross the liability threshold. This is consistent with recent literature showing that diagnosed individuals with low common-variant risk are more likely to harbor rare pathogenic variants^11^ or have a monogenic diagnosis.^12^ There are many other recent examples of work involving the interplay of polygenic and monogenic risk, especially demonstrating how common-variant polygenic risk can modulate the penetrance or expressivity of monogenic risk variants. Disorders for which this has been demonstrated include breast cancer^13^^,^^14^^,^^15^ (MIM: 114480); Huntington disease^16^^,^^17^ (MIM: 143100); glaucoma 1, open angle, A^18^^,^^19^ (MIM: 137750); familial hypercholesterolemia (FH)^13^^,^^20^; obesity^20^ (MIM: 601665); developmental disorders^21^^,^^22^; chronic kidney disease^23^; and schizophrenia^24^ (MIM: 181500).

Here, we test for enrichment of rare (minor-allele frequency [MAF] < 0.1%) deleterious variants in UK Biobank (UKB) individuals who deviate from their genetically expected phenotype based on PGS (Figure 1), using common-variant PGSs calculated by Genomics PLC^25^ and provided through the UKB. We assess seven continuous traits (standing height, low-density lipoprotein cholesterol [LDL-C], BMI, glycated hemoglobin [HbA1c], bone mineral density [BMD], intraocular pressure [IOP], and age at menopause [AAM]) and three dichotomous disease status traits (type 2 diabetes mellitus ([T2D] [MIM: 125853]), coronary artery disease [CAD], and osteoporosis [OP] [MIM: 166710]) in our analysis. These traits were selected due to having polygenic and monogenic components of heritability. The selection of dichotomous traits was also motivated by the availability of their corresponding continuous “liability” traits (HbA1c for T2D, LDL-C for CAD, BMD for OP), which enabled us to use many of the same gene sets between traits. For continuous traits, we test for rare putatively deleterious variation in individuals with misaligned phenotypes using an existing framework^26^ and develop a testing framework to enable the analysis of dichotomous traits.

We build the scope of our investigation in stages. We begin our enrichment testing in a small set of “canonical” genes, curated for each trait, linked to monogenic disorders or strongly associated with the trait. In this context, we have a strong prior for misaligned individuals to show enrichment for deleterious rare variation, given previous work in a smaller subset of the UKB.^26^

We then expand our analysis to a larger set of genes considered to be of diagnostic grade for rare disorders related to our selected traits, taken from the Genomics England gene panel.^27^ This approach allows us to test many more genes than the canonical genes while maintaining a hypothesis of rare genetic disorder enrichment in misaligned individuals.

Finally, we explore whether this framework of testing for deleterious variant enrichment in misaligned individuals can be re-appropriated for discovery of pathogenic or protective genetic effects. To do this, we treat misalignment as the trait of interest and perform gene-level association testing of protective and pathogenic damaging rare variation across the exome.

Altogether, this study comprises a comprehensive investigation of phenotype deviation from common-variant polygenic expectation, leveraging the data available in the UKB to enable study of misalignment at greater scale (>2× larger sample size and >3× the number of phenotypes compared to previous work in continuous traits^26^; ∼8× larger sample size for similar studies in dichotomous traits^11^^,^^12^). We unify misalignment enrichment testing of continuous liability and dichotomous disease phenotypes under the liability threshold model and provide further evidence for counteracting pathogenic/protective common- and rare-variant effects.

## Methods

### Exome sequencing data quality control and assigning population labels

We performed sample-, variant- and genotype-level quality control (QC) for the UKB 450K exome sequencing (ES) dataset, following the approach of Karczewski et al*.*^28^ Broad continental genetic ancestry labels (Africans, Admixed Americans, East Asians, Europeans, South Asians) were assigned following the approach described by Lassen et al.*,*^29^ using a random forest (RF) classifier trained on the 1000 Genomes dataset.^30^ We subset to individuals with European genetic ancestry, resulting in a high-quality call set of 402,375 samples and 25,229,669 variants.

### Variant consequence annotation

We annotated ES variants using Variant Effect Predictor (VEP) v.105 (corresponding to GENCODE v.39)^31^ with the Loss-of-Function Transcript Effect Estimator (LOFTEE) v.1.04_GRCh38^32^ and dbNSFP^33^ plugins, annotating variants with Combined Annotation Dependent Depletion (CADD) v.1.6,^34^ and Rare Exome Variant Ensemble Learner (REVEL)^35^ using dbNSFP v.4.3 and loss-of-function (LoF) confidence using LOFTEE. We provide code and instructions for this step in our VEP_105_LOFTEE repository (https://github.com/BRaVa-genetics/vep105_loftee), which contains a Docker/Singularity container for reproducibility of annotations. Next, we ran SpliceAI v.1.3^36^ using the GENCODE v.39 gene annotation file to ensure alignment between VEP and SpliceAI transcript annotations. For variant-specific annotations, we use “canonical” transcripts. We separated variants by transcript using bcftools +split-vep and filtered to MANE Select^37^ protein-coding transcripts. If the gene lacked a MANE Select transcript, we selected the canonical transcript defined by GENCODE v.39. Using this collection of missense, predicted LoF (pLoF), splice metrics, and annotations of variant consequence on the canonical transcript, we determined four variant categories for gene-based testing.

### Variant consequence categories

(1) High confidence pLoF: high-confidence LoF variants as defined by LOFTEE^32^ (LOFTEE HC).
(2) Damaging missense/protein-altering: at least one of the following:
  (a) Variant annotated as missense/start-loss/stop-loss/in-frame insertion or deletion (indel) and (REVEL ≥0.773 or CADD ≥28.1 or both).
  (b) Any variant with SpliceAI delta score (DS) ≥0.2 where SpliceAI DS is the maximum of the set {DS_AG, DS_AL, DS_DG, DS_DL} for each annotated variant (where DS_AG, DS_AL, DS_DG, and DS_DL are DS [acceptor gain], DS [acceptor loss], DS [donor gain], and DS [donor loss], respectively).
  (c) Low-confidence LoF variants as defined by LOFTEE (LOFTEE LC)
(3) Other missense/protein-altering: missense/start-loss/stop-loss/in-frame indel not categorized in (2).
(4) Synonymous: synonymous variants with SpliceAI DS <0.2 in the gene.

REVEL and CADD score cutoffs are chosen to reflect the supporting level for pathogenicity (PP3) from the American College of Medical Genetics and Genomics and the Association for Molecular Pathology (ACMG/AMP) criteria.^38^ For our main analyses we only test pLoF and damaging missense variants. The two other variant consequence categories (“other missense/protein-altering” and “synonymous”) are only used for our singleton burden analysis (Note S4).

### Phenotype curation

#### Continuous phenotypes

We selected phenotypes among those with “standard polygenic risk scores (PRS)” available in the UKB.^25^ Standing height (UKB field ID 50) and LDL-C (UKB field ID 30780), the two phenotypes analyzed by a previous study of phenotype misalignment,^26^ were chosen to benchmark our approach.

The five other continuous traits (BMI [UKB field ID 21001], estimated BMD [UKB field ID 78], IOP [UKB field IDs 5262 and 5254], HbA1c [UKB field ID 30750], and AAM [UKB field ID 3581]) were prioritized for inclusion based on sample size in the UKB, heritability, how closely the trait available in the UKB matched the trait used to create the PGS, and whether there was prior evidence of monogenic disorders or genes with strong rare variant burden effect contributing to trait variability (Table 1). IOP was calculated as the mean between left (UKB field ID 5262) and right (UKB field ID 5254) corneal compensated measurements. We chose the corneal compensated measurement method instead of the Goldmann-correlated measurement method because it has been reported to be a better indicator for glaucomatous eyes.^39^ For AAM, we excluded individuals who responded “do not know” or “prefer not to answer.”

**Table 1:** Canonical genes associated with monogenic disorders for continuous phenotypes

**Trait**	**Disorder**	**Effect direction of LoF**	**Genes**
Standing height	growth disorder	negative (pathogenic)	*EZH2*, *FBN1*, *NSD1*
		positive (pathogenic)	*ACAN*, *FGFR3*, *IGF1*, *IGF1R*, *NPR2*, *SHOX*
LDL-C	familial hypercholesterolemia	negative (protective)	*APOB*, *PCSK9*
		positive (pathogenic)	*LDLR*
BMI	monogenic obesity	positive (pathogenic)	*LEP*, *LEPR*, *MC4R*, *PCSK1*, *POMC*
IOP	glaucoma 1, open angle, A	positive (pathogenic)	*MYOC*
BMD	osteogenesis imperfecta, type II	negative (pathogenic)	*COL1A1*, *COL1A2*
HbA1C	MODY	positive (pathogenic)	*GCK*, *HNF1A*
AAM	N/A	positive (protective)	*CHEK2*

Genes for each disorder are listed in alphabetical order. See methods for a description of how genes were selected. N/A, not applicable.

#### Dichotomous phenotypes

We included three dichotomous disease status phenotypes: T2D (International Classification of Diseases, 10th Revision [ICD-10] code E11), CAD (ICD-10 code I25.1), and OP (ICD-10 codes M80 and M81). Case status was defined by ICD-10 diagnoses (UKB field ID 41270). For each disease, individuals with the disease ICD-10 code(s) were defined as cases, while everyone lacking the disease ICD-10 code(s) but for whom UKB field ID 41270 was defined, were assigned control status.

#### PGSs

We used PGSs determined by Genomics PLC^25^ and provided through the UKB (data field IDs in Table S1) for our analyses. We chose to use the “standard PRS” (UKB category 301) scores instead of the “enhanced PRS” (UKB category 302) because the standard PRS category of scores is trained solely using non-UKB data,^25^ making it less prone to overfitting to the UKB, and as a result, due to their availability for more than three times as many UKB samples compared to the enhanced PRS.

### Gene curation for monogenic and rare disorders

#### Curation of canonical genes for monogenic disorders

We defined a pilot set of “canonical” genes associated with monogenic disorders (hereafter referred to as canonical genes), where we believed we would be most likely to observe enrichment of deleterious variants in misaligned individuals. These are genes linked to some of the most common monogenic disorders associated with the phenotype being studied. Our curation process for each phenotype, informed by evidence from literature, was as follows.

For standing height and LDL-C, we selected the gene set associated with monogenic disorders provided in a study of misaligned individuals in a UKB subset^26^ for both consistency and to check for recapitulation of associations (Table 1). For BMI, we selected the first five genes discovered to be associated with monogenic obesity^40^: *MC4R* (MIM: 155541), *PCSK1* (MIM: 162150), *LEP* (MIM: 164160), *POMC* (MIM: 176830), and *LEPR* (MIM: 601007). For BMD, we chose the two genes (*COL1A1* [MIM: 120150] and *COL1A2* [MIM: 120160]) where pathogenic variation is the most common cause of monogenic OP.^41^ For IOP, we chose *MYOC* (MIM 601652), in which pathogenic variants are the most common single-gene cause of primary open-angle glaucoma via increased IOP.^42^ For HbA1c, we chose the genes where pathogenic variation is commonly responsible for maturity-onset diabetes of the young (MODY), the most common monogenic form of diabetes.^43^ Within the subtypes of MODY, *GCK*-MODY (MODY, type 2 [MIM: 125851]) and *HNF1A*-MODY (MODY, type 3 [MIM: 600496]), caused by pathogenic variants in *GCK* (MIM: 138079) and *HNF1A* (MIM: 142410), respectively, are the two most common subtypes, accounting for around 80% of MODY cases in the UK.^44^^,^^45^ Patients with both subtypes can present with hyperglycemia,^43^^,^^46^ which can be indicated by higher-than-normal HbA1c levels.^47^ While there are other monogenic disorders that may have effects on HbA1c (e.g., anemia; congenital; nonspherocytic hemolytic, 1^48^^,^^49^ [MIM: 300908]), we were primarily interested in using HbA1c as a liability trait for T2D and thus focused on genes with a glycemic association. For AAM, there are no common monogenic disorders. As a proxy, we selected *CHEK2* (MIM: 604373), the gene with highest strength of association in the burden test for pLoF variants for AAM in Genebass^28^ (pLoF burden *p* = 3.75 × 10^−32^). Early menopause can be caused by primary ovarian insufficiency (POI), a partly monogenic disorder.^50^ However, there are more than 100 genes putatively associated with monogenic POI, and in the UKB, most POI cases are not caused by monogenic mechanisms.^50^ For T2D, we selected the four genes associated with the most common subtypes of MODY in the UK: *HNF1A*, *GCK*, *HNF4A* (MIM: 600281), and *HNF1B*^44^ (MIM: 189907). We included *SLC30A8* (MIM: 611145) as a gene where LoF is implicated as being protective against T2D.^51^ For CAD, we selected the same genes associated with FH (*PCSK9* [MIM: 607786], *APOB* [MIM: 107730], and *LDLR* [MIM: 606945]) as used for LDL-C because monogenic risk variants in these genes have been shown to interact with CAD polygenic risk to modulate overall disease risk.^13^ We also included *ANGPTL3* (MIM: 604774), where LoF variants cause hypobetalipoproteinemia, familial, 2 (MIM: 605019) and are implicated as being protective against CAD.^52^ Finally, for OP, we selected the same osteogenesis imperfecta, type II (MIM: 166210) genes as used for BMD (*COL1A1* and *COL1A2*).

#### Gene curation for rare disorders

To broaden the scan for enrichment to more genes and objectively define gene sets associated with rare disorders, we used the evidence-based, expertly curated, and frequently reviewed Genomics England (GEL) PanelApp resource.^27^^,^^53^

When selecting gene sets associated with rare disorders within the GEL PanelApp, we selected disorders that cause extreme, early-onset, or monogenic forms of the phenotypes in our analysis. We only included genes rated as “green” (diagnosticgrade, conservatively defined) in the GEL PanelApp and excluded genes where the mode of pathogenicity was “other” or started with “loss-of-function variants (as defined in pop up message) DO NOT cause this phenotype.” We included genes with all models of inheritance, including both dominant and recessive inheritance models. This resulted in our final list of diagnostic-grade genes for each selected GEL rare disorder so that LoF variants in each gene were expected to cause the disorder.

### Misaligned phenotype classification

#### Continuous traits

To determine individuals whose observed continuous phenotypes are aligned or misaligned with their genetically predicted phenotype, we followed Hawkes et al*.*^26^ Briefly, covariates are residualized from the observed phenotype. Mahalanobis distance is calculated for each individual in the bivariate distribution formed by the residualized observed phenotype and the genetically predicted phenotype and converted to *p* values.^54^ Residualized observed phenotypes are then regressed against genetically predicted phenotypes, and residuals are calculated for each individual. We then classify an individual as misaligned or aligned if they satisfied the following conditions.^26^

##### Misaligned individuals

• Mahalanobis *p* value <0.001 and
• Absolute residual between residualized observed and expected phenotypes >2.

##### Aligned individuals

• Absolute difference between residualized observed and expected phenotypes <1.

In the first residualization step, the covariates were *sex*, *age*, *age*^*2*^, *sex* × *age*, *sex* × *age*^*2*^, the first 21 principal components (PCs) (calculated on UKB genotype array data^55^ and provided by the UKB), and UKB assessment center. Unlike Hawkes et al.,^26^ we did not account for statins in our main results when adjusting LDL-C for covariates to maintain standardized covariates across all traits. This omission is conservative in that it is more likely to result in false negatives than false positives, as we expect cholesterol-lowering medication to attenuate the misalignment effects of deleterious variants associated with hypercholesterolemia. Indeed, in a case study of adjusting LDL-C for cholesterol-lowering medication, power for canonical gene enrichment appears to be improved (Note S3).

For misaligned phenotype classification in sex-specific analyses, stratification by sex occurred as the first step, before any residualization. All downstream steps were the same as in the sex-combined analysis, apart from the exclusion of any sex-related covariates (i.e., *sex* and any of its cross terms) when performing covariate residualization.

#### Case-control dichotomous traits

To contrast individuals with aligned versus misaligned disease status, we set percentile thresholds on covariate-residualized PRS distributions (Figure S9). Percentile thresholds were chosen to compare the top versus bottom halves (threshold: 50%), quartiles (thresholds: 25%, 75%), and deciles (thresholds: 10%, 90%). This followed the approach of Lu et al.*,*^11^ who compared the top and bottom halves of the PRS distribution using point estimates for pathogenic variant prevalence. We included more extreme thresholds (quartiles, deciles) to assess whether enrichment increased in the extremes of the distribution.

#### Filtering to unrelated individuals

We excluded individuals with a high degree of relatedness (third degree or closer) when defining our aligned and misaligned individuals. Using relatedness estimates (*G*) from a genetic relatedness matrix (GRM) created by the scalable and accurate implementation of generalized mixed model (SAIGE)^56^ from genotype array data, we filtered to a maximal unrelated set of individuals. We created a greedy algorithm that iterated through a list of individuals (sorted by the number of relatives, with fewest relatives first) with at least one relative (*G* > 2^−3.5^; corresponding to relatives of the third degree or closer), adding their relatives to the list of individuals for removal. Individuals who had already been added to the list for removal were skipped if encountered later in the iteration. We performed this relatedness filter on each phenotype separately, pre-filtering to individuals with non-missing phenotype and covariate data before applying the relatedness filter, in order to maximize sample size for each phenotype.

### Enrichment testing of variant presence and misaligned status

#### Exact tests on contingency tables

To test whether individuals misaligned with genetic expectation are enriched for a binary variable (e.g., variant presence—whether an individual carries one or more damaging variants in a gene), we used Fisher’s exact tests,^57^ applied to 2 × 2 contingency tables.

#### Calculating empirical *p* values with simulated PGSs

Spurious misaligned enrichment is possible in our continuous trait misalignment testing framework, whether due to a phenotype effect that is stronger than the misaligned effect, a PGS that explains limited phenotype variability, or a combination of both (Figure S2). To avoid these possibilities, we calculate an empirical *p* value.

To establish empirical null distributions for enrichment test statistics, we tested the misalignment enrichment method using simulated PGSs. The simulated PGSs were generated independent of phenotypes, which means the expected phenotypic variance explained is zero. The PGSs were simulated from a multivariate standard normal distribution with zero covariance between individuals.

For each real (using non-simulated PGS) enrichment test in which individuals harbored deleterious variants in the gene(s) tested, we estimated the empirical null by generating replicates of enrichment test statistics based on simulated PGSs rather than real PGSs (Figure S2). This involved running the misalignment classification procedure using simulated PGSs instead of the real PGSs and then testing for enrichment of deleterious variants in the misaligned individuals. The empirical *p* value was calculated by the following equation:

$$P_{\text{emp}}=\frac{r+1}{n+1}\text{,}$$

where *n* is the number of test statistic replicates drawn from the null, and *r* is the number of replicate test statistics that are greater than or equal to the observed test statistic.^58^^,^^59^ We used −log_10_(one-sided Fisher’s exact test *p*) as our test statistic when calculating the empirical *p* value. We did not exclude replicates where misaligned individuals lacked the variants we were testing; instead, we assigned a Fisher’s exact test *p* = 1.

We simulated 10,000 replicates for all false discovery rate (FDR)-adjusted significant trait-gene-consequence combinations from enrichment tests of canonical and GEL PanelApp green genes. For significant genes resulting from the exome-wide scan, we simulated 1,000 replicates, using this reduced number of replicates due to the high computational and financial cost when running replicates on the DNAnexus Research Analysis Platform (RAP) for a large number of significant genes. The main downside of only using 1,000 replicates for the significant genes from the exome-wide scan instead of 10,000 is that the empirical *p* value can be no less than $\frac{1}{1,000+1}$ (9.99 × 10^−4^) rather than $\frac{1}{10,000+1}$ (1.00 × 10^−4^).

### Hypergeometric test for the probability of observing no variant presence

When testing misaligned individuals for enrichment of rare (MAF <0.1%) pLoF variants in genes associated with monogenic disorders, there were many instances where, due to the rarity of being misaligned compounded with the rarity of harboring rare pLoF variants, there were no misaligned individuals with rare pLoF variants. To quantify the high likelihood of observing no overlap in misaligned status and harboring a pLoF variant, we used the hypergeometric distribution to calculate the probability of observing no variant presence (*k* = 0) in a sample of *n* individuals (misaligned individuals) from a larger group of *N* individuals (aligned and misaligned individuals) with *K* individuals harboring a pLoF variant:

$$P\left(k=0;n,N,K\right)=\frac{\left(\begin{array}{c} K\\ 0 \end{array}\right)\left(\begin{array}{c} N-K\\ n \end{array}\right)}{\left(\begin{array}{c} N\\ n \end{array}\right)}=\frac{\left(\begin{array}{c} N-K\\ n \end{array}\right)}{\left(\begin{array}{c} N\\ n \end{array}\right)}\text{.}$$

### Burden testing on phenotype misalignment

We used REGENIE version 4.1^60^ to perform gene-level rare-variant (MAF < 0.1%) burden testing of phenotype misalignment for continuous (for exome-wide scan) and dichotomous traits (for canonical and GEL PanelApp green gene enrichment tests and exome-wide scan), using individuals with European genetic ancestry in the ES 450K tranche of the UKB (Table 2).

**Table 2:** REGENIE burden testing overview

**Trait type**	**REGENIE used for misaligned phenotype testing in…**	**Variant consequence burden masks**	**Dependent variable in regression**		**Hypothesized direction of burden effect**
Continuous	exome-wide scan	• pLoF • damaging missense	misaligned status [encoding]	high misaligned [1] vs. aligned [0]	positive (OR > 1)
				low misaligned [1] vs. aligned [0]	positive (OR > 1)
Dichotomous (disease status)	• canonical gene enrichment • GEL gene enrichment • exome-wide scan	pLoF	PRS’ (covariate-residualized PRS)	PRS’ in cases	negative
				PRS’ in controls	positive

Burden testing with REGENIE was performed for both continuous and dichotomous trait misalignment testing. In continuous traits, it was only used for the exome-wide scan, while in dichotomous traits, it was used for all three gene set categories. We tested both pLoF and putatively damaging missense variants for continuous traits but only pLoF for dichotomous traits. Misalignment testing in continuous traits treated the misaligned status as a dichotomous variable, with misaligned individuals always encoded as a 1 and aligned as 0. Misalignment testing in dichotomous traits was performed on covariate-residualized PRSs, a continuous variable. We use one-sided hypothesis tests throughout. For continuous traits, we always test for higher burden in misaligned individuals; for dichotomous traits, we test for a negative burden association in cases and a positive burden association in controls.

In REGENIE step 1, we fit the null model using UKB genotype array data QCed with PLINK^61^ (filtering flags: –maf 0.01 –mac 100 –geno 0.1 –mind 0.1). We included sequencing tranche, the tranche in which an individual was exome sequenced, as the only covariate. All other standard covariates (e.g., age, sex, PCs) had already been regressed out of the observed phenotype (continuous traits) or out of the PRS (dichotomous traits).

After fitting the null model in step 1, we carried out gene-level burden testing in REGENIE step 2 for pLoF (continuous and dichotomous traits) and damaging missense (continuous traits only) variants with MAF < 0.1% using the UKB ES 450K tranche data.

To test for an association between rare deleterious variation and misaligned status in continuous traits, we treated misaligned status as a dichotomous phenotype (using the --bt flag in REGENIE). We tested high misaligned vs. aligned and low misaligned vs. aligned separately. When testing misalignment in disease status, we did not apply inverse rank-normal transformation (IRNT) to residualized PRSs within REGENIE because the PRSs were already normalized.

To compare the statistical power in testing misaligned individuals for gene enrichment to traditional approaches, we ran gene burden tests on the original continuous and dichotomous phenotypes used as input to the misaligned phenotype classification framework. We applied IRNT within REGENIE to all continuous phenotypes using the --apply-rint flag and used the --bt flag for dichotomous traits.

For sex-specific analyses of canonical and GEL PanelApp green genes in dichotomous traits, we performed linear regression using statsmodels 0.14.6 in Python to test the association of pLoF burden association with PRSs, using the same regression model we had applied with REGENIE.

#### Conditioning on common imputed variants when testing misaligned disease status

When testing rare-variant burden effects on common-variant PRSs for misaligned disease status, there is a possibility that PRSs could correlate with rare variants at a given gene due to linkage disequilibrium (LD). This would lead to spurious burden associations. To conditionally orthogonalize the PRS from rare-variant burden, we conditioned on common (MAF > 0.1%) imputed v3 variants located in or within a 1 Mb radius of gene(s) tested (LD in Europeans tends not to extend beyond 1 Mb^30^), with gene start and stop locations in GRCh37 coordinates to match the imputed v3 variants. We used PLINK to QC these variants, following similar filtering thresholds used to select variants that generated the PRSs^25^ (PLINK filtering flags: –maf 0.001 –hwe 1e-10, variants with INFO score < 0.8 excluded). We conditioned on these QCed variants in REGENIE step 2 by passing a gene-specific (or grouped-gene) PLINK pgen file with the flag –condition-file. In our exome-wide scan for misaligned disease status, we performed this conditioning on FDR-significant genes to check the robustness of our results.

### Defining significant associations

For each stage of enrichment tests, we applied the Benjamini-Hochberg (BH) procedure to all associations, adjusting for FDR ≤ 5%. Statistically significant associations were defined as having FDR-adjusted *p* < 0.05. As our gene sets (canonical genes, GEL PanelApp green gene panels, exome-wide scan) overlapped, we excluded previously tested genes from consideration when calculating FDR-adjusted *p* values. After FDR correction, we filtered results on gene-level allele counts to avoid spurious associations in genes with low allele counts. For continuous trait misaligned status testing, only genes with minor-allele count (MAC) ≥ 5 in the misaligned group being tested were retained. For dichotomous trait misaligned disease status testing, only genes with alternate allele count (AAC) ≥ 10 were retained.

### Ethical approval

This study used data from the UKB. The North West Multi-centre Research Ethics Committee (MREC) gave approval to UKB as a research tissue bank (RTB) approval. This approval means that researchers do not require separate ethical clearance and can operate under the RTB approval.

## Results

### Identifying individuals with misaligned continuous phenotypes

Individuals were classified as deviating from their genetically expected phenotype if their observed phenotype, adjusted for covariates, was a significant outlier in the bivariate distribution of phenotype and PGS and if the covariate-residualized observed phenotype did not align with a linear model for observed phenotype and PGS (methods). We applied this method to seven continuous traits in the UKB, with the percentage of misaligned individuals ranging from 0.07% (HbA1c) to 0.12% (AAM) (Figure 1; Table S1; Figure S1).

### Individuals with misaligned continuous phenotypes are enriched for predicted LoF variants in canonical genes associated with monogenic disorders

Following categorization into aligned and misaligned individuals, misaligned individuals can be further split into those whose covariate-residualized observed phenotype is higher than expected (“high misaligned”) and lower than expected (“low misaligned”) relative to their PGS. In our enrichment tests, we tested for enrichment in one of the misaligned groups (either high or low misaligned) against the aligned group, with the aligned group acting as a control group (methods; Figure 1).

We are interested in finding genes that significantly contribute to phenotype misalignment. Due to the sparsity of individuals harboring rare deleterious variants, individuals with misaligned status, and the overlap of these two groups, we wish to control the possibility of spurious associations. Misaligned individuals also tend to have extreme covariate-residualized phenotypes. As the canonical and GEL PanelApp green genes we test are known to associate with the traits tested, we expect individuals harboring rare deleterious variants in these genes to cluster among individuals with extreme covariate-residualized phenotypes (high or low extremes depending on the effect direction of variants in the gene). Thus, we want to be sure that the misaligned associations we observe are distinct from extreme phenotype associations. Related to this, if a PGS explains limited phenotypic variability, then the slope of the phenotype-PGS regression will be close to zero. This would result in phenotype-PGS residuals that directly correspond to the covariate-residualized phenotype. Thus, the misalignment enrichment test would reduce to a test on the phenotype, an issue similar to the overlap between misaligned individuals and individuals with extreme covariate-residualized phenotypes.

To increase confidence that any continuous trait misalignment enrichment is distinct from extreme phenotype association and that the PGS explains a sufficient amount of phenotypic variability, we calculated an empirical *p* value for each putatively significant enrichment result by comparing the observed test statistic (one-sided Fisher’s exact test *p* value) against an empirical null distribution of test statistics (methods; Figure S2).

We tested misaligned individuals for enrichment of rare (MAF < 0.1%) pLoF variants (see methods for variant consequence definitions) in genes associated with monogenic disorders relevant to the misaligned phenotype (methods).

#### Height and LDL-C

**Figure 2:**
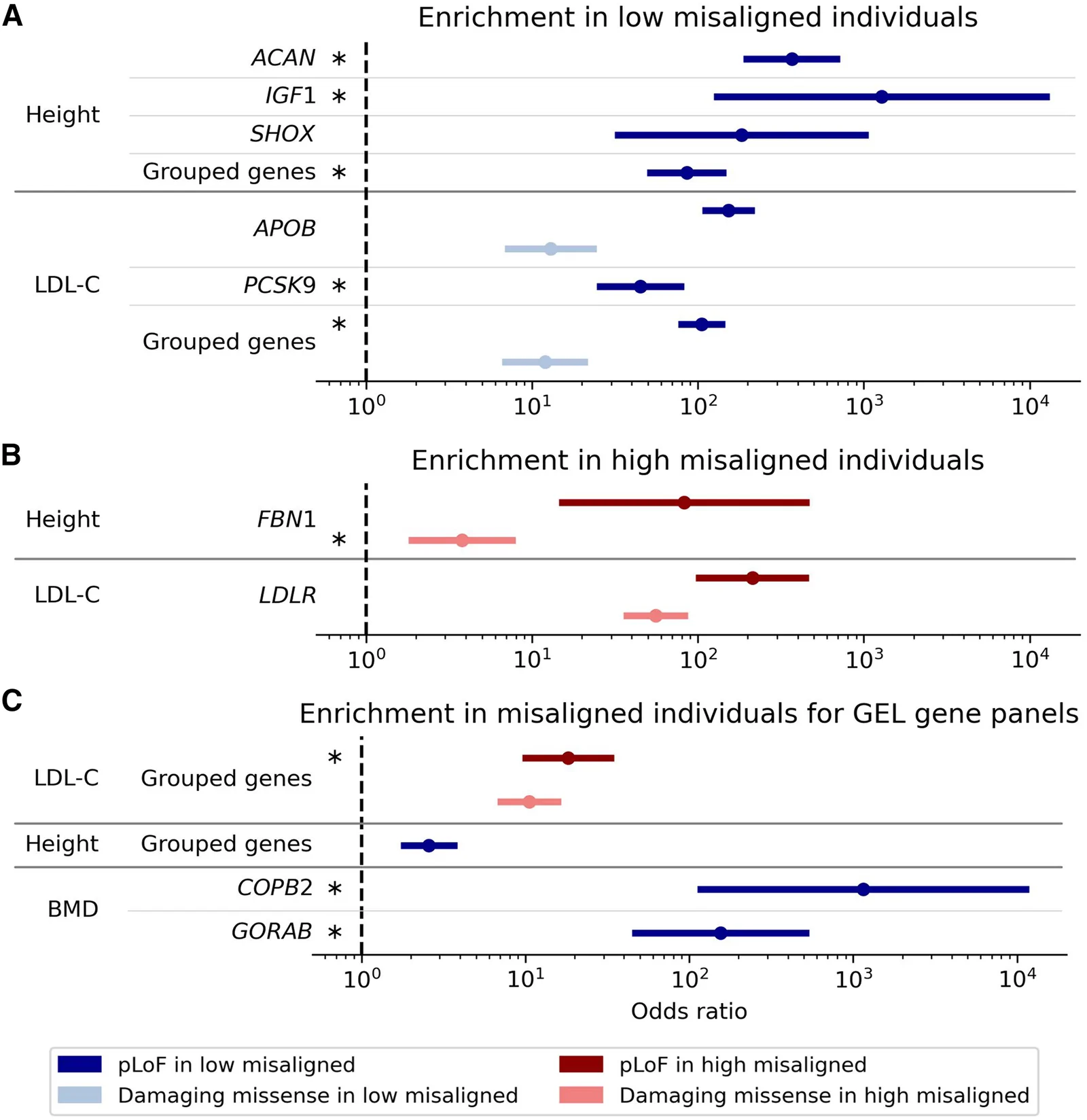
Enrichment of pLoF and damaging missense variants in canonical and GEL PanelApp green genes Shown is enrichment in (A) low and (B) high misaligned individuals for canonical genes associated with monogenic disorders and (C) misaligned individuals for GEL gene panels. “Low misaligned” individuals are individuals with phenotypes lower than genetically expected by PGS. “High misaligned” individuals are individuals with phenotypes higher than genetically expected by PGS. Error bars indicate two-sided 90% CI for OR so that the lower bound indicates the lower bound for a one-sided 95% CI. Only significant enrichment results (FDR ≤ 5%) are shown. Asterisks indicate results where the empirical *p* value is significant (*p*_emp_ < 0.05). Empirical null distributions can be seen in Figure S5.

Grouping variants across canonical genes associated with monogenic stature disorders (Table 1), we observed significant enrichment (FDR-adjusted one-sided Fisher’s exact test *p*_adj_ ≤ 0.05) of pLoF variants in individuals with shorter-than-expected stature (odds ratio (OR) [90% confidence interval (CI)] = 85.7 [49.4–149], *p*_adj_ = 1.13 × 10^−15^) (Figure 2; Table S2). We also tested single genes and observed significant enrichment in individuals with shorter-than-expected stature for *ACAN* (OR [90% CI] = 367 [188–717], *p*_adj_ = 3.02 × 10^−17^; MIM: 155760), *IGF1* (OR [90% CI] = 1,280 [125–13,200], *p*_adj_ = 4.08 × 10^−3^; MIM: 147440), and *SHOX* (OR [90% CI] = 183 [31.4–1,070], *p*_adj_ = 0.0148; MIM: 312865) (Figure 2; Table S2).

When testing a set of genes associated with FH (Table 1), we found that individuals with high or low LDL-C were enriched for pLoF variants in genes where LoF is known to cause high (*LDLR* OR [90% CI] = 213 [96.8–468], *p*_adj_ = 3.31 × 10^−10^) or low LDL-C (*APOB* OR [90% CI] = 153 [106–220], *p*_adj_ = 9.45 × 10^−45^; *PCSK9* OR [90% CI] = 45.0 [24.6–82.4], *p*_adj_ = 1.29 × 10^−10^), respectively (Figure 2; Table S2). Individuals with lower than genetically expected LDL-C were also enriched for harboring the combined burden of *PCSK9* and *APOB* pLoF variants (OR [90% CI] = 105 [75.9–146], *p*_adj_ = 5.86 × 10^−51^) (Figure 2; Table S2).

Sex-specific analyses recapitulated at nominal significance (*p* < 0.05) all sex-combined associations for height and LDL-C (Figure S6; Table S2).

#### Additional continuous phenotypes

For other continuous phenotypes, no misaligned individuals harbored pLoF variants in canonical genes associated with monogenic disorders (Figure S3; Table 1), likely due to the extremely low frequency of these variants in a population cohort such as the UKB. In each trait-gene pair, there was a relatively high probability (minimum of hypergeometric test *P*(*k* = 0) = 0.83; methods) of observing zero pLoF variants in the set of misaligned individuals, given the number of individuals harboring pLoF variants in the group of misaligned and aligned individuals tested.

Missense variants that are deleterious but not predicted to result in LoF (“damaging missense” variants; methods) are by definition expected to have less of an effect on gene product than pLoFs. Assuming a causal link between gene product and phenotype, this implies that damaging missense variants are also expected to have attenuated effect sizes on the phenotype compared to pLoF variants. Although effect size is generally attenuated for damaging missense variants, they tend to occur at higher frequencies. For example, the aggregated pLoF allele count across genes tested for enrichment was 3,114; for damaging missense alleles, the count was 17,984, almost 6-fold higher. We hypothesized that testing damaging missense variants instead might boost power in enrichment testing by increasing the number of individuals harboring putatively damaging variation, albeit at smaller effect size, among misaligned individuals.

### Individuals with misaligned continuous phenotypes are enriched for predicted damaging missense variants in genes associated with monogenic disorders

We tested for enrichment of damaging missense variants in misaligned individuals with the same methods as for pLoF variants, for the same set of continuous traits and corresponding genes associated with monogenic disorders.

We observed a significant enrichment (Fisher’s exact test FDR-adjusted *p*_adj_ ≤ 0.05) of damaging missense variants for four of the nine genes/gene sets where misaligned individuals were enriched for pLoF burden (Figure 2; Table S2). Individuals with higher than genetically expected height were enriched for damaging missense variants in *FBN1* (OR [90% CI] = 3.79 [1.80–7.97], *p*_adj_ = 0.0254; MIM: 134797). For LDL-C, high misaligned individuals were enriched for damaging missense variants in *LDLR* (OR [90% CI] = 55.7 [35.5–87.4], *p*_adj_ = 3.09 × 10^−21^), while low misaligned individuals were enriched for damaging missense variants in *APOB* (OR [90% CI] = 13.0 [6.87–24.6], *p*_adj_ = 5.64 × 10^−6^) and grouped *APOB* and *PCSK9* variants (OR [90% CI] = 12.0 [6.59–21.8], *p*_adj_ = 2.18 × 10^−8^).

All sex-combined FDR-significant results were recapitulated at nominal significance (*p* < 0.05) by at least one sex-specific analysis (Figure S6; Table S3). We observed one nominally significant single-gene result unique to the sex-specific analysis: enrichment of damaging missense variants in *POMC* (MIM: 176830) among males with higher than genetically expected BMI (OR [90% CI] = 17.3 [5.25–56.9], *p* = 6.62 × 10^−3^).

As expected, given that we are testing damaging missense variants, which are predicted (methods) to have a less deleterious effect than pLoF variants, the enrichment of damaging missense variants consistently had lower OR point estimates than pLoF variants (4/4 single- and grouped-gene tests with FDR ≤5% significant pLoF and damaging missense enrichment; Figure 2).

### Individuals whose continuous phenotype is misaligned with genetic expectation are enriched for predicted damaging variants in genes related to rare disorders

Having demonstrated that misaligned individuals are enriched for both pLoF and damaging missense variation in canonical genes associated with monogenic disorders for continuous traits, we expanded the set of genes analyzed to include genes associated with other rare disorders related to the traits analyzed. To do this, we extracted genes from the expertly reviewed GEL PanelApp gene sets^27^^,^^53^ for rare disorders relevant to the phenotypes included in our analysis (methods). We restricted analysis to genes that qualified for the highest-confidence category (green), designated to be of diagnostic grade.^27^^,^^53^

We tested all continuous traits for enrichment of pLoF and damaging missense variants in GEL PanelApp green genes in misaligned individuals. We used the same enrichment testing framework as described above for the canonical genes associated with monogenic disorders. We excluded 7.69% (38/494) of single- and grouped-gene masks because they had already been included in the enrichment testing of canonical genes. We performed the BH procedure^62^ to control FDR at 5% across all pLoF and damaging missense enrichment tests for continuous traits.

There were four single- and grouped-gene masks across three traits (height, LDL-C, and BMD) for which misaligned individuals were significantly enriched (Fisher’s exact test FDR-adjusted *p*_adj_ ≤ 0.05) for pLoF or damaging missense variants (Figure 2; Figure S4; Table S2).

Individuals with lower than genetically expected BMD were enriched for pLoF variants in *COPB2* (OR [90% CI] = 1,150 [112–11,900], *p*_adj_ = 0.0325; MIM: 606990) and *GORAB* (OR [90% CI] = 155 [44.5–539], *p*_adj_ = 3.17 × 10^−3^; MIM: 607983), genes selected from the osteogenesis imperfecta gene panel in the GEL PanelApp (Figure 2; Table S2). Although the presence of pLoF variants in the low misaligned individuals was extremely sparse (*COPB2 n* = 1, *GORAB n* = 2), both of these genes demonstrated significant empirical *p* values (*p*_emp_ < 0.05) (Figure S5), reinforcing the possibility of a true misalignment effect. The enrichment in *GORAB* was also supported by nominally significant (*p* < 0.05) enrichment in a male-only analysis (OR [90% CI] = 107 [18.8–609], *p* = 0.0104) but was also driven by only one individual harboring a pLoF variant in the misaligned group.

Individuals with higher than genetically expected LDL-C and individuals with lower-than-expected height were also enriched for deleterious variant burden in grouped GEL PanelApp genes. However, these significant grouped results were driven largely by variants in the previously tested canonical genes, with attenuated ORs compared to the results of the grouped canonical genes (Figure 2; Table S2).

#### Association of non-genetic factors with continuous trait misalignment

Although the primary focus of our work is identifying genes associated with phenotype misalignment, we tested whether misaligned status was associated with non-genetic factors, such as socioeconomic status (SES), medication, smoking status, and exercise (Note S1; Figure S7; Table S4). We observed nominal association (one-sided Wald test *p* < 0.05) of SES with pathogenic misalignment (defined as trait increasing, except for BMD and AAM, where it is trait decreasing) for each continuous trait. We also observed a strong enrichment of cholesterol-lowering medication in individuals with lower than genetically expected LDL-C (OR [90% CI] = 11.7 [8.98–15.2], one-sided Fisher’s exact test *p* = 1.21 × 10^−53^). These results indicate that such non-genetic factors may also play a role in misalignment, as shown in previous work.^26^

### Individuals whose disease status is misaligned with genetically expected risk are enriched for predicted LoF variants in genes associated with monogenic disorders

We examined misaligned disease status relative to PRS in three common complex diseases: T2D, CAD, and OP. These diseases have been included in previous UKB studies involving PRS and penetrance of pathogenic variants.^11^^,^^13^ We tested the hypotheses that (1) disease cases with low PRS are enriched for pathogenic rare pLoF variants and (2) disease controls with high PRS are enriched for protective rare pLoF variants.

For each hypothesis, we took a two-step approach. First, within a given disease status group (cases or controls), we used linear regression to test the association of PRS with (pathogenic or protective) pLoF variant presence. Variant presence is a binary variable, defined as whether an individual carries at least one pLoF in the gene(s) tested. Second, similar to earlier work in a smaller subset of the UKB,^11^ we defined low and high PRS percentile groups and tested enrichment of pLoFs between the groups.

To validate our approach, we first applied it to binarized versions of height and LDL-C, continuous traits for which we have already observed an enrichment of rare deleterious variants in individuals whose phenotypes deviate from genetic expectation. Among simulated dichotomized traits with a case prevalence of 5% and 10% (similar to the common diseases we aim to study), we replicated all but one (8/9) FDR-significant single- and grouped-gene pLoF associations for misaligned height and LDL-C at nominal significance (one-sided *t* test *p* < 0.05) (Figure S8; Table S5). This demonstrated a proof of concept that our pLoF enrichment testing approach for dichotomous case-control traits could recapitulate similar pLoF enrichment in misaligned individuals observed for continuous traits.

#### Disease cases with low polygenic risk are enriched for rare pathogenic predicted LoF variants in genes associated with monogenic disorders

Among T2D, CAD, and OP cases, we tested for association of rare (MAF < 0.1%) pathogenic pLoF burden with lower PRS. We also tested for association of pLoF burden in low-PRS cases versus high-PRS cases. As with the enrichment testing of misaligned continuous traits, we used a curated set of canonical genes associated with monogenic disorders (Table 3; methods). Our burden tests were conditioned on common imputed variants in or within 1 Mb of each gene being tested (methods). We performed the BH^62^ procedure to control FDR at 5% separately for the regression association tests and Fisher’s exact tests.

**Table 3:** Canonical genes associated with monogenic disorders tested for pLoF enrichment in disease traits

**Disease**	**Associated disorder**	**Effect of LoF**	**Genes**
T2D	MODY^63^	pathogenic	*GCK*, *HNF1A*, *HNF4A*, *HNF1B*
	N/A	protective^51^	*SLC30A8*
CAD	familial hypercholesterolemia hypobetalipoproteinemia, familial, 2^52^	protective^64^^,^^65^^,^^66^	*APOB*, *PCSK9*
		pathogenic^67^	*LDLR*
		protective	*ANGPTL3*
OP	osteogenesis imperfecta, type II^68^	pathogenic	*COL1A1*, *COL1A2*

N/A, not applicable.

**Figure 3:**
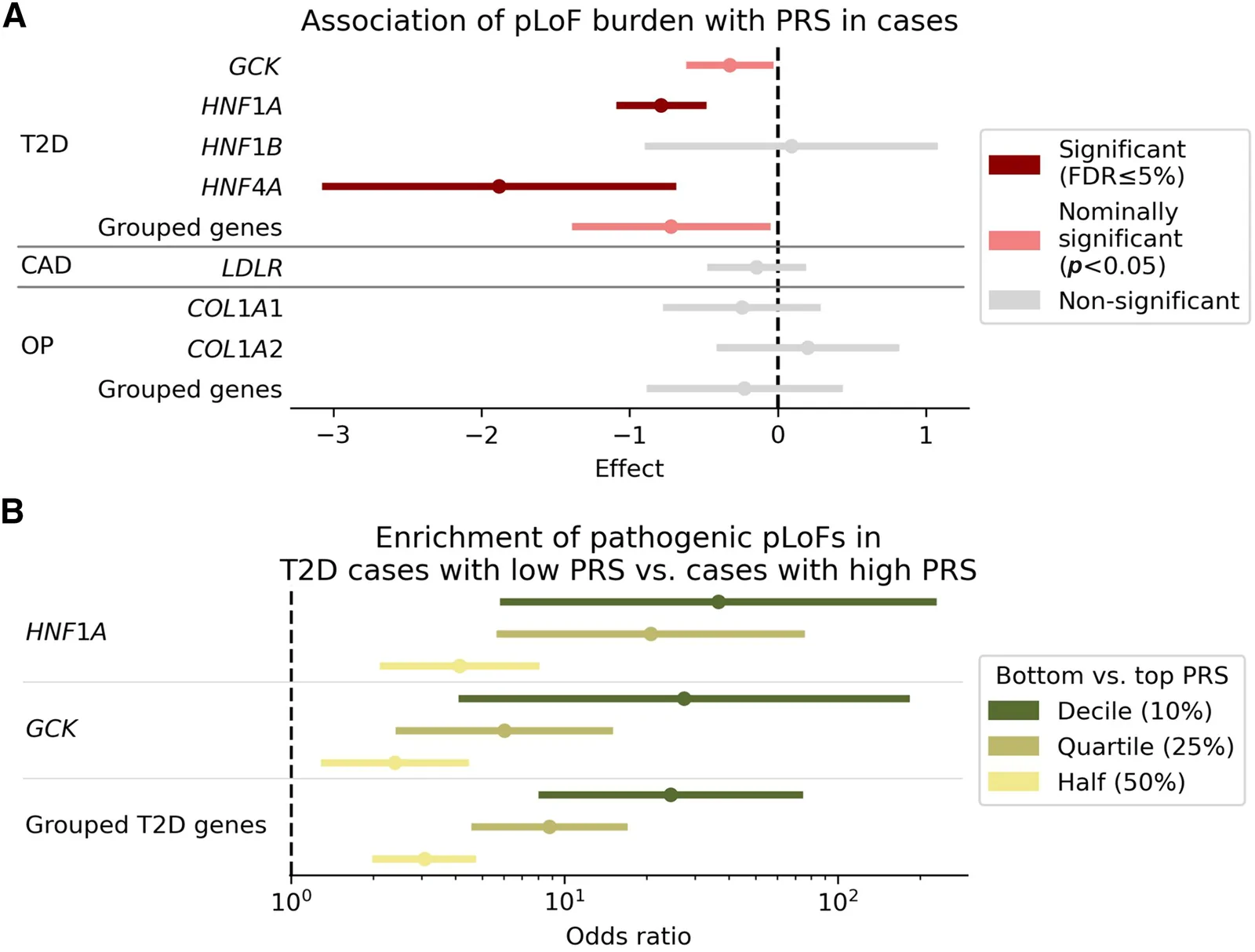
Pathogenic pLoFs are associated with lower PRS in disease cases (A) Effect of pLoF variants on PRS among disease cases. Association tests were conditioned on common (MAF > 0.1%) imputed variants in or within 1 Mb of each gene tested. Significant associations are defined by FDR-adjusted one-sided burden *p*_adj_ ≤ 0.05. Nominally significant associations are defined by one-sided burden *p* < 0.05. (B) Comparing pathogenic pLoF enrichment in extremes of PRS for disease cases, for genes with significant enrichment results in (A) except for *HNF4A*, which had insufficient individuals harboring pLoF variants to perform enrichment testing in extremes. Error bars indicate two-sided 90% CI for OR so that the upper bound in (A) or lower bound in (B) indicates the upper or lower bound, respectively, for a one-sided 95% CI. pLoF, predicted loss of function; PRS, polygenic risk score; CAD, coronary artery disease; OP, osteoporosis; T2D, type 2 diabetes.

In T2D cases, pLoF burden was significantly associated (FDR-adjusted one-sided burden *p*_adj_ ≤ 0.05) with lower PRS for burden in *HNF1A* (*β* [90% CI] = −0.788 [−1.09, −0.484], *p* = 1.03 × 10^−5^, *p*_adj_ = 1.44 × 10^−4^) and *HNF4A* (*β* [90% CI] = −1.88 [−3.08, −0.685], *p* = 4.83 × 10^−3^, *p*_adj_ = 0.0338), with nominally significant (one-sided *p* < 0.05) burden associations in *GCK* and grouped genes (Figure 3A; Table S6). The *HNF1A* result was supported by nominally significant (*p* < 0.05) male- and female-specific analyses, while the *HNF4A* result was driven solely by two female individuals harboring pLoF variants (*β* [90% CI] = −2.22 [−3.30, −1.15], *p* = 3.24 × 10^−4^) (Figure S10; Table S8).

When testing pLoF enrichment in low- vs. high-PRS T2D cases for these single- or grouped-gene masks, we observed a monotonic trend in increasing OR point estimates when comparing ever more extreme PRS quantiles (Figure 3B; Table S7). This suggests that enrichment of pLoFs increases as we compare individuals increasingly misaligned with genetic expectation (low-PRS cases) to individuals increasingly aligned with genetic expectation (high-PRS cases).

#### Disease controls with high polygenic risk are enriched for rare protective predicted LoF variants in genes associated with monogenic disorders

**Figure 4:**
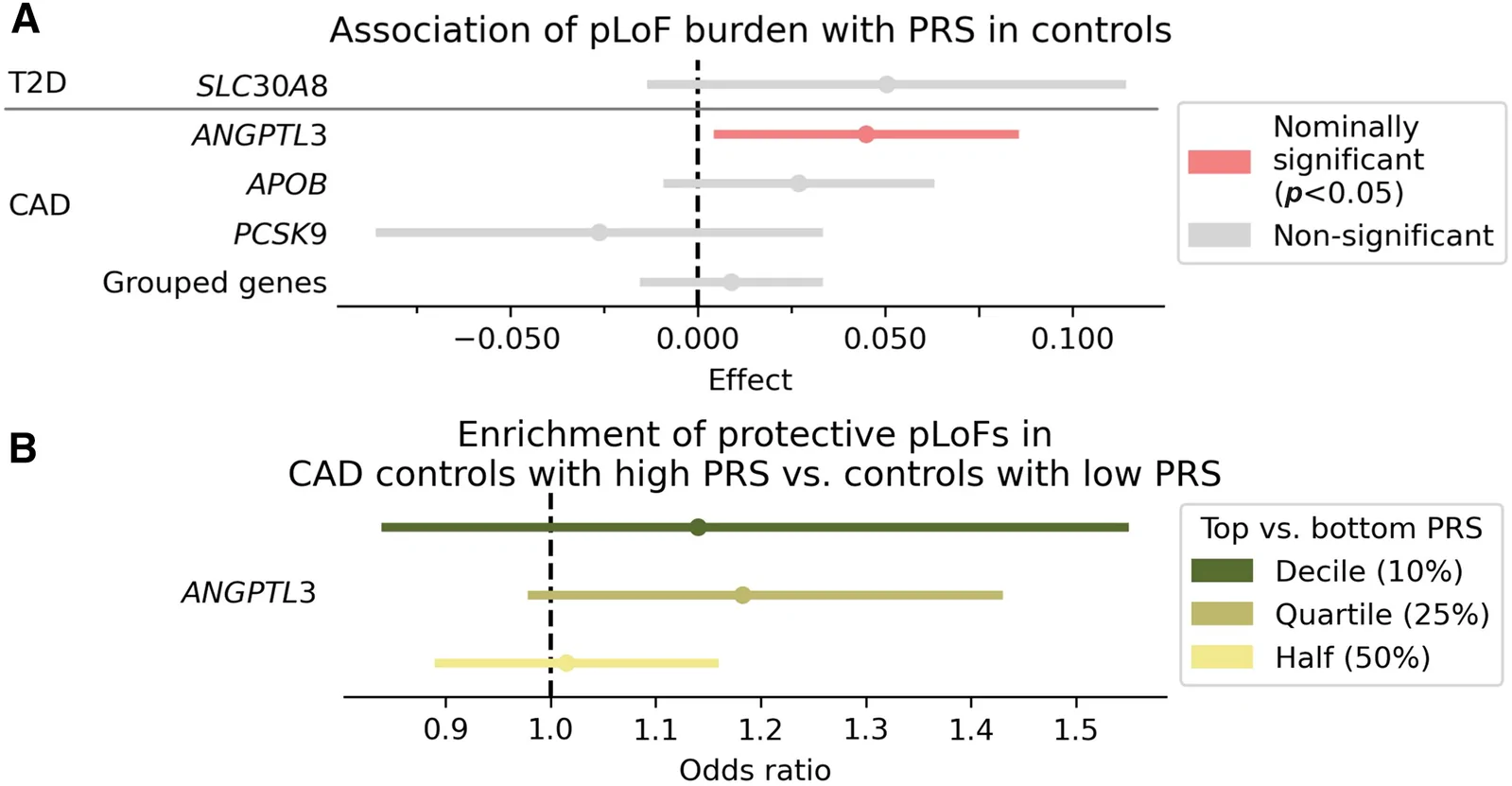
Protective pLoFs are associated with higher PRS in disease controls (A) Effect of pLoF variants on PRS among disease controls. Association tests were conditioned on common (MAF > 0.1%) imputed variants in or within 1 Mb of each gene tested. Nominally significant associations are indicated by one-sided burden *p* < 0.05. (B) Comparing pathogenic pLoF enrichment in extremes of PRS for disease controls for genes with significant enrichment results in (A). Error bars indicate two-sided 90% CI for OR so that the lower bound indicates the lower bound for a one-sided 95% CI.

We tested for enrichment of rare pLoF variants in *APOB*, *PCSK9*, and *ANGPTL3* for CAD controls and *SLC30A8* for T2D controls; these are genes where LoF is expected to be protective for the corresponding disease (Table 3; Figure 4A). Among CAD controls, we observed nominally significant (one-sided burden *p* < 0.05) association of pLoF burden in *ANGPTL3* with increased CAD PRS (*β* [90% CI] = 0.0449 [4.22 × 10^−3^, 0.0856], *p* = 0.0347, *p*_adj_ = 0.108). Sex-stratified analyses suggested that the *ANGPTL3* burden association is primarily driven by males (*β* [90% CI] = 0.145 [0.0500, 0.239], *p* = 5.97 × 10^−3^) rather than females (*β* [90% CI] = −3.19 × 10^−3^ [−0.0852, 0.0788], *p* = 0.526; Figure S10; Table S8). Comparing relative enrichment of pLoF variant presence in disease controls with high versus low PRS, OR point estimates did not demonstrate the same monotonicity as observed for pathogenic pLoFs in disease cases (Figure 4B; Table S7).

#### Limited evidence for enrichment of predicted LoF variants in GEL PanelApp green genes for disease cases with misaligned disease status

We extended our analysis in dichotomous trait misalignment from canonical genes to GEL PanelApp gene panels. There were no GEL PanelApp green genes where pLoF burden associated significantly (FDR-adjusted one-sided burden *p*_adj_ ≤ 0.05) with lower PRS in cases (Table S6). There were three genes with nominal associations: *BMP1* (MIM: 112264) for OP (*β* [90% CI] = −0.618 [−1.18, −0.0573], *p* = 0.0349), *LMNA* (MIM: 150330) for T2D (*β* [90% CI] = −0.567 [−1.12, −0.0117], *p* = 0.0465), and *LIPA* (MIM: 613497) for CAD (*β* [90% CI] = −0.479 [−0.956, −2.70 × 10^−3^], *p* = 0.0490).

#### Association of non-genetic factors with misalignment in disease status

As performed for the continuous traits, we also tested dichotomous trait misalignment for association with non-genetic factors (Note S1; Figure S7; Table S4). We observed a strong protective misaligned association (one-sided *t* test *p* < 2 × 10^−61^) for self-reported disease-specific medication across all three diseases. We also observed that T2D pathogenic and protective misalignments are associated with smoking status (*p* = 2.12 × 10^−4^) and exercise (*p* = 3.80 × 10^−7^), respectively (Note S1; Figure S7; Table S4).

#### Exome-wide scan for genes associated with misaligned status in continuous traits

Hypothesizing that there may be genes associated with misaligned status that were not included in the canonical or GEL PanelApp green gene sets, we scanned all genes for deleterious missense variation associated with misaligned status. Genes where deleterious missense variation causes individuals to have lower than genetically expected disease risk or lower-than-expected liability phenotype would be ideal targets for therapeutic deactivation. Conversely, a gene where missense variation is pathogenic could be therapeutically targeted for increased expression, assuming druggability of the gene.

For misaligned status of each continuous trait, we performed gene burden testing of rare (MAF < 0.1%) pLoF and damaging missense variants with REGENIE,^60^ testing separately for protective and pathogenic effects on phenotype misalignment (methods). Protective effects were defined as those that are trait decreasing, except for BMD and AAM, where protective effects are trait increasing. Pathogenic effects are defined as trait increasing, except for BMD and AAM, where they are trait-decreasing. For continuous phenotypes, these definitions are oriented by the diseases of interest for which the continuous phenotypes act as liability phenotypes. The exception was height, where protective effects were defined to be trait decreasing and pathogenic effects trait increasing. These directions were arbitrarily chosen, as there exist both disorders of short and tall stature. When testing misaligned status for protective or pathogenic effects, we always test for enrichment in the misaligned group corresponding to the effect on the original trait (lower-than-expected misaligned when effect is negative, higher-than-expected misaligned when effect is positive).

Burden test *p* values were controlled using the BH procedure^62^ for FDR ≤ 5% across all phenotypes and all genes, separately for protective and pathogenic misalignment tests, with previously tested canonical monogenic disorder and GEL PanelApp green genes excluded. Following FDR control, genes were filtered to those with MAC ≥ 5 in the misaligned group being tested to limit spurious associations. An empirical null *p* value was calculated for each significant gene enrichment result (Figure S12; methods).

#### Protective effect enrichment tests

**Figure 5:**
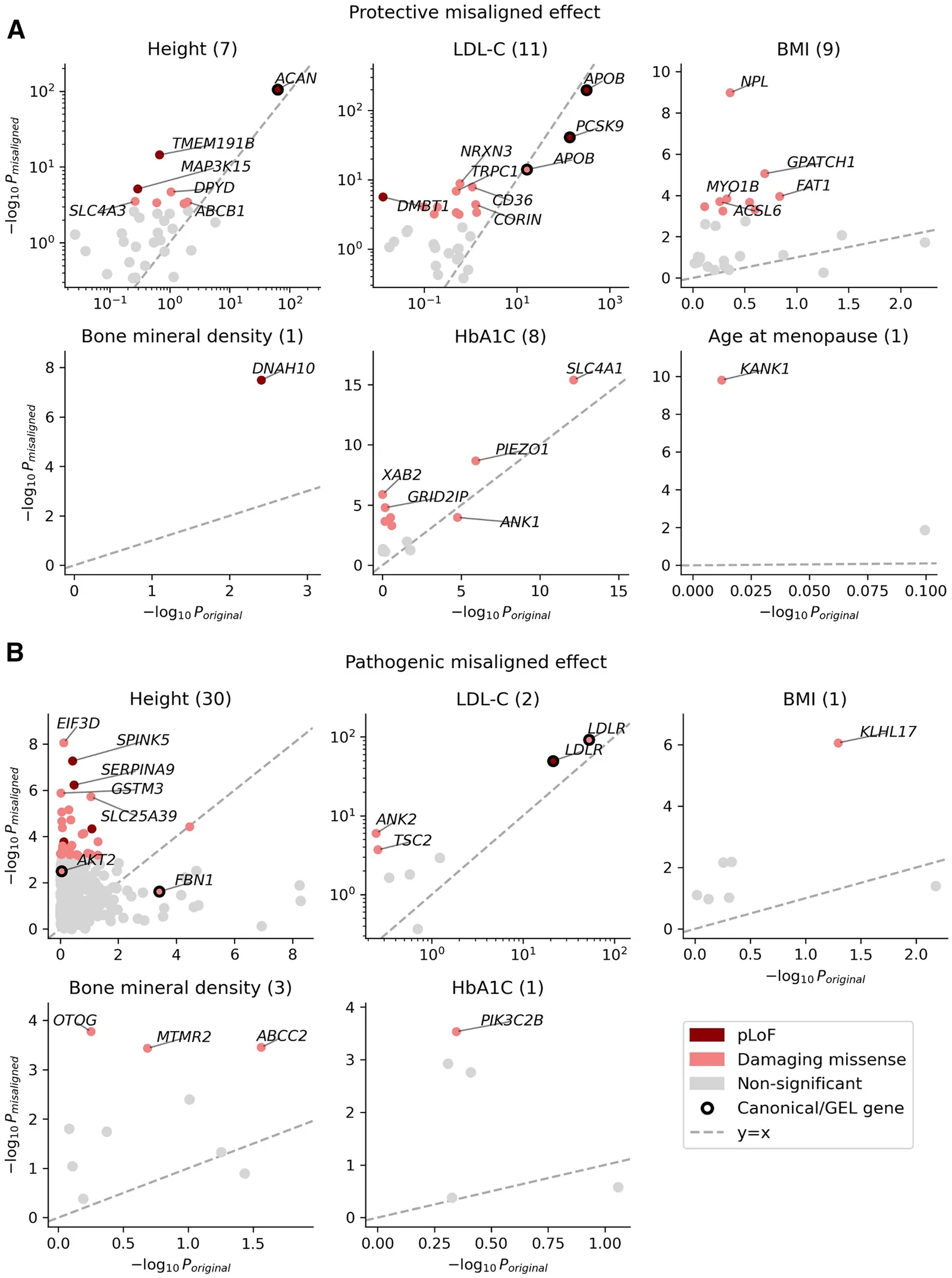
Strength of misaligned burden association, comparing burden test on original phenotype versus burden test of misaligned status (A) Protective misaligned effect. (B) Pathogenic misaligned effect. *p*_misaligned_ is a one-sided *p* value, with the alternate hypothesis that burden of damaging variants is higher in misaligned individuals (cases) than aligned individuals (controls). *p*_original_ is a two-sided *p* value for the burden association with the original continuous trait. Only genes with MAC_case_ ≥ 5 are shown. The top 5 FDR-significant genes with highest −log_10_*p*_misaligned_ are labeled with a gene symbol. Canonical and GEL PanelApp green genes are also labeled. The number of FDR-significant genes for each trait is indicated in parentheses in each subplot title. Protective misaligned status corresponds to low misaligned status and pathogenic misaligned status to high misaligned status, except for BMD and AAM, where the effects are flipped (protective = high misaligned, pathogenic = low misaligned).

In our exome-wide burden tests for “protective” misaligned status (lower-than-expected misalignment, except for BMD and AAM, for which protective misaligned status is higher-than-expected misalignment), 37 genes across six traits harbored significant enrichment (FDR-adjusted one-sided burden *p*_adj_ ≤ 0.05) of pLoF or damaging missense variants (Figure 5A; Table S9).

For lower-than-expected BMI misalignment (9 significant genes), damaging missense associations with plausible biological mechanisms included two genes with links to fat metabolism: *ACSL6* (burden *p*_misaligned_ = 1.99 × 10^−4^, *p*_adj_ = 0.0182, *p*_emp_ = 0.106; Figure 5A; Table S9; MIM: 604443), involved in lipid synthesis, so that knockdown in rat skeletal muscle results in decreased lipid accumulation,^69^ and *FAT1* (burden *p*_misaligned_ = 1.12 × 10^−4^, *p*_adj_ = 0.0116, *p*_emp_ = 0.0260; MIM: 600976), involved in fatty acid conversion.^70^ The gene with the strongest association, *NPL* (burden *p*_misaligned_ = 1.06 × 10^−9^, *p*_adj_ = 6.94 × 10^−7^, *p*_emp_ = 9.99 × 10^−4^; MIM: 611412), has prior experimental evidence supporting a plausible link with decreased BMI. Deficiency of NPL has been shown to result in loss of skeletal muscle in humans, zebrafish, and mice.^71^ This loss of muscle mass could lead to lower-than-expected BMI. All three plausible genes have LoF observed/expected upper bound fraction (LOEUF) scores above the Genome Aggregation Database (gnomAD)^32^ v.4.1.1 heuristic threshold of 0.45, which suggests that they are likely to be tolerant to LoF. Likewise, none of these three genes are directly implicated in severe disorders in Online Mendelian Inheritance in Man (OMIM) (https://omim.org/). Both of these lines of evidence point to the possibility of therapeutic gene perturbation without lethal consequences. Of the three genes, *ACSL6* is the only one with evidence of knockdown directly influencing lipid accumulation, a mechanism central to obesity. Investigating its therapeutic tractability in Open Targets,^72^ it is predicted to have a signal peptide or transmembrane regions, which makes it more amenable to antibody-based therapeutics.

Individuals with higher-than-expected AAM were enriched for damaging missense variants in *KANK1* (burden *p*_misaligned_ = 1.55 × 10^−10^, *p*_adj_ = 1.27 × 10^−7^, *p*_emp_ = 3.00 × 10^−3^; Figure 5A; Table S9; MIM: 607704). When assessing the original trait, association of damaging missense burden in *KANK1* with AAM was null in our study (two-sided burden *p* = 0.964) and in Genebass^28^ (missense|low-confidence LoF (LC) burden *p* = 0.879). *KANK1* is associated with uterine fibroid risk,^73^ much like *CHEK2*, a gene associated with tumor suppression,^74^ where LoF has previously been shown to be associated with increased AAM.^75^ Uterine fibroids become less common after menopause,^76^ so if damaging variation in a gene was protective for POI, increasing AAM, then it might extend the window of time for uterine fibroids to appear, increasing the odds of developing uterine fibroids. *KANK1* is also implicated in the Reactome^77^ pathway “estrogen-dependent gene expression,” with participation in the reaction “estrogen-responsive *CXXC5* gene expression.” There is a strong link between menopause and estrogen,^78^ with estrogen levels typically declining due to menopause. If *KANK1* were to be causally implicated in menopause, then it may also be implicated in the decline of estrogen associated with menopause. Regarding its viability as a therapeutic target, *KANK1* is not under strong selective constraint (LOEUF = 1.2; gnomAD^32^ v.4.1.1), which suggests that it may be perturbable without patient lethality. Although heterozygous full-gene deletion of *KANK1* has been linked to congenital cerebral palsy,^79^ this was a case study of a single family, and only individuals who inherited the full-gene deletion from their parents were affected. There is no evidence that damaging missense variants would have such an effect. Furthermore, therapeutic tractability evidence collated by Open Targets suggests that *KANK1* may be an accessible target for antibody-based therapies; three collated sources (UniProt, Gene Ontology, and the Human Protein Atlas) predict with high confidence that the gene location is at the plasma membrane or extracellular region or is secreted by the cell.

For lower-than-expected HbA1c misalignment (8 significant genes), we replicated several known HbA1c associations, including *PIEZO1*^80^ (MIM: 611184), and two genes (*SLC4A1* [MIM: 109270] and *ANK1* [MIM: 612641]) linked with hereditary spherocytosis,^81^ a disorder that can lead to low HbA1c measurements.^82^

#### Pathogenic effect enrichment tests

In our exome-wide burden tests for “pathogenic” misaligned status (higher-than-expected misalignment, except for BMD and AAM, for which pathogenic misaligned status is lower-than-expected misalignment), 37 genes across five traits harbored significant enrichment (FDR-adjusted one-sided burden *p*_adj_ ≤ 0.05) of pLoF or damaging missense variants (Figure 5B; Table S9).

For taller-than-expected height, significant enrichment was observed for both pLoF (4 genes) and damaging missense variants (26 genes). Several of the significant genes are associated with autosomal-recessive genetic syndromes that can result in short stature, including *SPINK5* (MIM: 605010; Netherton syndrome^83^ [MIM: 256500]), *PLOD2* (MIM: 601865; Bruck syndrome 2^84^ [MIM: 609220]), and *GALNS* (MIM: 612222; mucopolysaccharidosis, type IVA^85^ [MIM: 253000]). These are counter to the direction of effect expected for taller-than-expected height misalignment; however, none of the taller-than-expected height outliers were homozygous or compound heterozygous for ClinVar^86^ pathogenic variants from the variant category (pLoF or damaging missense) being tested (Figure S11). This suggests that these carriers are unlikely to have these syndromes, resolving this paradox.

For higher-than expected LDL-C (2 significant genes), one gene with a biologically plausible mechanism was *TSC2* (burden *p*_misaligned_ = 2.01 × 10^−4^, *p*_adj_ = 0.0188, *p*_emp_ = 4.00 × 10^−3^; Figure 5B; Table S9; MIM: 191092). *TSC2* inhibits the protein kinase mechanistic target of rapamycin complex 1 (mTORC1)^87^; when mTORC1 is left uninhibited, it can promote *de novo* lipogenesis.^88^ The other significant gene, *ANK2* (burden *p*_misaligned_ = 1.09 × 10^−6^, *p*_adj_ = 2.96 × 10^−4^; MIM: 106410), is implicated in cardiac arrhythmia, ankyrin-B related^89^ (MIM: 600919), but no evidence in the literature suggests that cardiac arrhythmia can cause high cholesterol.

For higher-than-expected HbA1c misalignment, the only significant association was for damaging missense burden in *PIK3C2B* (burden *p*_misaligned_ = 2.93 × 10^−4^, *p*_adj_ = 0.0252, *p*_emp_ = 0.218; Figure 5B; Table S9; MIM: 602838). There is a plausible pathway implicating this gene in HbA1c, as it encodes a protein involved in the phosphatidylinositol 3-kinase (PI3K)/AKT/mTOR pathway, a critical regulator of insulin signaling and overall glucose homeostasis.^90^^,^^91^

For the remaining traits, there were significant pathogenic damaging missense burden associations for higher-than-expected BMI (*KLHL17* [MIM: 619262]) and lower-than-expected BMD (*OTOG* [MIM: 604487], *ABCC2* [MIM: 601107], and *MTMR2* [MIM: 603557]). However, these genes did not have strong evidence from existing literature to support their biological plausibility.

## Discussion

In this study, we tested a collection of continuous and dichotomous traits for enrichment of rare deleterious variants in individuals whose observed phenotype deviates from common-variant genetic expectation. We began with a narrow focus, testing a small curated set of genes canonically implicated in each trait, before expanding to testing larger gene panels from GEL for rare disorders related to the traits. Finally, we broadened our scope to an exome-wide scan, searching for gene burden associations with phenotype misalignment.

Using an existing method of misaligned phenotype classification in continuous traits, we replicated many of the pLoF enrichment results previously observed^26^ for misaligned height and LDL-C. We also found that individuals with lower than genetically expected LDL-C and higher than genetically expected stature were enriched for rare damaging missense variants in *APOB* and *FBN1*, respectively. This suggests that phenotype misalignment may be influenced by variants expected to have more moderate effects on gene function. Another observation was that, in BMD, a trait not investigated by previous studies of misaligned phenotypes, misaligned individuals were enriched for rare pLoF variants in *COPB2* and *GORAB*, genes linked with a monogenic form of OP. These results further validate the use of misalignment classification for prioritizing individuals when screening for rare genetic disorders related to height, LDL-C, and BMD.

Testing for pLoF enrichment in individuals misaligned with disease status, we found that T2D disease cases harboring pathogenic rare pLoFs in certain genes associated with MODY tended to have significantly lower PRSs. We also observed that CAD disease controls harboring nominally significant protective rare pLoFs in *ANGPTL3* tended to have higher PRSs. As with the continuous traits, these results support the use of PRS when prioritizing individuals for rare disorder screening. These results also provide strong evidence of a liability threshold model where disease status is determined by a balance of pathogenic and protective effects from common and rare variants (Figure 1).

Moving from validation to discovery, we tested exome wide for genes associated with protective or pathogenic misaligned continuous phenotype status and identified several genes with plausible biological pathways for their role in causing high or low misaligned phenotypes, including genes associated with lower-than-expected BMI (*ACSL6*, *FAT1*, and *NPL*) and higher-than-expected LDL-C (*TSC2*) and AAM (*KANK1*). This demonstrated that misalignment classification of continuous traits could improve power in gene discovery, especially for noisy self-reported phenotypes, such as AAM.^92^ We also performed an exome-wide scan for genes associated with misaligned disease status (Note S2), but this was less fruitful, yielding a single association in *HLA-DRB5* (MIM: 604776) for CAD-protective pLoF burden. This could be because there are not many genes with rare variant burden effects large enough to counteract common variant effects and cause misaligned disease status.

There were some limitations in this study. First, testing for enrichment of rare damaging variants among misaligned individuals has inherently limited statistical power due to sparse variant presence among misaligned individuals. For example, when testing continuous traits, there were zero misaligned individuals harboring rare deleterious variants for more than half (32/58) of the canonical gene enrichment tests and 78.7% (389/494) of GEL PanelApp gene enrichment tests across all pLoF and damaging missense enrichment tests. This made it impossible to assess relative enrichment of damaging variants in misaligned individuals versus aligned individuals in these instances. In addition to the double jeopardy in statistical power when testing for enrichment of rare variants in a rare group of individuals, there are also expected to be fewer pathogenic variants in the UKB because, on average, UKB participants are healthier than the general population.^93^ Second, we used variant consequence categories based on computational prediction (methods) rather than strictly clinical evidence. Using computational prediction may lead to some miscategorized variants but enabled us to scale the enrichment tests to many more variants than would be feasible through manual curation of single variants informed by clinical case studies. Third, throughout this paper, we use PGSs/PRSs generated by a single study,^25^ provided by the UKB. We chose these scores because they were rigorously defined, readily available, and generated independent from our work. Evaluating the performance of our misalignment testing approach across PGSs generated by other methods could further generalize our findings and determine what aspects of a PGS make it particularly suited to misalignment testing. Finally, this study was performed solely in individuals classified as having European genetic ancestry (methods). Identifying misaligned individuals in non-European populations is difficult, as these populations are generally less represented in genome-wide association studies (GWASs) with sufficient sample size to generate informative PGSs. However, as increasingly diverse large biobanks are created, such as the *All of Us* (AoU) dataset,^94^ misalignment classification will become feasible for more ancestry groups.

There are several areas of opportunity to build on this work. Non-genetic factors, such as SES, medication, or lifestyle choices, could also explain phenotype misalignment. We show evidence that such factors are significantly associated with phenotype misalignment (Note S1). Correcting for these non-genetic factors could also improve power for detecting rare genetic effects on misalignment. For instance, adjusting LDL-C for cholesterol-lowering medication appeared to improve power for canonical gene burden associations (Note S3). Expanding this level of bespoke adjustment for non-genetic factors to all traits could boost power in future work. Replication of results in an independent cohort, such as the AoU dataset,^94^ would further increase confidence in results. Some traits had a much lower sample size (BMD, IOP, and AAM; *n* < 204, 000) than others (height, LDL-C, BMI, and HbA1c; *n* > 320, 000), which made it difficult to interpret how effective this analysis was in finding associations. Replicating the results in a larger dataset, with greater coverage for those low-sample-size traits, could enable a better comparison of statistical power. Hypothesizing that singletons observed in the UKB could be harboring large-effect variants such as *de novo* variants, misaligned individuals could be tested for enrichment of singleton burden. We performed this analysis and found moderate evidence pointing to deleterious singleton burden associating with shorter-than-expected stature (Note S4). Singleton burden analysis could be refined by calling *de novo* variants and testing their burden directly; however, this was beyond the scope of this project. Unlike the gene-level tests used throughout our work, performing a variant-level exome-wide scan for association could highlight the specific variants driving gene-level associations. However, this higher-resolution approach may only be practical for rare but not ultra-rare variants, as statistical power to detect very rare variant associations (e.g., MAC ≤ 10) is attenuated compared to associations in more common variants with the same effect size.^95^ Last, when performing exome-wide scans for protective genetic effects associated with PRS in disease controls, we only tested pLoF burden. This was because we expected pLoFs to have a much stronger effect than other missense variants, with a strong effect presumably necessary to cause disease status misalignment. However, as observed when testing gene burden associated with misaligned status in continuous traits, there may be value in expanding to damaging missense variants.

We demonstrated that, for a range of heritable continuous traits and complex diseases, misaligned individuals are enriched for rare damaging variants. This work highlights the value of considering both common and rare genetic components of trait variability, as it may prioritize certain individuals for rare-disease diagnosis and could be leveraged to find pathogenic or protective associations.

## Data and code availability

Full summary statistics for the exome-wide scan and empirical null analyses are available at https://doi.org/10.5281/zenodo.20110184. Code used for this analysis is available at https://github.com/lindgrengroup/ukb_misaligned. Our pipeline for REGENIE-based association testing is available at https://github.com/lindgrengroup/ukb_wes_450k_gwas.

## Acknowledgments

N.A.B. is supported by the Pembroke College Oxford-Bendich Graduate Scholarship, the Clarendon Fund, and Wellcome Trust grant 224890/Z/21/Z. S.S.V. is supported by the Rhodes Scholarships, the Clarendon Fund, and the Medical Sciences Doctoral Training Centre at the University of Oxford. F.H.L. is supported by the Wellcome Trust (award 224894/Z/21/Z) and the Medical Sciences Doctoral Training Centre at the University of Oxford. C.M.L. is supported by the Li Ka Shing Foundation; the NIHR Oxford Biomedical Research Centre, Oxford; the NIH (1P50HD104224-01); and the Gates Foundation (INV-024200). This research was supported by Wellcome Trust core award grant 203141/Z/16/Z and a Wellcome Trust residual award (221782/Z/20/Z) with additional support from the NIHR Oxford BRC. The views expressed are those of the authors and not necessarily those of the NHS, the NIHR, or the Department of Health. H.C. is supported by the Wellcome Trust (318918/Z/24/Z) and the NIH (1P50HD104224-01). D.S.P. is supported by the Pioneer Center for Statistical and Computational Methods for Advanced Research to Transform Biomedicine (SMARTbiomed), DNRF grant P4. This research was conducted using the UK Biobank resource under application number 11867. We thank UK Biobank participants for their contributions.

## Author contributions

Conceptualization, C.M.L. and N.A.B.; funding acquisition, C.M.L.; data curation, N.A.B., F.H.L., and B.H.; methodology, N.A.B., D.S.P., S.S.V., and H.C.; software, N.A.B., F.H.L., and B.H.; formal analysis, N.A.B. and F.H.L.; writing – original draft, N.A.B.; writing – review & editing, N.A.B. and D.S.P.; visualization, N.A.B.; project administration, N.A.B., C.M.L., and D.S.P.; supervision, C.M.L. and D.S.P.

## Declaration of interests

S.S.V. is currently employed by Illumina but while she conducted the research described in this manuscript was only affiliated with the University of Oxford. C.M.L. is currently a part-time employee of the Ellison Institute of Technology and Population Health Partners (PHP), is chief science officer of Aneira Health, owns equity in PHP and its subsidiaries, and has a partner who works at Vertex.

## Supplemental information

Document S1. Figures S1–S17, Notes S1–S4, and Tables S1, S4, and S11–S13

Data S1. Tables S2, S3, and S5–S10

Document S2. Article plus supplemental information
