# Supplementary material for: Individuals who deviate from polygenic expectation are enriched for damaging variants in genes linked to rare disease
Source: Am J Hum Genet. 2026 Jun 22;113(7):1415–33. doi: 10.1016/j.ajhg.2026.05.013 (PMC13504349; doi:10.1016/j.ajhg.2026.05.013)
Supplement: Supplementary file 1 [file mmc1.pdf]

The American Journal of Human Genetics, Volume *113*

Supplemental information

Individuals who deviate from polygenic expectation

are enriched for damaging variants

in genes linked to rare disease

Nikolas A. Baya, Frederik H. Lassen, Barney Hill, Samvida S. Venkatesh, Hannah Currant, Cecilia M. Lindgren, and Duncan S. Palmer

Supplemental notes

Note S1: Association of non-genetic factors with continuous phenotype and disease status misalignment relative to genetic expectation

Phenotype misalignment relative to common-variant polygenic score may partly be explained by "non-genetic" factors. We tested association of misaligned status in continuous and dichotomous traits with SES, medication, smoking status, and exercise (Figure S7; Table S4).

Socioeconomic status

Using Townsend Deprivation Index (TDI) (UKB data field: 22189) as a measure for SES, with high TDI corresponding to lower SES, we tested whether higher TDI was associated with a pathogenic misaligned effect. For height, we assumed a "pathogenic" misaligned effect would manifest in shorter stature, per previous evidence[1]. For other continuous traits we tested the misaligned group associated with pathogenic direction of effect, as described in our exome-wide scan for pathogenic burden.

Using logistic regression to test the effect of TDI on pathogenic misaligned status in continuous traits, we observed that misaligned individuals tended to have higher TDI than aligned individuals in every trait ($\beta < 0$, one-sided Wald test $P < 0.05$; Figure S7; Table S4). Among the strongest associations were those in higher than genetically expected BMI ($P = 2.63 \times 10^{-10}$) and HbA1c ($P = 1.07 \times 10^{-8}$), and shorter than genetically expected stature ($P = 1.16 \times 10^{-13}$).

In dichotomous traits, we expected that higher deprivation would have a pathogenic misaligned effect. We used linear regression to test whether higher TDI was associated with decreased PRS in cases and observed significant negative associations ($\beta < 0$, one-sided t-test $P < 0.05$) for T2D and OP (Figure S7; Table S4).

Medication

We investigated the relationship between self-reported medication and phenotype misalignment for three dichotomous traits (T2D, CAD, OP) and their corresponding continuous liability phenotypes (HbA1c, LDL-C, BMD). We mapped self-reported medication (UKB data field: 20003) to Anatomical Therapeutic Chemical (ATC) Index 2026 (`https://atcddd.fhi.no/atc_ddd_index/`) categories using Supplementary Data 1 from Wu *et al.*[2] to define individuals who self-report taking the following ATC categories of medications:

Code	Description	Disease	Liability Trait
A10B	BLOOD GLUCOSE LOWERING DRUGS, EXCL. INSULINS	T2D	HbA1c
C10	LIPID MODIFYING AGENTS	CAD	LDL-C
M05B	DRUGS AFFECTING BONE STRUCTURE AND MINERALIZATION	OP	BMD

We hypothesized that medication would be associated with a protective misaligned effect. Therefore, we tested whether disease controls who reported medication for the disease being tested tended to have higher PRS than individuals not reporting medication. For the liability traits, we tested whether individuals with lower than genetically expected HbA1c or LDL-C, or higher than genetically expected BMD were more likely to report medication relevant to the trait being tested.

We observed that disease controls who reported medication for the tested disease had significantly higher PRS ($\beta > 0$, one-sided t-test $P < 2 \times 10^{-61}$) than individuals not reporting the relevant medication (Figure S7; Table S4), consistent with a protective misaligned effect.

Among the three liability traits, the only trait to show a significant enrichment (Fisher's exact test $P < 0.05$) was LDL-C (OR [90% CI] = 11.7 [8.98-15.2], $P = 1.21 \times 10^{-53}$) (Figure S7; Table S4).

Smoking

We tested whether smoking behavior, specifically if an individual had ever smoked (UKB data field: 20160), was associated with a pathogenic effect on phenotype misalignment.

Using Fisher's exact test, we tested each continuous trait (excluding height, for lack of an expected pathogenic direction of effect), and observed that individuals with lower than genetically expected BMD and higher than genetically expected BMI, HbA1c, and LDL-C were more likely to report having ever smoked (OR > 1, one-sided Fisher's exact test $P < 0.05$; Figure S7).

In dichotomous traits, we used linear regression to test whether smoking status was associated with lower PRS in cases, which implies a pathogenic misaligned effect. We observed a significant association in T2D cases (β [90% CI]=-0.0391 [-0.0574, -0.0209], one-sided t-test $P = 2.12 \times 10^{-4}$).

Exercise

We used summed Metabolic Equivalent of Task (MET) minutes per week for all activity (UKB data field: 22040) as an indicator for amount of weekly exercise, hypothesizing that higher amount of exercise would be associated with a protective misaligned effect.

Among continuous traits (with height excluded, for lack of hypothesized "protective" misaligned effect), we observed a significant association (one-sided Wald test $P < 0.05$) in BMI, with low-misaligned individuals having higher MET scores (β [90% CI]=5.21×10^{-5} [4.41×10^{-6}, 9.97×10^{-5}], $P = 0.0361$; Figure S7).

Among disease traits, we found a positive association of MET scores with PRS in T2D controls (β [90% CI] =3.69×10^{-6} [2.46×10^{-6}, 4.92×10^{-6}], one-sided t-test $P = 3.81 \times 10^{-7}$; Figure S7), consistent with a protective misaligned effect of exercise.

Note S2: Exome-wide scan for genes associated with misaligned disease status

Given the significant associations of pathogenic and protective pLoF burden with PRS among disease cases and controls, respectively, we hypothesized that we could discover genes with pathogenic or protective variants by testing exome-wide for PRS association with gene burden.

We performed an exome-wide scan for gene burden associated with protective and pathogenic misaligned disease status, testing burden of rare (MAF $< 0.1\%$) pLoF variants. To test for protective burden, we subset to disease controls for T2D, CAD, and OP, and used REGENIE to test for burden associated with increased PRS (Figure 1). When testing pathogenic burden, we subset to disease cases and tested for burden associated with decreased PRS. As a point of comparison, we also performed traditional burden tests on disease status. All tests were one-sided and P-values were adjusted for FDR$\leq 5\%$ using the BH procedure[3]. FDR correction was performed across results from all phenotypes, but separately for pathogenic and protective tests. There were no FDR-significant associations for pathogenic burden associated with decreased PRS in disease cases (Figure S15; Table S10), thus only the protective burden associations are presented here.

There were two genes where rare (MAF $< 0.1\%$) pLoF variant burden was significantly associated (FDR-adjusted one-sided burden $P_{\text{adj}} \leq 0.05$) with increased PRS in controls for CAD (*HLA-DRB5*) and OP (*NBPF3* [MIM 612992]) (Figure S14, Table S10). After conditioning on common imputed variants within 1 Mb of the gene, the burden association in *HLA-DRB5* remained nominally significant (one-sided burden $P = 3.89 \times 10^{-4}$).

The gene *HLA-DRB5* (major histocompatibility complex, class II, DR beta 5) codes for the beta chain in a human leukocyte antigen (HLA) class II cell surface complex, involved in generating immune responses[4]. Although CAD is linked with immune response in the context of inflammation from atherogenesis[5], there is no evidence in the literature of a plausible connection between *HLA-DRB5* LoF and reduced risk of CAD onset.

Note S3: Adjusting LDL-C for cholesterol-lowering medication

To evaluate the effects of adjusting for a non-genetic factor on enrichment results, we performed a pilot study on LDL-C adjusted for cholesterol-lowering medications for canonical genes associated with monogenic LDL-C disorders. We calculated adjusted LDL-C following the approach of Patel *et al.*[6], which involves applying literature-informed medication-specific adjustments (e.g. dividing observed LDL-C by 0.70 if individuals reported taking statins, or dividing by 0.90 if taking fibrates; see Patel *et al.* Supplemental Table I). We mapped self-reported medication (UKB data field: 20003) to ATC Index 2026 categories using Supplementary Data 1 from Wu *et al.*[2], using the following ATC codes for each cholesterol-lowering medication:

If an individual reported taking medication for cholesterol (UKB data fields: 6177 [males], 6153 [females]) but did not specify the medication in data field 20003, they were assumed to be taking statins, per Patel *et al.*

Code	Medication
C10AA	HMG CoA reductase inhibitors (Statin)
C10AB	Fibrates
C10AC	Bile acid sequestrants
C10AD	Nicotinic acid and derivatives (Niacin)
C10AX09	Ezetimibe

Testing enrichment of rare deleterious burden in canonical genes associated with monogenic LDL-C disorders, we observed that in all but one gene-consequence pair medication-adjusted LDL-C had higher odds ratio point estimates and lower Fisher's exact test P-values than unadjusted LDL-C (Figure S13; Table S11). The only exception was a marginally higher P-value for the test of pLoF burden in *PCSK9* ($P_{\text{unadjusted}} = 2.97 \times 10^{-11}$, $P_{\text{medication\_adjusted}} = 5.02 \times 10^{-11}$). There was also a nominally significant ($P < 0.05$) result for damaging missense variants in *PCSK9* (OR [90% CI] = 17.0 [5.25, 55.3], one-sided Fisher's exact test $P = 6.59 \times 10^{-3}$), which had been non-significant when using unadjusted LDL-C (OR = 7.33 [1.40, 38.3] $P = 0.129$).

Note S4: Individuals with genetically unexpected phenotypes are nominally enriched for consequence-stratified singleton burden in intolerant genes

Methods for singleton burden association

Singleton variants were defined as variants with an alternate allele count of 1 and alternate allele frequency $< 0.01\%$. We used PLINK[7] to aggregate singleton counts for each chromosome and each variant consequence category. We aggregated singleton burden across all genes predicted to be intolerant to LoF variants. We defined intolerant genes using the gene intolerance threshold (LOEUF< 0.6) proposed by Karczewski *et al.*[8], using gnomAD v4.1 constraint metrics downloaded from the gnomAD website. Of the 18,623 canonical transcripts available, 3,900 were defined as intolerant. Subsetting to intolerant genes is expected to increase signal-to-noise ratio when testing variant effects, because variants in tolerant genes are not expected to affect the function of the genes, and by extension, not expected to affect the trait. This filtering is particularly important for exome-wide aggregated burden because there are many more variants being aggregated together than in the previous single- or grouped-gene tests.

We tested for the effect of consequence-specific singleton burden (s_c, for consequence category c) using regression, controlling for overall singleton burden by including as a covariate the total singleton burden (s_{total}) across consequence categories, per Genovese *et al.*[9]. We used logistic regression when testing misaligned status in continuous traits, and linear regression when testing for effect of singleton burden on covariate-residualised PRS (PRS$'$) in cases and controls for dichotomous case-control traits:

Continuous traits		Misaligned status $\sim s_c + s_{\text{total}} + 1$
Dichotomous traits	Cases	$\text{PRS}'_{\text{cases}} \sim s_c + s_{\text{total}} + 1$
	Controls	$\text{PRS}'_{\text{controls}} \sim s_c + s_{\text{total}} + 1$

We calculated one-sided P-values for the singleton burden term, given our hypothesis that ultra-rare variants are more likely to cause individuals to be misaligned rather than aligned. We stratified the analysis across four protein-coding variant consequence categories: pLoF, damaging missense, other missense and synonymous (see Methods for variant consequence definitions).

Monotonic point estimates of singleton enrichment in height suggests deleterious singleton burden is associated with shorter stature

Among continuous traits, there were no significant (FDR-adjusted one-sided t-test $P_{\text{adj}} \leq 0.05$) enrichment results (Figure S16; Table S12) after FDR correction. Approaching the results qualitatively, one notable observation was the monotonic trend across functional categories in enrichment effect sizes for height (Figure S16), a trait that showed strong enrichment when testing deleterious burden in genes associated with monogenic and rare disorders. Individuals with lower-than-expected height were increasingly enriched for more deleterious singleton burden, while individuals with higher-than-expected height were increasingly depleted for more deleterious singleton burden. This suggests that, in general, deleterious singletons are more likely to decrease rather than increase height. This is consistent with the work of Backman *et al.*[10], who found that, among genes where pLoF or likely deleterious missense singleton burden was significantly associated with standing height, the burden effects in most (8/10) genes decreased height (see Backman *et al.*[10] Supplementary Data 2). This also aligns with a recent analysis of UKB whole-genome sequencing data[11], in which four genes where pLoF singletons were significantly associated with height, three genes were associated with decreased height (see Hawkes *et al.*[11] Supplementary Data 6).

Limited evidence for association of singleton burden with misaligned disease status

We performed one-sided t-tests for association of singleton burden with PRS for CAD, OP, and T2D. In cases we tested whether higher singleton burden was associated with lower PRS, and in controls we tested whether higher singleton burden was associated with higher PRS. This was based on the same hypotheses used for the canonical and GEL tests: Association of variant burden with lower or higher PRS could indicate effects which are pathogenic or protective, respectively, against common-variant risk scores.

After FDR correction, there were no significant associations (FDR-adjusted one-sided t-test $P_{\text{adj}} \leq 0.05$) (Figure S17; Table S13). In CAD cases, effect size point estimates monotonically decreased with consequence deleteriousness, with pLoF and damaging missense (the two most deleterious consequences) singleton burden being nominally associated (one-sided t-test $P < 0.05$) with lower PRS. This suggests that deleterious singleton burden in intolerant genes could be pathogenic for CAD. We also observed similar monotonicity in effect size point estimates for T2D cases, suggestive of deleterious burden in intolerant genes being pathogenic for T2D, however, none of the enrichment tests were significant.

Supplemental figures

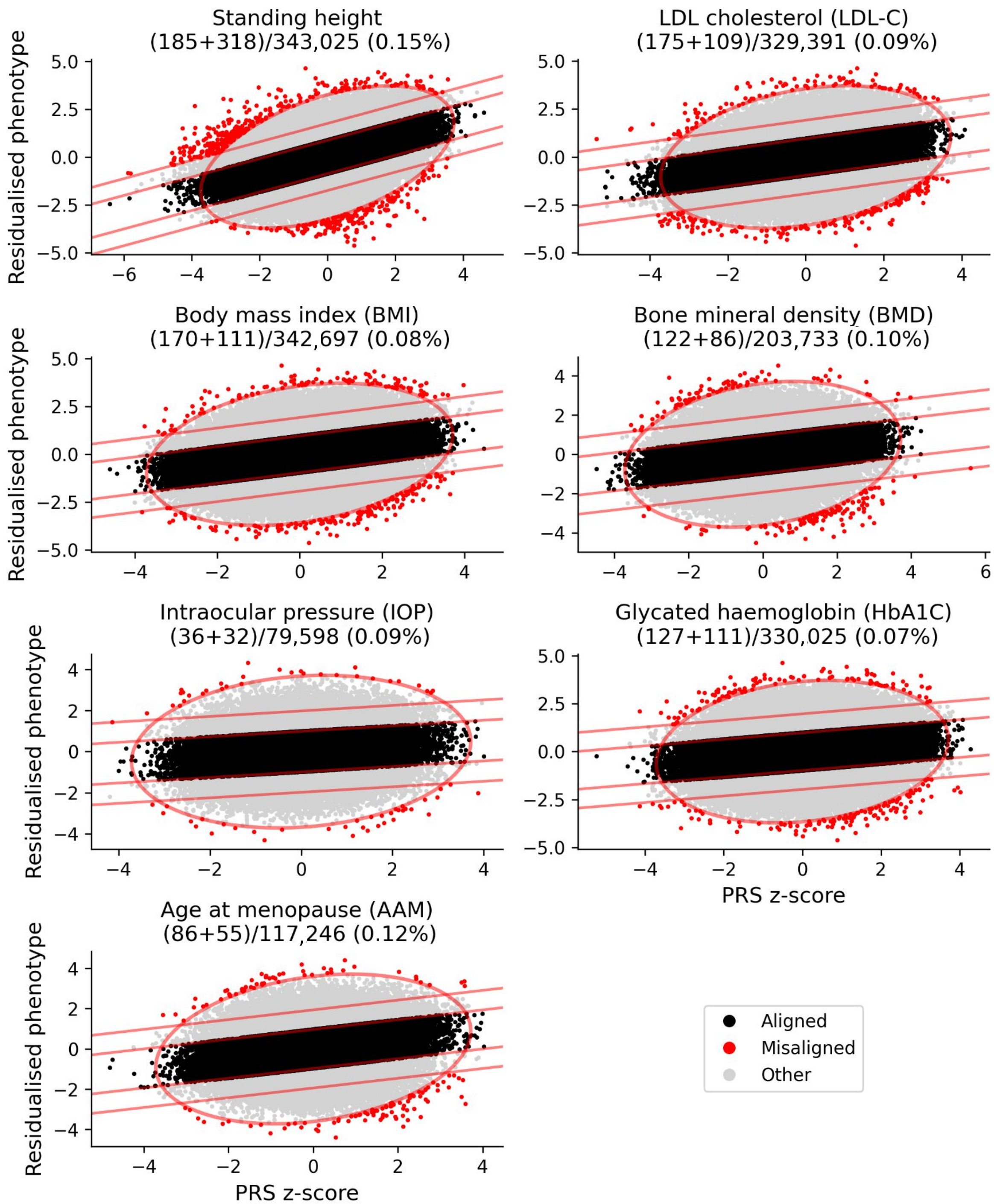

Figure S1: Classification of misaligned individuals for seven continuous traits. In the title of each plot is the name of the phenotype, the sum of low and high misaligned individuals divided by the total number of individuals with defined phenotype, and the total percentage of individuals who are classified as misaligned. The red oval delineates the Mahalanobis distance threshold, while the red lines indicate thresholds on the residuals to define aligned and misaligned individuals.

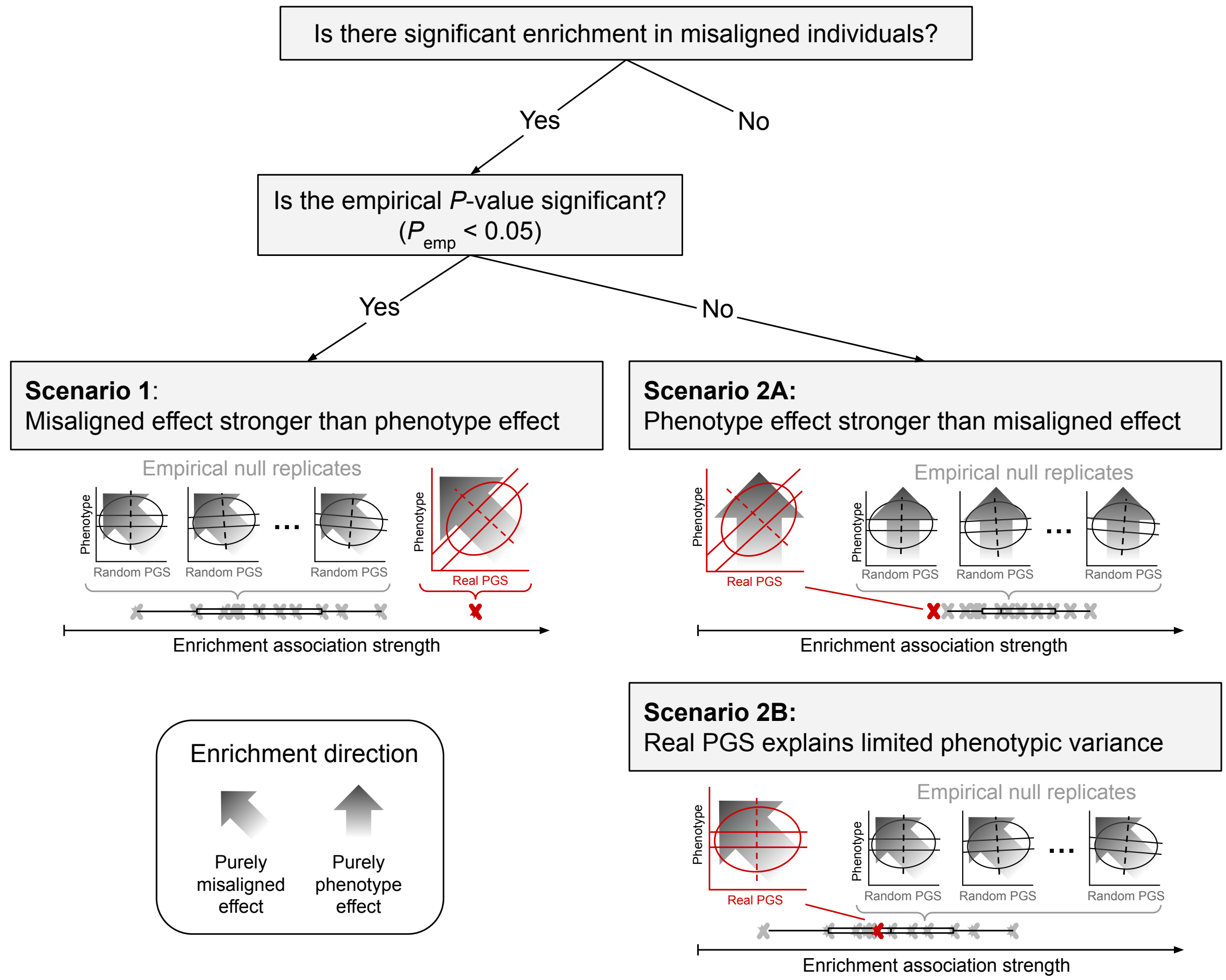

Figure S2: Possible scenarios for the empirical P-value. First, note that the empirical P-value is only calculated for genes which demonstrate significant enrichment of rare deleterious variants among misaligned individuals. The empirical P-value may either be significant or non-significant. To help visualize misaligned enrichment, the gradient of rare deleterious variant enrichment among individuals is shown here as an arrow with gradient shading. The "misaligned axis", orthogonal to the regression of observed phenotype and PGS, is shown as a dashed line. In this schematic, the alignment of the dashed misaligned axis with the enrichment gradient arrows suggests a strong enrichment. The wider the angle between these two, the weaker the enrichment. **Scenario 1** provides an example of a significant empirical P-value, where the observed test statistic (red "×"), based on misaligned enrichment testing using real PGS, outperforms empirical null test statistics (grey ×'s, with box-and-whisker plot for their distribution) generated from misaligned enrichment testing using simulated random PGS. **Scenarios 2A/B** describe examples where spurious enrichment in misaligned individuals can occur, but can be controlled with empirical P-values. Note that in reality, the effects described by these scenarios may be more subtle. It is also possible for a mix of both scenarios to occur. **Scenario 2A** describes the possibility that enrichment of individuals is stronger along the observed phenotype axis, rather than along the misaligned axis (orthogonal to the regression of observed phenotype and PGS). Here, empirical null enrichment tests are expected to outperform the real test statistic because, with the random PGS, the enrichment gradient (arrow) is closely matched with the misaligned axis (dashed line). This results in a non-significant empirical null P-value. **Scenario 2B** describes the possibility of a real PGS explaining limited phenotypic variance, such that the real test statistic is indistinguishable from the empirical null distribution, resulting in a non-significant empirical P-value.

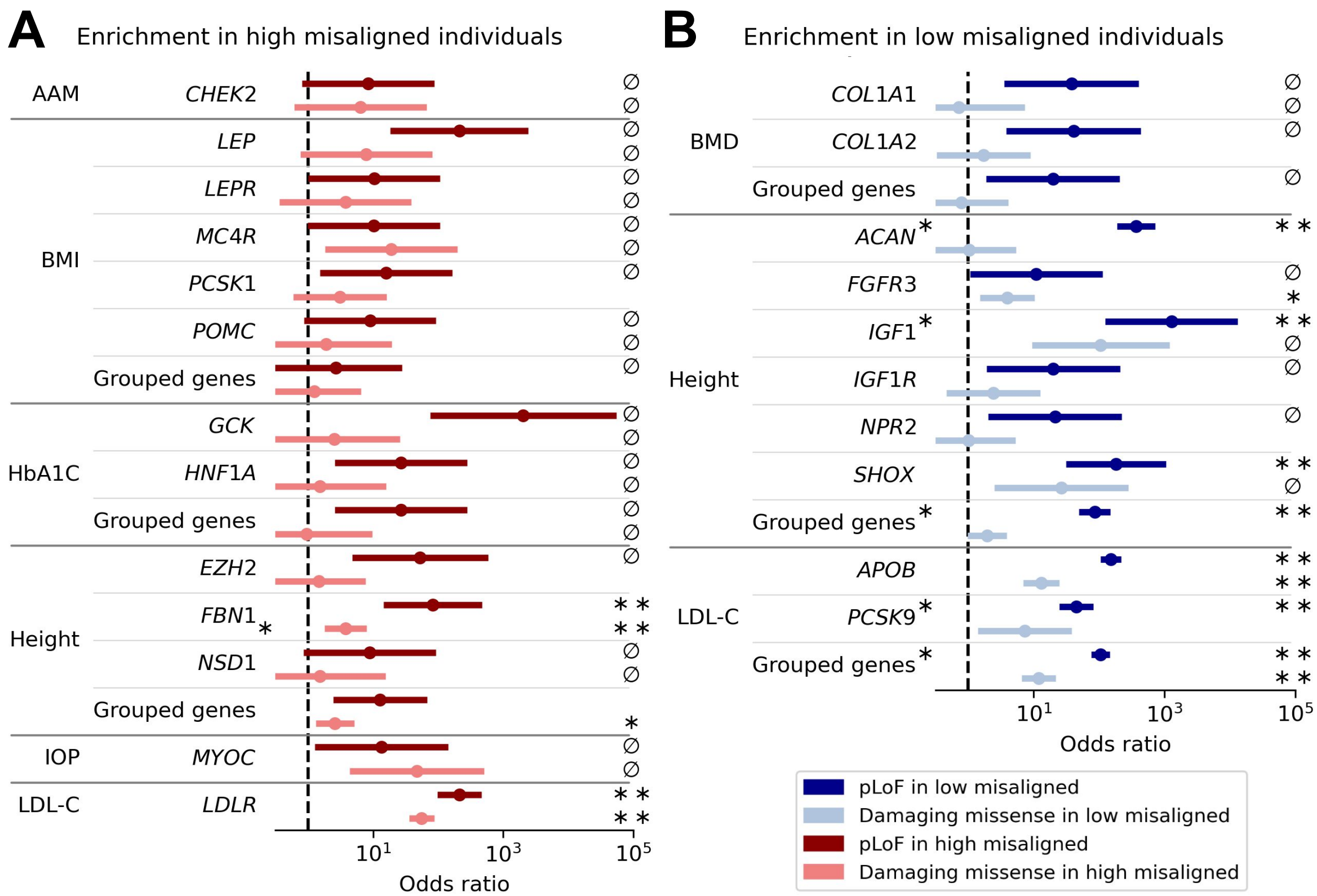

Figure S3: Enrichment of deleterious variants in canonical genes associated with monogenic disorders for continuous traits. (**A**) Enrichment testing in high misaligned individuals, in genes where deleterious variants are expected to cause higher-than-expected phenotypes. (**B**) Enrichment testing in low misaligned individuals, in genes where deleterious variants are expected to cause lower-than-expected phenotypes. Symbols to the right of each forest plot indicate whether the enrichment test was significant ($^{*}P < 0.05$, $^{**}P_{\text{adj}} < 0.05$) or whether misaligned individuals lacked variants ($\emptyset$) to be able to perform Fisher's exact test on a fully non-zero contingency table. Asterisks immediately to the right of the gene symbol indicate tests where the observed enrichment outperformed the empirical null ($P_{\text{emp}} < 0.05$). Odds ratios are calculated for all enrichment tests (with zeros in contingency tables filled with 0.5) and plotted with 90% confidence intervals, such that the lower bound corresponds to the lower bound of a one-sided 95% confidence interval.

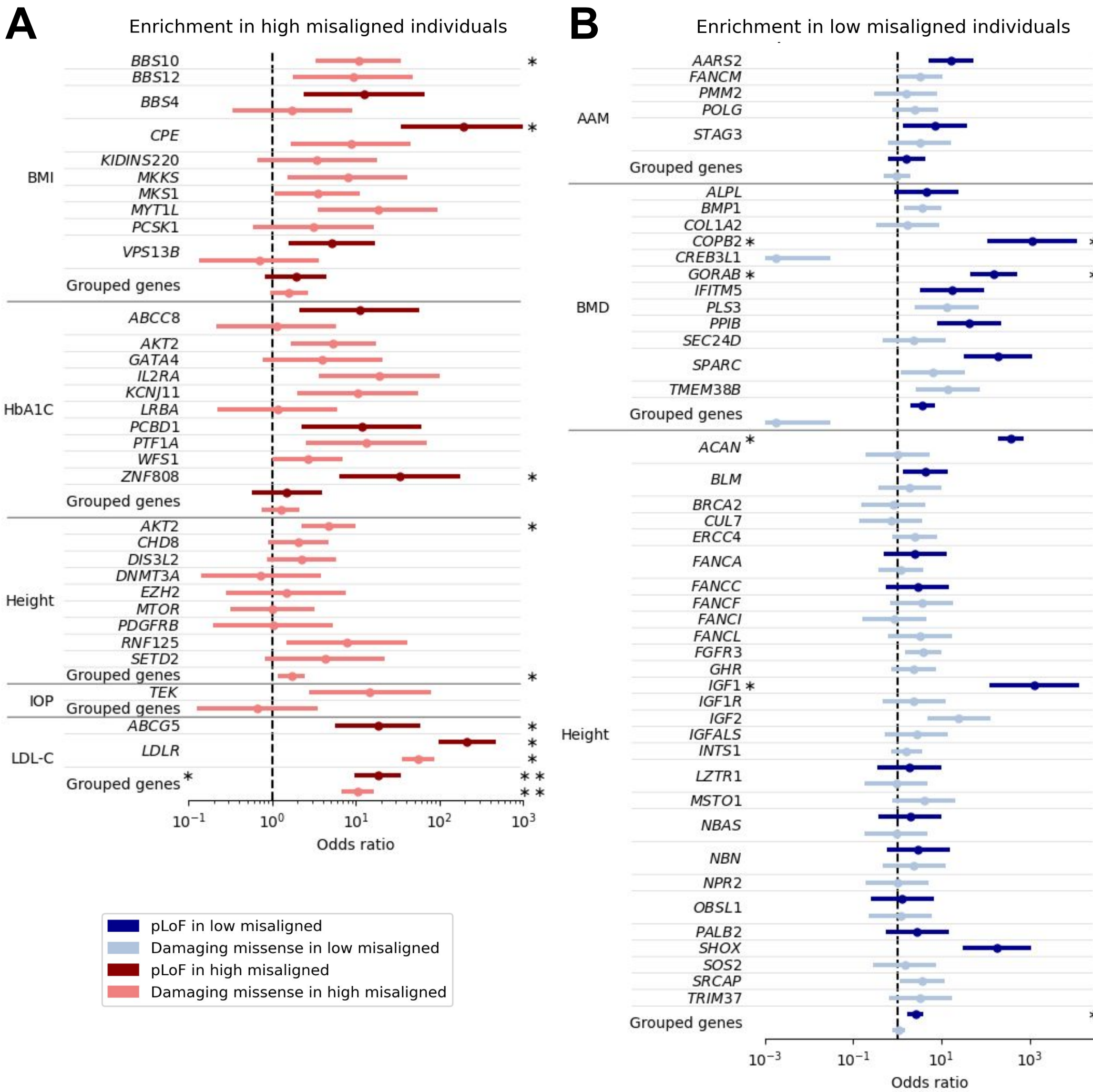

Figure S4: Enrichment of deleterious variants in GEL genes for continuous traits. (**A**) Enrichment testing in high misaligned individuals, in genes where deleterious variants are expected to cause higher-than-expected phenotypes. (**B**) Enrichment testing in low misaligned individuals, in genes where deleterious variants are expected to cause lower-than-expected phenotypes. Asterisks to the right of each forest plot indicate whether the enrichment test was significant ($^*P < 0.05$, $^{**}P_{\text{adj}} < 0.05$). Asterisks immediately to the right of the gene symbol indicate tests where the observed enrichment outperformed the empirical null ($P_{\text{emp}} < 0.05$). Odds ratios are calculated for all enrichment tests and plotted with 90% confidence intervals, such that the lower bound corresponds to the lower bound of a one-sided 95% confidence interval. Only enrichment results with fully non-zero contingency tables are shown.

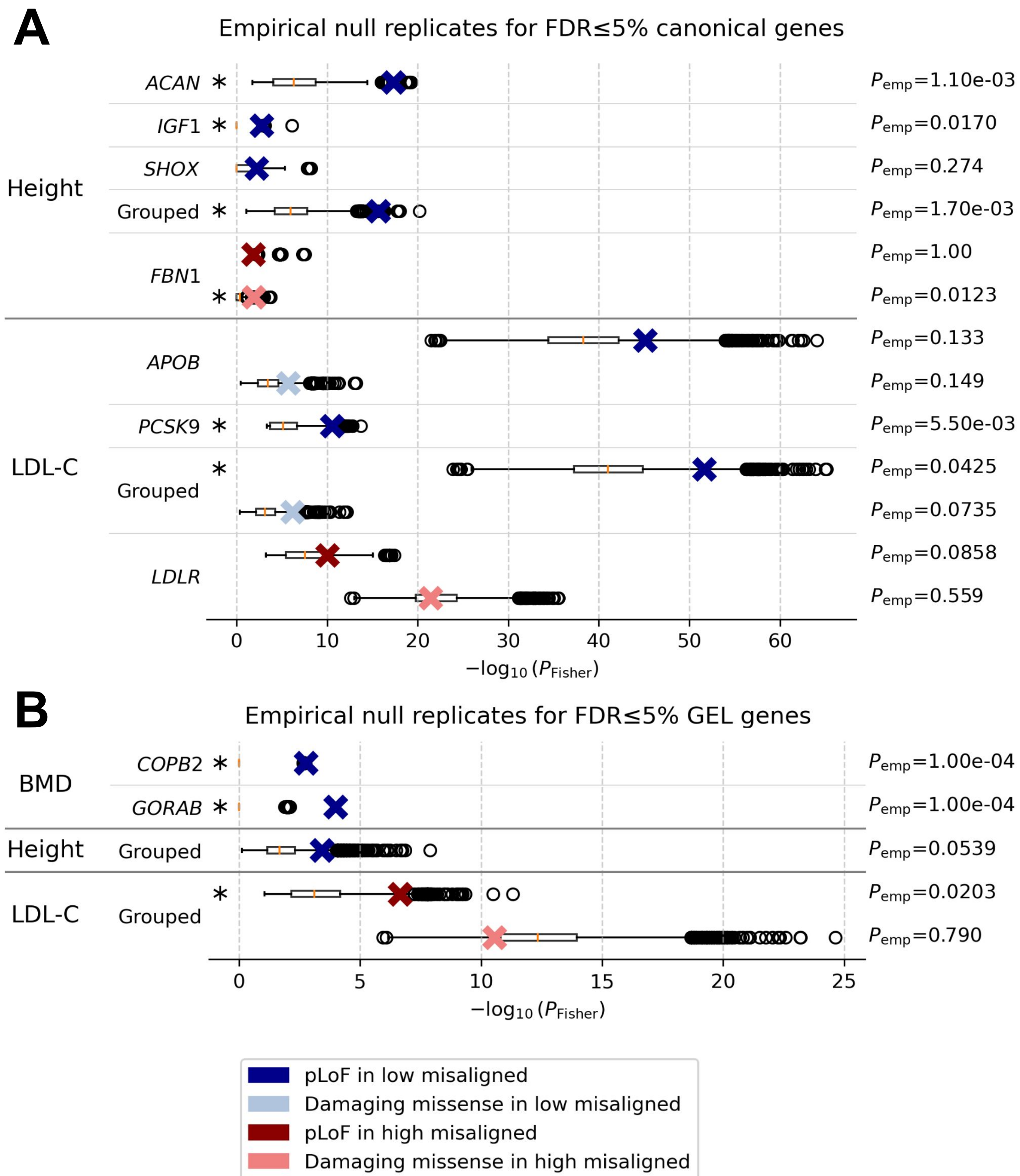

Figure S5: Empirical null replicates for canonical and GEL genes. (**A**) Canonical genes. (**B**) GEL genes. The empirical P-value (P_{emp}, labeled on the right of each plot) of a gene enrichment result indicates how the observed test statistic (one-sided Fisher's exact test P-value) compares against the empirical null distribution of test statistics (Methods). Asterisks immediately to the right of the gene symbol indicate tests where the observed enrichment outperformed the empirical null ($P_{\text{emp}} < 0.05$).

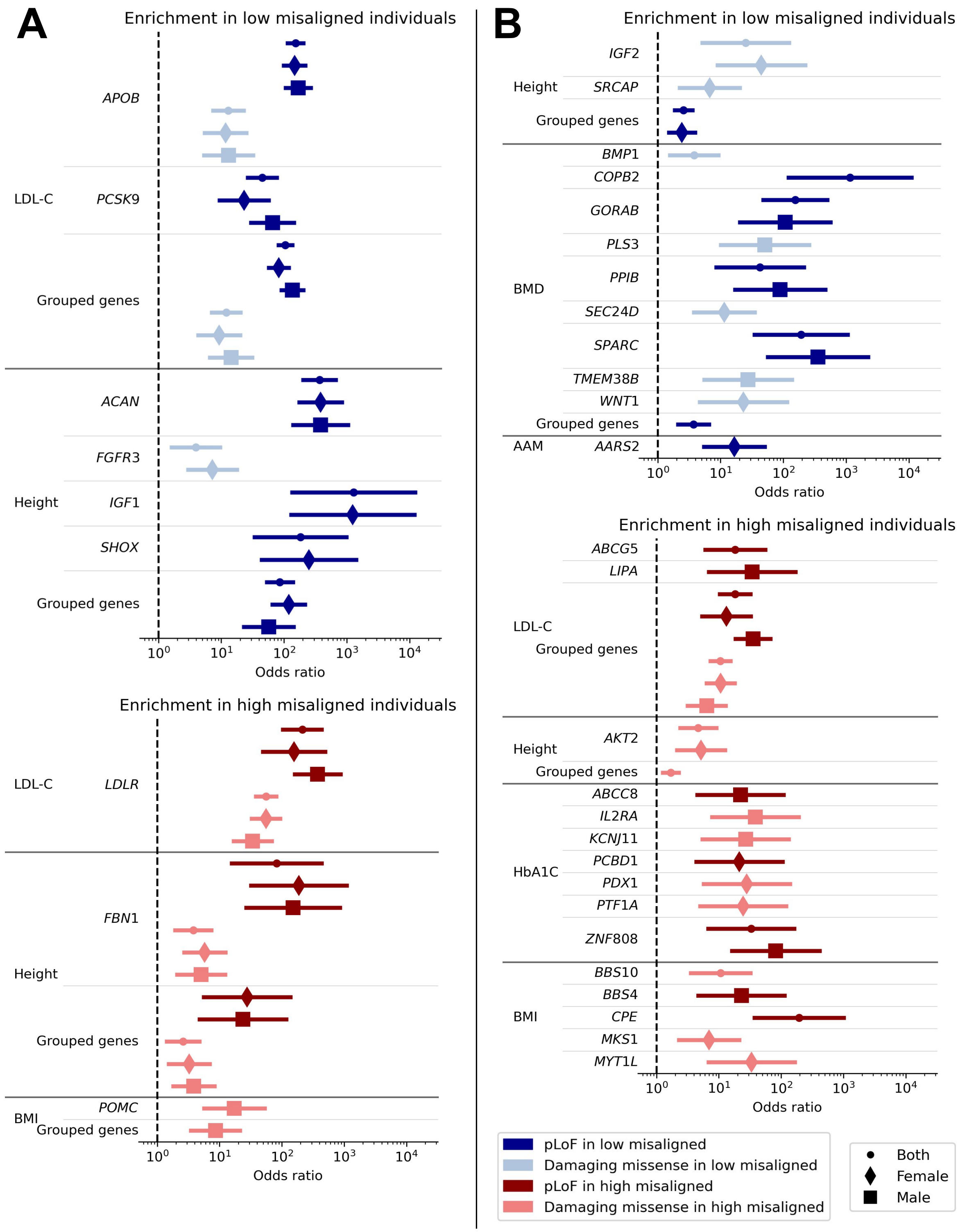

Figure S6: Sex-specific continuous trait enrichment results for canonical and GEL genes. (**A**) Canonical genes. (**B**) GEL genes. Only nominally-significant (one-sided Fisher's exact test < 0.05) results are shown.

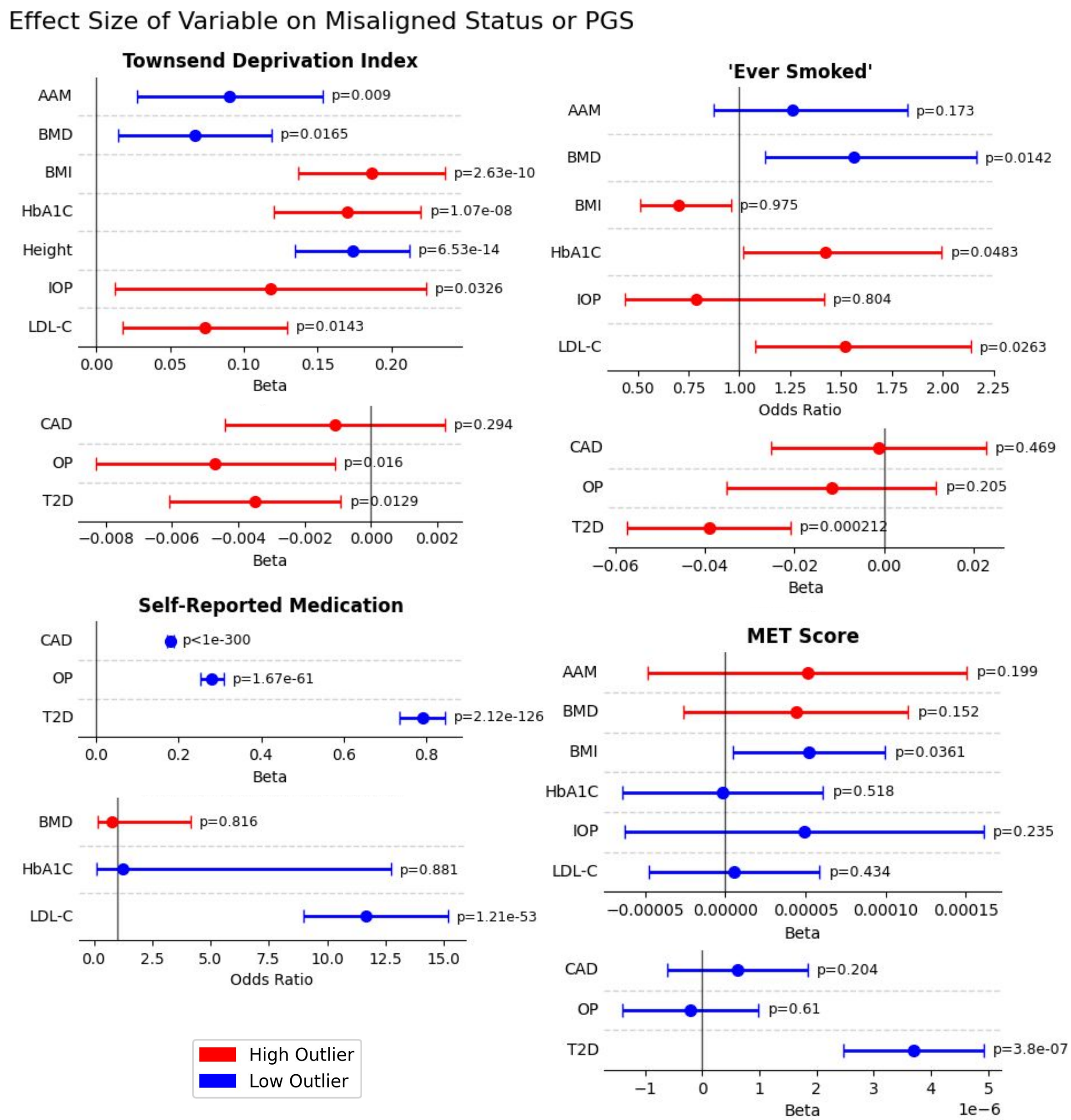

Figure S7: Effects of non-genetic variables on misaligned status. All P-values are one-sided and error bars represent 90% CIs. Fisher's exact test was used to test enrichment of overlap between binary variables (continuous trait misaligned status tested against medication status or 'Ever Smoked'), logistic regression was used to test association between binary dependent variables and continuous independent variables (continuous trait misaligned status tested against Townsend Deprivation Index and MET score), and linear regression was used to test association of continuous dependent variables and any independent variable (dichotomous trait case/control-specific PRS tested against any variable). Self-reported medication was specific to each disease or liability trait (e.g. lipid-lowering medication for CAD and LDL-C).

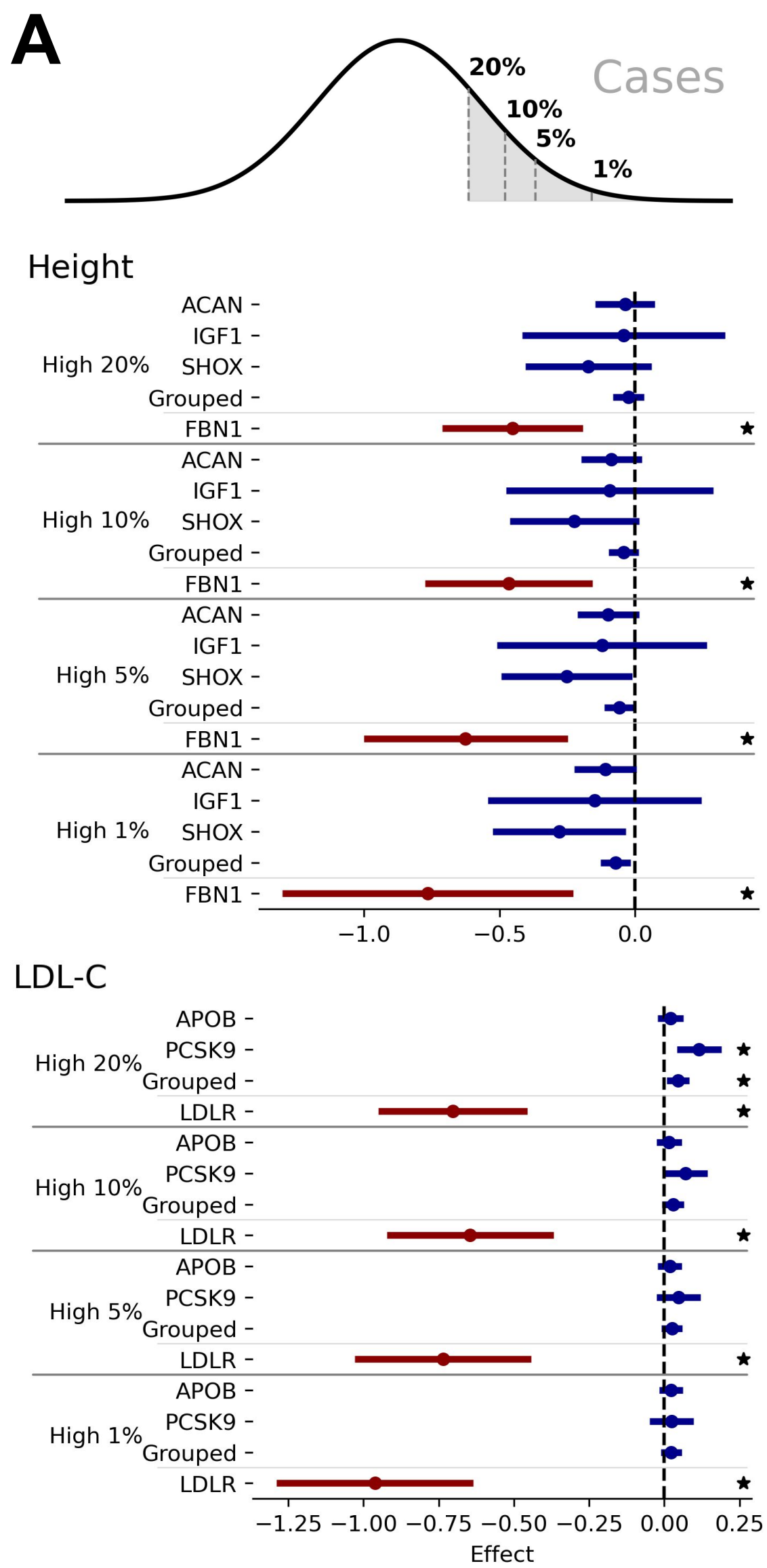

Figure S8: Enrichment testing in FDR-significant canonical genes associated with monogenic disorders for simulated dichotomous traits. (**A**) Dichotomous traits simulated with the upper tail as cases and everyone else as controls. (**B**) Dichotomous traits simulated with the lower tail as cases and everyone else as controls. Only genes and grouped genes which are significant (one-sided Fisher's exact test $P_{\text{adj}} < 0.05$) from misaligned enrichment testing of canonical genes in continuous traits are shown. Stars to the right indicate nominal significance (one-sided t-test $P < 0.05$) resulting from regression of PRS on variant presence in individuals.

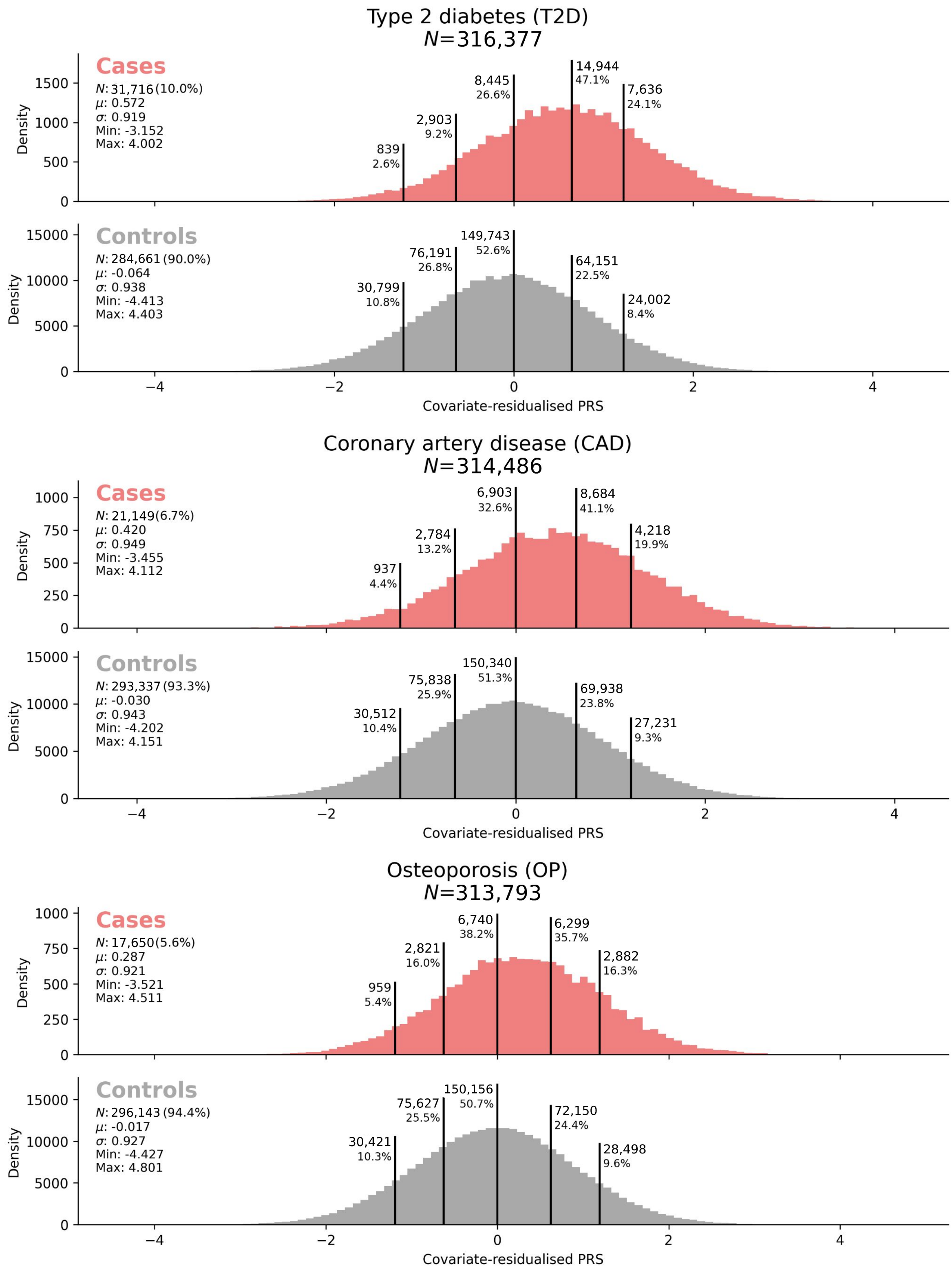

Figure S9: Distributions of covariate-residualised PRS stratified by case status. Vertical lines indicate 10th, 25th, 50th, 75th and 90th PRS percentiles, calculated on the combined distribution of PRS in both cases and controls. Numbers at the top of vertical lines, to the left or right, indicate the number and percentage of samples in the case/control group that lie to the left or right of the vertical line, respectively.

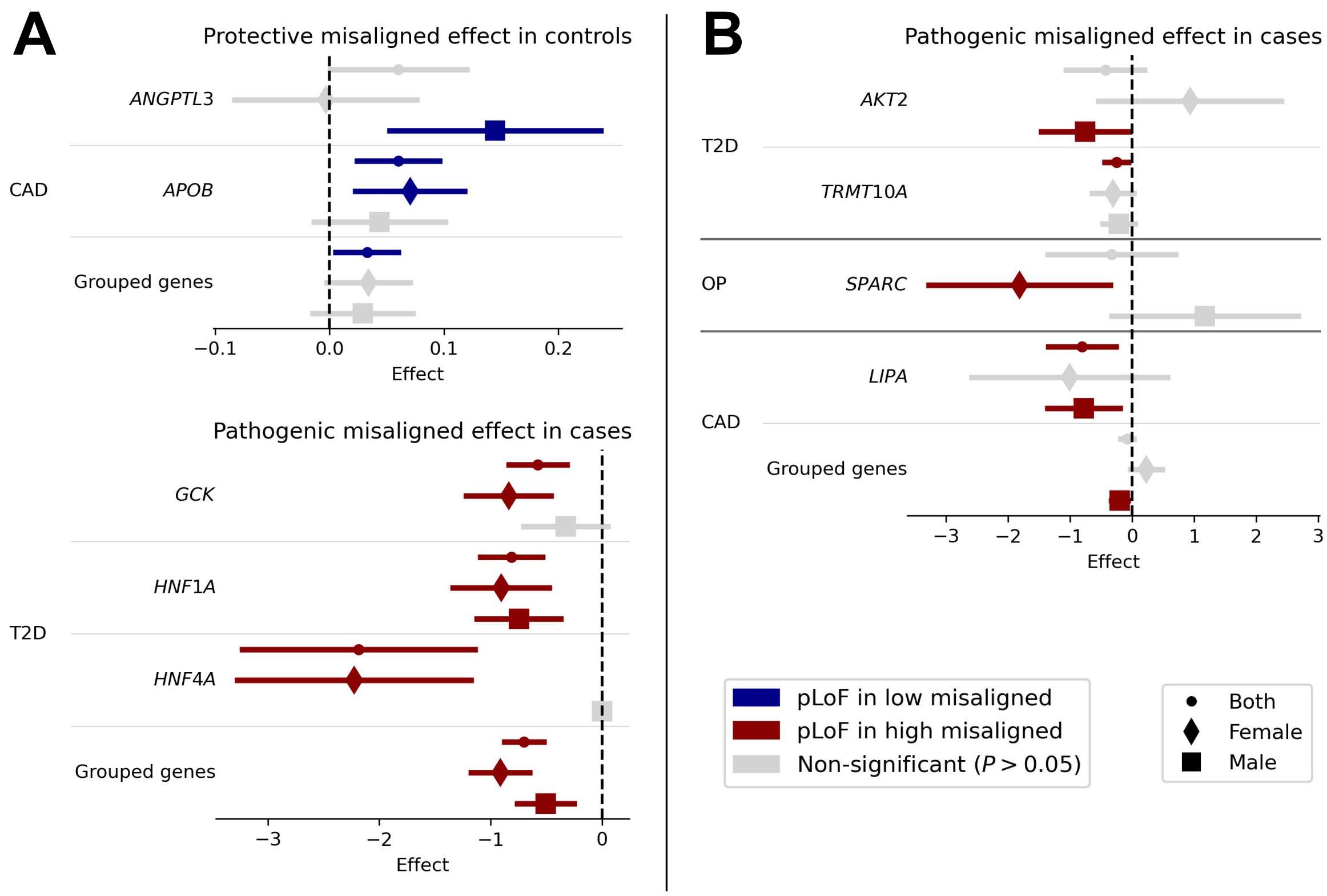

Figure S10: Sex-specific dichotomous trait results for canonical and GEL genes. (**A**) Canonical genes. (**B**) GEL genes. Only genes with at least one nominally-significant (one-sided Fisher's exact test < 0.05) result in the sex-combined cohort ("Both"), or sex-specific cohorts ("Female", "Male") are shown.

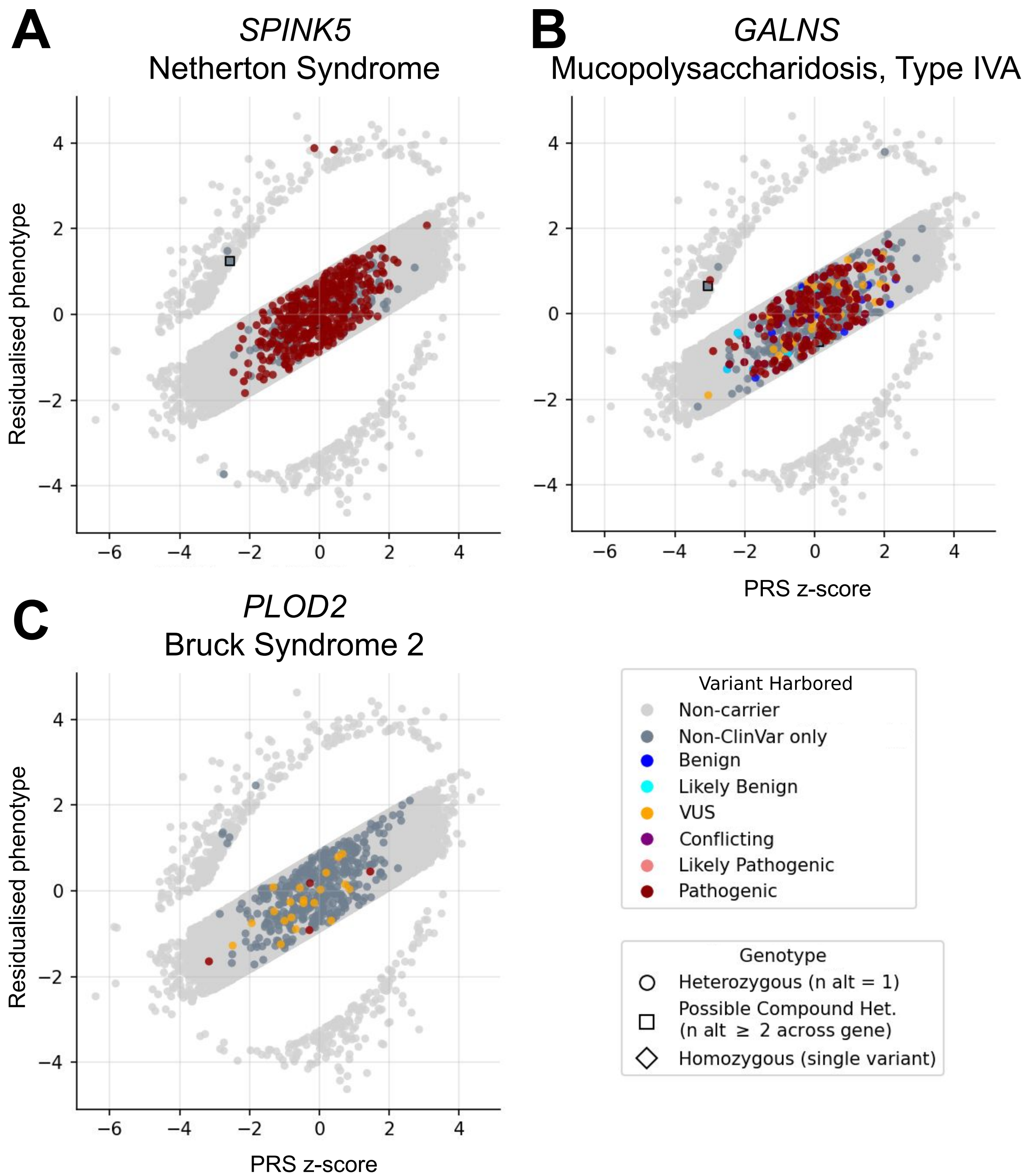

Figure S11: Enrichment of deleterious variants among high outliers for height in genes associated with short stature syndromes. (**A**) Enrichment of pLoF variants in *SPINK5*. Enrichment of damaging missense variants in (**B**) *GALNS* and (**C**) *PLOD2*. Only individuals harboring the relevant variant consequence category are highlighted (only pLoF or only damaging missense). "Possible Compound Het." individuals are defined as individuals with two or more alternate alleles of the relevant deleterious variant class (pLoF or damaging missense) across the whole gene, but not at the same variant. Homozygous individuals are those with two alternate alleles at the same variant. ClinVar[12] annotations for each gene-disorder pairing were obtained from the Open Targets platform[13].

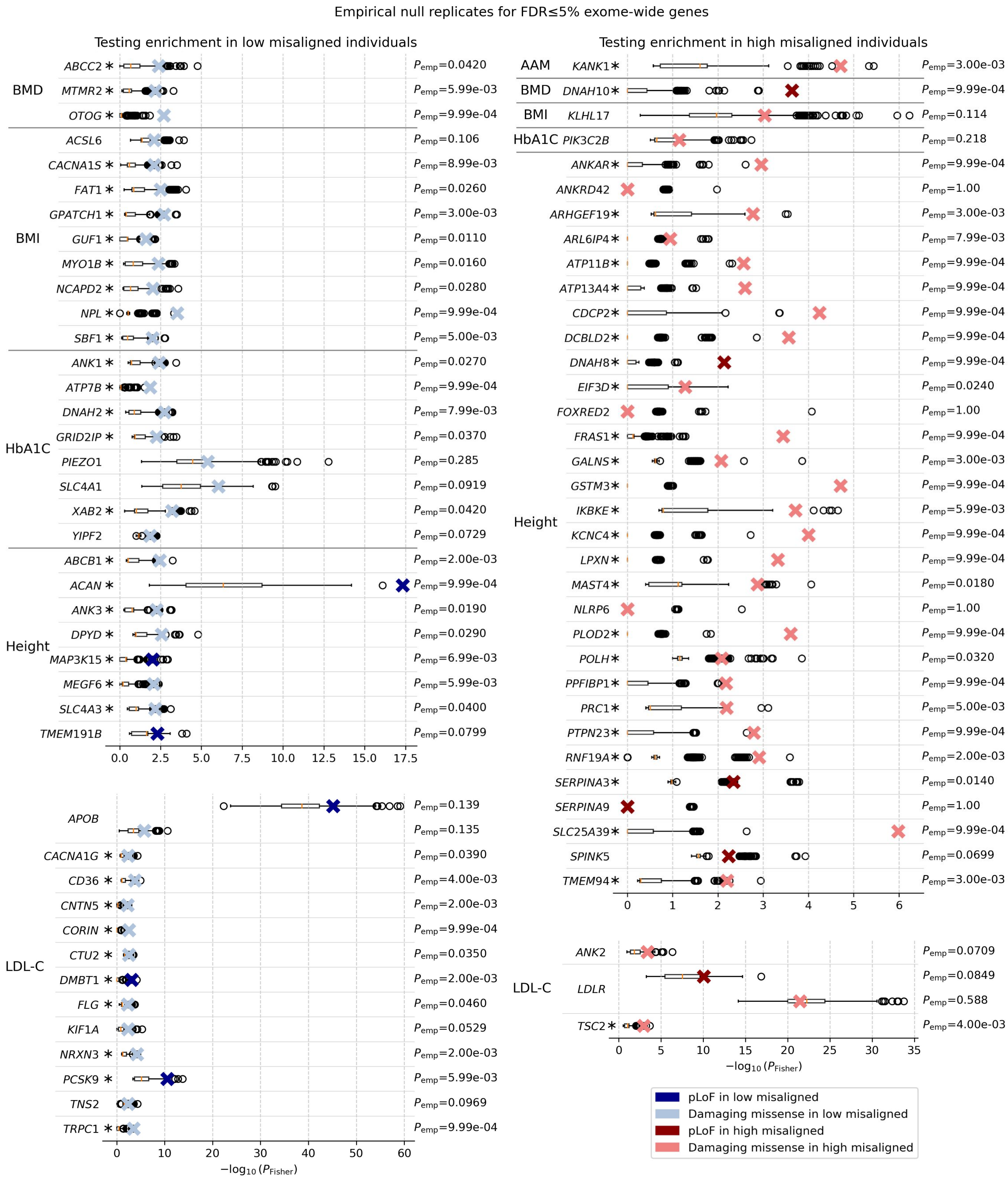

Figure S12: Empirical null replicates for FDR-significant genes nominated by the exome-wide scan. The empirical P-value (P_{emp}, labeled on the right of each plot) of a gene enrichment result indicates how the observed test statistic (one-sided Fisher's exact test P-value) compares against the empirical null distribution of test statistics (Methods). Asterisks immediately to the right of the gene symbol indicate tests where the observed enrichment outperformed the empirical null ($P_{\text{emp}} < 0.05$). LDL-C is plotted on a different x-axis scale because it tends to have stronger associations.

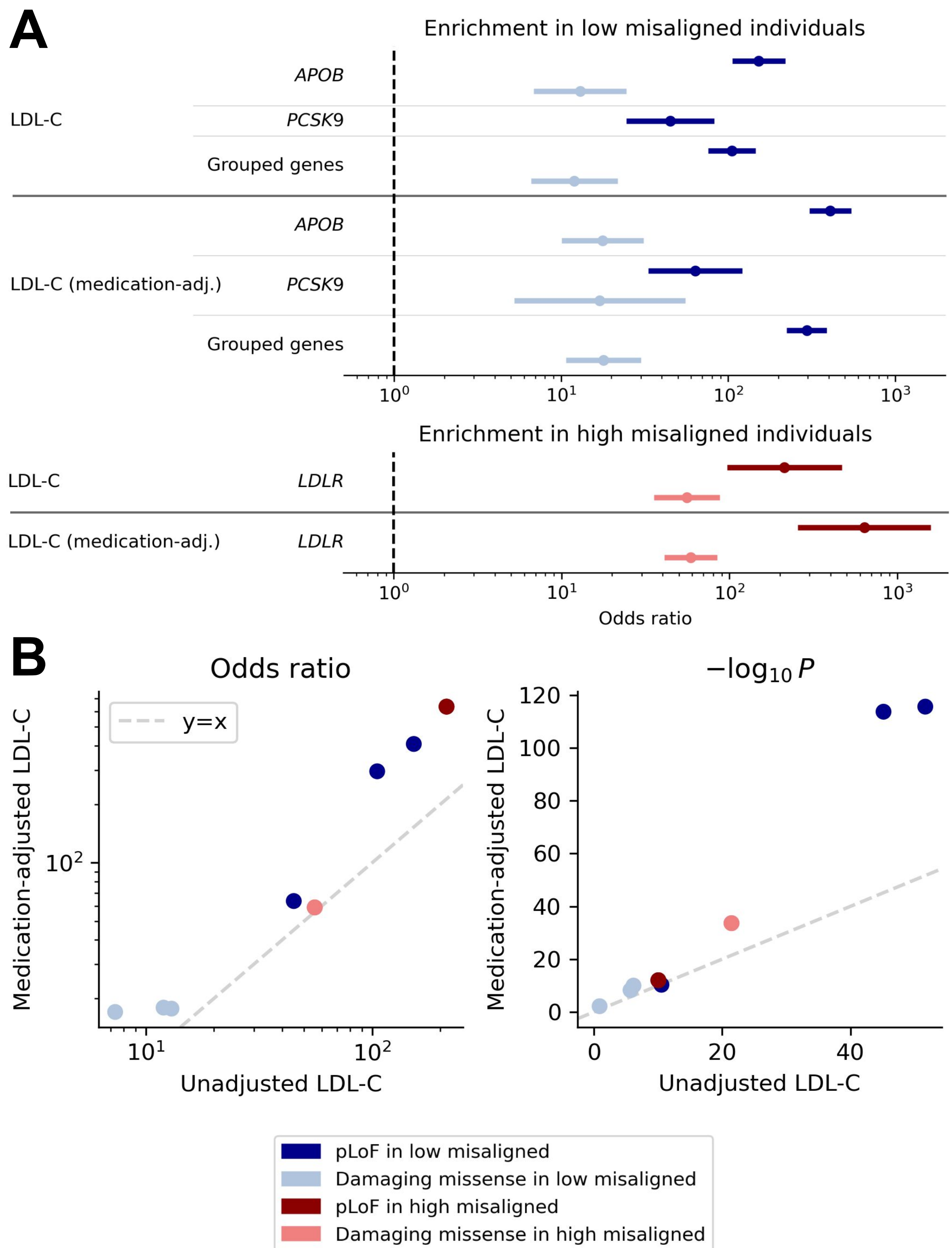

Figure S13: Comparing canonical gene enrichment results for unadjusted LDL-C to LDL-C adjusted for cholesterol-lowering medication. (**A**) Forest plot of canonical gene enrichment results. Error bars represent 90% confidence intervals. (**B**) Comparison of odds ratios and $-\log_{10} P$ for unadjusted (x-axis) and medication-adjusted (y-axis) LDL-C, where P is calculated from a one-sided Fisher's exact test. See Note S3 for description of cholesterol-lowering medication adjustment.

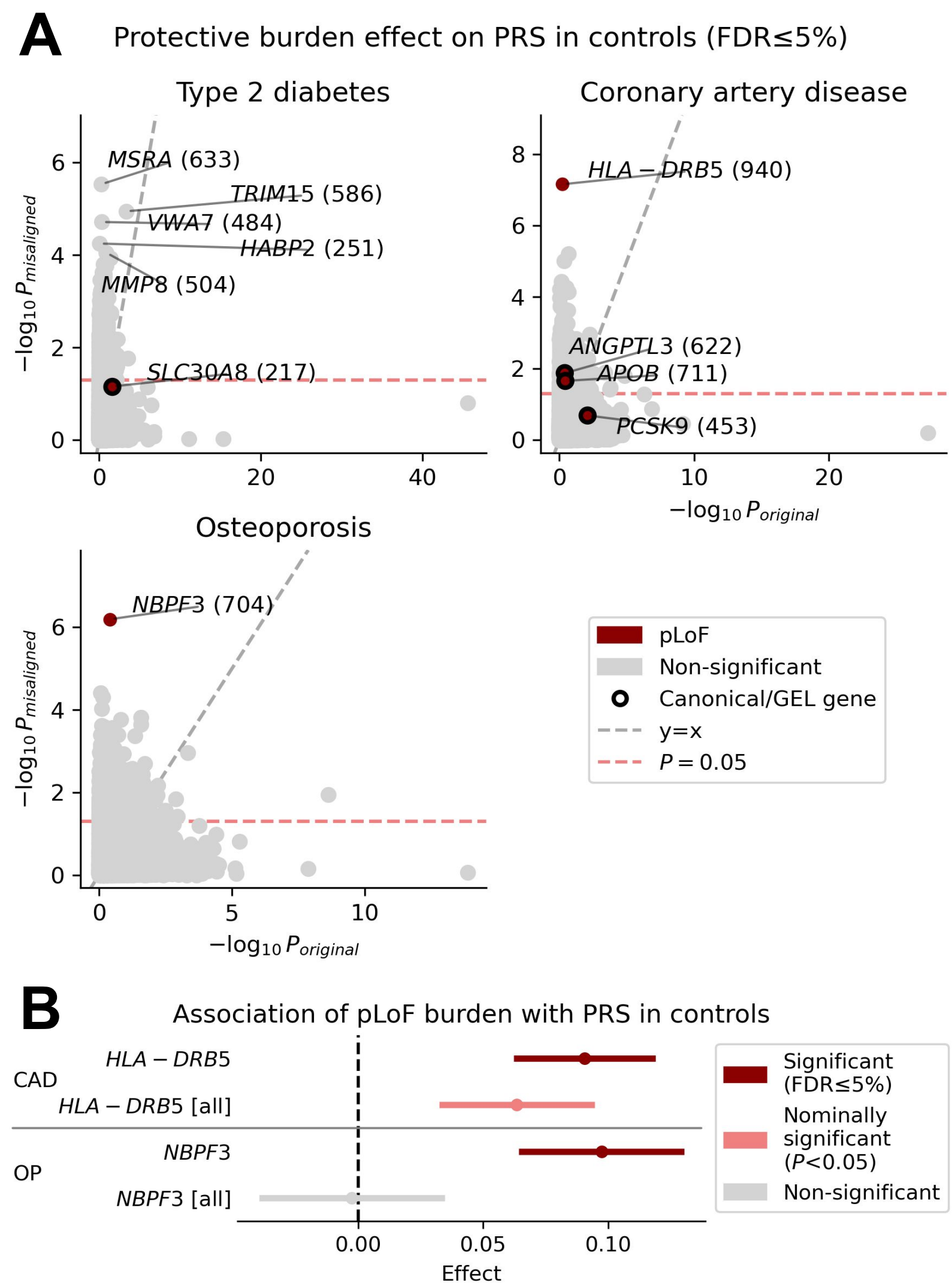

Figure S14: Strength of protective burden association with PRS in disease controls. (**A**) Comparison of strength of burden association between original trait (disease case/control status) and protective misaligned effect (effect of pLoF burden on PRS in controls). $P_{\text{misaligned}}$ is a one-sided P-value, with the alternate hypothesis that burden of pLoF variants is associated with higher PRS in disease controls. P_{original} is a two-sided P-value for the burden association with the original case-control disease trait. Significant genes are defined as having FDR-adjusted one-sided $P_{\text{misaligned}} \leq 0.05$ and are labeled with their gene symbol and, in parentheses, the total gene-level alternate allele count from REGENIE for variants included in the gene burden mask. Canonical and GEL genes are also labeled. Only genes with total alternate allele count ≥ 10 are shown. The total number of significant genes for each disease is shown in parentheses in the title of each plot. (**B**) Protective burden effects in FDR-significant genes when not conditioned or conditioned (indicated with "[all]" tag) on all common (MAF $> 0.1\%$) QCed imputed variants in or within a 1 Mb radius of the gene tested (Methods). PRS, polygenic risk scores; pLoF, predicted loss-of-function; FDR, false discovery rate

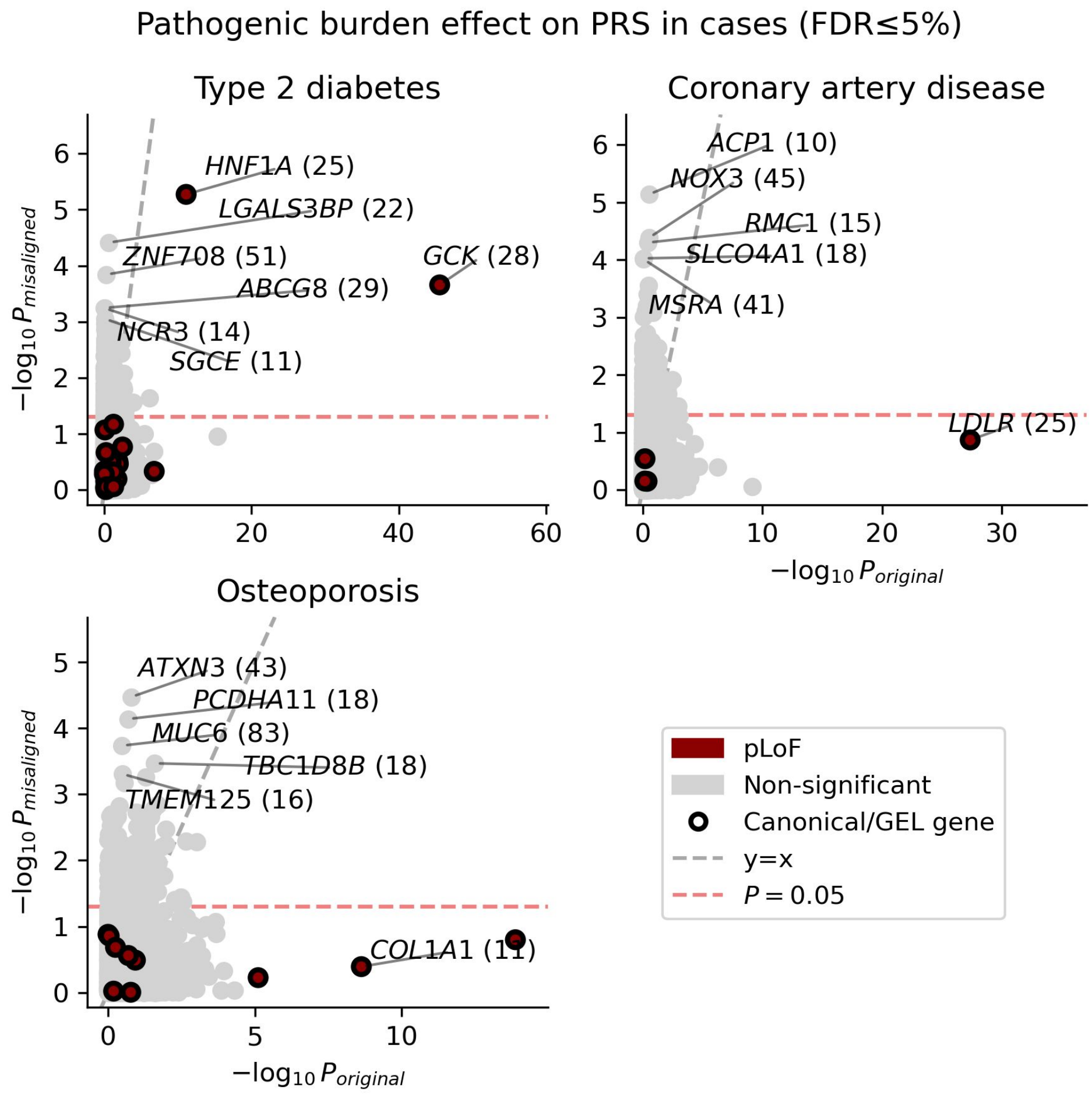

Figure S15: Strength of pathogenic burden association with PRS in disease cases. $P_{\text{misaligned}}$ is a one-sided P-value, with the alternate hypothesis that burden of pLoF variants is associated with lower PRS in disease controls. P_{original} is a two-sided P-value for the burden association with the original case-control disease trait. Significant genes, of which there were none for the pathogenic burden association testing shown here, are defined as having FDR-adjusted one-sided $P_{\text{misaligned}} \leq 0.05$. The top five genes for each trait with the strongest association strength (highest $-\log_{10} P_{\text{misaligned}}$) are labeled with their gene symbol and, in parentheses, the total gene-level alternate allele count from REGENIE for variants included in the gene burden mask. Canonical and GEL genes are also labeled. Only genes with total alternate allele count ≥ 10 are shown. PRS, polygenic risk scores; pLoF, predicted loss-of-function; FDR, false discovery rate

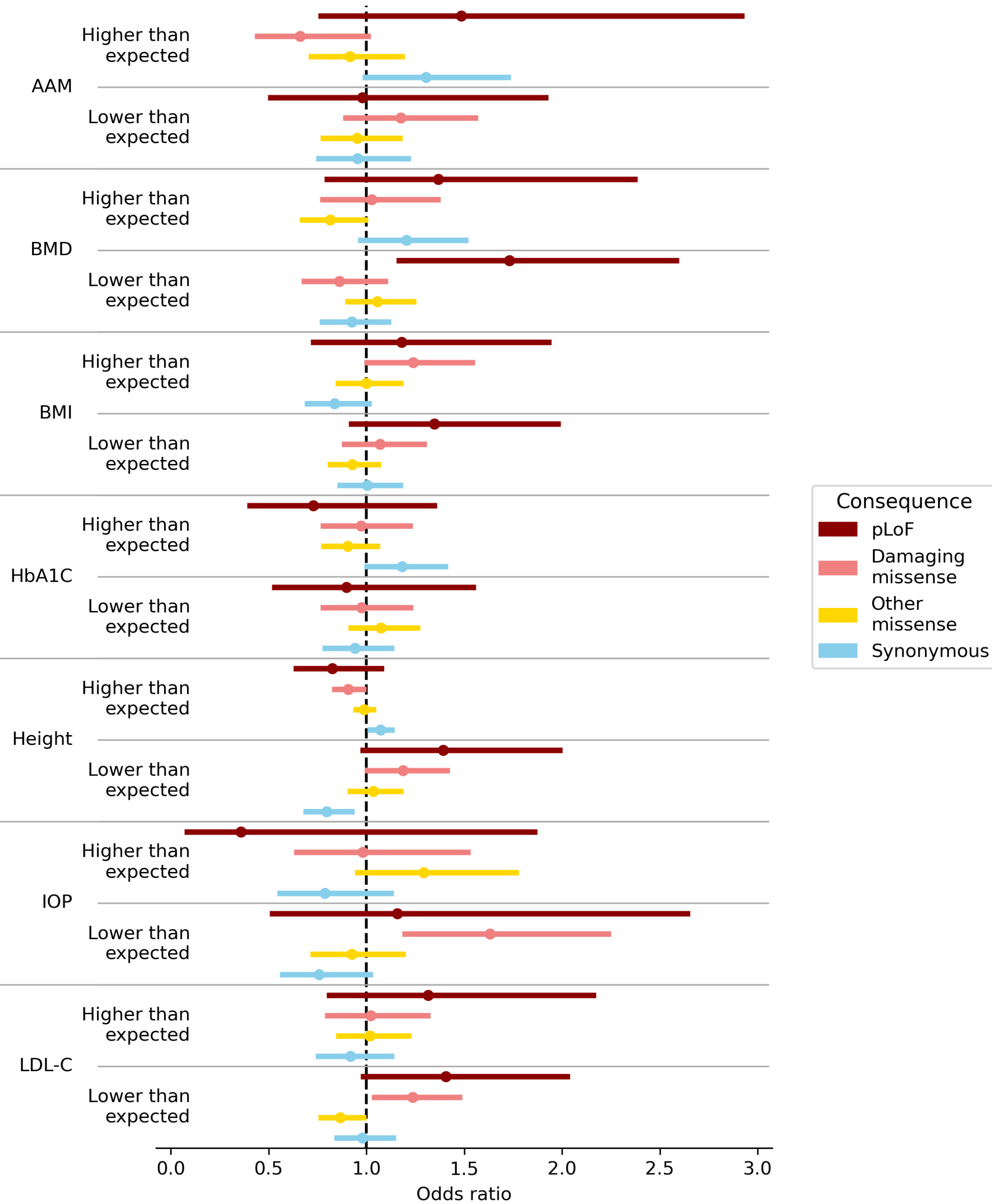

Figure S16: Enrichment of singleton burden in intolerant genes among misaligned individuals for continuous traits. Error bars are two-sided 90% CIs for OR, such that the lower error bar indicates the lower bound of the one-sided 95% CI of the OR from logistic regression of misaligned status against number of singletons, adjusted for total number of singletons (Methods).

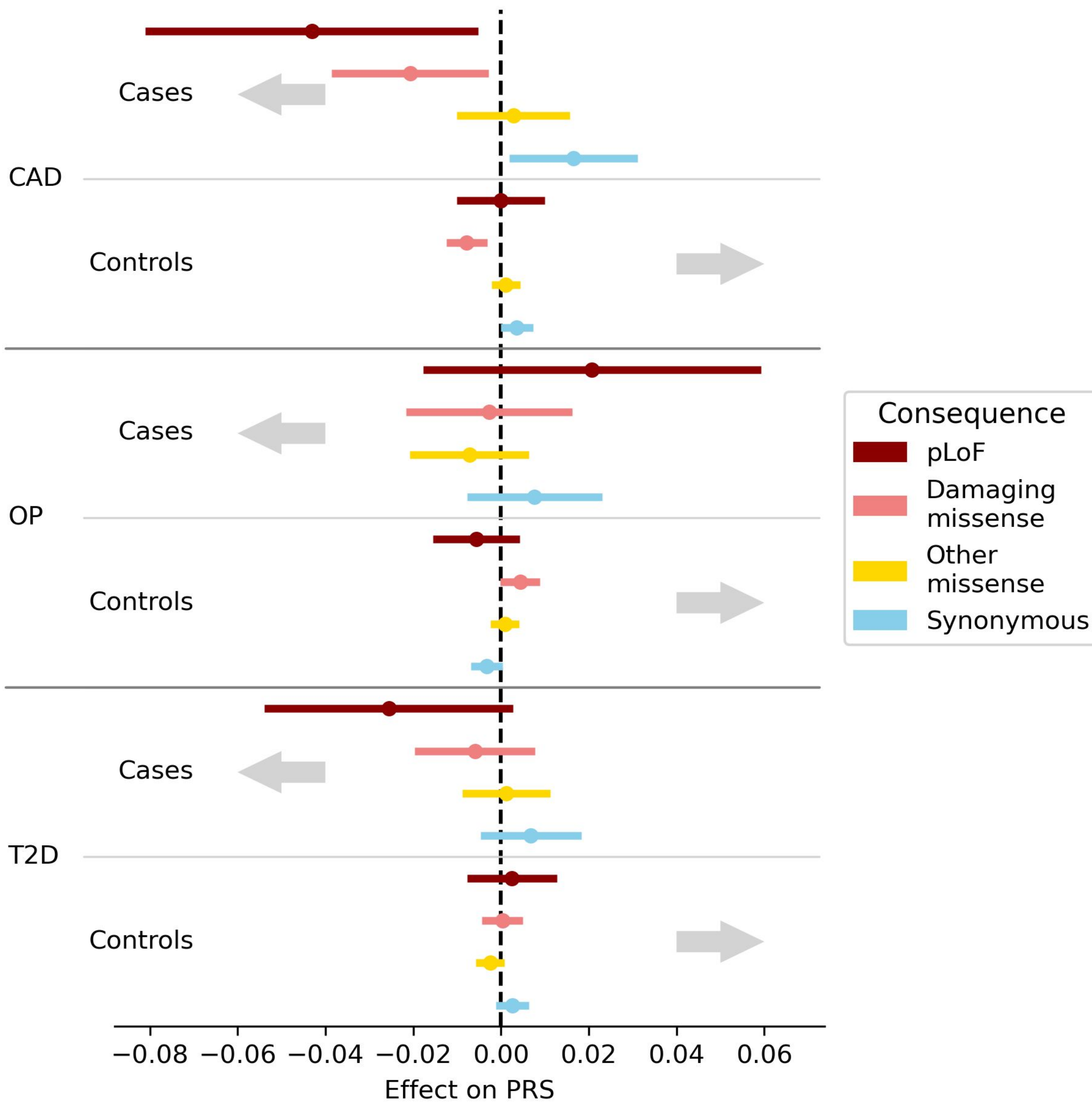

Figure S17: Enrichment of singleton burden in intolerant genes among misaligned individuals for disease traits. Error bars are two-sided 90% CIs for the effect of consequence-specific singleton burden on PRS (with total number of singletons as a covariate; see Methods), such that the upper bound (when testing cases) and lower bound (when testing controls) correspond to the upper and lower bounds, respectively, of the one-sided 95% CI. Grey arrows indicate the hypothesized direction of effect for the one-sided tests.

Supplemental tables

For supplemental tables 2-3 and 5-10, see attached supplemental material.

Phenotype (abbreviation)	pheno_id	Original phenotype	UKB data field(s) used	ICD10 code(s)	N total (subset to unrelated)	N misaligned			N aligned	N cases (subset to unrelated)	N controls (subset to unrelated)	Case prevalence	PGS	PGS UKB data field	R^2 ('PRS_only', from Thompson et al. 2024 Supp Table 4)	AUC ('PRS_only' from Thompson et al. 2024 Supp Table 3)	GEL disorder (PanelApp reviewed 'green' genes)
						Low	High	Total									
Standing height	height	Standing height	50	-	343025	185	318	503	235731	-	-	-	Standard PRS for height (HEIGHT)	26240	0.089	-	Monogenic short stature (66)<br>Beckwith-Wiedemann syndrome and other congenital overgrowth disorders (24)
LDL cholesterol (LDL-C)	ldl	LDL direct	30780	-	329391	175	109	284	225815	-	-	-	Standard PRS for low density lipoprotein cholesterol (LDL_SF)	26250	0.162	-	Familial hypercholesterolemia (9)
Body mass index (BMI)	bmi	BMI	21001	-	342697	170	111	281	234617	-	-	-	Standard PRS for body mass index (BMI)	26216	0.076	-	Severe early onset obesity (33)
Bone mineral density (BMD)	bmd	Heel BMD t-score automated	78	-	203733	122	86	208	139485	-	-	-	Standard PRS for estimated bone mineral density t-score (EBMDT)	26234	0.043	-	Osteogenesis imperfecta (34)
Intraocular pressure (IOP)	iopccmean	Intra-ocular pressure, corneal-compensated	5262, 5254	-	79598	36	32	68	54315	-	-	-	Standard PRS for intraocular pressure (IOP)	26246	0.014	-	Glaucoma, developmental (18)
Glycated haemoglobin (HbA1C)	hba1c	Glycated haemoglobin	30750	-	330025	127	111	238	225972	-	-	-	Standard PRS for glycated haemoglobin (HBA1C_DF)	26238	0.077	-	Monogenic diabetes (35)<br>Neonatal diabetes (34)
Age at menopause (AAM)	aam	Age at menopause (last menstrual period)	3581	-	117246	86	55	141	80964	-	-	-	Standard PRS for age at menopause (AAM)	26202	0.102	-	Primary ovarian insufficency (28)
Type 2 diabetes (T2D)	t2d	-	41270	E11	316377	-	-	-	-	31716	284661	0.1002	Standard PRS for type 2 diabetes (T2D)	26285	-	0.704	Monogenic diabetes (35)<br>Neonatal diabetes (34)
Coronary artery disease (CAD)	cad	-	41270	I25.1	314486	-	-	-	-	21149	293337	0.0672	Standard PRS for coronary artery disease (CAD)	26227	-	0.665	Familial hypercholesterolemia (9)<br>Extreme early-onset hypertension (15)<br>Pulmonary arterial hypertension (11)
Osteoporosis (OP)	osteoporosis	-	41270	M80+M81	313793	-	-	-	-	17650	296143	0.0562	Standard PRS for osteoporosis (OP)	26258	-	0.619	Osteogenesis imperfecta (34)

Table S1: Phenotype/PGS overview.

pheno_id	outlier_type	variable_tested	odds_ratio_90ci_fmt	pval_fisher	n_inliers	n_outliers	n_inliers_with_variable_true	n_outliers_with_variable_true	n	n_outlier	n_inlier	beta_90ci_fmt	pval_1sided_fmt
hba1c	low_outlier	atc_medication_code_prefix_A10B	1.24 [0.12, 12.73]	0.8807896528	225972	127	717	0					
bmd	high_outlier	atc_medication_code_prefix_M05B	0.79 [0.15, 4.14]	0.8158068538	139485	86	2044	1					
ldl	low_outlier	atc_medication_code_prefix_C10	11.67 [8.98, 15.17]	1.21E-53	225815	175	31844	115					
ldl	high_outlier	ever_smoked	1.52 [1.08, 2.14]	0.02625329098	224978	109	135626	76					
bmi	high_outlier	ever_smoked	0.7 [0.51, 0.96]	0.975007912	233706	110	143610	58					
bmd	low_outlier	ever_smoked	1.56 [1.13, 2.16]	0.01415429231	138977	121	83674	85					
iopccmean	high_outlier	ever_smoked	0.79 [0.44, 1.42]	0.8041299888	54065	32	33524	18					
hba1c	high_outlier	ever_smoked	1.43 [1.02, 1.99]	0.04831329822	225135	111	135911	76					
aam	low_outlier	ever_smoked	1.26 [0.88, 1.82]	0.1730638236	80713	86	46148	54					
height	low_outlier	tdi							235630	184	235446	0.174 [0.135, 0.212]	6.53E-14
ldl	high_outlier	tdi							225642	109	225533	0.0737 [0.0183, 0.129]	0.01434
bmi	high_outlier	tdi							234435	111	234324	0.187 [0.137, 0.236]	2.63E-10
bmd	low_outlier	tdi							139471	122	139349	0.0669 [0.0153, 0.118]	0.01648
iopccmean	high_outlier	tdi							54289	32	54257	0.118 [0.0127, 0.223]	0.03264
hba1c	high_outlier	tdi							225804	111	225693	0.17 [0.12, 0.22]	1.07E-08
aam	low_outlier	tdi							80952	86	80866	0.0904 [0.0275, 0.153]	9.00E-03
ldl	low_outlier	met_score							177506	132	177374	5.38e-06 [-4.78e-05, 5.86e-05]	0.4339
bmi	low_outlier	met_score							185280	127	185153	5.21e-05 [4.41e-06, 9.97e-05]	0.03615
bmd	high_outlier	met_score							108554	64	108490	4.38e-05 [-2.63e-05, 0.000114]	0.1521
iopccmean	low_outlier	met_score							43138	24	43114	4.91e-05 [-6.27e-05, 0.000161]	0.235
hba1c	low_outlier	met_score							177168	100	177068	-1.72e-06 [-6.42e-05, 6.07e-05]	0.5181
aam	high_outlier	met_score							60637	36	60601	5.11e-05 [-4.83e-05, 0.00015]	0.199
t2d	low_outlier	atc_medication_code_prefix_A10B										0.791 [0.737, 0.845]	2.12E-126
osteoporosis	low_outlier	atc_medication_code_prefix_M05B										0.281 [0.253, 0.309]	1.67E-61
cad	low_outlier	atc_medication_code_prefix_C10										0.181 [0.173, 0.188]	0.00E+00
t2d	high_outlier	tdi										-0.00349 [-0.00607, -0.000916]	0.01287
cad	high_outlier	tdi										-0.00109 [-0.0044, 0.00222]	0.2944
osteoporosis	high_outlier	tdi										-0.00469 [-0.00829, -0.00109]	0.01601
t2d	high_outlier	ever_smoked										-0.0391 [-0.0574, -0.0209]	2.12E-04
cad	high_outlier	ever_smoked										-0.00114 [-0.0251, 0.0228]	0.4687
osteoporosis	high_outlier	ever_smoked										-0.0117 [-0.0352, 0.0117]	0.2054
t2d	low_outlier	met_score										3.69e-06 [2.46e-06, 4.92e-06]	3.80E-07
cad	low_outlier	met_score										6.15e-07 [-6.08e-07, 1.84e-06]	0.204
osteoporosis	low_outlier	met_score										-2.02e-07 [-1.39e-06, 9.89e-07]	0.6099

Table S4: Association of non-genetic factors with phenotype misalignment.

pheno_id	csq_subset	max_maf	outlier_type	gene_symbol	n_total	n_inlier	n_outlier_type	n_inlier_w_variant	n_outlier_type_w_variant	n_alt	n_alt_in_subset	n_alt_in_inliers	n_alt_in_outlier_type	odds_ratio_90ci_fmt	pval_fisher_1sided_fmt
ldl_adj	pLoF	0.001	low_outlier	APOB	329391	226345	169	267	55	1107	572	516	56	409 [306-545]	2.20E-114
ldl_adj	damaging_missense	0.001	low_outlier	APOB	329391	226345	169	716	9	1143	725	716	9	17.7 [10.1-31.2]	5.31E-09
ldl_adj	pLoF	0.001	low_outlier	PCSK9	329391	226345	169	154	7	498	164	156	8	63.5 [33.2-121]	5.02E-11
ldl_adj	damaging_missense	0.001	low_outlier	PCSK9	329391	226345	169	159	2	273	161	159	2	17.0 [5.25-55.3]	6.59E-03
ldl_adj	pLoF	0.001	low_outlier	grouped:APOB-F	329391	226345	169	421	60	1605	736	672	64	295 [224-389]	2.64E-116
ldl_adj	damaging_missense	0.001	low_outlier	grouped:APOB-F	329391	226345	169	875	11	1416	886	875	11	17.9 [10.7-30.1]	1.02E-10
ldl_adj	pLoF	0.001	high_outlier	LDLR	329391	226345	183	10	5	93	15	10	5	636 [256-1580]	9.72E-13
ldl_adj	damaging_missense	0.001	high_outlier	LDLR	329391	226345	183	607	25	1248	634	609	25	58.8 [41.0-84.4]	2.39E-34
ldl	pLoF	0.001	low_outlier	APOB	329391	225815	175	258	26	1107	524	498	26	153 [106-220]	7.27E-46
ldl	damaging_missense	0.001	low_outlier	APOB	329391	225815	175	722	7	1143	729	722	7	13.0 [6.87-24.6]	1.95E-06
ldl	pLoF	0.001	low_outlier	PCSK9	329391	225815	175	240	8	498	252	244	8	45.0 [24.6-82.4]	2.97E-11
ldl	damaging_missense	0.001	low_outlier	PCSK9	329391	225815	175	177	1	273	178	177	1	7.33 [1.40-38.3]	0.129
ldl	pLoF	0.001	low_outlier	grouped:APOB-F	329391	225815	175	498	33	1605	776	742	34	105 [75.9-146]	2.25E-52
ldl	damaging_missense	0.001	low_outlier	grouped:APOB-F	329391	225815	175	899	8	1416	907	899	8	12.0 [6.59-21.8]	6.71E-07
ldl	pLoF	0.001	high_outlier	LDLR	329391	225815	109	51	5	93	57	52	5	213 [96.8-468]	8.92E-11
ldl	damaging_missense	0.001	high_outlier	LDLR	329391	225815	109	695	16	1248	713	697	16	55.7 [35.5-87.4]	3.56E-22

Table S11: LDL-C adjusted for cholesterol-lowering medication - Canonical genes associated with monogenic disorders.

pheno_id	outlier_type	csq_subset	n_inlier	n_outlier_type	n_alt	n_alt_in_subset	odds_ratio_90ci_fmt	pval_singletonburden_n_alt_1sided_fmt	qval_singletonburden_n_alt_1sided_fmt
aam	high_outlier	pLoF	80964	55	9131	6185	1.49 [0.753-2.93]	0.169	0.609
aam	high_outlier	damaging_missense	80964	55	50824	35379	0.661 [0.428-1.02]	0.941	0.969
aam	high_outlier	other_missense	80964	55	185646	129279	0.917 [0.703-1.20]	0.705	0.969
aam	high_outlier	synonymous	80964	55	92961	64948	1.31 [0.980-1.74]	0.063	0.373
aam	low_outlier	pLoF	80964	86	9131	6185	0.980 [0.497-1.93]	0.52	0.969
aam	low_outlier	damaging_missense	80964	86	50824	35402	1.18 [0.880-1.57]	0.179	0.609
aam	low_outlier	other_missense	80964	86	185646	129319	0.952 [0.765-1.18]	0.645	0.969
aam	low_outlier	synonymous	80964	86	92961	64957	0.954 [0.742-1.23]	0.62	0.969
bmd	high_outlier	pLoF	139485	86	16479	11177	1.37 [0.785-2.39]	0.177	0.609
bmd	high_outlier	damaging_missense	139485	86	90571	62018	1.03 [0.764-1.38]	0.443	0.956
bmd	high_outlier	other_missense	139485	86	325773	222913	0.815 [0.659-1.01]	0.943	0.969
bmd	high_outlier	synonymous	139485	86	162238	111165	1.21 [0.956-1.52]	0.0926	0.432
bmd	low_outlier	pLoF	139485	122	16479	11185	1.73 [1.15-2.60]	0.013	0.365
bmd	low_outlier	damaging_missense	139485	122	90571	62030	0.861 [0.669-1.11]	0.834	0.969
bmd	low_outlier	other_missense	139485	122	325773	223002	1.06 [0.890-1.26]	0.297	0.792
bmd	low_outlier	synonymous	139485	122	162238	111187	0.925 [0.760-1.13]	0.742	0.969
bmi	high_outlier	pLoF	234617	111	28165	18971	1.18 [0.716-1.95]	0.292	0.792
bmi	high_outlier	damaging_missense	234617	111	156722	106524	1.24 [0.988-1.56]	0.0598	0.373
bmi	high_outlier	other_missense	234617	111	565886	386034	1.00 [0.842-1.19]	0.498	0.969
bmi	high_outlier	synonymous	234617	111	283751	193683	0.838 [0.684-1.03]	0.925	0.969
bmi	low_outlier	pLoF	234617	170	28165	18978	1.35 [0.910-1.99]	0.106	0.455
bmi	low_outlier	damaging_missense	234617	170	156722	106539	1.07 [0.873-1.31]	0.295	0.792
bmi	low_outlier	other_missense	234617	170	565886	386098	0.928 [0.801-1.07]	0.798	0.969
bmi	low_outlier	synonymous	234617	170	283751	193731	1.00 [0.851-1.19]	0.481	0.969
hba1c	high_outlier	pLoF	225972	111	27031	18167	0.729 [0.390-1.36]	0.798	0.969
hba1c	high_outlier	damaging_missense	225972	111	150710	103003	0.973 [0.766-1.24]	0.574	0.969
hba1c	high_outlier	other_missense	225972	111	543893	374008	0.905 [0.767-1.07]	0.837	0.969
hba1c	high_outlier	synonymous	225972	111	272601	187555	1.18 [0.988-1.42]	0.062	0.373
hba1c	low_outlier	pLoF	225972	127	27031	18169	0.898 [0.517-1.56]	0.626	0.969
hba1c	low_outlier	damaging_missense	225972	127	150710	103003	0.974 [0.766-1.24]	0.571	0.969
hba1c	low_outlier	other_missense	225972	127	543893	374019	1.07 [0.906-1.27]	0.243	0.757
hba1c	low_outlier	synonymous	225972	127	272601	187532	0.940 [0.774-1.14]	0.7	0.969
height	high_outlier	pLoF	235731	318	28204	18957	0.826 [0.627-1.09]	0.872	0.969
height	high_outlier	damaging_missense	235731	318	156889	106309	0.908 [0.825-0.999]	0.951	0.969
height	high_outlier	other_missense	235731	318	566455	384251	0.989 [0.932-1.05]	0.616	0.969
height	high_outlier	synonymous	235731	318	284066	192493	1.07 [1.01-1.14]	0.0381	0.373
height	low_outlier	pLoF	235731	185	28204	18939	1.39 [0.969-2.00]	0.0667	0.373
height	low_outlier	damaging_missense	235731	185	156889	106021	1.19 [0.990-1.43]	0.0598	0.373
height	low_outlier	other_missense	235731	185	566455	382709	1.04 [0.903-1.19]	0.335	0.853
height	low_outlier	synonymous	235731	185	284066	191501	0.798 [0.677-0.939]	0.988	0.988
iopccmean	high_outlier	pLoF	54315	32	6567	4458	0.359 [0.0688-1.87]	0.846	0.969
iopccmean	high_outlier	damaging_missense	54315	32	37418	25742	0.982 [0.629-1.53]	0.527	0.969
iopccmean	high_outlier	other_missense	54315	32	137618	93565	1.29 [0.941-1.78]	0.0918	0.432
iopccmean	high_outlier	synonymous	54315	32	69953	47946	0.788 [0.545-1.14]	0.856	0.969
iopccmean	low_outlier	pLoF	54315	36	6567	4461	1.16 [0.504-2.65]	0.386	0.94
iopccmean	low_outlier	damaging_missense	54315	36	37418	25759	1.63 [1.18-2.25]	6.25E-03	0.35
iopccmean	low_outlier	other_missense	54315	36	137618	93580	0.925 [0.713-1.20]	0.688	0.969
iopccmean	low_outlier	synonymous	54315	36	69953	47957	0.759 [0.558-1.03]	0.929	0.969
ldl	high_outlier	pLoF	225815	109	27085	18450	1.32 [0.796-2.17]	0.185	0.609
ldl	high_outlier	damaging_missense	225815	109	150355	103468	1.02 [0.788-1.33]	0.444	0.956
ldl	high_outlier	other_missense	225815	109	542792	373745	1.02 [0.844-1.23]	0.435	0.956
ldl	high_outlier	synonymous	225815	109	272283	187994	0.919 [0.739-1.14]	0.737	0.969
ldl	low_outlier	pLoF	225815	175	27085	18459	1.41 [0.969-2.04]	0.0658	0.373
ldl	low_outlier	damaging_missense	225815	175	150355	103517	1.24 [1.03-1.49]	0.0294	0.373
ldl	low_outlier	other_missense	225815	175	542792	373850	0.867 [0.753-0.999]	0.951	0.969
ldl	low_outlier	synonymous	225815	175	272283	188061	0.981 [0.835-1.15]	0.58	0.969

Table S12: Singleton burden enrichment – Continuous traits.

pheno_id	outlier_type	csq_subset	n_case	n_ctrl	n_case_w_variant	n_ctrl_w_variant	beta_n_alt_90ci	pval_n_alt_1sided	qval_n_alt_1sided	q_low	q_high	odds_ratio_90ci	pval_fisher_1sided	qval_fisher_1sided
t2d	low_outlier	pLoF	31716	284661	2788	22147	2.46e-03 [-7.75e-03, 0.0127]	0.346	0.757	0.5	0.5	1.01 [0.989, 1.04]	0.190	0.880
t2d	low_outlier	pLoF	31716	284661	2788	22147	2.46e-03 [-7.75e-03, 0.0127]	0.346	0.757	0.25	0.75	1.000 [0.967, 1.03]	0.510	0.998
t2d	low_outlier	pLoF	31716	284661	2788	22147	2.46e-03 [-7.75e-03, 0.0127]	0.346	0.757	0.1	0.9	1.01 [0.955, 1.06]	0.417	0.998
t2d	high_outlier	pLoF	31716	284661	2788	22147	-0.0256 [-0.0539, 2.74e-03]	0.0687	0.757	0.5	0.5	1.06 [0.986, 1.14]	0.0959	0.780
t2d	high_outlier	pLoF	31716	284661	2788	22147	-0.0256 [-0.0539, 2.74e-03]	0.0687	0.757	0.25	0.75	1.06 [0.948, 1.20]	0.196	0.880
t2d	high_outlier	pLoF	31716	284661	2788	22147	-0.0256 [-0.0539, 2.74e-03]	0.0687	0.757	0.1	0.9	0.962 [0.769, 1.20]	0.632	0.998
t2d	low_outlier	damaging_misse	31716	284661	11081	99868	3.58e-04 [-4.31e-03, 5.03e-03]	0.450	0.757	0.5	0.5	1.00 [0.992, 1.02]	0.278	0.978
t2d	low_outlier	damaging_misse	31716	284661	11081	99868	3.58e-04 [-4.31e-03, 5.03e-03]	0.450	0.757	0.25	0.75	0.994 [0.976, 1.01]	0.715	0.998
t2d	low_outlier	damaging_misse	31716	284661	11081	99868	3.58e-04 [-4.31e-03, 5.03e-03]	0.450	0.757	0.1	0.9	0.975 [0.947, 1.00]	0.918	0.998
t2d	high_outlier	damaging_misse	31716	284661	11081	99868	-5.94e-03 [-0.0197, 7.77e-03]	0.238	0.757	0.5	0.5	1.03 [0.984, 1.07]	0.156	0.880
t2d	high_outlier	damaging_misse	31716	284661	11081	99868	-5.94e-03 [-0.0197, 7.77e-03]	0.238	0.757	0.25	0.75	1.02 [0.955, 1.10]	0.299	0.978
t2d	high_outlier	damaging_misse	31716	284661	11081	99868	-5.94e-03 [-0.0197, 7.77e-03]	0.238	0.757	0.1	0.9	1.02 [0.898, 1.15]	0.423	0.998
t2d	low_outlier	other_missense	31716	284661	23329	213522	-2.46e-03 [-5.79e-03, 8.66e-04]	0.888	0.757	0.5	0.5	0.995 [0.981, 1.01]	0.735	0.998
t2d	low_outlier	other_missense	31716	284661	23329	213522	-2.46e-03 [-5.79e-03, 8.66e-04]	0.888	0.757	0.25	0.75	0.988 [0.968, 1.01]	0.837	0.998
t2d	low_outlier	other_missense	31716	284661	23329	213522	-2.46e-03 [-5.79e-03, 8.66e-04]	0.888	0.757	0.1	0.9	0.995 [0.963, 1.03]	0.609	0.998
t2d	high_outlier	other_missense	31716	284661	23329	213522	1.19e-03 [-8.81e-03, 0.0112]	0.578	0.757	0.5	0.5	0.999 [0.953, 1.05]	0.515	0.998
t2d	high_outlier	other_missense	31716	284661	23329	213522	1.19e-03 [-8.81e-03, 0.0112]	0.578	0.757	0.25	0.75	1.05 [0.970, 1.13]	0.169	0.880
t2d	high_outlier	other_missense	31716	284661	23329	213522	1.19e-03 [-8.81e-03, 0.0112]	0.578	0.757	0.1	0.9	0.880 [0.770, 1.01]	0.947	0.998
t2d	low_outlier	synonymous	31716	284661	15303	145110	2.56e-03 [-1.19e-03, 6.31e-03]	0.131	0.757	0.5	0.5	0.993 [0.981, 1.01]	0.822	0.998
t2d	low_outlier	synonymous	31716	284661	15303	145110	2.56e-03 [-1.19e-03, 6.31e-03]	0.131	0.757	0.25	0.75	1.00 [0.984, 1.02]	0.431	0.998
t2d	low_outlier	synonymous	31716	284661	15303	145110	2.56e-03 [-1.19e-03, 6.31e-03]	0.131	0.757	0.1	0.9	0.988 [0.960, 1.02]	0.760	0.998
t2d	high_outlier	synonymous	31716	284661	15303	145110	6.81e-03 [-4.68e-03, 0.0183]	0.835	0.757	0.5	0.5	1.01 [0.964, 1.05]	0.422	0.998
t2d	high_outlier	synonymous	31716	284661	15303	145110	6.81e-03 [-4.68e-03, 0.0183]	0.835	0.757	0.25	0.75	0.971 [0.908, 1.04]	0.775	0.998
t2d	high_outlier	synonymous	31716	284661	15303	145110	6.81e-03 [-4.68e-03, 0.0183]	0.835	0.757	0.1	0.9	1.00 [0.888, 1.13]	0.510	0.998
cad	low_outlier	pLoF	21149	293337	1672	23070	-7.65e-05 [-0.0101, 9.98e-03]	0.505	0.757	0.5	0.5	0.993 [0.971, 1.02]	0.703	0.998
cad	low_outlier	pLoF	21149	293337	1672	23070	-7.65e-05 [-0.0101, 9.98e-03]	0.505	0.757	0.25	0.75	0.998 [0.966, 1.03]	0.544	0.998
cad	low_outlier	pLoF	21149	293337	1672	23070	-7.65e-05 [-0.0101, 9.98e-03]	0.505	0.757	0.1	0.9	0.960 [0.912, 1.01]	0.909	0.998
cad	high_outlier	pLoF	21149	293337	1672	23070	-0.0431 [-0.0810, -5.20e-03]	0.0307	0.757	0.5	0.5	1.07 [0.980, 1.17]	0.108	0.780
cad	high_outlier	pLoF	21149	293337	1672	23070	-0.0431 [-0.0810, -5.20e-03]	0.0307	0.757	0.25	0.75	1.20 [1.06, 1.36]	0.0116	0.279
cad	high_outlier	pLoF	21149	293337	1672	23070	-0.0431 [-0.0810, -5.20e-03]	0.0307	0.757	0.1	0.9	1.18 [0.959, 1.46]	0.106	0.780
cad	low_outlier	damaging_misse	21149	293337	7156	103237	-7.77e-03 [-0.0124, -3.16e-03]	0.997	0.757	0.5	0.5	0.978 [0.965, 0.990]	0.998	0.998
cad	low_outlier	damaging_misse	21149	293337	7156	103237	-7.77e-03 [-0.0124, -3.16e-03]	0.997	0.757	0.25	0.75	0.972 [0.955, 0.990]	0.995	0.998
cad	low_outlier	damaging_misse	21149	293337	7156	103237	-7.77e-03 [-0.0124, -3.16e-03]	0.997	0.757	0.1	0.9	0.955 [0.928, 0.983]	0.996	0.998
cad	high_outlier	damaging_misse	21149	293337	7156	103237	-0.0207 [-0.0386, -2.80e-03]	0.0285	0.757	0.5	0.5	1.03 [0.977, 1.08]	0.194	0.880
cad	high_outlier	damaging_misse	21149	293337	7156	103237	-0.0207 [-0.0386, -2.80e-03]	0.0285	0.757	0.25	0.75	1.06 [0.987, 1.15]	0.0901	0.780
cad	high_outlier	damaging_misse	21149	293337	7156	103237	-0.0207 [-0.0386, -2.80e-03]	0.0285	0.757	0.1	0.9	1.12 [0.986, 1.26]	0.0773	0.780
cad	low_outlier	other_missense	21149	293337	15328	220347	1.11e-03 [-2.19e-03, 4.40e-03]	0.290	0.757	0.5	0.5	0.987 [0.974, 1.00]	0.930	0.998
cad	low_outlier	other_missense	21149	293337	15328	220347	1.11e-03 [-2.19e-03, 4.40e-03]	0.290	0.757	0.25	0.75	0.989 [0.969, 1.01]	0.826	0.998
cad	low_outlier	other_missense	21149	293337	15328	220347	1.11e-03 [-2.19e-03, 4.40e-03]	0.290	0.757	0.1	0.9	0.970 [0.940, 1.00]	0.943	0.998
cad	high_outlier	other_missense	21149	293337	15328	220347	2.86e-03 [-0.0100, 0.0157]	0.642	0.757	0.5	0.5	0.976 [0.924, 1.03]	0.780	0.998
cad	high_outlier	other_missense	21149	293337	15328	220347	2.86e-03 [-0.0100, 0.0157]	0.642	0.757	0.25	0.75	0.974 [0.899, 1.06]	0.713	0.998
cad	high_outlier	other_missense	21149	293337	15328	220347	2.86e-03 [-0.0100, 0.0157]	0.642	0.757	0.1	0.9	0.959 [0.839, 1.09]	0.715	0.998
cad	low_outlier	synonymous	21149	293337	10185	149546	3.64e-03 [-7.58e-05, 7.35e-03]	0.0536	0.757	0.5	0.5	0.997 [0.985, 1.01]	0.657	0.998
cad	low_outlier	synonymous	21149	293337	10185	149546	3.64e-03 [-7.58e-05, 7.35e-03]	0.0536	0.757	0.25	0.75	0.993 [0.976, 1.01]	0.752	0.998
cad	low_outlier	synonymous	21149	293337	10185	149546	3.64e-03 [-7.58e-05, 7.35e-03]	0.0536	0.757	0.1	0.9	0.990 [0.963, 1.02]	0.734	0.998
cad	high_outlier	synonymous	21149	293337	10185	149546	0.0166 [1.95e-03, 0.0312]	0.969	0.757	0.5	0.5	0.954 [0.909, 1.00]	0.946	0.998
cad	high_outlier	synonymous	21149	293337	10185	149546	0.0166 [1.95e-03, 0.0312]	0.969	0.757	0.25	0.75	0.980 [0.912, 1.05]	0.690	0.998
cad	high_outlier	synonymous	21149	293337	10185	149546	0.0166 [1.95e-03, 0.0312]	0.969	0.757	0.1	0.9	1.01 [0.899, 1.14]	0.448	0.998
osteoporosis	low_outlier	pLoF	17650	296143	1478	23268	-5.61e-03 [-0.0155, 4.26e-03]	0.825	0.825	0.5	0.5	0.989 [0.967, 1.01]	0.793	0.998
osteoporosis	low_outlier	pLoF	17650	296143	1478	23268	-5.61e-03 [-0.0155, 4.26e-03]	0.825	0.825	0.25	0.75	0.992 [0.960, 1.02]	0.673	0.998
osteoporosis	low_outlier	pLoF	17650	296143	1478	23268	-5.61e-03 [-0.0155, 4.26e-03]	0.825	0.825	0.1	0.9	0.963 [0.916, 1.01]	0.893	0.998
osteoporosis	high_outlier	pLoF	17650	296143	1478	23268	0.0207 [-0.0177, 0.0592]	0.812	0.825	0.5	0.5	0.932 [0.849, 1.02]	0.900	0.998
osteoporosis	high_outlier	pLoF	17650	296143	1478	23268	0.0207 [-0.0177, 0.0592]	0.812	0.825	0.25	0.75	0.953 [0.831, 1.09]	0.731	0.998
osteoporosis	high_outlier	pLoF	17650	296143	1478	23268	0.0207 [-0.0177, 0.0592]	0.812	0.825	0.1	0.9	0.782 [0.620, 0.986]	0.967	0.998
osteoporosis	low_outlier	damaging_misse	17650	296143	6086	104160	4.41e-03 [-1.10e-04, 8.92e-03]	0.0543	0.825	0.5	0.5	1.02 [1.01, 1.03]	6.09e-03	0.279
osteoporosis	low_outlier	damaging_misse	17650	296143	6086	104160	4.41e-03 [-1.10e-04, 8.92e-03]	0.0543	0.825	0.25	0.75	1.03 [1.01, 1.05]	8.44e-03	0.279
osteoporosis	low_outlier	damaging_misse	17650	296143	6086	104160	4.41e-03 [-1.10e-04, 8.92e-03]	0.0543	0.825	0.1	0.9	1.02 [0.991, 1.05]	0.132	0.865
osteoporosis	high_outlier	damaging_misse	17650	296143	6086	104160	-2.68e-03 [-0.0216, 0.0163]	0.408	0.825	0.5	0.5	1.03 [0.973, 1.08]	0.213	0.895
osteoporosis	high_outlier	damaging_misse	17650	296143	6086	104160	-2.68e-03 [-0.0216, 0.0163]	0.408	0.825	0.25	0.75	0.973 [0.899, 1.05]	0.727	0.998
osteoporosis	high_outlier	damaging_misse	17650	296143	6086	104160	-2.68e-03 [-0.0216, 0.0163]	0.408	0.825	0.1	0.9	0.935 [0.820, 1.07]	0.810	0.998
osteoporosis	low_outlier	other_missense	17650	296143	12846	222313	8.70e-04 [-2.36e-03, 4.10e-03]	0.329	0.825	0.5	0.5	1.02 [1.00, 1.03]	0.0360	0.518
osteoporosis	low_outlier	other_missense	17650	296143	12846	222313	8.70e-04 [-2.36e-03, 4.10e-03]	0.329	0.825	0.25	0.75	1.01 [0.988, 1.03]	0.264	0.978
osteoporosis	low_outlier	other_missense	17650	296143	12846	222313	8.70e-04 [-2.36e-03, 4.10e-03]	0.329	0.825	0.1	0.9	1.04 [1.01, 1.07]	0.0222	0.400
osteoporosis	high_outlier	other_missense	17650	296143	12846	222313	-7.20e-03 [-0.0208, 6.40e-03]	0.192	0.825	0.5	0.5	1.01 [0.952, 1.07]	0.417	0.998
osteoporosis	high_outlier	other_missense	17650	296143	12846	222313	-7.20e-03 [-0.0208, 6.40e-03]	0.192	0.825	0.25	0.75	0.931 [0.856, 1.01]	0.925	0.998
osteoporosis	high_outlier	other_missense	17650	296143	12846	222313	-7.20e-03 [-0.0208, 6.40e-03]	0.192	0.825	0.1	0.9	0.922 [0.804, 1.06]	0.845	0.998
osteoporosis	low_outlier	synonymous	17650	296143	8609	150840	-3.21e-03 [-6.85e-03, 4.34e-04]	0.926	0.825	0.5	0.5	0.998 [0.986, 1.01]	0.589	0.998
osteoporosis	low_outlier	synonymous	17650	296143	8609	150840	-3.21e-03 [-6.85e-03, 4.34e-04]	0.926	0.825	0.25	0.75	1.01 [0.989, 1.02]	0.288	0.978
osteoporosis	low_outlier	synonymous	17650	296143	8609	150840	-3.21e-03 [-6.85e-03, 4.34e-04]	0.926	0.825	0.1	0.9	1.01 [0.986, 1.04]	0.224	0.895
osteoporosis	high_outlier	synonymous	17650	296143	8609	150840	7.61e-03 [-7.73e-03, 0.0230]	0.793	0.825	0.5	0.5	0.971 [0.923, 1.02]	0.832	0.998
osteoporosis	high_outlier	synonymous	17650	296143	8609	150840	7.61e-03 [-7.73e-03, 0.0230]	0.793	0.825	0.25	0.75	0.910 [0.844, 0.980]	0.982	0.998
osteoporosis	high_outlier	synonymous	17650	296143	8609	150840	7.61e-03 [-7.73e-03, 0.0230]	0.793	0.825	0.1	0.9	0.885 [0.783, 1.00]	0.953	0.998

Table S13: Singleton burden enrichment – Dichotomous traits.

Supplemental references

1. Hawkes, G., Yengo, L., Vedantam, S., Marouli, E., Beaumont, R.N., GIANT Consortium, Tyrrell, J., Weedon, M.N., Hirschhorn, J., Frayling, T.M., et al. (2023). Identification and analysis of individuals who deviate from their genetically-predicted phenotype. PLoS Genet. *19*, e1010934. 10.1371/journal.pgen.1010934.
2. Wu, Y., Byrne, E.M., Zheng, Z., Kemper, K.E., Yengo, L., Mallett, A.J., Yang, J., Visscher, P.M., Wray, N.R. (2019). Genome-wide association study of medication-use and associated disease in the UK Biobank. Nat. Commun. *10*, 1891. 10.1038/s41467-019-09572-5.
3. Benjamini, Y., Hochberg, Y. (1995). Controlling the False Discovery Rate: A Practical and Powerful Approach to Multiple Testing. J. R. Stat. Soc. Ser. B Methodol. *57*, 289–300.
4. Tieber, V.L., Abruzzini, L.F., Didier, D.K., Schwartz, B.D., Rotwein, P. (1986). Complete characterization and sequence of an HLA class II DR beta chain cDNA from the DR5 haplotype. J. Biol. Chem. *261*, 2738–2742. 10.1016/S0021-9258(17)35847-7.
5. Ajoolabady, A., Pratico, D., Lin, L., Mantzoros, C.S., Bahijri, S., Tuomilehto, J., Ren, J. (2024). Inflammation in atherosclerosis: pathophysiology and mechanisms. Cell Death Dis. *15*, 817. 10.1038/s41419-024-07228-3.
6. Patel, A.P., Wang, M., Pirruccello, J.P., Ellinor, P.T., Ng, K., Kathiresan, S., Khera, A.V. (2021). Lipoprotein(a) concentrations and incident atherosclerotic cardiovascular disease: new insights from a large national biobank. Arterioscler. Thromb. Vasc. Biol. *41*, 465–474. 10.1161/ATVBAHA.120.315291.
7. Purcell, S., Neale, B., Todd-Brown, K., Thomas, L., Ferreira, M.A.R., Bender, D., Maller, J., Sklar, P., Bakker, P.I.W. de, Daly, M.J., et al. (2007). PLINK: a tool set for whole-genome association and population-based linkage analyses. Am. J. Hum. Genet. *81*, 559–575. 10.1086/519795.
8. Karczewski, K.J., Francioli, L.C., Tiao, G., Cummings, B.B., Alföldi, J., Wang, Q., Collins, R.L., Laricchia, K.M., Ganna, A., Birnbaum, D.P., et al. (2020). The mutational constraint spectrum quantified from variation in 141,456 humans. Nature *581*, 434–443. 10.1038/s41586-020-2308-7.
9. Genovese, G., Fromer, M., Stahl, E.A., Ruderfer, D.M., Chambert, K., Landén, M., Moran, J.L., Purcell, S.M., Sklar, P., Sullivan, P.F., et al. (2016). Increased burden of ultra-rare protein-altering variants among 4,877 individuals with schizophrenia. Nat. Neurosci. *19*, 1433–1441. 10.1038/nn.4402.

10. Backman, J.D., Li, A.H., Marcketta, A., Sun, D., Mbatchou, J., Kessler, M.D., Benner, C., Liu, D., Locke, A.E., Balasubramanian, S., et al. (2021). Exome sequencing and analysis of 454,787 UK Biobank participants. Nature *599*, 628–634. 10.1038/s41586-021-04103-z.

11. Hawkes, G., Beaumont, R.N., Li, Z., Mandla, R., Li, X., Albert, C.M., Arnett, D.K., Ashley-Koch, A.E., Ashrani, A.A., Barnes, K.C., et al. (2024). Whole-genome sequencing in 333,100 individuals reveals rare non-coding single variant and aggregate associations with height. Nat. Commun. *15*, 8549. 10.1038/s41467-024-52579-w.

12. Landrum, M.J., Lee, J.M., Riley, G.R., Jang, W., Rubinstein, W.S., Church, D.M., Maglott, D.R. (2014). ClinVar: public archive of relationships among sequence variation and human phenotype. Nucleic Acids Res. *42*, D980–5. 10.1093/nar/gkt1113.

13. Buniello, A., Suveges, D., Cruz-Castillo, C., Llinares, M.B., Cornu, H., Lopez, I., Tsukanov, K., Roldán-Romero, J.M., Mehta, C., Fumis, L., et al. (2025). Open Targets Platform: facilitating therapeutic hypotheses building in drug discovery. Nucleic Acids Res. *53*, D1467–D1475. 10.1093/nar/gkae1128.
