# Supplementary material for: Individuals who deviate from polygenic expectation are enriched for damaging variants in genes linked to rare disease
Source: Am J Hum Genet. 2026 Jun 22;113(7):1415–33. doi: 10.1016/j.ajhg.2026.05.013 (PMC13504349; doi:10.1016/j.ajhg.2026.05.013)
Supplement: Supplementary file 2 [file mmc3.pdf]

Individuals who deviate from polygenic expectation are enriched for damaging variants in genes linked to rare disease

Authors

Nikolas A. Baya, Frederik H. Lassen, Barney Hill, Samvida S. Venkatesh, Hannah Currant, Cecilia M. Lindgren, Duncan S. Palmer

Correspondence

nikolasbaya@gmail.com (N.A.B.),
duncan.stuart.palmer@gmail.com (D.S.P.)

Polygenic scores stratify disease risk but often miss individual variation. We show that individuals whose traits deviate from polygenic expectation are enriched for rare, damaging variants. This misalignment framework supports a liability threshold model of counteracting common and rare variant effects, offering a powerful tool to explore complex disease architecture.

Baya et al., 2026, The American Journal of Human Genetics *113*, 1415–1433
July 2, 2026 © 2026 The Author(s). Published by Elsevier Inc. on behalf of American Society of Human Genetics.
https://doi.org/10.1016/j.ajhg.2026.05.013

Individuals who deviate from polygenic expectation are enriched for damaging variants in genes linked to rare disease

Nikolas A. Baya,[1,2,3,*] Frederik H. Lassen,[1,2] Barney Hill,[1,4] Samvida S. Venkatesh,[1,2] Hannah Currant,[1,5] Cecilia M. Lindgren,[1,2,5,6,8] and Duncan S. Palmer[1,3,6,7,8,*]

Summary

Polygenic scores (PGSs) stratify disease risk but often fail to capture individual variation. "Misaligned" individuals, whose observed phenotypes deviate from their genetically expected values based on PGS, provide a powerful model for identifying factors beyond common-variant effects, including additional genetic factors. Here, we apply misalignment classification and enrichment testing frameworks to seven continuous traits and three diseases, assessing whether misaligned individuals in the UK Biobank are enriched for rare (minor-allele frequency [MAF] <0.1%) damaging genetic variation. We identified significant enrichment of predicted loss-of-function (pLoF) variants in *COPB2* and *GORAB* among individuals with lower-than-expected bone mineral density. Regarding stature, shorter-than-expected individuals were enriched for pLoF variants in *ACAN* and *IGF1*, while taller-than-expected individuals showed enrichment for damaging missense variants in *FBN1*. Transitioning from validation to discovery, we performed an exome-wide scan for genes associated with misalignment and identified 74 significant genes, including *KANK1*, a gene which may have a protective role against primary ovarian insufficiency, and *ACSL6*, a lipid metabolism gene where damaging missense variation was associated with lower-than-expected BMI. For diseases, results supported a liability threshold model involving counteracting common and rare variant effects. Diagnosed type 2 diabetes mellitus patients with rare pathogenic variants in *HNF1A* and *HNF4A* possessed significantly lower polygenic risk than those without. Conversely, coronary artery disease controls harboring protective *ANGPTL3* variants had nominally higher polygenic risk. This study highlights the power of misalignment-based analyses in complex continuous phenotypes and disease with the potential to validate known genetic contributors to traits and identify previously unassociated genes.

Introduction

Modeling *how* an individual develops disease is key to preventing disease. One approach to modeling genetic disease risk is the liability threshold model.[1,2] This model assumes a continuous "liability" for disease, comprised of genetic and environmental contributions.[2] An individual develops disease if their liability exceeds a certain threshold. The genetic risk portion of this liability can be separated into risk contributed by common variants and rare variants for a heritable trait[3] (Figure 1).

One way to quantify the common-variant genetic component of a phenotype is with polygenic scores (PGSs).[4,5] PGSs have transformed the study of complex traits by enabling the prediction of phenotypes and disease risk based on the aggregate contribution of common genetic variation.[6–9] However, given the many other contributors to phenotypic variability in the population, assessing how an individual's observed phenotype deviates from expectation based on their PGS could refine signal for other components (e.g., rare variants, environmental effects) and improve power in association studies. For example, adjusting for PGS has been demonstrated to improve power in rare-variant association analyses.[10]

There are other applications for this notion of divergence from the genetically expected phenotype measured by aggregating common-variant effects. Under a liability threshold model, individuals diagnosed with disease but with low common-variant risk may be expected to have other independent components of risk causing them to cross the liability threshold. This is consistent with recent literature showing that diagnosed individuals with low common-variant risk are more likely to harbor rare pathogenic variants[11] or have a monogenic diagnosis.[12] There are many other recent examples of work involving the interplay of polygenic and monogenic risk, especially demonstrating how common-variant polygenic risk can modulate the penetrance or expressivity of monogenic risk variants. Disorders for which this has been demonstrated include breast cancer[13–15] (MIM: 114480); Huntington disease[16,17] (MIM: 143100); glaucoma 1, open angle, A[18,19] (MIM: 137750); familial hypercholesterolemia (FH)[13,20]; obesity[20] (MIM: 601665); developmental

[1]Big Data Institute, Li Ka Shing Centre for Health Information and Discovery, University of Oxford, Oxford, UK; [2]Centre for Human Genetics, Nuffield Department of Medicine, University of Oxford, Oxford, UK; [3]Department of Statistics, University of Oxford, Oxford, UK; [4]Nuffield Department of Population Health, Medical Sciences Division, University of Oxford, Oxford, UK; [5]Nuffield Department of Women's and Reproductive Health, Medical Sciences Division, University of Oxford, Oxford, UK; [6]Broad Institute of MIT and Harvard, Cambridge, MA, USA; [7]The Pioneer Centre for SMARTbiomed, Big Data Institute, Li Ka Shing Centre for Health Information and Discovery, University of Oxford, Oxford, UK
[8]Senior author
*Correspondence: nikolasbaya@gmail.com (N.A.B.), duncan.stuart.palmer@gmail.com (D.S.P.)
https://doi.org/10.1016/j.ajhg.2026.05.013.
© 2026 The Author(s). Published by Elsevier Inc. on behalf of American Society of Human Genetics.
This is an open access article under the CC BY license (http://creativecommons.org/licenses/by/4.0/).

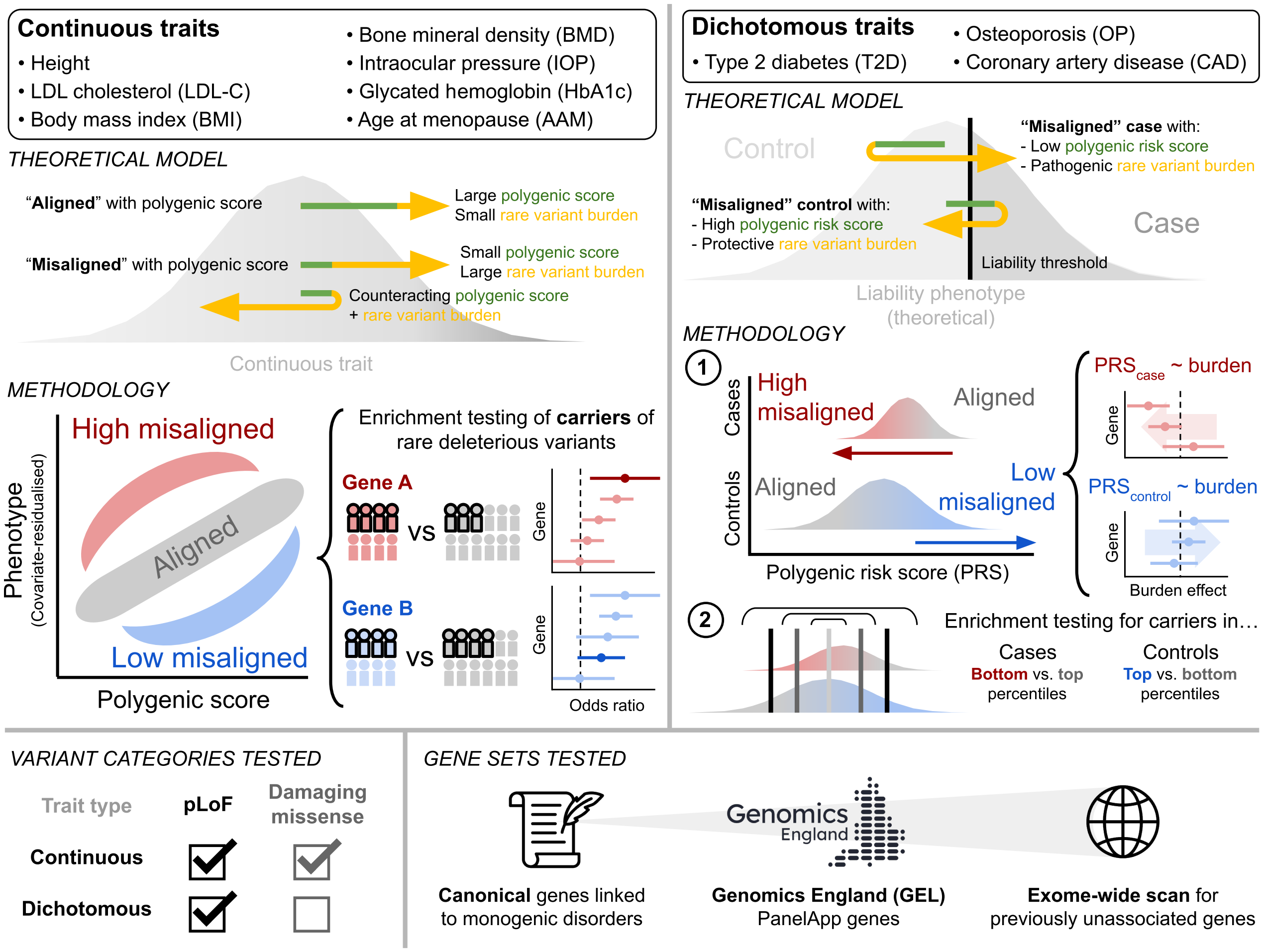

Figure 1. Overview of phenotypic misalignment analysis

We investigated seven continuous and three dichotomous traits to determine whether deviation from common-variant genetic prediction is driven by rare damaging variants. Theoretical model: for continuous traits, misaligned individuals are defined by large residuals between observed and expected phenotypes (PGSs). For dichotomous traits, under the liability threshold model, misaligned individuals include cases with low PRSs and controls with high PRSs, with misalignment driven by pathogenic or protective rare variants. Methodology: enrichment in continuous traits was assessed by comparing odds of harboring rare variants in high/low misaligned groups against aligned individuals. For dichotomous traits, we utilized a two-step process: regression of PRS against rare variant burden and enrichment testing based on PRS percentiles. Variant categories tested: we computationally annotated variants and used pLoF or putatively damaging missense variants in our enrichment and burden tests. Only pLoF variants were tested for dichotomous traits. Gene sets tested: tests were applied in stages across canonical genes linked to monogenic disorders, GEL PanelApp gene panel genes, and an exome-wide scan.

disorders[21,22]; chronic kidney disease[23]; and schizophrenia[24] (MIM: 181500).

Here, we test for enrichment of rare (minor-allele frequency [MAF] < 0.1%) deleterious variants in UK Biobank (UKB) individuals who deviate from their genetically expected phenotype based on PGS (Figure 1), using common-variant PGSs calculated by Genomics PLC[25] and provided through the UKB. We assess seven continuous traits (standing height, low-density lipoprotein cholesterol [LDL-C], BMI, glycated hemoglobin [HbA1c], bone mineral density [BMD], intraocular pressure [IOP], and age at menopause [AAM]) and three dichotomous disease status traits (type 2 diabetes mellitus ([T2D] [MIM: 125853]), coronary artery disease [CAD], and osteoporosis [OP] [MIM: 166710]) in our analysis. These traits were selected due to having polygenic and monogenic components of heritability. The selection of dichotomous traits was also motivated by the availability of their corresponding continuous "liability" traits (HbA1c for T2D, LDL-C for CAD, BMD for OP), which enabled us to use many of the same gene sets between traits. For continuous traits, we test for rare putatively deleterious variation in individuals with misaligned phenotypes using an existing framework[26] and develop a testing framework to enable the analysis of dichotomous traits.

We build the scope of our investigation in stages. We begin our enrichment testing in a small set of "canonical" genes, curated for each trait, linked to monogenic disorders or strongly associated with the trait. In this context, we have a strong prior for misaligned individuals to

show enrichment for deleterious rare variation, given previous work in a smaller subset of the UKB.[26]

We then expand our analysis to a larger set of genes considered to be of diagnostic grade for rare disorders related to our selected traits, taken from the Genomics England gene panel.[27] This approach allows us to test many more genes than the canonical genes while maintaining a hypothesis of rare genetic disorder enrichment in misaligned individuals.

Finally, we explore whether this framework of testing for deleterious variant enrichment in misaligned individuals can be re-appropriated for discovery of pathogenic or protective genetic effects. To do this, we treat misalignment as the trait of interest and perform gene-level association testing of protective and pathogenic damaging rare variation across the exome.

Altogether, this study comprises a comprehensive investigation of phenotype deviation from common-variant polygenic expectation, leveraging the data available in the UKB to enable study of misalignment at greater scale (>2× larger sample size and >3× the number of phenotypes compared to previous work in continuous traits[26]; ∼8× larger sample size for similar studies in dichotomous traits[11,12]). We unify misalignment enrichment testing of continuous liability and dichotomous disease phenotypes under the liability threshold model and provide further evidence for counteracting pathogenic/protective common- and rare-variant effects.

Methods

Exome sequencing data quality control and assigning population labels

We performed sample-, variant- and genotype-level quality control (QC) for the UKB 450K exome sequencing (ES) dataset, following the approach of Karczewski et al.[28] Broad continental genetic ancestry labels (Africans, Admixed Americans, East Asians, Europeans, South Asians) were assigned following the approach described by Lassen et al.,[29] using a random forest (RF) classifier trained on the 1000 Genomes dataset.[30] We subset to individuals with European genetic ancestry, resulting in a high-quality call set of 402,375 samples and 25,229,669 variants.

Variant consequence annotation

We annotated ES variants using Variant Effect Predictor (VEP) v.105 (corresponding to GENCODE v.39)[31] with the Loss-of-Function Transcript Effect Estimator (LOFTEE) v.1.04_GRCh38[32] and dbNSFP[33] plugins, annotating variants with Combined Annotation Dependent Depletion (CADD) v.1.6,[34] and Rare Exome Variant Ensemble Learner (REVEL)[35] using dbNSFP v.4.3 and loss-of-function (LoF) confidence using LOFTEE. We provide code and instructions for this step in our VEP_105_LOFTEE repository (https://github.com/BRaVa-genetics/vep105_loftee), which contains a Docker/Singularity container for reproducibility of annotations. Next, we ran SpliceAI v.1.3[36] using the GENCODE v.39 gene annotation file to ensure alignment between VEP and SpliceAI transcript annotations. For variant-specific annotations, we use "canonical" transcripts. We separated variants by transcript using `bcftools +split-vep` and filtered to MANE Select[37] protein-coding transcripts. If the gene lacked a MANE Select transcript, we selected the canonical transcript defined by GENCODE v.39. Using this collection of missense, predicted LoF (pLoF), splice metrics, and annotations of variant consequence on the canonical transcript, we determined four variant categories for gene-based testing.

Variant consequence categories

(1) High confidence pLoF: high-confidence LoF variants as defined by LOFTEE[32] (LOFTEE HC).
(2) Damaging missense/protein-altering: at least one of the following:
  (a) Variant annotated as missense/start-loss/stop-loss/in-frame insertion or deletion (indel) and (REVEL ≥0.773 or CADD ≥28.1 or both).
  (b) Any variant with SpliceAI delta score (DS) ≥0.2 where SpliceAI DS is the maximum of the set {DS_AG, DS_AL, DS_DG, DS_DL} for each annotated variant (where DS_AG, DS_AL, DS_DG, and DS_DL are DS [acceptor gain], DS [acceptor loss], DS [donor gain], and DS [donor loss], respectively).
  (c) Low-confidence LoF variants as defined by LOFTEE (LOFTEE LC)
(3) Other missense/protein-altering: missense/start-loss/stop-loss/in-frame indel not categorized in (2).
(4) Synonymous: synonymous variants with SpliceAI DS <0.2 in the gene.

REVEL and CADD score cutoffs are chosen to reflect the supporting level for pathogenicity (PP3) from the American College of Medical Genetics and Genomics and the Association for Molecular Pathology (ACMG/AMP) criteria.[38] For our main analyses we only test pLoF and damaging missense variants. The two other variant consequence categories ("other missense/protein-altering" and "synonymous") are only used for our singleton burden analysis (Note S4).

Phenotype curation

Continuous phenotypes

We selected phenotypes among those with "standard polygenic risk scores (PRS)" available in the UKB.[25] Standing height (UKB field ID 50) and LDL-C (UKB field ID 30780), the two phenotypes analyzed by a previous study of phenotype misalignment,[26] were chosen to benchmark our approach.

The five other continuous traits (BMI [UKB field ID 21001], estimated BMD [UKB field ID 78], IOP [UKB field IDs 5262 and 5254], HbA1c [UKB field ID 30750], and AAM [UKB field ID 3581]) were prioritized for inclusion based on sample size in the UKB, heritability, how closely the trait available in the UKB matched the trait used to create the PGS, and whether there was prior evidence of monogenic disorders or genes with strong rare variant burden effect contributing to trait variability (Table 1). IOP was calculated as the mean between left (UKB field ID 5262) and right (UKB field ID 5254) corneal compensated measurements. We chose the corneal compensated measurement method instead of the Goldmann-correlated measurement method because it has been reported to be a better indicator for glaucomatous eyes.[39] For AAM, we excluded individuals who responded "do not know" or "prefer not to answer."

Table 1. Canonical genes associated with monogenic disorders for continuous phenotypes

Trait	Disorder	Effect direction of LoF	Genes
Standing height	growth disorder	negative (pathogenic)	*EZH2*, *FBN1*, *NSD1*
		positive (pathogenic)	*ACAN*, *FGFR3*, *IGF1*, *IGF1R*, *NPR2*, *SHOX*
LDL-C	familial hypercholesterolemia	negative (protective)	*APOB*, *PCSK9*
		positive (pathogenic)	*LDLR*
BMI	monogenic obesity	positive (pathogenic)	*LEP*, *LEPR*, *MC4R*, *PCSK1*, *POMC*
IOP	glaucoma 1, open angle, A	positive (pathogenic)	*MYOC*
BMD	osteogenesis imperfecta, type II	negative (pathogenic)	*COL1A1*, *COL1A2*
HbA1C	MODY	positive (pathogenic)	*GCK*, *HNF1A*
AAM	N/A	positive (protective)	*CHEK2*

Genes for each disorder are listed in alphabetical order. See methods for a description of how genes were selected. N/A, not applicable.

Dichotomous phenotypes

We included three dichotomous disease status phenotypes: T2D (International Classification of Diseases, 10th Revision [ICD-10] code E11), CAD (ICD-10 code I25.1), and OP (ICD-10 codes M80 and M81). Case status was defined by ICD-10 diagnoses (UKB field ID 41270). For each disease, individuals with the disease ICD-10 code(s) were defined as cases, while everyone lacking the disease ICD-10 code(s) but for whom UKB field ID 41270 was defined, were assigned control status.

PGSs

We used PGSs determined by Genomics PLC[25] and provided through the UKB (data field IDs in Table S1) for our analyses. We chose to use the "standard PRS" (UKB category 301) scores instead of the "enhanced PRS" (UKB category 302) because the standard PRS category of scores is trained solely using non-UKB data,[25] making it less prone to overfitting to the UKB, and as a result, due to their availability for more than three times as many UKB samples compared to the enhanced PRS.

Gene curation for monogenic and rare disorders

Curation of canonical genes for monogenic disorders

We defined a pilot set of "canonical" genes associated with monogenic disorders (hereafter referred to as canonical genes), where we believed we would be most likely to observe enrichment of deleterious variants in misaligned individuals. These are genes linked to some of the most common monogenic disorders associated with the phenotype being studied. Our curation process for each phenotype, informed by evidence from literature, was as follows.

For standing height and LDL-C, we selected the gene set associated with monogenic disorders provided in a study of misaligned individuals in a UKB subset[26] for both consistency and to check for recapitulation of associations (Table 1). For BMI, we selected the first five genes discovered to be associated with monogenic obesity[40]: *MC4R* (MIM: 155541), *PCSK1* (MIM: 162150), *LEP* (MIM: 164160), *POMC* (MIM: 176830), and *LEPR* (MIM: 601007). For BMD, we chose the two genes (*COL1A1* [MIM: 120150] and *COL1A2* [MIM: 120160]) where pathogenic variation is the most common cause of monogenic OP.[41] For IOP, we chose *MYOC* (MIM 601652), in which pathogenic variants are the most common single-gene cause of primary open-angle glaucoma via increased IOP.[42] For HbA1c, we chose the genes where pathogenic variation is commonly responsible for maturity-onset diabetes of the young (MODY), the most common monogenic form of diabetes.[43] Within the subtypes of MODY, *GCK*-MODY (MODY, type 2 [MIM: 125851]) and *HNF1A*-MODY (MODY, type 3 [MIM: 600496]), caused by pathogenic variants in *GCK* (MIM: 138079) and *HNF1A* (MIM: 142410), respectively, are the two most common subtypes, accounting for around 80% of MODY cases in the UK.[44,45] Patients with both subtypes can present with hyperglycemia,[43,46] which can be indicated by higher-than-normal HbA1c levels.[47] While there are other monogenic disorders that may have effects on HbA1c (e.g., anemia; congenital; nonspherocytic hemolytic, 1[48,49] [MIM: 300908]), we were primarily interested in using HbA1c as a liability trait for T2D and thus focused on genes with a glycemic association. For AAM, there are no common monogenic disorders. As a proxy, we selected *CHEK2* (MIM: 604373), the gene with highest strength of association in the burden test for pLoF variants for AAM in Genebass[28] (pLoF burden $p = 3.75 \times 10^{-32}$). Early menopause can be caused by primary ovarian insufficiency (POI), a partly monogenic disorder.[50] However, there are more than 100 genes putatively associated with monogenic POI, and in the UKB, most POI cases are not caused by monogenic mechanisms.[50] For T2D, we selected the four genes associated with the most common subtypes of MODY in the UK: *HNF1A*, *GCK*, *HNF4A* (MIM: 600281), and *HNF1B*[44] (MIM: 189907). We included *SLC30A8* (MIM: 611145) as a gene where LoF is implicated as being protective against T2D.[51] For CAD, we selected the same genes associated with FH (*PCSK9* [MIM: 607786], *APOB* [MIM: 107730], and *LDLR* [MIM: 606945]) as used for LDL-C because monogenic risk variants in these genes have been shown to interact with CAD polygenic risk to modulate overall disease risk.[13] We also included *ANGPTL3* (MIM: 604774), where LoF variants cause hypobetalipoproteinemia, familial, 2 (MIM: 605019) and are implicated as being protective against CAD.[52] Finally, for OP, we selected the same osteogenesis imperfecta, type II (MIM: 166210) genes as used for BMD (*COL1A1* and *COL1A2*).

Gene curation for rare disorders

To broaden the scan for enrichment to more genes and objectively define gene sets associated with rare disorders, we used the evidence-based, expertly curated, and frequently reviewed Genomics England (GEL) PanelApp resource.[27,53]

When selecting gene sets associated with rare disorders within the GEL PanelApp, we selected disorders that cause extreme, early-onset, or monogenic forms of the phenotypes in our analysis. We only included genes rated as "green"

(diagnosticgrade, conservatively defined) in the GEL PanelApp and excluded genes where the mode of pathogenicity was “other” or started with “loss-of-function variants (as defined in pop up message) DO NOT cause this phenotype.” We included genes with all models of inheritance, including both dominant and recessive inheritance models. This resulted in our final list of diagnostic-grade genes for each selected GEL rare disorder so that LoF variants in each gene were expected to cause the disorder.

Misaligned phenotype classification

Continuous traits

To determine individuals whose observed continuous phenotypes are aligned or misaligned with their genetically predicted phenotype, we followed Hawkes et al.[26] Briefly, covariates are residualized from the observed phenotype. Mahalanobis distance is calculated for each individual in the bivariate distribution formed by the residualized observed phenotype and the genetically predicted phenotype and converted to p values.[54] Residualized observed phenotypes are then regressed against genetically predicted phenotypes, and residuals are calculated for each individual. We then classify an individual as misaligned or aligned if they satisfied the following conditions.[26]

Misaligned individuals.
- Mahalanobis p value <0.001 and
- Absolute residual between residualized observed and expected phenotypes >2.

Aligned individuals.
- Absolute difference between residualized observed and expected phenotypes <1.

In the first residualization step, the covariates were *sex*, *age*, age^2, $sex \times age$, $sex \times age^2$, the first 21 principal components (PCs) (calculated on UKB genotype array data[55] and provided by the UKB), and UKB assessment center. Unlike Hawkes et al.,[26] we did not account for statins in our main results when adjusting LDL-C for covariates to maintain standardized covariates across all traits. This omission is conservative in that it is more likely to result in false negatives than false positives, as we expect cholesterol-lowering medication to attenuate the misalignment effects of deleterious variants associated with hypercholesterolemia. Indeed, in a case study of adjusting LDL-C for cholesterol-lowering medication, power for canonical gene enrichment appears to be improved (Note S3).

For misaligned phenotype classification in sex-specific analyses, stratification by sex occurred as the first step, before any residualization. All downstream steps were the same as in the sex-combined analysis, apart from the exclusion of any sex-related covariates (i.e., *sex* and any of its cross terms) when performing covariate residualization.

Case-control dichotomous traits

To contrast individuals with aligned versus misaligned disease status, we set percentile thresholds on covariate-residualized PRS distributions (Figure S9). Percentile thresholds were chosen to compare the top versus bottom halves (threshold: 50%), quartiles (thresholds: 25%, 75%), and deciles (thresholds: 10%, 90%). This followed the approach of Lu et al.,[11] who compared the top and bottom halves of the PRS distribution using point estimates for pathogenic variant prevalence. We included more extreme thresholds (quartiles, deciles) to assess whether enrichment increased in the extremes of the distribution.

Filtering to unrelated individuals

We excluded individuals with a high degree of relatedness (third degree or closer) when defining our aligned and misaligned individuals. Using relatedness estimates (G) from a genetic relatedness matrix (GRM) created by the scalable and accurate implementation of generalized mixed model (SAIGE)[56] from genotype array data, we filtered to a maximal unrelated set of individuals. We created a greedy algorithm that iterated through a list of individuals (sorted by the number of relatives, with fewest relatives first) with at least one relative ($G > 2^{-3.5}$; corresponding to relatives of the third degree or closer), adding their relatives to the list of individuals for removal. Individuals who had already been added to the list for removal were skipped if encountered later in the iteration. We performed this relatedness filter on each phenotype separately, pre-filtering to individuals with non-missing phenotype and covariate data before applying the relatedness filter, in order to maximize sample size for each phenotype.

Enrichment testing of variant presence and misaligned status

Exact tests on contingency tables

To test whether individuals misaligned with genetic expectation are enriched for a binary variable (e.g., variant presence—whether an individual carries one or more damaging variants in a gene), we used Fisher’s exact tests,[57] applied to 2×2 contingency tables.

Calculating empirical p values with simulated PGSs

Spurious misaligned enrichment is possible in our continuous trait misalignment testing framework, whether due to a phenotype effect that is stronger than the misaligned effect, a PGS that explains limited phenotype variability, or a combination of both (Figure S2). To avoid these possibilities, we calculate an empirical p value.

To establish empirical null distributions for enrichment test statistics, we tested the misalignment enrichment method using simulated PGSs. The simulated PGSs were generated independent of phenotypes, which means the expected phenotypic variance explained is zero. The PGSs were simulated from a multivariate standard normal distribution with zero covariance between individuals.

For each real (using non-simulated PGS) enrichment test in which individuals harbored deleterious variants in the gene(s) tested, we estimated the empirical null by generating replicates of enrichment test statistics based on simulated PGSs rather than real PGSs (Figure S2). This involved running the misalignment classification procedure using simulated PGSs instead of the real PGSs and then testing for enrichment of deleterious variants in the misaligned individuals. The empirical p value was calculated by the following equation:

$$P_{\text{emp}} = \frac{r + 1}{n + 1},$$

where n is the number of test statistic replicates drawn from the null, and r is the number of replicate test statistics that are greater than or equal to the observed test statistic.[58,59] We used $-\log_{10}$(one-sided Fisher’s exact test p) as our test statistic when calculating the empirical p value. We did not exclude replicates where misaligned individuals lacked the variants we were testing; instead, we assigned a Fisher’s exact test $p = 1$.

We simulated 10,000 replicates for all false discovery rate (FDR)-adjusted significant trait-gene-consequence combinations from enrichment tests of canonical and GEL PanelApp green

Table 2. REGENIE burden testing overview

Trait type	REGENIE used for misaligned phenotype testing in...	Variant consequence burden masks	Dependent variable in regression		Hypothesized direction of burden effect
Continuous	exome-wide scan	• pLoF<br>• damaging missense	misaligned status [encoding]	high misaligned [1] vs. aligned [0]	positive (OR > 1)
				low misaligned [1] vs. aligned [0]	positive (OR > 1)
Dichotomous (disease status)	• canonical gene enrichment<br>• GEL gene enrichment<br>• exome-wide scan	pLoF	PRS' (covariate-residualized PRS)	PRS' in cases	negative
				PRS' in controls	positive

Burden testing with REGENIE was performed for both continuous and dichotomous trait misalignment testing. In continuous traits, it was only used for the exome-wide scan, while in dichotomous traits, it was used for all three gene set categories. We tested both pLoF and putatively damaging missense variants for continuous traits but only pLoF for dichotomous traits. Misalignment testing in continuous traits treated the misaligned status as a dichotomous variable, with misaligned individuals always encoded as a 1 and aligned as 0. Misalignment testing in dichotomous traits was performed on covariate-residualized PRSs, a continuous variable. We use one-sided hypothesis tests throughout. For continuous traits, we always test for higher burden in misaligned individuals; for dichotomous traits, we test for a negative burden association in cases and a positive burden association in controls.

genes. For significant genes resulting from the exome-wide scan, we simulated 1,000 replicates, using this reduced number of replicates due to the high computational and financial cost when running replicates on the DNAnexus Research Analysis Platform (RAP) for a large number of significant genes. The main downside of only using 1,000 replicates for the significant genes from the exome-wide scan instead of 10,000 is that the empirical p value can be no less than $\frac{1}{1{,}000+1}$ (9.99×10^{-4}) rather than $\frac{1}{10{,}000+1}$ (1.00×10^{-4}).

Hypergeometric test for the probability of observing no variant presence

When testing misaligned individuals for enrichment of rare (MAF <0.1%) pLoF variants in genes associated with monogenic disorders, there were many instances where, due to the rarity of being misaligned compounded with the rarity of harboring rare pLoF variants, there were no misaligned individuals with rare pLoF variants. To quantify the high likelihood of observing no overlap in misaligned status and harboring a pLoF variant, we used the hypergeometric distribution to calculate the probability of observing no variant presence ($k = 0$) in a sample of n individuals (misaligned individuals) from a larger group of N individuals (aligned and misaligned individuals) with K individuals harboring a pLoF variant:

$$P(k = 0; n, N, K) = \frac{\binom{K}{0}\binom{N-K}{n}}{\binom{N}{n}} = \frac{\binom{N-K}{n}}{\binom{N}{n}}.$$

Burden testing on phenotype misalignment

We used REGENIE version 4.1[60] to perform gene-level rare-variant (MAF < 0.1%) burden testing of phenotype misalignment for continuous (for exome-wide scan) and dichotomous traits (for canonical and GEL PanelApp green gene enrichment tests and exome-wide scan), using individuals with European genetic ancestry in the ES 450K tranche of the UKB (Table 2).

In REGENIE step 1, we fit the null model using UKB genotype array data QCed with PLINK[61] (filtering flags: `–maf 0.01 –mac 100 –geno 0.1 –mind 0.1`). We included sequencing tranche, the tranche in which an individual was exome sequenced, as the only covariate. All other standard covariates (e.g., age, sex, PCs) had already been regressed out of the observed phenotype (continuous traits) or out of the PRS (dichotomous traits).

After fitting the null model in step 1, we carried out gene-level burden testing in REGENIE step 2 for pLoF (continuous and dichotomous traits) and damaging missense (continuous traits only) variants with MAF < 0.1% using the UKB ES 450K tranche data.

To test for an association between rare deleterious variation and misaligned status in continuous traits, we treated misaligned status as a dichotomous phenotype (using the `--bt` flag in REGENIE). We tested high misaligned vs. aligned and low misaligned vs. aligned separately. When testing misalignment in disease status, we did not apply inverse rank-normal transformation (IRNT) to residualized PRSs within REGENIE because the PRSs were already normalized.

To compare the statistical power in testing misaligned individuals for gene enrichment to traditional approaches, we ran gene burden tests on the original continuous and dichotomous phenotypes used as input to the misaligned phenotype classification framework. We applied IRNT within REGENIE to all continuous phenotypes using the `--apply-rint` flag and used the `--bt` flag for dichotomous traits.

For sex-specific analyses of canonical and GEL PanelApp green genes in dichotomous traits, we performed linear regression using statsmodels 0.14.6 in Python to test the association of pLoF burden association with PRSs, using the same regression model we had applied with REGENIE.

Conditioning on common imputed variants when testing misaligned disease status

When testing rare-variant burden effects on common-variant PRSs for misaligned disease status, there is a possibility that PRSs could correlate with rare variants at a given gene due to linkage disequilibrium (LD). This would lead to spurious burden associations. To conditionally orthogonalize the PRS from rare-variant burden, we conditioned on common (MAF > 0.1%) imputed v3 variants located in or within a 1 Mb radius of gene(s) tested (LD in Europeans tends not to extend beyond 1 Mb[30]), with gene start and stop locations in GRCh37 coordinates to match the imputed v3 variants. We used PLINK to QC these variants, following similar filtering thresholds used to select variants that generated the PRSs[25] (PLINK filtering flags: `–maf 0.001 –hwe`

1e-10, variants with INFO score < 0.8 excluded). We conditioned on these QCed variants in REGENIE step 2 by passing a gene-specific (or grouped-gene) PLINK pgen file with the flag -condition-file. In our exome-wide scan for misaligned disease status, we performed this conditioning on FDR-significant genes to check the robustness of our results.

Defining significant associations

For each stage of enrichment tests, we applied the Benjamini-Hochberg (BH) procedure to all associations, adjusting for FDR $\leq$ 5%. Statistically significant associations were defined as having FDR-adjusted $p < 0.05$. As our gene sets (canonical genes, GEL PanelApp green gene panels, exome-wide scan) overlapped, we excluded previously tested genes from consideration when calculating FDR-adjusted p values. After FDR correction, we filtered results on gene-level allele counts to avoid spurious associations in genes with low allele counts. For continuous trait misaligned status testing, only genes with minor-allele count (MAC) ≥ 5 in the misaligned group being tested were retained. For dichotomous trait misaligned disease status testing, only genes with alternate allele count (AAC) ≥ 10 were retained.

Ethical approval

This study used data from the UKB. The North West Multi-centre Research Ethics Committee (MREC) gave approval to UKB as a research tissue bank (RTB) approval. This approval means that researchers do not require separate ethical clearance and can operate under the RTB approval.

Results

Identifying individuals with misaligned continuous phenotypes

Individuals were classified as deviating from their genetically expected phenotype if their observed phenotype, adjusted for covariates, was a significant outlier in the bivariate distribution of phenotype and PGS and if the covariate-residualized observed phenotype did not align with a linear model for observed phenotype and PGS (methods). We applied this method to seven continuous traits in the UKB, with the percentage of misaligned individuals ranging from 0.07% (HbA1c) to 0.12% (AAM) (Figure 1; Table S1; Figure S1).

Individuals with misaligned continuous phenotypes are enriched for predicted LoF variants in canonical genes associated with monogenic disorders

Following categorization into aligned and misaligned individuals, misaligned individuals can be further split into those whose covariate-residualized observed phenotype is higher than expected ("high misaligned") and lower than expected ("low misaligned") relative to their PGS. In our enrichment tests, we tested for enrichment in one of the misaligned groups (either high or low misaligned) against the aligned group, with the aligned group acting as a control group (methods; Figure 1).

We are interested in finding genes that significantly contribute to phenotype misalignment. Due to the sparsity of individuals harboring rare deleterious variants, individuals with misaligned status, and the overlap of these two groups, we wish to control the possibility of spurious associations. Misaligned individuals also tend to have extreme covariate-residualized phenotypes. As the canonical and GEL PanelApp green genes we test are known to associate with the traits tested, we expect individuals harboring rare deleterious variants in these genes to cluster among individuals with extreme covariate-residualized phenotypes (high or low extremes depending on the effect direction of variants in the gene). Thus, we want to be sure that the misaligned associations we observe are distinct from extreme phenotype associations. Related to this, if a PGS explains limited phenotypic variability, then the slope of the phenotype-PGS regression will be close to zero. This would result in phenotype-PGS residuals that directly correspond to the covariate-residualized phenotype. Thus, the misalignment enrichment test would reduce to a test on the phenotype, an issue similar to the overlap between misaligned individuals and individuals with extreme covariate-residualized phenotypes.

To increase confidence that any continuous trait misalignment enrichment is distinct from extreme phenotype association and that the PGS explains a sufficient amount of phenotypic variability, we calculated an empirical p value for each putatively significant enrichment result by comparing the observed test statistic (one-sided Fisher's exact test p value) against an empirical null distribution of test statistics (methods; Figure S2).

We tested misaligned individuals for enrichment of rare (MAF < 0.1%) pLoF variants (see methods for variant consequence definitions) in genes associated with monogenic disorders relevant to the misaligned phenotype (methods).

Height and LDL-C

Grouping variants across canonical genes associated with monogenic stature disorders (Table 1), we observed significant enrichment (FDR-adjusted one-sided Fisher's exact test $p_{adj} \leq 0.05$) of pLoF variants in individuals with shorter-than-expected stature (odds ratio (OR) [90% confidence interval (CI)] = 85.7 [49.4–149], $p_{adj} = 1.13 \times 10^{-15}$) (Figure 2; Table S2). We also tested single genes and observed significant enrichment in individuals with shorter-than-expected stature for *ACAN* (OR [90% CI] = 367 [188–717], $p_{adj} = 3.02 \times 10^{-17}$; MIM: 155760), *IGF1* (OR [90% CI] = 1,280 [125–13,200], $p_{adj} = 4.08 \times 10^{-3}$; MIM: 147440), and *SHOX* (OR [90% CI] = 183 [31.4–1,070], $p_{adj} = 0.0148$; MIM: 312865) (Figure 2; Table S2).

When testing a set of genes associated with FH (Table 1), we found that individuals with high or low LDL-C were enriched for pLoF variants in genes where LoF is known to cause high (*LDLR* OR [90% CI] = 213 [96.8–468], $p_{adj} = 3.31 \times 10^{-10}$) or low LDL-C (*APOB* OR [90% CI] = 153 [106–220], $p_{adj} = 9.45 \times 10^{-45}$; *PCSK9* OR [90% CI] = 45.0 [24.6–82.4], $p_{adj} = 1.29 \times 10^{-10}$), respectively (Figure 2; Table S2). Individuals with lower than

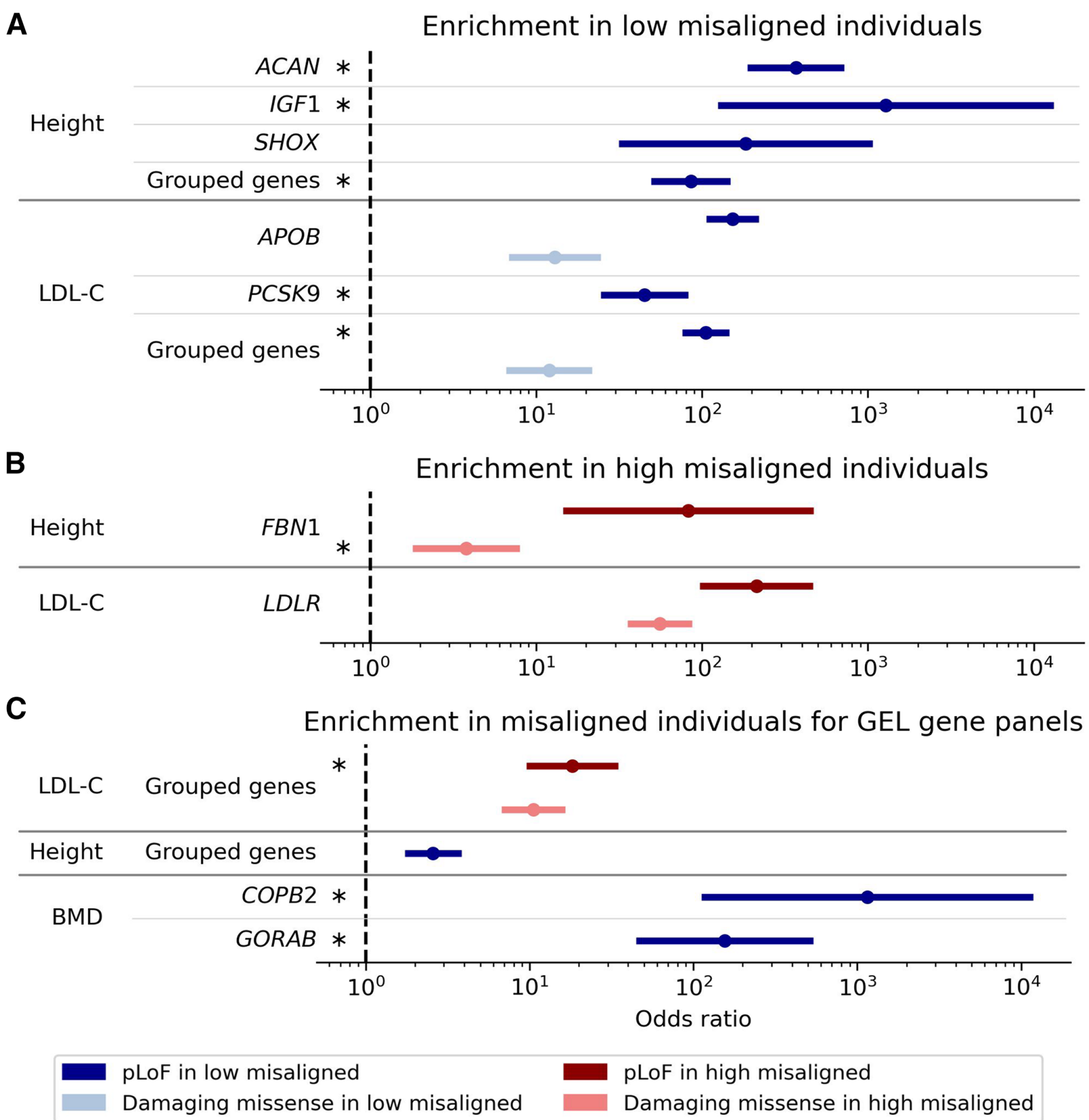

Figure 2. Enrichment of pLoF and damaging missense variants in canonical and GEL PanelApp green genes
Shown is enrichment in (A) low and (B) high misaligned individuals for canonical genes associated with monogenic disorders and (C) misaligned individuals for GEL gene panels. "Low misaligned" individuals are individuals with phenotypes lower than genetically expected by PGS. "High misaligned" individuals are individuals with phenotypes higher than genetically expected by PGS. Error bars indicate two-sided 90% CI for OR so that the lower bound indicates the lower bound for a one-sided 95% CI. Only significant enrichment results (FDR $\leq$ 5%) are shown. Asterisks indicate results where the empirical p value is significant ($p_{emp} < 0.05$). Empirical null distributions can be seen in Figure S5.

genetically expected LDL-C were also enriched for harboring the combined burden of *PCSK9* and *APOB* pLoF variants (OR [90% CI] = 105 [75.9–146], $p_{adj} = 5.86 \times 10^{-51}$) (Figure 2; Table S2).

Sex-specific analyses recapitulated at nominal significance ($p < 0.05$) all sex-combined associations for height and LDL-C (Figure S6; Table S2).

Additional continuous phenotypes

For other continuous phenotypes, no misaligned individuals harbored pLoF variants in canonical genes associated with monogenic disorders (Figure S3; Table 1), likely due to the extremely low frequency of these variants in a population cohort such as the UKB. In each trait-gene pair, there was a relatively high probability (minimum of hypergeometric test $P(k = 0) = 0.83$; methods) of observing zero pLoF variants in the set of misaligned individuals, given the number of individuals harboring pLoF variants in the group of misaligned and aligned individuals tested.

Missense variants that are deleterious but not predicted to result in LoF ("damaging missense" variants; methods) are by definition expected to have less of an effect on gene product than pLoFs. Assuming a causal link between gene product and phenotype, this implies that damaging missense variants are also expected to have attenuated effect sizes on the phenotype compared to pLoF variants. Although effect size is generally attenuated for damaging missense variants, they tend to occur at higher frequencies. For example, the aggregated pLoF allele count across genes tested for enrichment was 3,114; for damaging missense alleles, the count was 17,984, almost 6-fold higher. We hypothesized that testing damaging missense variants instead might boost power in enrichment testing by increasing the number of individuals harboring putatively damaging variation, albeit at smaller effect size, among misaligned individuals.

Individuals with misaligned continuous phenotypes are enriched for predicted damaging missense variants in genes associated with monogenic disorders

We tested for enrichment of damaging missense variants in misaligned individuals with the same methods as for pLoF variants, for the same set of continuous traits and corresponding genes associated with monogenic disorders.

We observed a significant enrichment (Fisher's exact test FDR-adjusted $p_{adj} \leq 0.05$) of damaging missense variants for four of the nine genes/gene sets where misaligned individuals were enriched for pLoF burden (Figure 2; Table S2). Individuals with higher than genetically expected height were enriched for damaging missense variants in *FBN1* (OR [90% CI] = 3.79 [1.80–7.97], $p_{adj} = 0.0254$; MIM: 134797). For LDL-C, high misaligned individuals were enriched for damaging missense variants in *LDLR* (OR [90% CI] = 55.7 [35.5–87.4], $p_{adj} = 3.09 \times 10^{-21}$), while low misaligned individuals were enriched for damaging missense variants in *APOB* (OR [90% CI] = 13.0 [6.87–24.6], $p_{adj} = 5.64 \times 10^{-6}$) and grouped *APOB* and *PCSK9* variants (OR [90% CI] = 12.0 [6.59–21.8], $p_{adj} = 2.18 \times 10^{-8}$).

All sex-combined FDR-significant results were recapitulated at nominal significance ($p < 0.05$) by at least one sex-specific analysis (Figure S6; Table S3). We observed one nominally significant single-gene result unique to the sex-specific analysis: enrichment of damaging missense variants in *POMC* (MIM: 176830) among males with higher than genetically expected BMI (OR [90% CI] = 17.3 [5.25–56.9], $p = 6.62 \times 10^{-3}$).

As expected, given that we are testing damaging missense variants, which are predicted (methods) to have a less deleterious effect than pLoF variants, the enrichment of damaging missense variants consistently had lower OR point estimates than pLoF variants (4/4 single- and grouped-gene tests with FDR $\leq$5% significant pLoF and damaging missense enrichment; Figure 2).

Individuals whose continuous phenotype is misaligned with genetic expectation are enriched for predicted damaging variants in genes related to rare disorders

Having demonstrated that misaligned individuals are enriched for both pLoF and damaging missense variation in canonical genes associated with monogenic disorders for continuous traits, we expanded the set of genes analyzed to include genes associated with other rare disorders related to the traits analyzed. To do this, we extracted genes from the expertly reviewed GEL PanelApp gene sets[27,53] for rare disorders relevant to the phenotypes included in our analysis (methods). We restricted analysis to genes that qualified for the highest-confidence category (green), designated to be of diagnostic grade.[27,53]

We tested all continuous traits for enrichment of pLoF and damaging missense variants in GEL PanelApp green genes in misaligned individuals. We used the same enrichment testing framework as described above for the canonical genes associated with monogenic disorders. We excluded 7.69% (38/494) of single- and grouped-gene masks because they had already been included in the enrichment testing of canonical genes. We performed the BH procedure[62] to control FDR at 5% across all pLoF and damaging missense enrichment tests for continuous traits.

There were four single- and grouped-gene masks across three traits (height, LDL-C, and BMD) for which misaligned individuals were significantly enriched (Fisher's exact test FDR-adjusted $p_{\text{adj}} \leq 0.05$) for pLoF or damaging missense variants (Figure 2; Figure S4; Table S2).

Individuals with lower than genetically expected BMD were enriched for pLoF variants in *COPB2* (OR [90% CI] = 1,150 [112–11,900], $p_{\text{adj}} = 0.0325$; MIM: 606990) and *GORAB* (OR [90% CI] = 155 [44.5–539], $p_{\text{adj}} = 3.17 \times 10^{-3}$; MIM: 607983), genes selected from the osteogenesis imperfecta gene panel in the GEL PanelApp (Figure 2; Table S2). Although the presence of pLoF variants in the low misaligned individuals was extremely sparse (*COPB2* $n = 1$, *GORAB* $n = 2$), both of these genes demonstrated significant empirical p values ($p_{\text{emp}} < 0.05$) (Figure S5), reinforcing the possibility of a true misalignment effect. The enrichment in *GORAB* was also supported by nominally significant ($p < 0.05$) enrichment in a male-only analysis (OR [90% CI] = 107 [18.8–609], $p = 0.0104$) but was also driven by only one individual harboring a pLoF variant in the misaligned group.

Individuals with higher than genetically expected LDL-C and individuals with lower-than-expected height were also enriched for deleterious variant burden in grouped GEL PanelApp genes. However, these significant grouped results were driven largely by variants in the previously tested canonical genes, with attenuated ORs compared to the results of the grouped canonical genes (Figure 2; Table S2).

Association of non-genetic factors with continuous trait misalignment

Although the primary focus of our work is identifying genes associated with phenotype misalignment, we tested whether misaligned status was associated with non-genetic factors, such as socioeconomic status (SES), medication, smoking status, and exercise (Note S1; Figure S7; Table S4). We observed nominal association (one-sided Wald test $p < 0.05$) of SES with pathogenic misalignment (defined as trait increasing, except for BMD and AAM, where it is trait decreasing) for each continuous trait. We also observed a strong enrichment of cholesterol-lowering medication in individuals with lower than genetically expected LDL-C (OR [90% CI] = 11.7 [8.98–15.2], one-sided Fisher's exact test $p = 1.21 \times 10^{-53}$). These results indicate that such non-genetic factors may also play a role in misalignment, as shown in previous work.[26]

Individuals whose disease status is misaligned with genetically expected risk are enriched for predicted LoF variants in genes associated with monogenic disorders

We examined misaligned disease status relative to PRS in three common complex diseases: T2D, CAD, and OP. These diseases have been included in previous UKB studies involving PRS and penetrance of pathogenic variants.[11,13] We tested the hypotheses that (1) disease cases with low PRS are enriched for pathogenic rare pLoF variants and (2) disease controls with high PRS are enriched for protective rare pLoF variants.

For each hypothesis, we took a two-step approach. First, within a given disease status group (cases or controls), we used linear regression to test the association of PRS with (pathogenic or protective) pLoF variant presence. Variant presence is a binary variable, defined as whether an individual carries at least one pLoF in the gene(s) tested. Second, similar to earlier work in a smaller subset of the UKB,[11] we defined low and high PRS percentile groups and tested enrichment of pLoFs between the groups.

To validate our approach, we first applied it to binarized versions of height and LDL-C, continuous traits for which we have already observed an enrichment of rare deleterious variants in individuals whose phenotypes deviate

Table 3. **Canonical genes associated with monogenic disorders tested for pLoF enrichment in disease traits**

Disease	Associated disorder	Effect of LoF	Genes
T2D	MODY[63]	pathogenic	*GCK*, *HNF1A*, *HNF4A*, *HNF1B*
	N/A	protective[51]	*SLC30A8*
CAD	familial hypercholesterolemia hypobetalipoproteinemia, familial, 2[52]	protective[64–66]	*APOB*, *PCSK9*
		pathogenic[67]	*LDLR*
		protective	*ANGPTL3*
OP	osteogenesis imperfecta, type II[68]	pathogenic	*COL1A1*, *COL1A2*

N/A, not applicable.

from genetic expectation. Among simulated dichotomized traits with a case prevalence of 5% and 10% (similar to the common diseases we aim to study), we replicated all but one (8/9) FDR-significant single- and grouped-gene pLoF associations for misaligned height and LDL-C at nominal significance (one-sided t test $p < 0.05$) (Figure S8; Table S5). This demonstrated a proof of concept that our pLoF enrichment testing approach for dichotomous case-control traits could recapitulate similar pLoF enrichment in misaligned individuals observed for continuous traits.

Disease cases with low polygenic risk are enriched for rare pathogenic predicted LoF variants in genes associated with monogenic disorders

Among T2D, CAD, and OP cases, we tested for association of rare (MAF < 0.1%) pathogenic pLoF burden with lower PRS. We also tested for association of pLoF burden in low-PRS cases versus high-PRS cases. As with the enrichment testing of misaligned continuous traits, we used a curated set of canonical genes associated with monogenic disorders (Table 3; methods). Our burden tests were conditioned on common imputed variants in or within 1 Mb of each gene being tested (methods). We performed the BH[62] procedure to control FDR at 5% separately for the regression association tests and Fisher's exact tests.

In T2D cases, pLoF burden was significantly associated (FDR-adjusted one-sided burden $p_{adj} \leq 0.05$) with lower PRS for burden in *HNF1A* (β [90% CI] = -0.788 [-1.09, -0.484], $p = 1.03 \times 10^{-5}$, $p_{adj} = 1.44 \times 10^{-4}$) and *HNF4A* ($\beta$ [90% CI] = -1.88 [-3.08, -0.685], $p = 4.83 \times 10^{-3}$, $p_{adj} = 0.0338$), with nominally significant (one-sided $p < 0.05$) burden associations in *GCK* and grouped genes (Figure 3A; Table S6). The *HNF1A* result was supported by nominally significant ($p < 0.05$) male- and female-specific analyses, while the *HNF4A* result was driven solely by two female individuals harboring pLoF variants (β [90% CI] = -2.22 [-3.30, -1.15], $p = 3.24 \times 10^{-4}$) (Figure S10; Table S8).

When testing pLoF enrichment in low- vs. high-PRS T2D cases for these single- or grouped-gene masks, we

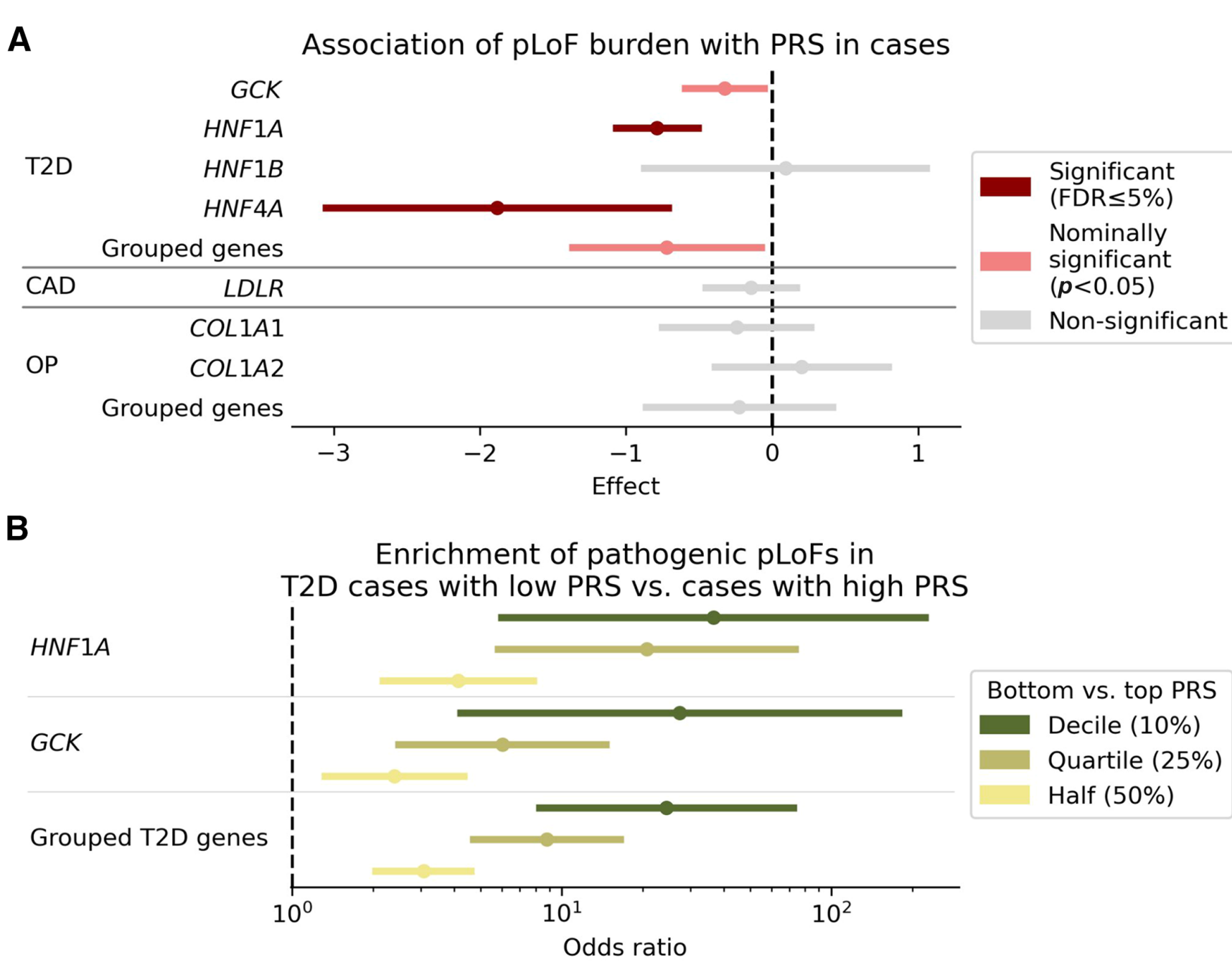

Figure 3. Pathogenic pLoFs are associated with lower PRS in disease cases
(A) Effect of pLoF variants on PRS among disease cases. Association tests were conditioned on common (MAF > 0.1%) imputed variants in or within 1 Mb of each gene tested. Significant associations are defined by FDR-adjusted one-sided burden $p_{adj} \leq 0.05$. Nominally significant associations are defined by one-sided burden $p < 0.05$.
(B) Comparing pathogenic pLoF enrichment in extremes of PRS for disease cases, for genes with significant enrichment results in (A) except for *HNF4A*, which had insufficient individuals harboring pLoF variants to perform enrichment testing in extremes. Error bars indicate two-sided 90% CI for OR so that the upper bound in (A) or lower bound in (B) indicates the upper or lower bound, respectively, for a one-sided 95% CI. pLoF, predicted loss of function; PRS, polygenic risk score; CAD, coronary artery disease; OP, osteoporosis; T2D, type 2 diabetes.

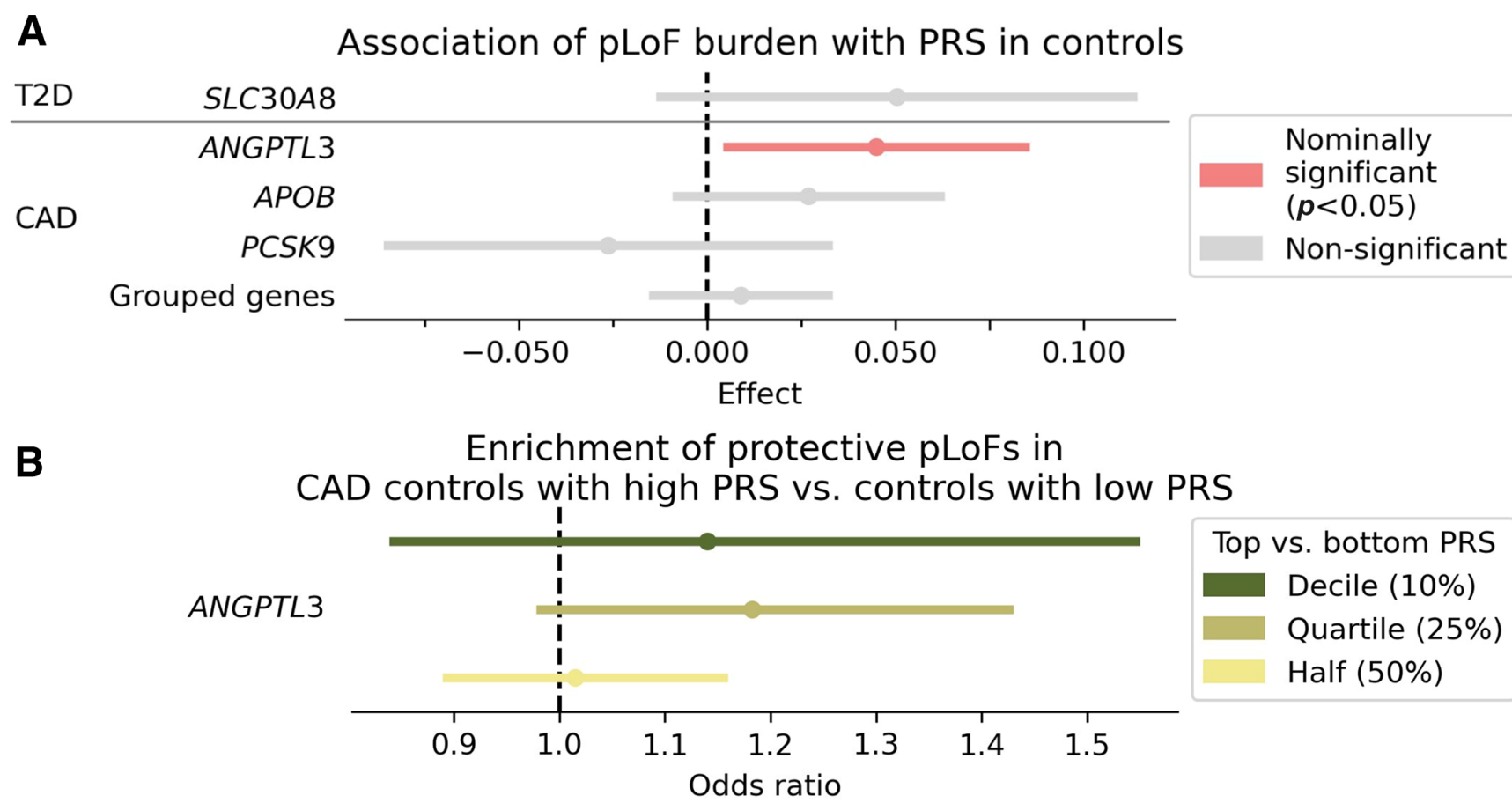

Figure 4. Protective pLoFs are associated with higher PRS in disease controls

(A) Effect of pLoF variants on PRS among disease controls. Association tests were conditioned on common (MAF > 0.1%) imputed variants in or within 1 Mb of each gene tested. Nominally significant associations are indicated by one-sided burden $p < 0.05$.

(B) Comparing pathogenic pLoF enrichment in extremes of PRS for disease controls for genes with significant enrichment results in (A). Error bars indicate two-sided 90% CI for OR so that the lower bound indicates the lower bound for a one-sided 95% CI.

observed a monotonic trend in increasing OR point estimates when comparing ever more extreme PRS quantiles (Figure 3B; Table S7). This suggests that enrichment of pLoFs increases as we compare individuals increasingly misaligned with genetic expectation (low-PRS cases) to individuals increasingly aligned with genetic expectation (high-PRS cases).

Disease controls with high polygenic risk are enriched for rare protective predicted LoF variants in genes associated with monogenic disorders

We tested for enrichment of rare pLoF variants in *APOB*, *PCSK9*, and *ANGPTL3* for CAD controls and *SLC30A8* for T2D controls; these are genes where LoF is expected to be protective for the corresponding disease (Table 3; Figure 4A). Among CAD controls, we observed nominally significant (one-sided burden $p < 0.05$) association of pLoF burden in *ANGPTL3* with increased CAD PRS (β [90% CI] = 0.0449 [4.22×10^{-3}, 0.0856], $p = 0.0347$, $p_{adj} = 0.108$). Sex-stratified analyses suggested that the *ANGPTL3* burden association is primarily driven by males (β [90% CI] = 0.145 [0.0500, 0.239], $p = 5.97 \times 10^{-3}$) rather than females (β [90% CI] = -3.19×10^{-3} [−0.0852, 0.0788], $p = 0.526$; Figure S10; Table S8). Comparing relative enrichment of pLoF variant presence in disease controls with high versus low PRS, OR point estimates did not demonstrate the same monotonicity as observed for pathogenic pLoFs in disease cases (Figure 4B; Table S7).

Limited evidence for enrichment of predicted LoF variants in GEL PanelApp green genes for disease cases with misaligned disease status

We extended our analysis in dichotomous trait misalignment from canonical genes to GEL PanelApp gene panels. There were no GEL PanelApp green genes where pLoF burden associated significantly (FDR-adjusted one-sided burden $p_{adj} \leq 0.05$) with lower PRS in cases (Table S6). There were three genes with nominal associations: *BMP1* (MIM: 112264) for OP (β [90% CI] = −0.618 [−1.18, −0.0573], $p = 0.0349$), *LMNA* (MIM: 150330) for T2D (β [90% CI] = −0.567 [−1.12, −0.0117], $p = 0.0465$), and *LIPA* (MIM: 613497) for CAD (β [90% CI] = −0.479 [−0.956, -2.70×10^{-3}], $p = 0.0490$).

Association of non-genetic factors with misalignment in disease status

As performed for the continuous traits, we also tested dichotomous trait misalignment for association with non-genetic factors (Note S1; Figure S7; Table S4). We observed a strong protective misaligned association (one-sided t test $p < 2 \times 10^{-61}$) for self-reported disease-specific medication across all three diseases. We also observed that T2D pathogenic and protective misalignments are associated with smoking status ($p = 2.12 \times 10^{-4}$) and exercise ($p = 3.80 \times 10^{-7}$), respectively (Note S1; Figure S7; Table S4).

Exome-wide scan for genes associated with misaligned status in continuous traits

Hypothesizing that there may be genes associated with misaligned status that were not included in the canonical or GEL PanelApp green gene sets, we scanned all genes for deleterious missense variation associated with misaligned status. Genes where deleterious missense variation causes individuals to have lower than genetically expected disease risk or lower-than-expected liability phenotype would be ideal targets for therapeutic deactivation. Conversely, a gene where missense variation is pathogenic could be therapeutically targeted for increased expression, assuming druggability of the gene.

For misaligned status of each continuous trait, we performed gene burden testing of rare (MAF < 0.1%) pLoF and damaging missense variants with REGENIE,[60] testing separately for protective and pathogenic effects on phenotype misalignment (methods). Protective effects were defined as those that are trait decreasing, except for BMD and AAM, where protective effects are trait increasing. Pathogenic effects are defined as trait increasing, except for BMD and AAM, where they are trait-decreasing. For continuous phenotypes, these definitions are oriented by the diseases of interest for which the continuous phenotypes act as liability phenotypes. The exception was height, where protective effects were

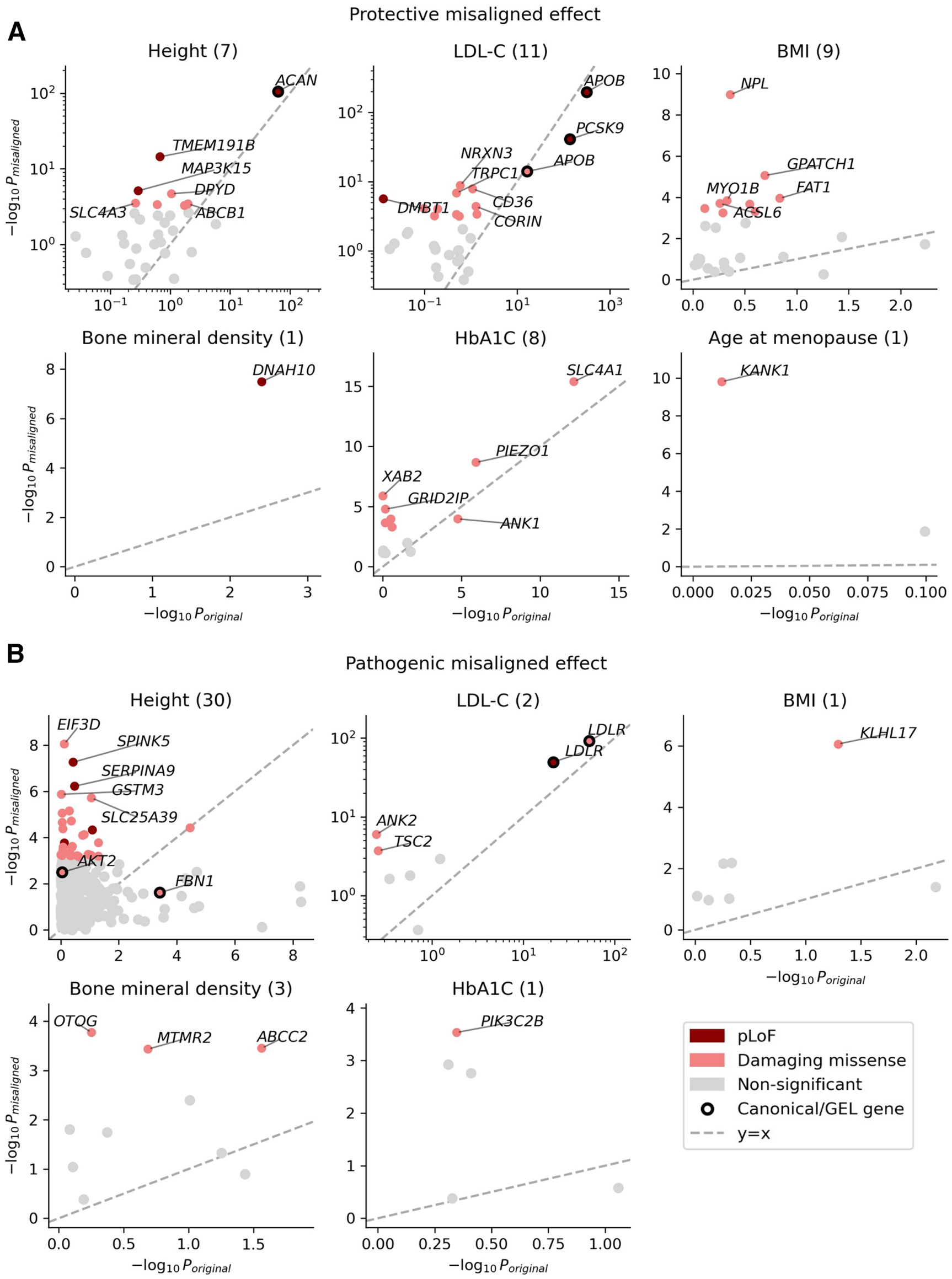

Figure 5. Strength of misaligned burden association, comparing burden test on original phenotype versus burden test of misaligned status

(A) Protective misaligned effect.

(B) Pathogenic misaligned effect. $p_{misaligned}$ is a one-sided p value, with the alternate hypothesis that burden of damaging variants is higher in misaligned individuals (cases) than aligned individuals (controls). $p_{original}$ is a two-sided p value for the burden association with the original continuous trait. Only genes with $MAC_{case} \geq 5$ are shown. The top 5 FDR-significant genes with highest $-\log_{10} p_{misaligned}$ are labeled with a gene symbol. Canonical and GEL PanelApp green genes are also labeled. The number of FDR-significant genes for each trait is indicated in parentheses in each subplot title. Protective misaligned status corresponds to low misaligned status and pathogenic misaligned status to high misaligned status, except for BMD and AAM, where the effects are flipped (protective = high misaligned, pathogenic = low misaligned).

defined to be trait decreasing and pathogenic effects trait increasing. These directions were arbitrarily chosen, as there exist both disorders of short and tall stature. When testing misaligned status for protective or pathogenic effects, we always test for enrichment in the misaligned group corresponding to the effect on the original trait (lower-than-expected misaligned when effect is negative, higher-than-expected misaligned when effect is positive).

Burden test p values were controlled using the BH procedure[62] for FDR $\leq$ 5% across all phenotypes and all genes, separately for protective and pathogenic misalignment tests, with previously tested canonical monogenic disorder and GEL PanelApp green genes excluded. Following FDR control, genes were filtered to those with MAC $\geq$ 5 in the misaligned group being tested to limit spurious associations. An empirical null p value was calculated for each significant gene enrichment result (Figure S12; methods).

Protective effect enrichment tests

In our exome-wide burden tests for "protective" misaligned status (lower-than-expected misalignment, except for BMD and AAM, for which protective misaligned status is higher-than-expected misalignment), 37 genes across six traits harbored significant enrichment (FDR-adjusted one-sided burden $p_{adj} \leq 0.05$) of pLoF or damaging missense variants (Figure 5A; Table S9).

For lower-than-expected BMI misalignment (9 significant genes), damaging missense associations with plausible biological mechanisms included two genes with links to fat metabolism: *ACSL6* (burden $p_{misaligned} = 1.99 \times 10^{-4}$, $p_{adj} = 0.0182$, $p_{emp} = 0.106$; Figure 5A; Table S9; MIM: 604443), involved in lipid synthesis, so that knockdown in rat skeletal muscle results in decreased lipid accumulation,[69] and *FAT1* (burden $p_{misaligned} = 1.12 \times 10^{-4}$, $p_{adj} = 0.0116$, $p_{emp} = 0.0260$; MIM: 600976), involved in fatty acid conversion.[70] The gene with the strongest association, *NPL* (burden $p_{misaligned} = 1.06 \times 10^{-9}$, $p_{adj} = 6.94 \times 10^{-7}$, $p_{emp} = 9.99 \times 10^{-4}$; MIM: 611412), has prior experimental evidence supporting a plausible link with decreased BMI. Deficiency of NPL has been shown to result in loss of skeletal muscle in humans, zebrafish, and mice.[71] This loss of muscle mass could lead to lower-than-expected BMI. All three plausible genes have LoF observed/expected upper bound fraction (LOEUF) scores above the Genome Aggregation Database (gnomAD)[32] v.4.1.1 heuristic threshold of 0.45,

which suggests that they are likely to be tolerant to LoF. Likewise, none of these three genes are directly implicated in severe disorders in Online Mendelian Inheritance in Man (OMIM) (https://omim.org/). Both of these lines of evidence point to the possibility of therapeutic gene perturbation without lethal consequences. Of the three genes, *ACSL6* is the only one with evidence of knockdown directly influencing lipid accumulation, a mechanism central to obesity. Investigating its therapeutic tractability in Open Targets,[72] it is predicted to have a signal peptide or transmembrane regions, which makes it more amenable to antibody-based therapeutics.

Individuals with higher-than-expected AAM were enriched for damaging missense variants in *KANK1* (burden $p_{\text{misaligned}} = 1.55 \times 10^{-10}$, $p_{\text{adj}} = 1.27 \times 10^{-7}$, $p_{\text{emp}} = 3.00 \times 10^{-3}$; Figure 5A; Table S9; MIM: 607704). When assessing the original trait, association of damaging missense burden in *KANK1* with AAM was null in our study (two-sided burden $p = 0.964$) and in Genebass[28] (missense|-low-confidence LoF (LC) burden $p = 0.879$). *KANK1* is associated with uterine fibroid risk,[73] much like *CHEK2*, a gene associated with tumor suppression,[74] where LoF has previously been shown to be associated with increased AAM.[75] Uterine fibroids become less common after menopause,[76] so if damaging variation in a gene was protective for POI, increasing AAM, then it might extend the window of time for uterine fibroids to appear, increasing the odds of developing uterine fibroids. *KANK1* is also implicated in the Reactome[77] pathway "estrogen-dependent gene expression," with participation in the reaction "estrogen-responsive *CXXC5* gene expression." There is a strong link between menopause and estrogen,[78] with estrogen levels typically declining due to menopause. If *KANK1* were to be causally implicated in menopause, then it may also be implicated in the decline of estrogen associated with menopause. Regarding its viability as a therapeutic target, *KANK1* is not under strong selective constraint (LOEUF = 1.2; gnomAD[32] v.4.1.1), which suggests that it may be perturbable without patient lethality. Although heterozygous full-gene deletion of *KANK1* has been linked to congenital cerebral palsy,[79] this was a case study of a single family, and only individuals who inherited the full-gene deletion from their parents were affected. There is no evidence that damaging missense variants would have such an effect. Furthermore, therapeutic tractability evidence collated by Open Targets suggests that *KANK1* may be an accessible target for antibody-based therapies; three collated sources (UniProt, Gene Ontology, and the Human Protein Atlas) predict with high confidence that the gene location is at the plasma membrane or extracellular region or is secreted by the cell.

For lower-than-expected HbA1c misalignment (8 significant genes), we replicated several known HbA1c associations, including *PIEZO1*[80] (MIM: 611184), and two genes (*SLC4A1* [MIM: 109270] and *ANK1* [MIM: 612641]) linked with hereditary spherocytosis,[81] a disorder that can lead to low HbA1c measurements.[82]

Pathogenic effect enrichment tests

In our exome-wide burden tests for "pathogenic" misaligned status (higher-than-expected misalignment, except for BMD and AAM, for which pathogenic misaligned status is lower-than-expected misalignment), 37 genes across five traits harbored significant enrichment (FDR-adjusted one-sided burden $p_{\text{adj}} \leq 0.05$) of pLoF or damaging missense variants (Figure 5B; Table S9).

For taller-than-expected height, significant enrichment was observed for both pLoF (4 genes) and damaging missense variants (26 genes). Several of the significant genes are associated with autosomal-recessive genetic syndromes that can result in short stature, including *SPINK5* (MIM: 605010; Netherton syndrome[83] [MIM: 256500]), *PLOD2* (MIM: 601865; Bruck syndrome 2[84] [MIM: 609220]), and *GALNS* (MIM: 612222; mucopolysaccharidosis, type IVA[85] [MIM: 253000]). These are counter to the direction of effect expected for taller-than-expected height misalignment; however, none of the taller-than-expected height outliers were homozygous or compound heterozygous for ClinVar[86] pathogenic variants from the variant category (pLoF or damaging missense) being tested (Figure S11). This suggests that these carriers are unlikely to have these syndromes, resolving this paradox.

For higher-than expected LDL-C (2 significant genes), one gene with a biologically plausible mechanism was *TSC2* (burden $p_{\text{misaligned}} = 2.01 \times 10^{-4}$, $p_{\text{adj}} = 0.0188$, $p_{\text{emp}} = 4.00 \times 10^{-3}$; Figure 5B; Table S9; MIM: 191092). *TSC2* inhibits the protein kinase mechanistic target of rapamycin complex 1 (mTORC1)[87]; when mTORC1 is left uninhibited, it can promote *de novo* lipogenesis.[88] The other significant gene, *ANK2* (burden $p_{\text{misaligned}} = 1.09 \times 10^{-6}$, $p_{\text{adj}} = 2.96 \times 10^{-4}$; MIM: 106410), is implicated in cardiac arrhythmia, ankyrin-B related[89] (MIM: 600919), but no evidence in the literature suggests that cardiac arrhythmia can cause high cholesterol.

For higher-than-expected HbA1c misalignment, the only significant association was for damaging missense burden in *PIK3C2B* (burden $p_{\text{misaligned}} = 2.93 \times 10^{-4}$, $p_{\text{adj}} = 0.0252$, $p_{\text{emp}} = 0.218$; Figure 5B; Table S9; MIM: 602838). There is a plausible pathway implicating this gene in HbA1c, as it encodes a protein involved in the phosphatidylinositol 3-kinase (PI3K)/AKT/mTOR pathway, a critical regulator of insulin signaling and overall glucose homeostasis.[90,91]

For the remaining traits, there were significant pathogenic damaging missense burden associations for higher-than-expected BMI (*KLHL17* [MIM: 619262]) and lower-than-expected BMD (*OTOG* [MIM: 604487], *ABCC2* [MIM: 601107], and *MTMR2* [MIM: 603557]). However, these genes did not have strong evidence from existing literature to support their biological plausibility.

Discussion

In this study, we tested a collection of continuous and dichotomous traits for enrichment of rare deleterious

variants in individuals whose observed phenotype deviates from common-variant genetic expectation. We began with a narrow focus, testing a small curated set of genes canonically implicated in each trait, before expanding to testing larger gene panels from GEL for rare disorders related to the traits. Finally, we broadened our scope to an exome-wide scan, searching for gene burden associations with phenotype misalignment.

Using an existing method of misaligned phenotype classification in continuous traits, we replicated many of the pLoF enrichment results previously observed[26] for misaligned height and LDL-C. We also found that individuals with lower than genetically expected LDL-C and higher than genetically expected stature were enriched for rare damaging missense variants in *APOB* and *FBN1*, respectively. This suggests that phenotype misalignment may be influenced by variants expected to have more moderate effects on gene function. Another observation was that, in BMD, a trait not investigated by previous studies of misaligned phenotypes, misaligned individuals were enriched for rare pLoF variants in *COPB2* and *GORAB*, genes linked with a monogenic form of OP. These results further validate the use of misalignment classification for prioritizing individuals when screening for rare genetic disorders related to height, LDL-C, and BMD.

Testing for pLoF enrichment in individuals misaligned with disease status, we found that T2D disease cases harboring pathogenic rare pLoFs in certain genes associated with MODY tended to have significantly lower PRSs. We also observed that CAD disease controls harboring nominally significant protective rare pLoFs in *ANGPTL3* tended to have higher PRSs. As with the continuous traits, these results support the use of PRS when prioritizing individuals for rare disorder screening. These results also provide strong evidence of a liability threshold model where disease status is determined by a balance of pathogenic and protective effects from common and rare variants (Figure 1).

Moving from validation to discovery, we tested exome wide for genes associated with protective or pathogenic misaligned continuous phenotype status and identified several genes with plausible biological pathways for their role in causing high or low misaligned phenotypes, including genes associated with lower-than-expected BMI (*ACSL6*, *FAT1*, and *NPL*) and higher-than-expected LDL-C (*TSC2*) and AAM (*KANK1*). This demonstrated that misalignment classification of continuous traits could improve power in gene discovery, especially for noisy self-reported phenotypes, such as AAM.[92] We also performed an exome-wide scan for genes associated with misaligned disease status (Note S2), but this was less fruitful, yielding a single association in *HLA-DRB5* (MIM: 604776) for CAD-protective pLoF burden. This could be because there are not many genes with rare variant burden effects large enough to counteract common variant effects and cause misaligned disease status.

There were some limitations in this study. First, testing for enrichment of rare damaging variants among misaligned individuals has inherently limited statistical power due to sparse variant presence among misaligned individuals. For example, when testing continuous traits, there were zero misaligned individuals harboring rare deleterious variants for more than half (32/58) of the canonical gene enrichment tests and 78.7% (389/494) of GEL PanelApp gene enrichment tests across all pLoF and damaging missense enrichment tests. This made it impossible to assess relative enrichment of damaging variants in misaligned individuals versus aligned individuals in these instances. In addition to the double jeopardy in statistical power when testing for enrichment of rare variants in a rare group of individuals, there are also expected to be fewer pathogenic variants in the UKB because, on average, UKB participants are healthier than the general population.[93] Second, we used variant consequence categories based on computational prediction (methods) rather than strictly clinical evidence. Using computational prediction may lead to some miscategorized variants but enabled us to scale the enrichment tests to many more variants than would be feasible through manual curation of single variants informed by clinical case studies. Third, throughout this paper, we use PGSs/PRSs generated by a single study,[25] provided by the UKB. We chose these scores because they were rigorously defined, readily available, and generated independent from our work. Evaluating the performance of our misalignment testing approach across PGSs generated by other methods could further generalize our findings and determine what aspects of a PGS make it particularly suited to misalignment testing. Finally, this study was performed solely in individuals classified as having European genetic ancestry (methods). Identifying misaligned individuals in non-European populations is difficult, as these populations are generally less represented in genome-wide association studies (GWASs) with sufficient sample size to generate informative PGSs. However, as increasingly diverse large biobanks are created, such as the *All of Us* (AoU) dataset,[94] misalignment classification will become feasible for more ancestry groups.

There are several areas of opportunity to build on this work. Non-genetic factors, such as SES, medication, or lifestyle choices, could also explain phenotype misalignment. We show evidence that such factors are significantly associated with phenotype misalignment (Note S1). Correcting for these non-genetic factors could also improve power for detecting rare genetic effects on misalignment. For instance, adjusting LDL-C for cholesterol-lowering medication appeared to improve power for canonical gene burden associations (Note S3). Expanding this level of bespoke adjustment for non-genetic factors to all traits could boost power in future work. Replication of results in an independent cohort, such as the AoU dataset,[94] would further increase confidence in results. Some traits had a much lower sample size (BMD, IOP, and AAM; $n < 204,000$) than others (height, LDL-C, BMI, and HbA1c; $n > 320,000$), which made it

difficult to interpret how effective this analysis was in finding associations. Replicating the results in a larger dataset, with greater coverage for those low-sample-size traits, could enable a better comparison of statistical power. Hypothesizing that singletons observed in the UKB could be harboring large-effect variants such as *de novo* variants, misaligned individuals could be tested for enrichment of singleton burden. We performed this analysis and found moderate evidence pointing to deleterious singleton burden associating with shorter-than-expected stature (Note S4). Singleton burden analysis could be refined by calling *de novo* variants and testing their burden directly; however, this was beyond the scope of this project. Unlike the gene-level tests used throughout our work, performing a variant-level exome-wide scan for association could highlight the specific variants driving gene-level associations. However, this higher-resolution approach may only be practical for rare but not ultra-rare variants, as statistical power to detect very rare variant associations (e.g., MAC $\leq$ 10) is attenuated compared to associations in more common variants with the same effect size.[95] Last, when performing exome-wide scans for protective genetic effects associated with PRS in disease controls, we only tested pLoF burden. This was because we expected pLoFs to have a much stronger effect than other missense variants, with a strong effect presumably necessary to cause disease status misalignment. However, as observed when testing gene burden associated with misaligned status in continuous traits, there may be value in expanding to damaging missense variants.

We demonstrated that, for a range of heritable continuous traits and complex diseases, misaligned individuals are enriched for rare damaging variants. This work highlights the value of considering both common and rare genetic components of trait variability, as it may prioritize certain individuals for rare-disease diagnosis and could be leveraged to find pathogenic or protective associations.

Data and code availability

Full summary statistics for the exome-wide scan and empirical null analyses are available at https://doi.org/10.5281/zenodo.20110184. Code used for this analysis is available at https://github.com/lindgrengroup/ukb_misaligned. Our pipeline for REGENIE-based association testing is available at https://github.com/lindgrengroup/ukb_wes_450k_gwas.

Acknowledgments

N.A.B. is supported by the Pembroke College Oxford-Bendich Graduate Scholarship, the Clarendon Fund, and Wellcome Trust grant 224890/Z/21/Z. S.S.V. is supported by the Rhodes Scholarships, the Clarendon Fund, and the Medical Sciences Doctoral Training Centre at the University of Oxford. F.H.L. is supported by the Wellcome Trust (award 224894/Z/21/Z) and the Medical Sciences Doctoral Training Centre at the University of Oxford. C.M.L. is supported by the Li Ka Shing Foundation; the NIHR Oxford Biomedical Research Centre, Oxford; the NIH (1P50HD104224-01); and the Gates Foundation (INV-024200). This research was supported by Wellcome Trust core award grant 203141/Z/16/Z and a Wellcome Trust residual award (221782/Z/20/Z) with additional support from the NIHR Oxford BRC. The views expressed are those of the authors and not necessarily those of the NHS, the NIHR, or the Department of Health. H.C. is supported by the Wellcome Trust (318918/Z/24/Z) and the NIH (1P50HD104224-01). D.S.P. is supported by the Pioneer Center for Statistical and Computational Methods for Advanced Research to Transform Biomedicine (SMARTbiomed), DNRF grant P4. This research was conducted using the UK Biobank resource under application number 11867. We thank UK Biobank participants for their contributions.

Author contributions

Conceptualization, C.M.L. and N.A.B.; funding acquisition, C.M.L.; data curation, N.A.B., F.H.L., and B.H.; methodology, N.A.B., D.S.P., S.S.V., and H.C.; software, N.A.B., F.H.L., and B.H.; formal analysis, N.A.B. and F.H.L.; writing – original draft, N.A.B.; writing – review & editing, N.A.B. and D.S.P.; visualization, N.A.B.; project administration, N.A.B., C.M.L., and D.S.P.; supervision, C.M.L. and D.S.P.

Declaration of interests

S.S.V. is currently employed by Illumina but while she conducted the research described in this manuscript was only affiliated with the University of Oxford. C.M.L. is currently a part-time employee of the Ellison Institute of Technology and Population Health Partners (PHP), is chief science officer of Aneira Health, owns equity in PHP and its subsidiaries, and has a partner who works at Vertex.

Supplemental information

Supplemental information can be found online at https://doi.org/10.1016/j.ajhg.2026.05.013.

Received: January 7, 2026
Accepted: May 26, 2026
Published: June 22, 2026

References

1. Falconer, D.S. (1965). The inheritance of liability to certain diseases, estimated from the incidence among relatives. Ann. Hum. Genet. *29*, 51–76. https://doi.org/10.1111/j.1469-1809.1965.tb00500.x.
2. Zuk, O., Hechter, E., Sunyaev, S.R., and Lander, E.S. (2012). The mystery of missing heritability: Genetic interactions create phantom heritability. Proc. Natl. Acad. Sci. USA *109*, 1193–1198. https://doi.org/10.1073/pnas.1119675109.
3. Gibson, G. (2012). Rare and common variants: twenty arguments. Nat. Rev. Genet. *13*, 135–145. https://doi.org/10.1038/nrg3118.
4. Ma, Y., and Zhou, X. (2021). Genetic prediction of complex traits with polygenic scores: a statistical review. Trends Genet. *37*, 995–1011. https://doi.org/10.1016/j.tig.2021.06.004.

5. Wray, N.R., Goddard, M.E., and Visscher, P.M. (2007). Prediction of individual genetic risk to disease from genome-wide association studies. Genome Res. *17*, 1520–1528. https://doi.org/10.1101/gr.6665407.
6. Khera, A.V., Emdin, C.A., Drake, I., Natarajan, P., Bick, A.G., Cook, N.R., Chasman, D.I., Baber, U., Mehran, R., Rader, D.J., et al. (2016). Genetic risk, adherence to a healthy lifestyle, and coronary disease. N. Engl. J. Med. *375*, 2349–2358. https://doi.org/10.1056/NEJMoa1605086.
7. Maas, P., Barrdahl, M., Joshi, A.D., Auer, P.L., Gaudet, M.M., Milne, R.L., Schumacher, F.R., Anderson, W.F., Check, D., Chattopadhyay, S., et al. (2016). Breast cancer risk from modifiable and nonmodifiable risk factors among white women in the United States. JAMA Oncol. *2*, 1295–1302. https://doi.org/10.1001/jamaoncol.2016.1025.
8. Natarajan, P., Young, R., Stitziel, N.O., Padmanabhan, S., Baber, U., Mehran, R., Sartori, S., Fuster, V., Reilly, D.F., Butterworth, A., et al. (2017). Polygenic risk score identifies subgroup with higher burden of atherosclerosis and greater relative benefit from statin therapy in the primary prevention setting. Circulation *135*, 2091–2101. https://doi.org/10.1161/CIRCULATIONAHA.116.024436.
9. Torkamani, A., Wineinger, N.E., and Topol, E.J. (2018). The personal and clinical utility of polygenic risk scores. Nat. Rev. Genet. *19*, 581–590. https://doi.org/10.1038/s41576-018-0018-x.
10. Jurgens, S.J., Pirruccello, J.P., Choi, S.H., Morrill, V.N., Chaffin, M., Lubitz, S.A., Lunetta, K.L., and Ellinor, P.T. (2023). Adjusting for common variant polygenic scores improves yield in rare variant association analyses. Nat. Genet. *55*, 544–548. https://doi.org/10.1038/s41588-023-01342-w.
11. Lu, T., Zhou, S., Wu, H., Forgetta, V., Greenwood, C.M.T., and Richards, J.B. (2021). Individuals with common diseases but with a low polygenic risk score could be prioritized for rare variant screening. Genet. Med. *23*, 508–515. https://doi.org/10.1038/s41436-020-01007-7.
12. Huang, Q.Q., Wigdor, E.M., Malawsky, D.S., Campbell, P., Samocha, K.E., Chundru, V.K., Danecek, P., Lindsay, S., Marchant, T., Koko, M., et al. (2024). Examining the role of common variants in rare neurodevelopmental conditions. Nature *636*, 404–411. https://doi.org/10.1038/s41586-024-08217-y.
13. Fahed, A.C., Wang, M., Homburger, J.R., Patel, A.P., Bick, A.G., Neben, C.L., Lai, C., Brockman, D., Philippakis, A., Ellinor, P.T., et al. (2020). Polygenic background modifies penetrance of monogenic variants for tier 1 genomic conditions. Nat. Commun. *11*, 3635–3639. https://doi.org/10.1038/s41467-020-17374-3.
14. Kuchenbaecker, K.B., McGuffog, L., Barrowdale, D., Lee, A., Soucy, P., Dennis, J., Domchek, S.M., Robson, M., Spurdle, A.B., Ramus, S.J., et al. (2017). Evaluation of Polygenic Risk Scores for Breast and Ovarian Cancer Risk Prediction in BRCA1 and BRCA2 Mutation Carriers. J. Natl. Cancer Inst. *109*, djw302. https://doi.org/10.1093/jnci/djw302.
15. Mars, N., Widén, E., Kerminen, S., Meretoja, T., Pirinen, M., Briotta Parolo, P. della, Palta, P., Palotie, A., Kaprio, J., Joensuu, H., et al. (2020). The role of polygenic risk and susceptibility genes in breast cancer over the course of life. Nat. Commun. *11*, 1–9. https://doi.org/10.1038/s41467-020-19966-5.
16. Genetic Modifiers of Huntington's Disease (GeM-HD) Consortium (2015). Identification of Genetic Factors that Modify Clinical Onset of Huntington's Disease. Cell *162*, 516–526. https://doi.org/10.1016/j.cell.2015.07.003.
17. Moss, D.J.H., Pardiñas, A.F., Langbehn, D., Lo, K., Leavitt, B.R., Roos, R., Durr, A., Mead, S., Coleman, A., Santos, R.D., et al. (2017). Identification of genetic variants associated with Huntington's disease progression: a genome-wide association study. Lancet Neurol. *16*, 701–711. https://doi.org/10.1016/S1474-4422(17)30161-8.
18. Craig, J.E., Han, X., Qassim, A., Hassall, M., Cooke Bailey, J.N., Kinzy, T.G., Khawaja, A.P., An, J., Marshall, H., Gharahkhani, P., et al. (2020). Multitrait analysis of glaucoma identifies new risk loci and enables polygenic prediction of disease susceptibility and progression. Nat. Genet. *52*, 160–166. https://doi.org/10.1038/s41588-019-0556-y.
19. Zebardast, N., Sekimitsu, S., Wang, J., Elze, T., Gharahkhani, P., Cole, B.S., Lin, M.M., Segrè, A.V., Wiggs, J.L., Aung, T., et al. (2021). Characteristics of p.Gln368Ter myocilin variant and influence of polygenic risk on glaucoma penetrance in the UK Biobank. Ophthalmology *128*, 1300–1311. https://doi.org/10.1016/j.ophtha.2021.03.007.
20. Oetjens, M.T., Kelly, M.A., Sturm, A.C., Martin, C.L., and Ledbetter, D.H. (2019). Quantifying the polygenic contribution to variable expressivity in eleven rare genetic disorders. Nat. Commun. *10*, 4897. https://doi.org/10.1038/s41467-019-12869-0.
21. Kingdom, R., Beaumont, R.N., Wood, A.R., Weedon, M.N., and Wright, C.F. (2024). Genetic modifiers of rare variants in monogenic developmental disorder loci. Nat. Genet. *56*, 861–868. https://doi.org/10.1038/s41588-024-01710-0.
22. Niemi, M.E.K., Martin, H.C., Rice, D.L., Gallone, G., Gordon, S., Kelemen, M., McAloney, K., McRae, J., Radford, E.J., Yu, S., et al. (2018). Common genetic variants contribute to risk of rare severe neurodevelopmental disorders. Nature *562*, 268–271. https://doi.org/10.1038/s41586-018-0566-4.
23. Khan, A., Shang, N., Nestor, J.G., Weng, C., Hripcsak, G., Harris, P.C., Gharavi, A.G., and Kiryluk, K. (2023). Polygenic risk alters the penetrance of monogenic kidney disease. Nat. Commun. *14*, 8318. https://doi.org/10.1038/s41467-023-43878-9.
24. Tansey, K.E., Rees, E., Linden, D.E., Ripke, S., Chambert, K.D., Moran, J.L., McCarroll, S.A., Holmans, P., Kirov, G., Walters, J., et al. (2015). Common alleles contribute to schizophrenia in CNV carriers. Mol. Psychiatry *21*, 1085–1089. https://doi.org/10.1038/mp.2015.143.
25. Thompson, D.J., Wells, D., Selzam, S., Peneva, I., Moore, R., Sharp, K., Tarran, W.A., Beard, E.J., Riveros-Mckay, F., Giner-Delgado, C., et al. (2024). A systematic evaluation of the performance and properties of the UK Biobank Polygenic Risk Score (PRS) Release. PLoS One *19*, e0307270. https://doi.org/10.1371/journal.pone.0307270.
26. Hawkes, G., Yengo, L., Vedantam, S., Marouli, E., Beaumont, R.N., Tyrrell, J., Weedon, M.N., Hirschhorn, J., Frayling, T.M., Wood, A.R., et al. (2023). Identification and analysis of individuals who deviate from their genetically-predicted phenotype. PLoS Genet. *19*, e1010934. https://doi.org/10.1371/journal.pgen.1010934.
27. Martin, A.R., Williams, E., Foulger, R.E., Leigh, S., Daugherty, L.C., Niblock, O., Leong, I.U.S., Smith, K.R., Gerasimenko, O., Haraldsdottir, E., et al. (2019). PanelApp crowdsources expert knowledge to establish consensus diagnostic gene panels. Nat. Genet. *51*, 1560–1565. https://doi.org/10.1038/s41588-019-0528-2.
28. Karczewski, K.J., Solomonson, M., Chao, K.R., Goodrich, J.K., Tiao, G., Lu, W., Riley-Gillis, B.M., Tsai, E.A., Kim, H.I.,

Zheng, X., et al. (2022). Systematic single-variant and gene-based association testing of thousands of phenotypes in 394,841 UK Biobank exomes. Cell Genom. *2*, 100168. https://doi.org/10.1016/j.xgen.2022.100168.

29. Lassen, F.H., Venkatesh, S.S., Baya, N., Hill, B., Zhou, W., Bloemendal, A., Neale, B.M., Kessler, B.M., Whiffin, N., Lindgren, C.M., and Palmer, D.S. (2024). Exome-wide evidence of compound heterozygous effects across common phenotypes in the UK Biobank. Cell Genom. *4*, 100602. https://doi.org/10.1016/j.xgen.2024.100602.
30. Genomes, P.C., Auton, A., Brooks, L.D., Durbin, R.M., Garrison, E.P., Kang, H.M., Korbel, J.O., Marchini, J.L., McCarthy, S., McVean, G.A., et al. (2015). A global reference for human genetic variation. Nature *526*, 68–74. https://doi.org/10.1038/nature15393.
31. McLaren, W., Gil, L., Hunt, S.E., Riat, H.S., Ritchie, G.R.S., Thormann, A., Flicek, P., and Cunningham, F. (2016). The Ensembl Variant Effect Predictor. Genome Biol. *17*, 122. https://doi.org/10.1186/s13059-016-0974-4.
32. Karczewski, K.J., Francioli, L.C., Tiao, G., Cummings, B.B., Alföldi, J., Wang, Q., Collins, R.L., Laricchia, K.M., Ganna, A., Birnbaum, D.P., et al. (2020). The mutational constraint spectrum quantified from variation in 141,456 humans. Nature *581*, 434–443. https://doi.org/10.1038/s41586-020-2308-7.
33. Liu, X., Li, C., Mou, C., Dong, Y., and Tu, Y. (2020). dbNSFP v4: a comprehensive database of transcript-specific functional predictions and annotations for human nonsynonymous and splice-site SNVs. Genome Med. *12*, 103. https://doi.org/10.1186/s13073-020-00803-9.
34. Rentzsch, P., Witten, D., Cooper, G.M., Shendure, J., and Kircher, M. (2019). CADD: predicting the deleteriousness of variants throughout the human genome. Nucleic Acids Res. *47*, D886–D894. https://doi.org/10.1093/nar/gky1016.
35. Ioannidis, N.M., Rothstein, J.H., Pejaver, V., Middha, S., McDonnell, S.K., Baheti, S., Musolf, A., Li, Q., Holzinger, E., Karyadi, D., et al. (2016). REVEL: An ensemble method for predicting the pathogenicity of rare missense variants. Am. J. Hum. Genet. *99*, 877–885. https://doi.org/10.1016/j.ajhg.2016.08.016.
36. Jaganathan, K., Kyriazopoulou Panagiotopoulou, S., McRae, J.F., Darbandi, S.F., Knowles, D., Li, Y.I., Kosmicki, J.A., Arbelaez, J., Cui, W., Schwartz, G.B., et al. (2019). Predicting Splicing from Primary Sequence with Deep Learning. Cell *176*, 535–548.e24. https://doi.org/10.1016/j.cell.2018.12.015.
37. Morales, J., Pujar, S., Loveland, J.E., Astashyn, A., Bennett, R., Berry, A., Cox, E., Davidson, C., Ermolaeva, O., Farrell, C.M., et al. (2022). A joint NCBI and EMBL-EBI transcript set for clinical genomics and research. Nature *604*, 310–315. https://doi.org/10.1038/s41586-022-04558-8.
38. Richards, S., Aziz, N., Bale, S., Bick, D., Das, S., Gastier-Foster, J., Grody, W.W., Hegde, M., Lyon, E., Spector, E., et al. (2015). Standards and guidelines for the interpretation of sequence variants: a joint consensus recommendation of the American College of Medical Genetics and Genomics and the Association for Molecular Pathology. Genet. Med. *17*, 405–424. https://doi.org/10.1038/gim.2015.30.
39. Ehrlich, J.R., Radcliffe, N.M., and Shimmyo, M. (2012). Goldmann applanation tonometry compared with corneal-compensated intraocular pressure in the evaluation of primary open-angle Glaucoma. BMC Ophthalmol. *12*, 52. https://doi.org/10.1186/1471-2415-12-52.
40. Loos, R.J.F., and Yeo, G.S.H. (2022). The genetics of obesity: from discovery to biology. Nat. Rev. Genet. *23*, 120–133. https://doi.org/10.1038/s41576-021-00414-z.
41. Mäkitie, R.E., Costantini, A., Kämpe, A., Alm, J.J., and Mäkitie, O. (2019). New Insights Into Monogenic Causes of Osteoporosis. Front. Endocrinol. *10*, 432844. https://doi.org/10.3389/fendo.2019.00070.
42. Kwon, Y.H., Fingert, J.H., Kuehn, M.H., and Alward, W.L.M. (2009). Primary open-angle glaucoma. N. Engl. J. Med. *360*, 1113–1124. https://doi.org/10.1056/NEJMra0804630.
43. Naylor, R.N., John, P.M., Winn, A.N., Carmody, D., Greeley, S.A.W., Philipson, L.H., Bell, G.I., and Huang, E.S. (2014). Cost-effectiveness of MODY genetic testing: translating genomic advances into practical health applications. Diabetes Care *37*, 202–209. https://doi.org/10.2337/dc13-0410.
44. Frayling, T.M., Evans, J.C., Bulman, M.P., Pearson, E., Allen, L., Owen, K., Bingham, C., Hannemann, M., Shepherd, M., Ellard, S., and Hattersley, A.T. (2001). beta-cell genes and diabetes: molecular and clinical characterization of mutations in transcription factors. Diabetes *50*, S94. https://doi.org/10.2337/diabetes.50.2007.s94.
45. Shields, B.M., Hicks, S., Shepherd, M.H., Colclough, K., Hattersley, A.T., and Ellard, S. (2010). Maturity-onset diabetes of the young (MODY): how many cases are we missing? Diabetologia *53*, 2504–2508. https://doi.org/10.1007/s00125-010-1799-4.
46. Steele, A.M., Shields, B.M., Shepherd, M., Ellard, S., Hattersley, A.T., and Pearson, E.R. (2010). Increased all-cause and cardiovascular mortality in monogenic diabetes as a result of mutations in the HNF1A gene. Diabet. Med. *27*, 157–161. https://doi.org/10.1111/j.1464-5491.2009.02913.x.
47. The International Expert Committee (2009). International Expert Committee report on the role of the A1C assay in the diagnosis of diabetes. Diabetes Care *32*, 1327–1334. https://doi.org/10.2337/dc09-9033.
48. Selvin, E. (2021). Hemoglobin A1c—Using Epidemiology to Guide Medical Practice: Kelly West Award Lecture 2020. Diabetes Care *44*, 2197–2204. https://doi.org/10.2337/dci21-0035.
49. Martin, S., Samuel, M., Stow, D., Ridsdale, A.M., Chen, J., Young, K.G., Green, H.D., Hattersley, A.T., L'Esperance, V., McKinley, T.J., et al. (2025). Undiagnosed G6PD Deficiency in Black and Asian Individuals Is Prevalent and Contributes to Health Inequalities in Type 2 Diabetes Diagnosis and Complications. Diabetes Care *48*, 1932–1941. https://doi.org/10.2337/dc25-0556.
50. Shekari, S., Stankovic, S., Gardner, E.J., Hawkes, G., Kentistou, K.A., Beaumont, R.N., Mörseburg, A., Wood, A.R., Prague, J.K., Mishra, G.D., et al. (2023). Penetrance of pathogenic genetic variants associated with premature ovarian insufficiency. Nat. Med. *29*, 1692–1699. https://doi.org/10.1038/s41591-023-02405-5.
51. Flannick, J., Thorleifsson, G., Beer, N.L., Jacobs, S.B.R., Grarup, N., Burtt, N.P., Mahajan, A., Fuchsberger, C., Atzmon, G., Benediktsson, R., et al. (2014). Loss-of-function mutations in SLC30A8 protect against type 2 diabetes. Nat. Genet. *46*, 357–363. https://doi.org/10.1038/ng.2915.
52. Stitziel, N.O., Khera, A.V., Wang, X., Bierhals, A.J., Vourakis, A.C., Sperry, A.E., Natarajan, P., Klarin, D., Emdin, C.A., Zekavat, S.M., et al. (2017). ANGPTL3 deficiency and protection against coronary artery disease. J. Am. Coll. Cardiol. *69*, 2054–2063. https://doi.org/10.1016/j.jacc.2017.02.030.

53. Stark, Z., Foulger, R.E., Williams, E., Thompson, B.A., Patel, C., Lunke, S., Snow, C., Leong, I.U.S., Puzriakova, A., Daugherty, L.C., et al. (2021). Scaling national and international improvement in virtual gene panel curation via a collaborative approach to discordance resolution. Am. J. Hum. Genet. *108*, 1551–1557. https://doi.org/10.1016/j.ajhg.2021.06.020.
54. Brereton, R.G. (2015). The chi squared and multinormal distributions: The chi squared and multinormal distributions. J. Chemom. *29*, 9–12. https://doi.org/10.1002/cem.2680.
55. Bycroft, C., Freeman, C., Petkova, D., Band, G., Elliott, L.T., Sharp, K., Motyer, A., Vukcevic, D., Delaneau, O., O'Connell, J., et al. (2018). The UK Biobank resource with deep phenotyping and genomic data. Nature *562*, 203–209. https://doi.org/10.1038/s41586-018-0579-z.
56. Zhou, W., Bi, W., Zhao, Z., Dey, K.K., Jagadeesh, K.A., Karczewski, K.J., Daly, M.J., Neale, B.M., and Lee, S. (2022). SAIGE-GENE+ improves the efficiency and accuracy of set-based rare variant association tests. Nat. Genet. *54*, 1466–1469. https://doi.org/10.1038/s41588-022-01178-w.
57. Fisher, R.A. (1935). The Logic of Inductive Inference. J. R. Stat. Soc. *98*, 39–82. https://doi.org/10.2307/2342435.
58. Davison, A.C., and Hinkley, D.V. (1997). Bootstrap Methods and Their Application (Cambridge University Press). https://doi.org/10.1017/CBO9780511802843.
59. North, B.V., Curtis, D., and Sham, P.C. (2002). A Note on the Calculation of Empirical P Values from Monte Carlo Procedures. Am. J. Hum. Genet. *71*, 439–441. https://doi.org/10.1086/341527.
60. Mbatchou, J., Barnard, L., Backman, J., Marcketta, A., Kosmicki, J.A., Ziyatdinov, A., Benner, C., O'Dushlaine, C., Barber, M., Boutkov, B., et al. (2021). Computationally efficient whole-genome regression for quantitative and binary traits. Nat. Genet. *53*, 1097–1103. https://doi.org/10.1038/s41588-021-00870-7.
61. Purcell, S., Neale, B., Todd-Brown, K., Thomas, L., Ferreira, M.A.R., Bender, D., Maller, J., Sklar, P., de Bakker, P.I.W., Daly, M.J., and Sham, P.C. (2007). PLINK: a tool set for whole-genome association and population-based linkage analyses. Am. J. Hum. Genet. *81*, 559–575. https://doi.org/10.1086/519795.
62. Benjamini, Y., and Hochberg, Y. (1995). Controlling the False Discovery Rate: A Practical and Powerful Approach to Multiple Testing. J. R. Stat. Soc. Ser. B Methodol. *57*, 289–300.
63. Bonnefond, A., Unnikrishnan, R., Doria, A., Vaxillaire, M., Kulkarni, R.N., Mohan, V., Trischitta, V., and Froguel, P. (2023). Monogenic diabetes. Nat. Rev. Dis. Primers *9*, 12. https://doi.org/10.1038/s41572-023-00421-w.
64. Cohen, J.C., Boerwinkle, E., Mosley, T.H., Jr., and Hobbs, H.H. (2006). Sequence variations in PCSK9, low LDL, and protection against coronary heart disease. N. Engl. J. Med. *354*, 1264–1272. https://doi.org/10.1056/NEJMoa054013.
65. Ference, B.A., Robinson, J.G., Brook, R.D., Catapano, A.L., Chapman, M.J., Neff, D.R., Voros, S., Giugliano, R.P., Davey Smith, G., Fazio, S., and Sabatine, M.S. (2016). Variation in PCSK9 and HMGCR and risk of cardiovascular disease and diabetes. N. Engl. J. Med. *375*, 2144–2153. https://doi.org/10.1056/NEJMoa1604304.
66. Peloso, G.M., Nomura, A., Khera, A.V., Chaffin, M., Won, H.-H., Ardissino, D., Danesh, J., Schunkert, H., Wilson, J.G., Samani, N., et al. (2019). Rare Protein-Truncating Variants in APOB, Lower Low-Density Lipoprotein Cholesterol, and Protection Against Coronary Heart Disease. Circ. Genom. Precis. Med *12*, e002376. https://doi.org/10.1161/CIRCGEN.118.002376.
67. Soutar, A.K., and Naoumova, R.P. (2007). Mechanisms of Disease: genetic causes of familial hypercholesterolemia. Nat. Clin. Pract. Cardiovasc. Med. *4*, 214–225. https://doi.org/10.1038/ncpcardio0836.
68. Rauch, F., and Glorieux, F.H. (2004). Osteogenesis imperfecta. Lancet *363*, 1377–1385. https://doi.org/10.1016/S0140-6736(04)16051-0.
69. Teodoro, B.G., Sampaio, I.H., Bomfim, L.H.M., Queiroz, A.L., Silveira, L.R., Souza, A.O., Fernandes, A.M.A.P., Eberlin, M.N., Huang, T.-Y., Zheng, D., et al. (2017). Long-chain acyl-CoA synthetase 6 regulates lipid synthesis and mitochondrial oxidative capacity in human and rat skeletal muscle. J. Physiol. *595*, 677–693. https://doi.org/10.1113/JP272962.
70. Tang, R., Harasymowicz, N.S., Wu, C.-L., Choi, Y.-R., Lenz, K., Oswald, S.J., and Guilak, F. (2024). Gene therapy for fat-1 prevents obesity-induced metabolic dysfunction, cellular senescence, and osteoarthritis. Proc. Natl. Acad. Sci. USA *121*, e2402954121. https://doi.org/10.1073/pnas.2402954121.
71. Da Silva, A., Dort, J., Orfi, Z., Pan, X., Huang, S., Kho, I., Heckel, E., Muscarnera, G., van Vliet, P.P., Sturiale, L., et al. (2023). N-acetylneuraminate pyruvate lyase controls sialylation of muscle glycoproteins essential for muscle regeneration and function. Sci. Adv. *9*, eade6308. https://doi.org/10.1126/sciadv.ade6308.
72. Buniello, A., Suveges, D., Cruz-Castillo, C., Llinares, M.B., Cornu, H., Lopez, I., Tsukanov, K., Roldán-Romero, J.M., Mehta, C., Fumis, L., et al. (2025). Open Targets Platform: facilitating therapeutic hypotheses building in drug discovery. Nucleic Acids Res. *53*, D1467–D1475. https://doi.org/10.1093/nar/gkae1128.
73. Gallagher, C.S., Mäkinen, N., Harris, H.R., Rahmioglu, N., Uimari, O., Cook, J.P., Shigesi, N., Ferreira, T., Velez-Edwards, D.R., Edwards, T.L., et al. (2019). Genome-wide association and epidemiological analyses reveal common genetic origins between uterine leiomyomata and endometriosis. Nat. Commun. *10*, 4857. https://doi.org/10.1038/s41467-019-12536-4.
74. Cybulski, C., Górski, B., Huzarski, T., Masojć, B., Mierzejewski, M., Debniak, T., Teodorczyk, U., Byrski, T., Gronwald, J., Matyjasik, J., et al. (2004). CHEK2 is a multiorgan cancer susceptibility gene. Am. J. Hum. Genet. *75*, 1131–1135. https://doi.org/10.1086/426403.
75. Ruth, K.S., Day, F.R., Hussain, J., Martínez-Marchal, A., Aiken, C.E., Azad, A., Thompson, D.J., Knoblochova, L., Abe, H., Tarry-Adkins, J.L., et al. (2021). Genetic insights into biological mechanisms governing human ovarian ageing. Nature *596*, 393–397. https://doi.org/10.1038/s41586-021-03779-7.
76. Ulin, M., Ali, M., Chaudhry, Z.T., Al-Hendy, A., and Yang, Q. (2020). Uterine Fibroids in Menopause and Perimenopause. Menopause *27*, 238–242. https://doi.org/10.1097/GME.0000000000001438.
77. Fabregat, A., Sidiropoulos, K., Garapati, P., Gillespie, M., Hausmann, K., Haw, R., Jassal, B., Jupe, S., Korninger, F., McKay, S., et al. (2016). The Reactome pathway Knowledgebase. Nucleic Acids Res. *44*, D481–D487. https://doi.org/10.1093/nar/gkv1351.
78. Yang, J.L., Hodara, E., Sriprasert, I., Shoupe, D., and Stanczyk, F.Z. (2024). Estrogen deficiency in the menopause and the role of hormone therapy: integrating the findings of basic science research with clinical trials. Menopause *31*, 926–939. https://doi.org/10.1097/GME.0000000000002407.

79. Lerer, I., Sagi, M., Meiner, V., Cohen, T., Zlotogora, J., and Abeliovich, D. (2005). Deletion of the ANKRD15 gene at 9p24.3 causes parent-of-origin-dependent inheritance of familial cerebral palsy. Hum. Mol. Genet. *14*, 3911–3920. https://doi.org/10.1093/hmg/ddi415.
80. Jacobs, B.M., Stow, D., Hodgson, S., Zöllner, J., Samuel, M., Kanoni, S., Bidi, S., Akhtar, S., Anwar, M., Arciero, E., et al. (2024). Genetic architecture of routinely acquired blood tests in a British South Asian cohort. Nat. Commun. *15*, 8929. https://doi.org/10.1038/s41467-024-53091-x.
81. Eber, S.W., Gonzalez, J.M., Lux, M.L., Scarpa, A.L., Tse, W.T., Dornwell, M., Herbers, J., Kugler, W., Ozcan, R., Pekrun, A., et al. (1996). Ankyrin-1 mutations are a major cause of dominant and recessive hereditary spherocytosis. Nat. Genet. *13*, 214–218. https://doi.org/10.1038/ng0696-214.
82. Rushakoff, R.J., MacMaster, H.W., and Shah, A.D. (2015). Hereditary spherocytosis and other factors that alter HBA1C levels. AACE Clin. Case Rep. *1*, e212–e213. https://doi.org/10.4158/ep14508.cor.
83. Sun, J.D., and Linden, K.G. (2006). Netherton syndrome: a case report and review of the literature. Int. J. Dermatol. *45*, 693–697. https://doi.org/10.1111/j.1365-4632.2005.02637.x.
84. van der Slot, A.J., Zuurmond, A.M., Bardoel, A.F.J., Wijmenga, C., Pruijs, H.E.H., Sillence, D.O., Brinckmann, J., Abraham, D.J., Black, C.M., Verzijl, N., et al. (2003). Identification of PLOD2 as Telopeptide Lysyl Hydroxylase, an Important Enzyme in Fibrosis. J. Biol. Chem. *278*, 40967–40972. https://doi.org/10.1074/jbc.M307380200.
85. Montaño, A.M., Tomatsu, S., Brusius, A., Smith, M., and Orii, T. (2008). Growth charts for patients affected with Morquio A disease. Am. J. Med. Genet. *146A*, 1286–1295. https://doi.org/10.1002/ajmg.a.32281.
86. Landrum, M.J., Lee, J.M., Riley, G.R., Jang, W., Rubinstein, W.S., Church, D.M., and Maglott, D.R. (2014). ClinVar: public archive of relationships among sequence variation and human phenotype. Nucleic Acids Res. *42*, D980–D985. https://doi.org/10.1093/nar/gkt1113.
87. Saxton, R.A., and Sabatini, D.M. (2017). mTOR Signaling in Growth, Metabolism, and Disease. Cell *168*, 960–976. https://doi.org/10.1016/j.cell.2017.02.004.
88. Porstmann, T., Santos, C.R., Griffiths, B., Cully, M., Wu, M., Leevers, S., Griffiths, J.R., Chung, Y.-L., and Schulze, A. (2008). SREBP activity is regulated by mTORC1 and contributes to Akt-dependent cell growth. Cell Metab. *8*, 224–236. https://doi.org/10.1016/j.cmet.2008.07.007.
89. Koenig, S.N., and Mohler, P.J. (2017). The Evolving Role of Ankyrin-B in Cardiovascular Disease. Heart Rhythm *14*, 1884–1889. https://doi.org/10.1016/j.hrthm.2017.07.032.
90. Huang, Y., Xu, W., Chen, M., Wu, Y., Wang, H., Li, J., and Pan, X. (2018). PI3K/AKT Pathway and Type 2 Diabetes Mellitus. Curr. Mol. Med. *18*, 51–63. https://doi.org/10.2174/1566524018666180517112009.
91. Maffei, M., Vitiello, M., Zampella, A., Nappo, G., Bifulco, M., and Diano, N. (2018). PI3K/AKT/mTOR Pathway and Type 2 Diabetes: A New Therapeutic Option for the Disease. Int. J. Mol. Sci. *19*, 4098. https://doi.org/10.3390/ijms19124098.
92. den Tonkelaar, I. (1997). Validity and reproducibility of self-reported age at menopause in women participating in the DOM-project. Maturitas *27*, 117–123. https://doi.org/10.1016/s0378-5122(97)01122-5.
93. Fry, A., Littlejohns, T.J., Sudlow, C., Doherty, N., Adamska, L., Sprosen, T., Collins, R., and Allen, N.E. (2017). Comparison of sociodemographic and health-related characteristics of UK Biobank participants with those of the general population. Am. J. Epidemiol. *186*, 1026–1034. https://doi.org/10.1093/aje/kwx246.
94. All of Us Research Program Genomics Investigators (2024). Genomic data in the All of Us Research Program. Nature *627*, 340–346. https://doi.org/10.1038/s41586-023-06957-x.
95. Liu, Y., Chen, S., Li, Z., Morrison, A.C., Boerwinkle, E., and Lin, X. (2019). ACAT: A fast and powerful p value combination method for rare-variant analysis in sequencing studies. Am. J. Hum. Genet. *104*, 410–421. https://doi.org/10.1016/j.ajhg.2019.01.002.

The American Journal of Human Genetics, Volume *113*

Supplemental information

Individuals who deviate from polygenic expectation are enriched for damaging variants in genes linked to rare disease

Nikolas A. Baya, Frederik H. Lassen, Barney Hill, Samvida S. Venkatesh, Hannah Currant, Cecilia M. Lindgren, and Duncan S. Palmer

Supplemental notes

Note S1: Association of non-genetic factors with continuous phenotype and disease status misalignment relative to genetic expectation

Phenotype misalignment relative to common-variant polygenic score may partly be explained by "non-genetic" factors. We tested association of misaligned status in continuous and dichotomous traits with SES, medication, smoking status, and exercise (Figure S7; Table S4).

Socioeconomic status

Using Townsend Deprivation Index (TDI) (UKB data field: 22189) as a measure for SES, with high TDI corresponding to lower SES, we tested whether higher TDI was associated with a pathogenic misaligned effect. For height, we assumed a "pathogenic" misaligned effect would manifest in shorter stature, per previous evidence[1]. For other continuous traits we tested the misaligned group associated with pathogenic direction of effect, as described in our exome-wide scan for pathogenic burden.

Using logistic regression to test the effect of TDI on pathogenic misaligned status in continuous traits, we observed that misaligned individuals tended to have higher TDI than aligned individuals in every trait ($\beta < 0$, one-sided Wald test $P < 0.05$; Figure S7; Table S4). Among the strongest associations were those in higher than genetically expected BMI ($P = 2.63 \times 10^{-10}$) and HbA1c ($P = 1.07 \times 10^{-8}$), and shorter than genetically expected stature ($P = 1.16 \times 10^{-13}$).

In dichotomous traits, we expected that higher deprivation would have a pathogenic misaligned effect. We used linear regression to test whether higher TDI was associated with decreased PRS in cases and observed significant negative associations ($\beta < 0$, one-sided t-test $P < 0.05$) for T2D and OP (Figure S7; Table S4).

Medication

We investigated the relationship between self-reported medication and phenotype misalignment for three dichotomous traits (T2D, CAD, OP) and their corresponding continuous liability phenotypes (HbA1c, LDL-C, BMD). We mapped self-reported medication (UKB data field: 20003) to Anatomical Therapeutic Chemical (ATC) Index 2026 (`https://atcddd.fhi.no/atc_ddd_index/`) categories using Supplementary Data 1 from Wu *et al.*[2] to define individuals who self-report taking the following ATC categories of medications:

Code	Description	Disease	Liability Trait
A10B	BLOOD GLUCOSE LOWERING DRUGS, EXCL. INSULINS	T2D	HbA1c
C10	LIPID MODIFYING AGENTS	CAD	LDL-C
M05B	DRUGS AFFECTING BONE STRUCTURE AND MINERALIZATION	OP	BMD

We hypothesized that medication would be associated with a protective misaligned effect. Therefore, we tested whether disease controls who reported medication for the disease being tested tended to have higher PRS than individuals not reporting medication. For the liability traits, we tested whether individuals with lower than genetically expected HbA1c or LDL-C, or higher than genetically expected BMD were more likely to report medication relevant to the trait being tested.

We observed that disease controls who reported medication for the tested disease had significantly higher PRS ($\beta > 0$, one-sided t-test $P < 2 \times 10^{-61}$) than individuals not reporting the relevant medication (Figure S7; Table S4), consistent with a protective misaligned effect.

Among the three liability traits, the only trait to show a significant enrichment (Fisher's exact test $P < 0.05$) was LDL-C (OR [90% CI] = 11.7 [8.98-15.2], $P = 1.21 \times 10^{-53}$) (Figure S7; Table S4).

Smoking

We tested whether smoking behavior, specifically if an individual had ever smoked (UKB data field: 20160), was associated with a pathogenic effect on phenotype misalignment.

Using Fisher's exact test, we tested each continuous trait (excluding height, for lack of an expected pathogenic direction of effect), and observed that individuals with lower than genetically expected BMD and higher than genetically expected BMI, HbA1c, and LDL-C were more likely to report having ever smoked (OR > 1, one-sided Fisher's exact test $P < 0.05$; Figure S7).

In dichotomous traits, we used linear regression to test whether smoking status was associated with lower PRS in cases, which implies a pathogenic misaligned effect. We observed a significant association in T2D cases (β [90% CI]$=-0.0391$ [-0.0574, -0.0209], one-sided t-test $P = 2.12 \times 10^{-4}$).

Exercise

We used summed Metabolic Equivalent of Task (MET) minutes per week for all activity (UKB data field: 22040) as an indicator for amount of weekly exercise, hypothesizing that higher amount of exercise would be associated with a protective misaligned effect.

Among continuous traits (with height excluded, for lack of hypothesized "protective" misaligned effect), we observed a significant association (one-sided Wald test $P < 0.05$) in BMI, with low-misaligned individuals having higher MET scores (β [90% CI]$=5.21 \times 10^{-5}$ [4.41×10^{-6}, 9.97×10^{-5}], $P = 0.0361$; Figure S7).

Among disease traits, we found a positive association of MET scores with PRS in T2D controls (β [90% CI] $=3.69 \times 10^{-6}$ [2.46×10^{-6}, 4.92×10^{-6}], one-sided t-test $P = 3.81 \times 10^{-7}$; Figure S7), consistent with a protective misaligned effect of exercise.

Note S2: Exome-wide scan for genes associated with misaligned disease status

Given the significant associations of pathogenic and protective pLoF burden with PRS among disease cases and controls, respectively, we hypothesized that we could discover genes with pathogenic or protective variants by testing exome-wide for PRS association with gene burden.

We performed an exome-wide scan for gene burden associated with protective and pathogenic misaligned disease status, testing burden of rare (MAF $< 0.1\%$) pLoF variants. To test for protective burden, we subset to disease controls for T2D, CAD, and OP, and used REGENIE to test for burden associated with increased PRS (Figure 1). When testing pathogenic burden, we subset to disease cases and tested for burden associated with decreased PRS. As a point of comparison, we also performed traditional burden tests on disease status. All tests were one-sided and P-values were adjusted for FDR$\leq 5\%$ using the BH procedure[3]. FDR correction was performed across results from all phenotypes, but separately for pathogenic and protective tests. There were no FDR-significant associations for pathogenic burden associated with decreased PRS in disease cases (Figure S15; Table S10), thus only the protective burden associations are presented here.

There were two genes where rare (MAF $< 0.1\%$) pLoF variant burden was significantly associated (FDR-adjusted one-sided burden $P_{\text{adj}} \leq 0.05$) with increased PRS in controls for CAD (*HLA-DRB5*) and OP (*NBPF3* [MIM 612992]) (Figure S14, Table S10). After conditioning on common imputed variants within 1 Mb of the gene, the burden association in *HLA-DRB5* remained nominally significant (one-sided burden $P = 3.89 \times 10^{-4}$).

The gene *HLA-DRB5* (major histocompatibility complex, class II, DR beta 5) codes for the beta chain in a human leukocyte antigen (HLA) class II cell surface complex, involved in generating immune responses[4]. Although CAD is linked with immune response in the context of inflammation from atherogenesis[5], there is no evidence in the literature of a plausible connection between *HLA-DRB5* LoF and reduced risk of CAD onset.

Note S3: Adjusting LDL-C for cholesterol-lowering medication

To evaluate the effects of adjusting for a non-genetic factor on enrichment results, we performed a pilot study on LDL-C adjusted for cholesterol-lowering medications for canonical genes associated with monogenic LDL-C disorders. We calculated adjusted LDL-C following the approach of Patel *et al.*[6], which involves applying literature-informed medication-specific adjustments (e.g. dividing observed LDL-C by 0.70 if individuals reported taking statins, or dividing by 0.90 if taking fibrates; see Patel *et al.* Supplemental Table I). We mapped self-reported medication (UKB data field: 20003) to ATC Index 2026 categories using Supplementary Data 1 from Wu *et al.*[2], using the following ATC codes for each cholesterol-lowering medication:

If an individual reported taking medication for cholesterol (UKB data fields: 6177 [males], 6153 [females]) but did not specify the medication in data field 20003, they were assumed to be taking statins, per Patel *et al.*

**Code**	**Medication**
C10AA	HMG CoA reductase inhibitors (Statin)
C10AB	Fibrates
C10AC	Bile acid sequestrants
C10AD	Nicotinic acid and derivatives (Niacin)
C10AX09	Ezetimibe

Testing enrichment of rare deleterious burden in canonical genes associated with monogenic LDL-C disorders, we observed that in all but one gene-consequence pair medication-adjusted LDL-C had higher odds ratio point estimates and lower Fisher's exact test P-values than unadjusted LDL-C (Figure S13; Table S11). The only exception was a marginally higher P-value for the test of pLoF burden in *PCSK9* ($P_{\text{unadjusted}} = 2.97 \times 10^{-11}$, $P_{\text{medication\_adjusted}} = 5.02 \times 10^{-11}$). There was also a nominally significant ($P < 0.05$) result for damaging missense variants in *PCSK9* (OR [90% CI] = 17.0 [5.25, 55.3], one-sided Fisher's exact test $P = 6.59 \times 10^{-3}$), which had been non-significant when using unadjusted LDL-C (OR = 7.33 [1.40, 38.3] $P = 0.129$).

Note S4: Individuals with genetically unexpected phenotypes are nominally enriched for consequence-stratified singleton burden in intolerant genes

Methods for singleton burden association

Singleton variants were defined as variants with an alternate allele count of 1 and alternate allele frequency < 0.01%. We used PLINK[7] to aggregate singleton counts for each chromosome and each variant consequence category. We aggregated singleton burden across all genes predicted to be intolerant to LoF variants. We defined intolerant genes using the gene intolerance threshold (LOEUF< 0.6) proposed by Karczewski *et al.*[8], using gnomAD v4.1 constraint metrics downloaded from the gnomAD website. Of the 18,623 canonical transcripts available, 3,900 were defined as intolerant. Subsetting to intolerant genes is expected to increase signal-to-noise ratio when testing variant effects, because variants in tolerant genes are not expected to affect the function of the genes, and by extension, not expected to affect the trait. This filtering is particularly important for exome-wide aggregated burden because there are many more variants being aggregated together than in the previous single- or grouped-gene tests.

We tested for the effect of consequence-specific singleton burden (s_c, for consequence category c) using regression, controlling for overall singleton burden by including as a covariate the total singleton burden (s_{total}) across consequence categories, per Genovese *et al.*[9]. We used logistic regression when testing misaligned status in continuous traits, and linear regression when testing for effect of singleton burden on covariate-residualised PRS (PRS′) in cases and controls for dichotomous case-control traits:

Continuous traits		Misaligned status $\sim s_c + s_{\text{total}} + 1$
Dichotomous traits	Cases	$\text{PRS}'_{\text{cases}} \sim s_c + s_{\text{total}} + 1$
	Controls	$\text{PRS}'_{\text{controls}} \sim s_c + s_{\text{total}} + 1$

We calculated one-sided P-values for the singleton burden term, given our hypothesis that ultra-rare variants are more likely to cause individuals to be misaligned rather than aligned. We stratified the analysis across four protein-coding variant consequence categories: pLoF, damaging missense, other missense and synonymous (see Methods for variant consequence definitions).

Monotonic point estimates of singleton enrichment in height suggests deleterious singleton burden is associated with shorter stature

Among continuous traits, there were no significant (FDR-adjusted one-sided t-test $P_{\mathrm{adj}} \leq 0.05$) enrichment results (Figure S16; Table S12) after FDR correction. Approaching the results qualitatively, one notable observation was the monotonic trend across functional categories in enrichment effect sizes for height (Figure S16), a trait that showed strong enrichment when testing deleterious burden in genes associated with monogenic and rare disorders. Individuals with lower-than-expected height were increasingly enriched for more deleterious singleton burden, while individuals with higher-than-expected height were increasingly depleted for more deleterious singleton burden. This suggests that, in general, deleterious singletons are more likely to decrease rather than increase height. This is consistent with the work of Backman *et al.*[10], who found that, among genes where pLoF or likely deleterious missense singleton burden was significantly associated with standing height, the burden effects in most (8/10) genes decreased height (see Backman *et al.*[10] Supplementary Data 2). This also aligns with a recent analysis of UKB whole-genome sequencing data[11], in which four genes where pLoF singletons were significantly associated with height, three genes were associated with decreased height (see Hawkes *et al.*[11] Supplementary Data 6).

Limited evidence for association of singleton burden with misaligned disease status

We performed one-sided t-tests for association of singleton burden with PRS for CAD, OP, and T2D. In cases we tested whether higher singleton burden was associated with lower PRS, and in controls we tested whether higher singleton burden was associated with higher PRS. This was based on the same hypotheses used for the canonical and GEL tests: Association of variant burden with lower or higher PRS could indicate effects which are pathogenic or protective, respectively, against common-variant risk scores.

After FDR correction, there were no significant associations (FDR-adjusted one-sided t-test $P_{\mathrm{adj}} \leq 0.05$) (Figure S17; Table S13). In CAD cases, effect size point estimates monotonically decreased with consequence deleteriousness, with pLoF and damaging missense (the two most deleterious consequences) singleton burden being nominally associated (one-sided t-test $P < 0.05$) with lower PRS. This suggests that deleterious singleton burden in intolerant genes could be pathogenic for CAD. We also observed similar monotonicity in effect size point estimates for T2D cases, suggestive of deleterious burden in intolerant genes being pathogenic for T2D, however, none of the enrichment tests were significant.

Supplemental figures

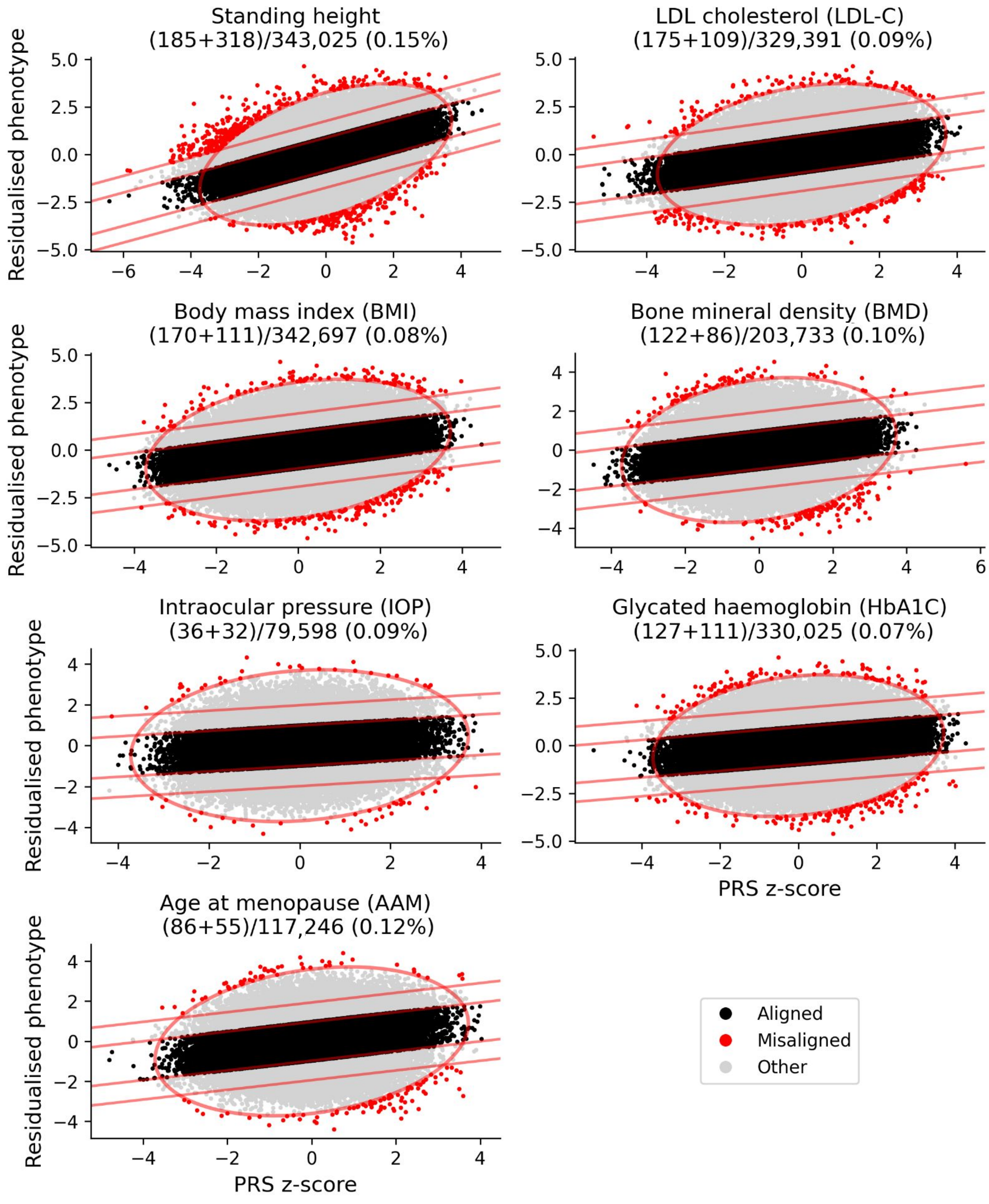

Figure S1: Classification of misaligned individuals for seven continuous traits. In the title of each plot is the name of the phenotype, the sum of low and high misaligned individuals divided by the total number of individuals with defined phenotype, and the total percentage of individuals who are classified as misaligned. The red oval delineates the Mahalanobis distance threshold, while the red lines indicate thresholds on the residuals to define aligned and misaligned individuals.

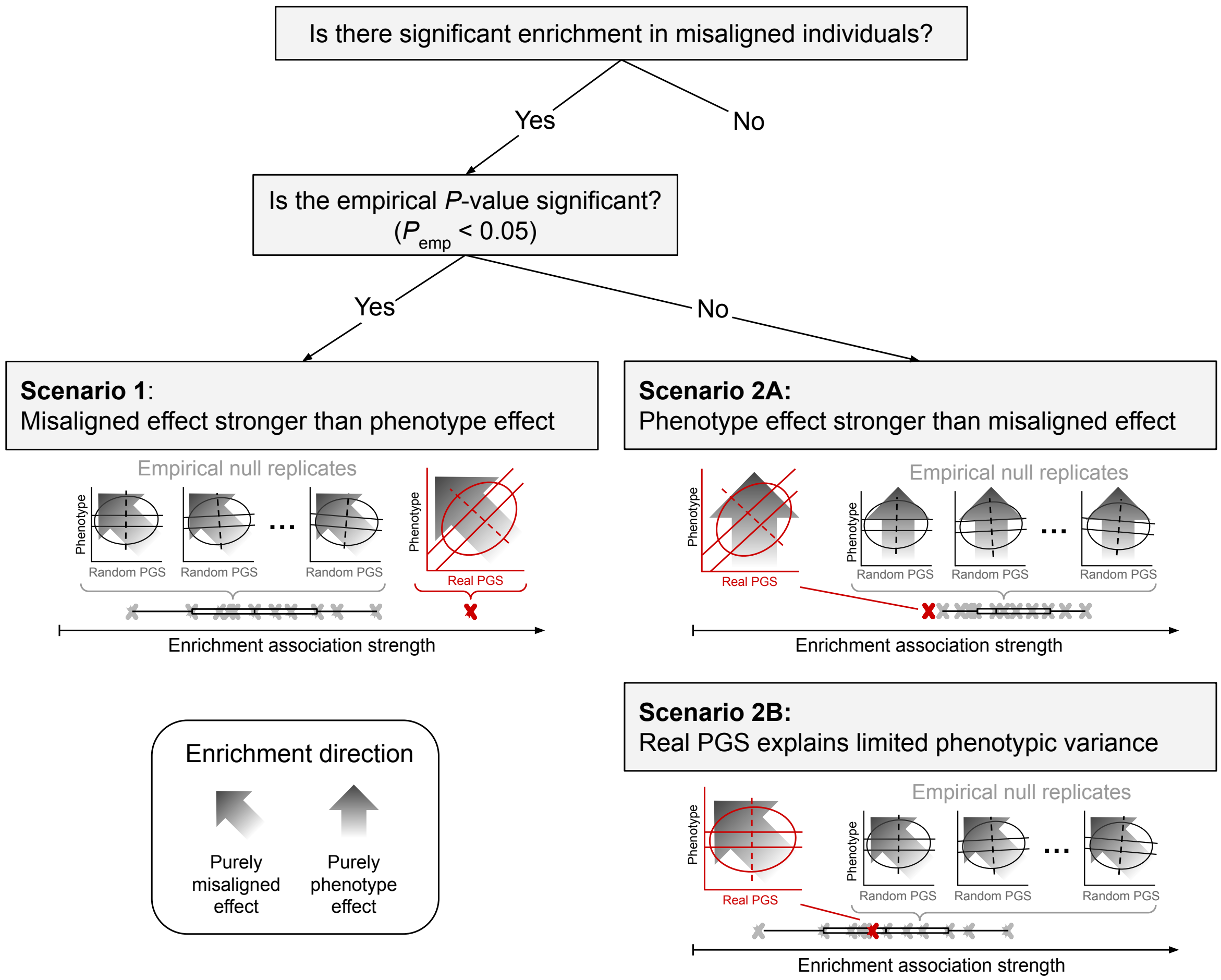

Figure S2: Possible scenarios for the empirical P-value. First, note that the empirical P-value is only calculated for genes which demonstrate significant enrichment of rare deleterious variants among misaligned individuals. The empirical P-value may either be significant or non-significant. To help visualize misaligned enrichment, the gradient of rare deleterious variant enrichment among individuals is shown here as an arrow with gradient shading. The "misaligned axis", orthogonal to the regression of observed phenotype and PGS, is shown as a dashed line. In this schematic, the alignment of the dashed misaligned axis with the enrichment gradient arrows suggests a strong enrichment. The wider the angle between these two, the weaker the enrichment. **Scenario 1** provides an example of a significant empirical P-value, where the observed test statistic (red "×"), based on misaligned enrichment testing using real PGS, outperforms empirical null test statistics (grey ×'s, with box-and-whisker plot for their distribution) generated from misaligned enrichment testing using simulated random PGS. **Scenarios 2A/B** describe examples where spurious enrichment in misaligned individuals can occur, but can be controlled with empirical P-values. Note that in reality, the effects described by these scenarios may be more subtle. It is also possible for a mix of both scenarios to occur. **Scenario 2A** describes the possibility that enrichment of individuals is stronger along the observed phenotype axis, rather than along the misaligned axis (orthogonal to the regression of observed phenotype and PGS). Here, empirical null enrichment tests are expected to outperform the real test statistic because, with the random PGS, the enrichment gradient (arrow) is closely matched with the misaligned axis (dashed line). This results in a non-significant empirical null P-value. **Scenario 2B** describes the possibility of a real PGS explaining limited phenotypic variance, such that the real test statistic is indistinguishable from the empirical null distribution, resulting in a non-significant empirical P-value.

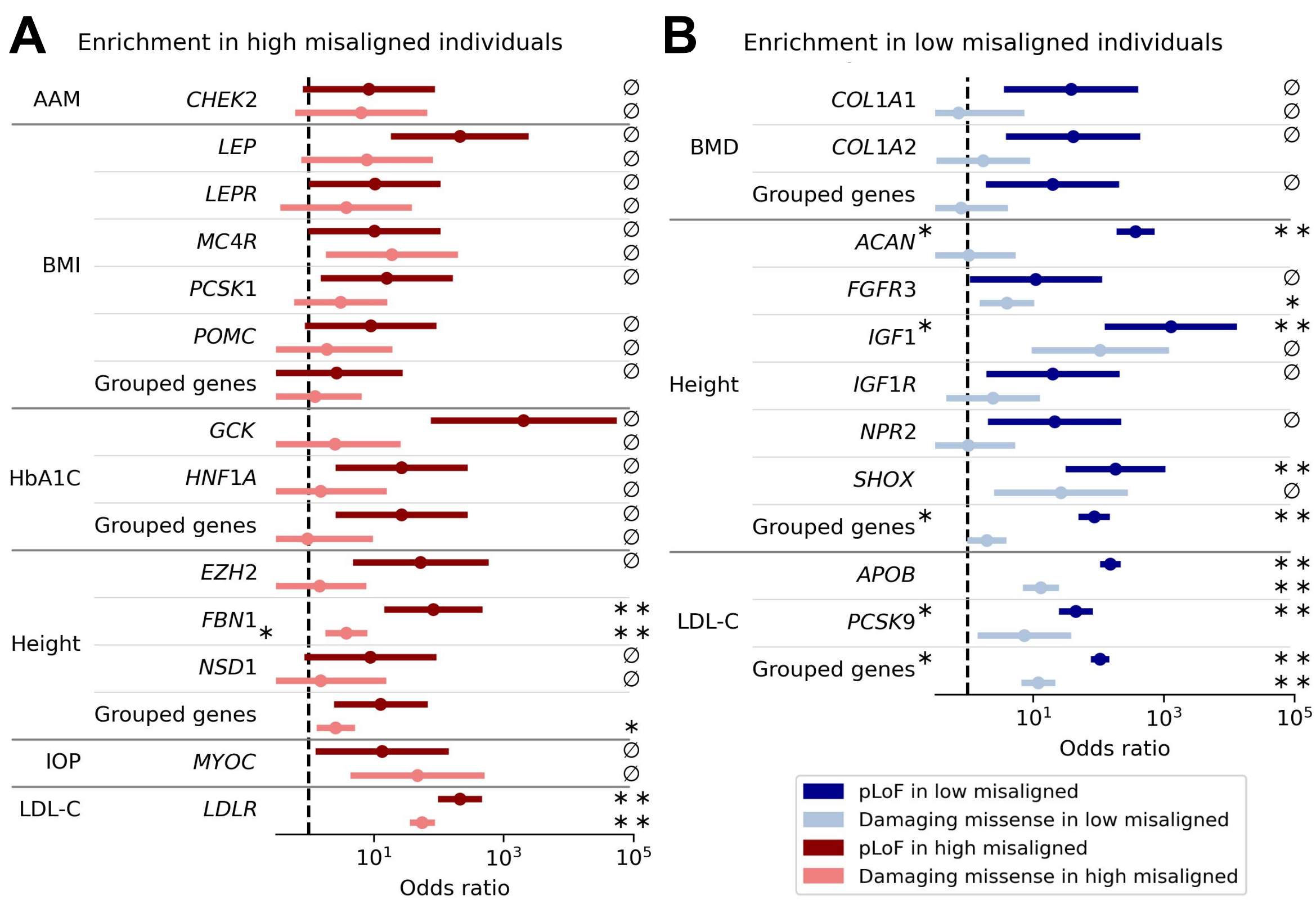

Figure S3: Enrichment of deleterious variants in canonical genes associated with monogenic disorders for continuous traits. (**A**) Enrichment testing in high misaligned individuals, in genes where deleterious variants are expected to cause higher-than-expected phenotypes. (**B**) Enrichment testing in low misaligned individuals, in genes where deleterious variants are expected to cause lower-than-expected phenotypes. Symbols to the right of each forest plot indicate whether the enrichment test was significant ($^{*}P < 0.05$, $^{**}P_{\text{adj}} < 0.05$) or whether misaligned individuals lacked variants ($\emptyset$) to be able to perform Fisher's exact test on a fully non-zero contingency table. Asterisks immediately to the right of the gene symbol indicate tests where the observed enrichment outperformed the empirical null ($P_{\text{emp}} < 0.05$). Odds ratios are calculated for all enrichment tests (with zeros in contingency tables filled with 0.5) and plotted with 90% confidence intervals, such that the lower bound corresponds to the lower bound of a one-sided 95% confidence interval.

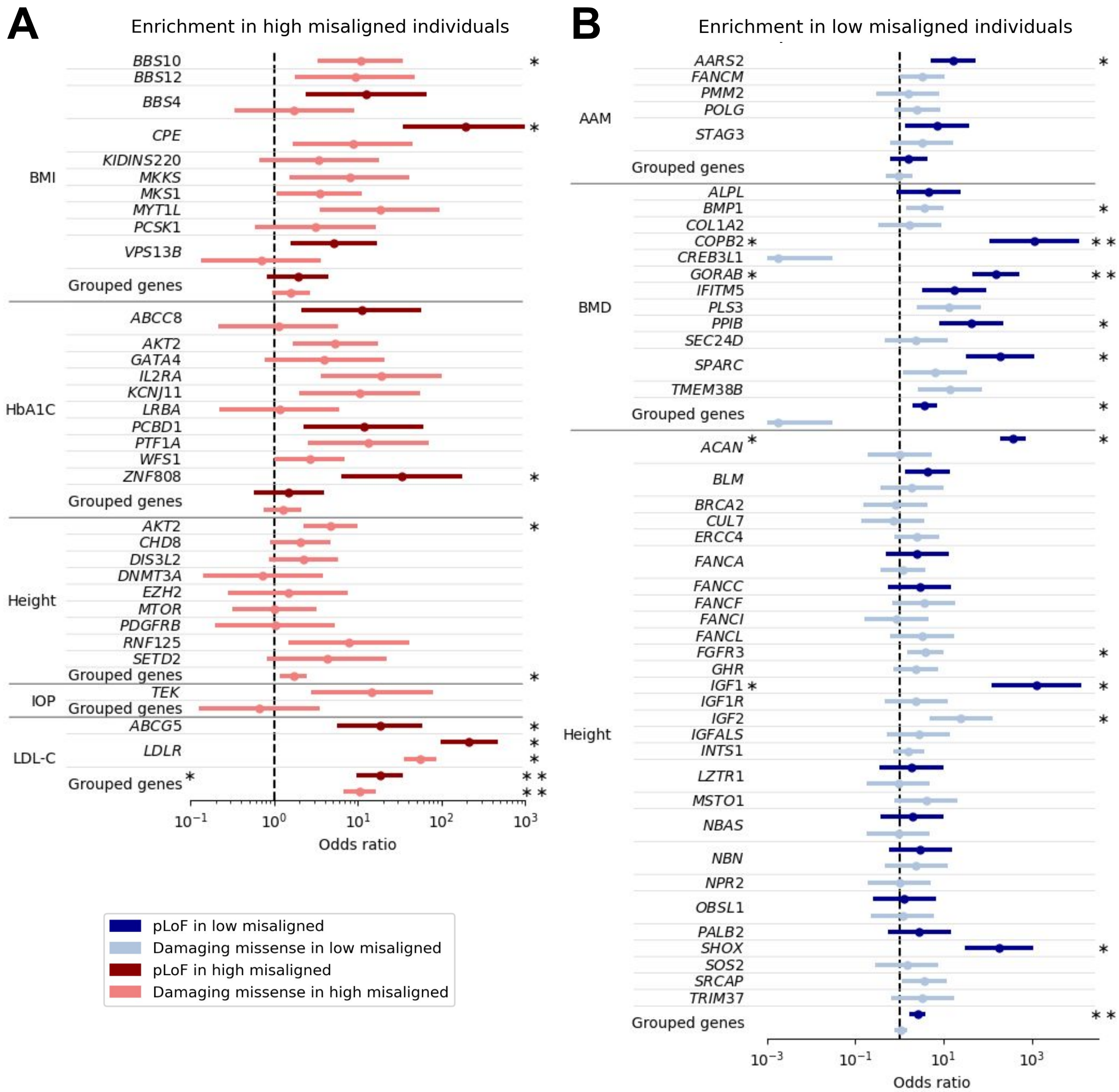

Figure S4: Enrichment of deleterious variants in GEL genes for continuous traits. (**A**) Enrichment testing in high misaligned individuals, in genes where deleterious variants are expected to cause higher-than-expected phenotypes. (**B**) Enrichment testing in low misaligned individuals, in genes where deleterious variants are expected to cause lower-than-expected phenotypes. Asterisks to the right of each forest plot indicate whether the enrichment test was significant ($^{*}P < 0.05$, $^{**}P_{\text{adj}} < 0.05$). Asterisks immediately to the right of the gene symbol indicate tests where the observed enrichment outperformed the empirical null ($P_{\text{emp}} < 0.05$). Odds ratios are calculated for all enrichment tests and plotted with 90% confidence intervals, such that the lower bound corresponds to the lower bound of a one-sided 95% confidence interval. Only enrichment results with fully non-zero contingency tables are shown.

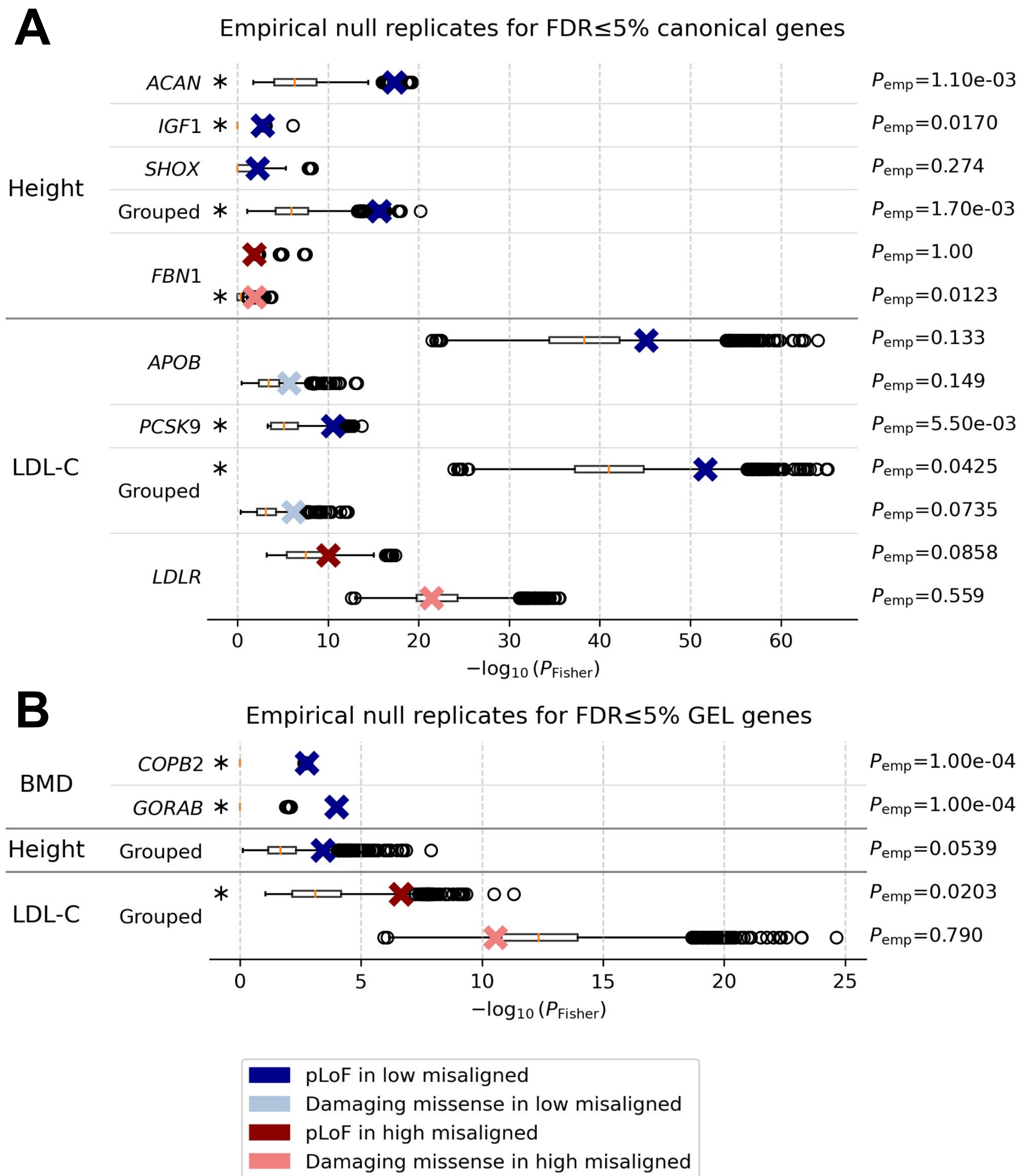

Figure S5: Empirical null replicates for canonical and GEL genes. (**A**) Canonical genes. (**B**) GEL genes. The empirical P-value (P_{emp}, labeled on the right of each plot) of a gene enrichment result indicates how the observed test statistic (one-sided Fisher's exact test P-value) compares against the empirical null distribution of test statistics (Methods). Asterisks immediately to the right of the gene symbol indicate tests where the observed enrichment outperformed the empirical null ($P_{\text{emp}} < 0.05$).

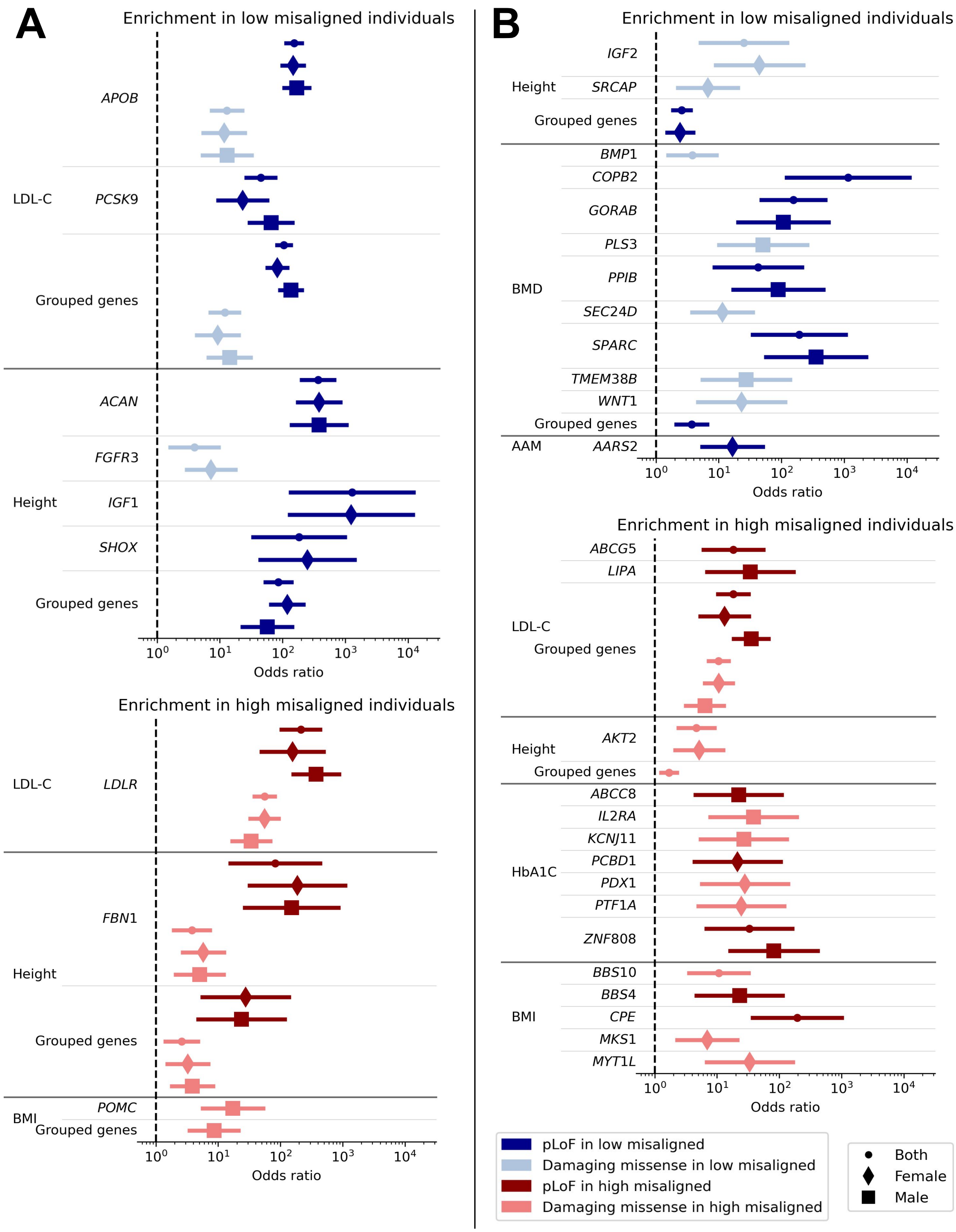

Figure S6: Sex-specific continuous trait enrichment results for canonical and GEL genes. (**A**) Canonical genes. (**B**) GEL genes. Only nominally-significant (one-sided Fisher's exact test < 0.05) results are shown.

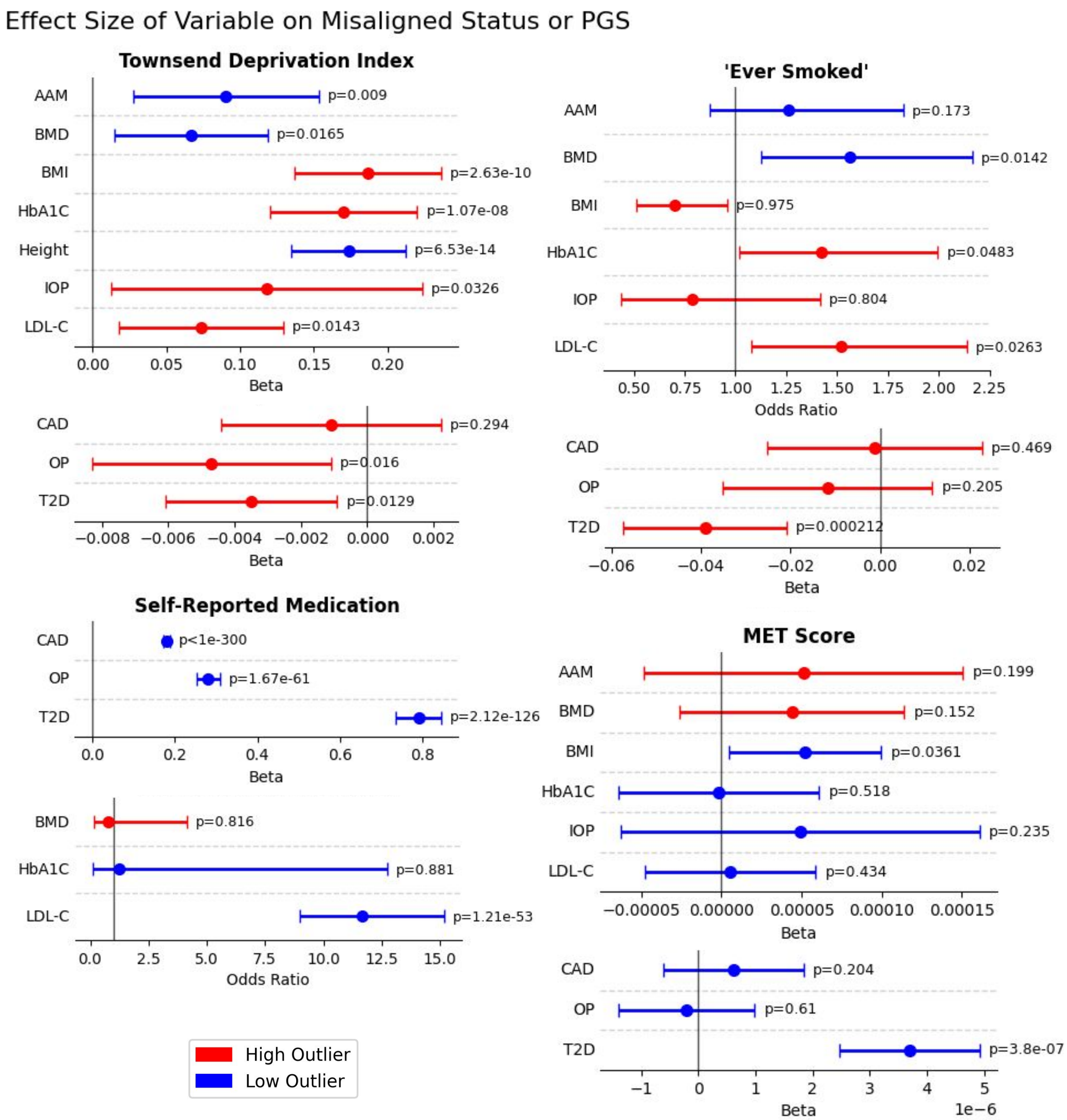

Figure S7: Effects of non-genetic variables on misaligned status. All P-values are one-sided and error bars represent 90% CIs. Fisher's exact test was used to test enrichment of overlap between binary variables (continuous trait misaligned status tested against medication status or 'Ever Smoked'), logistic regression was used to test association between binary dependent variables and continuous independent variables (continuous trait misaligned status tested against Townsend Deprivation Index and MET score), and linear regression was used to test association of continuous dependent variables and any independent variable (dichotomous trait case/control-specific PRS tested against any variable). Self-reported medication was specific to each disease or liability trait (e.g. lipid-lowering medication for CAD and LDL-C).

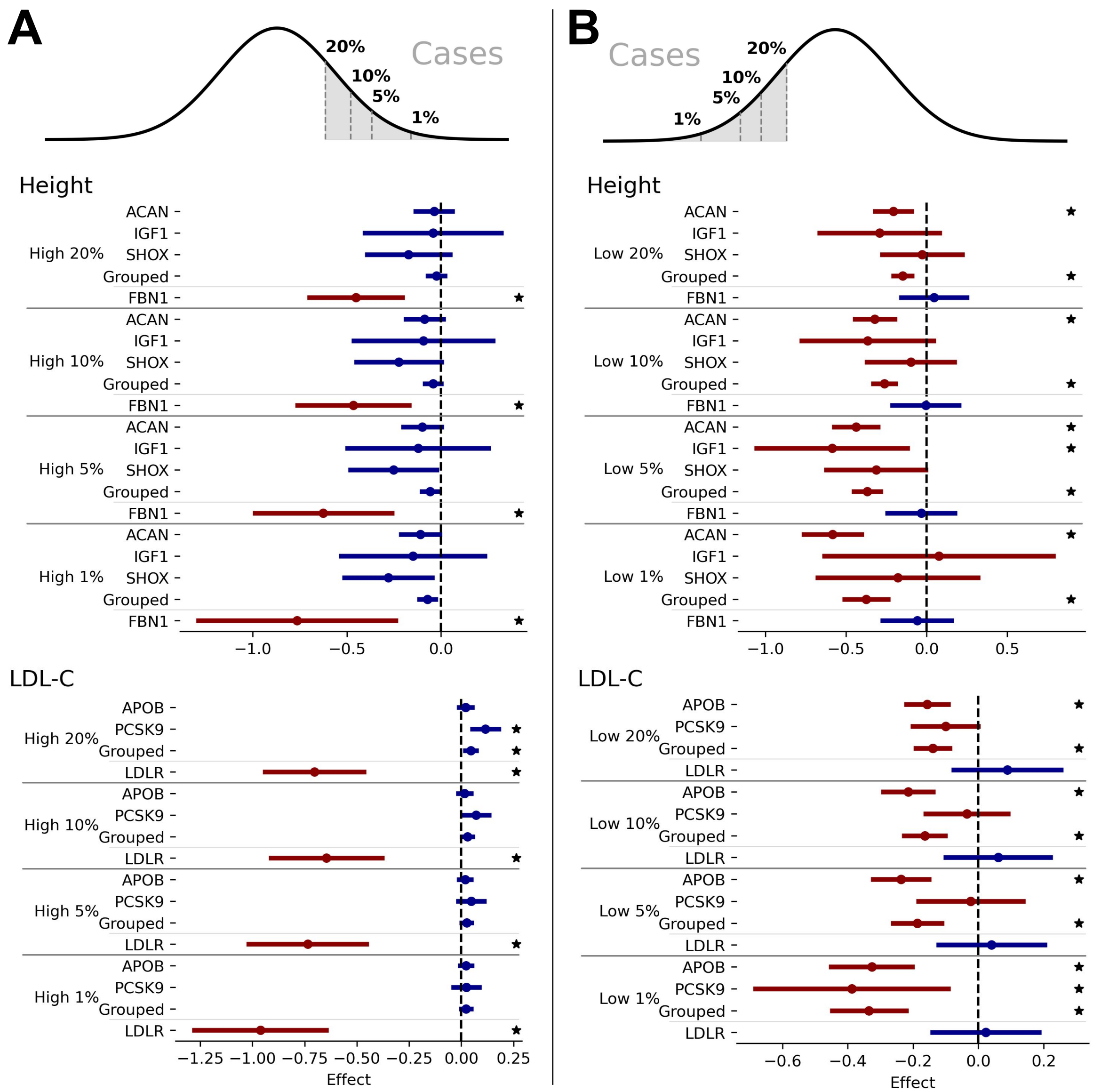

Figure S8: Enrichment testing in FDR-significant canonical genes associated with monogenic disorders for simulated dichotomous traits. (**A**) Dichotomous traits simulated with the upper tail as cases and everyone else as controls. (**B**) Dichotomous traits simulated with the lower tail as cases and everyone else as controls. Only genes and grouped genes which are significant (one-sided Fisher's exact test $P_{\text{adj}} < 0.05$) from misaligned enrichment testing of canonical genes in continuous traits are shown. Stars to the right indicate nominal significance (one-sided t-test $P < 0.05$) resulting from regression of PRS on variant presence in individuals.

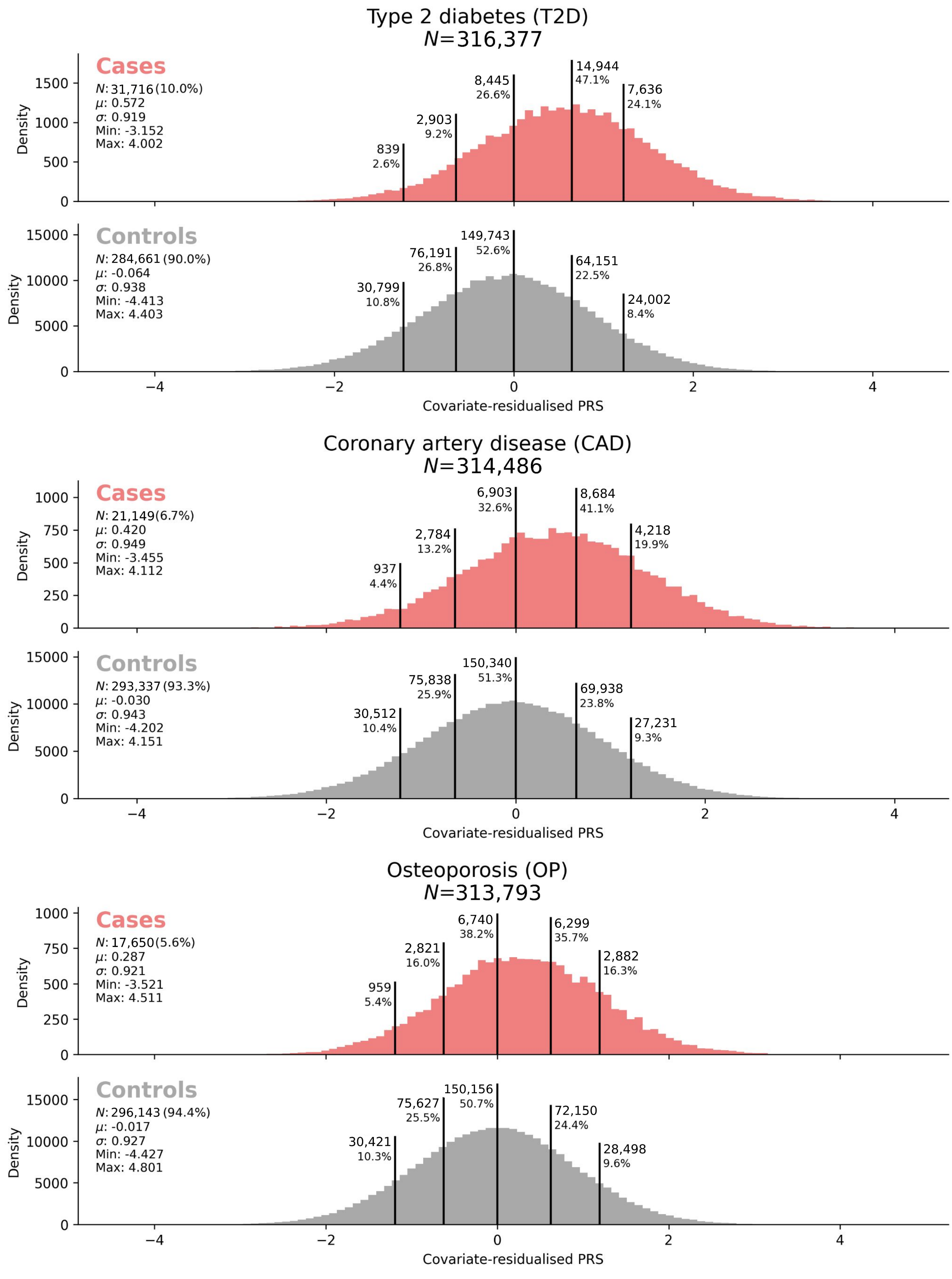

Figure S9: Distributions of covariate-residualised PRS stratified by case status. Vertical lines indicate 10th, 25th, 50th, 75th and 90th PRS percentiles, calculated on the combined distribution of PRS in both cases and controls. Numbers at the top of vertical lines, to the left or right, indicate the number and percentage of samples in the case/control group that lie to the left or right of the vertical line, respectively.

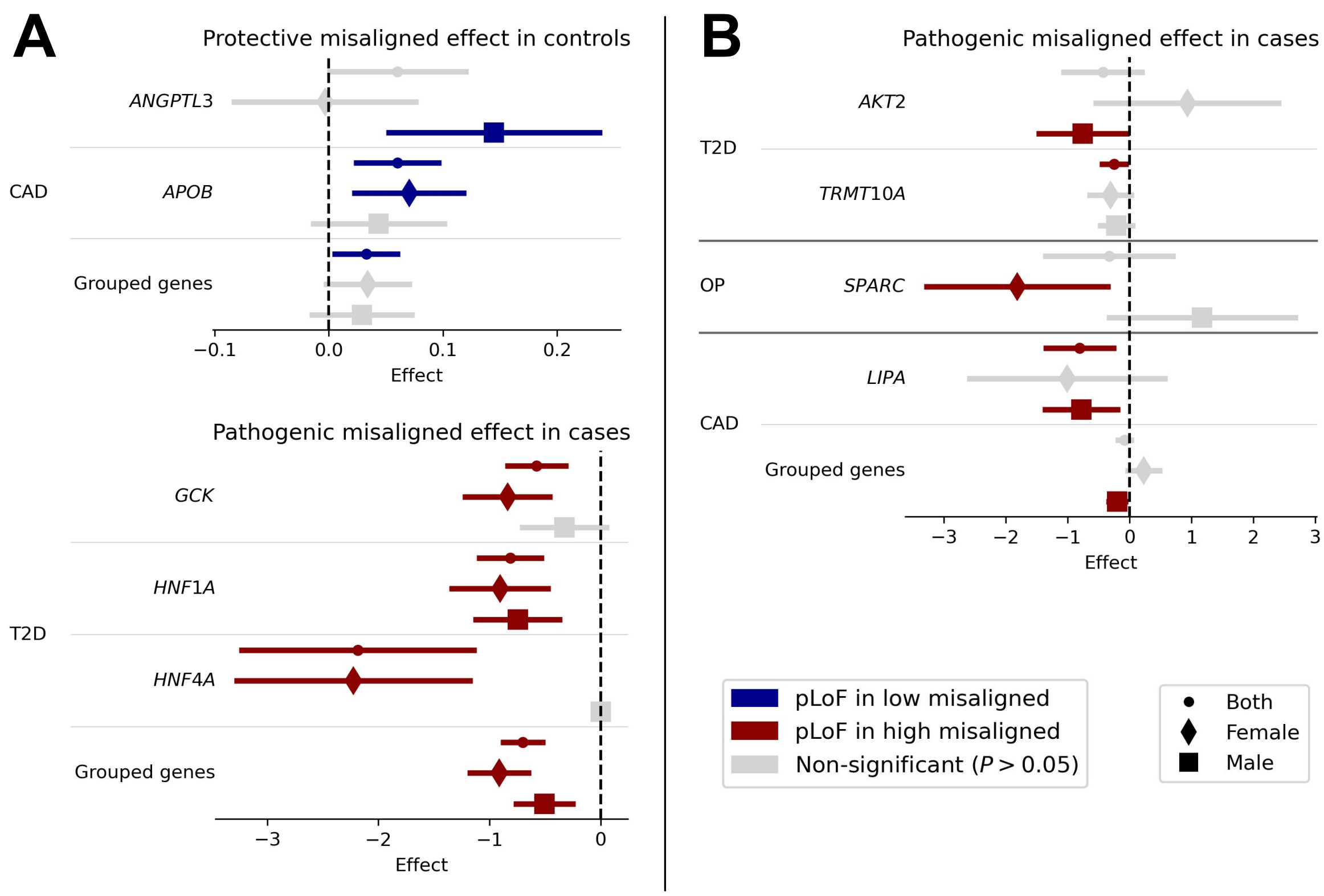

Figure S10: Sex-specific dichotomous trait results for canonical and GEL genes. (**A**) Canonical genes. (**B**) GEL genes. Only genes with at least one nominally-significant (one-sided Fisher's exact test < 0.05) result in the sex-combined cohort ("Both"), or sex-specific cohorts ("Female", "Male") are shown.

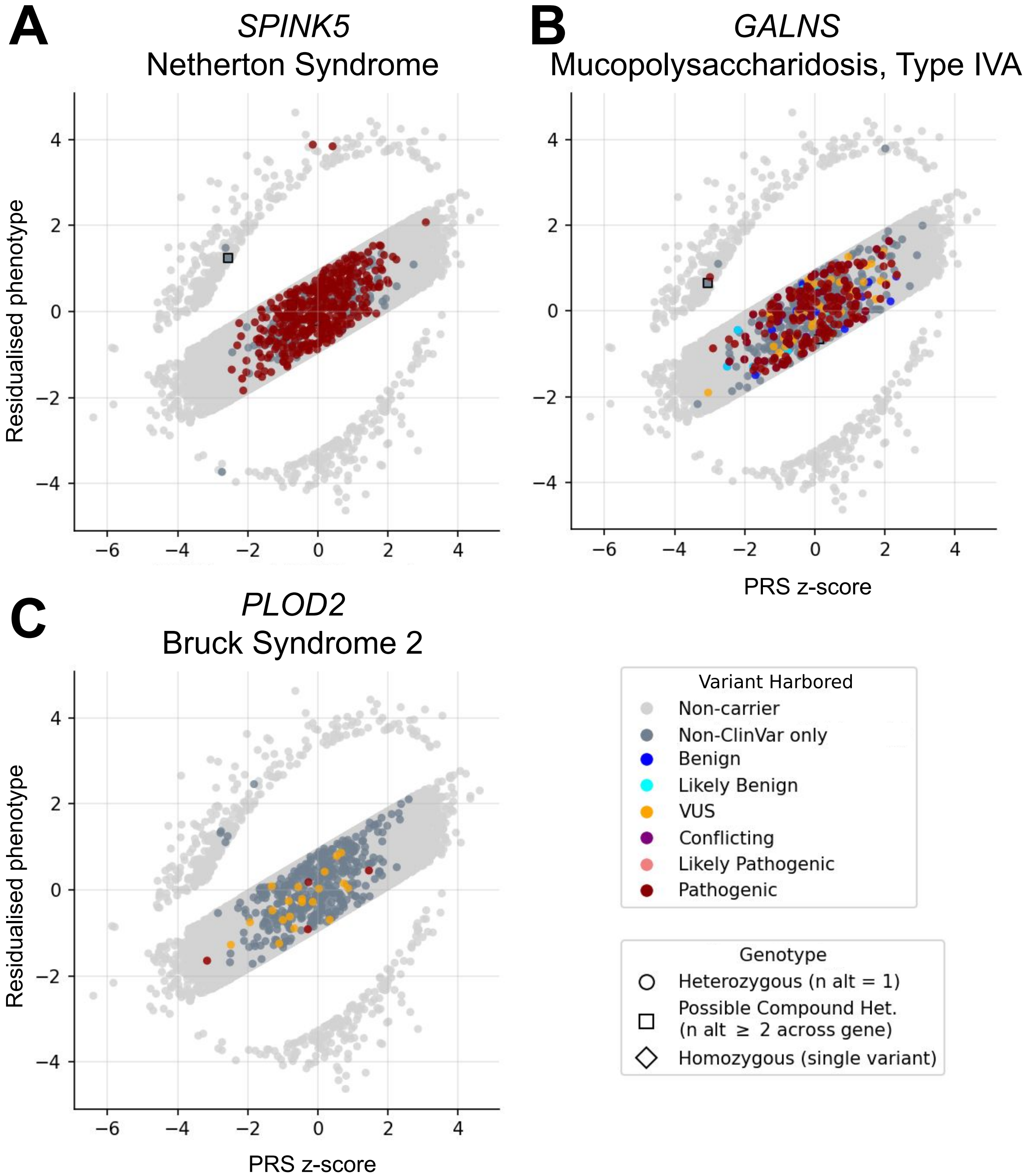

Figure S11: Enrichment of deleterious variants among high outliers for height in genes associated with short stature syndromes. (**A**) Enrichment of pLoF variants in *SPINK5*. Enrichment of damaging missense variants in (**B**) *GALNS* and (**C**) *PLOD2*. Only individuals harboring the relevant variant consequence category are highlighted (only pLoF or only damaging missense). "Possible Compound Het." individuals are defined as individuals with two or more alternate alleles of the relevant deleterious variant class (pLoF or damaging missense) across the whole gene, but not at the same variant. Homozygous individuals are those with two alternate alleles at the same variant. ClinVar[12] annotations for each gene-disorder pairing were obtained from the Open Targets platform[13].

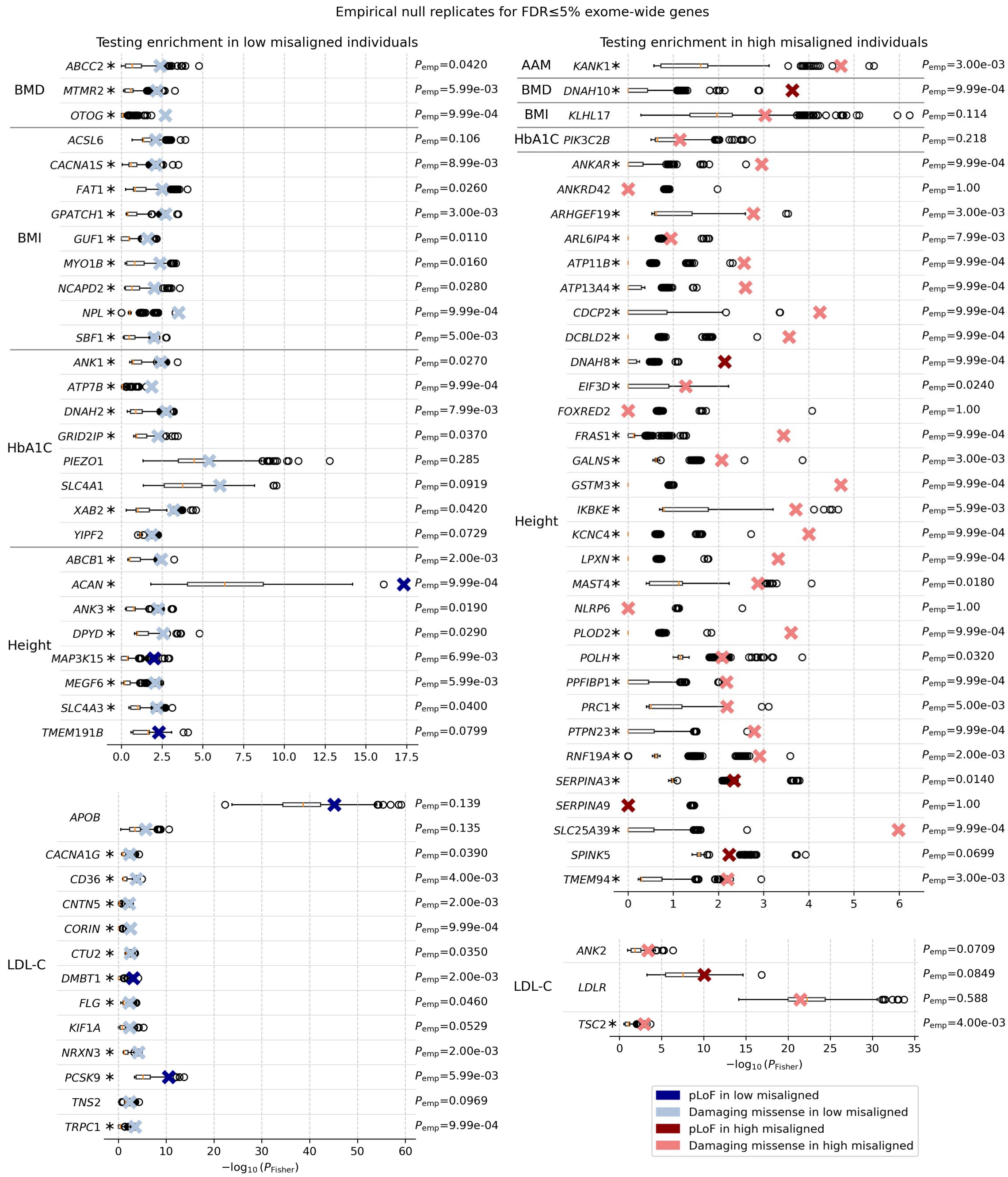

Figure S12: Empirical null replicates for FDR-significant genes nominated by the exome-wide scan. The empirical P-value (P_{emp}, labeled on the right of each plot) of a gene enrichment result indicates how the observed test statistic (one-sided Fisher's exact test P-value) compares against the empirical null distribution of test statistics (Methods). Asterisks immediately to the right of the gene symbol indicate tests where the observed enrichment outperformed the empirical null ($P_{\text{emp}} < 0.05$). LDL-C is plotted on a different x-axis scale because it tends to have stronger associations.

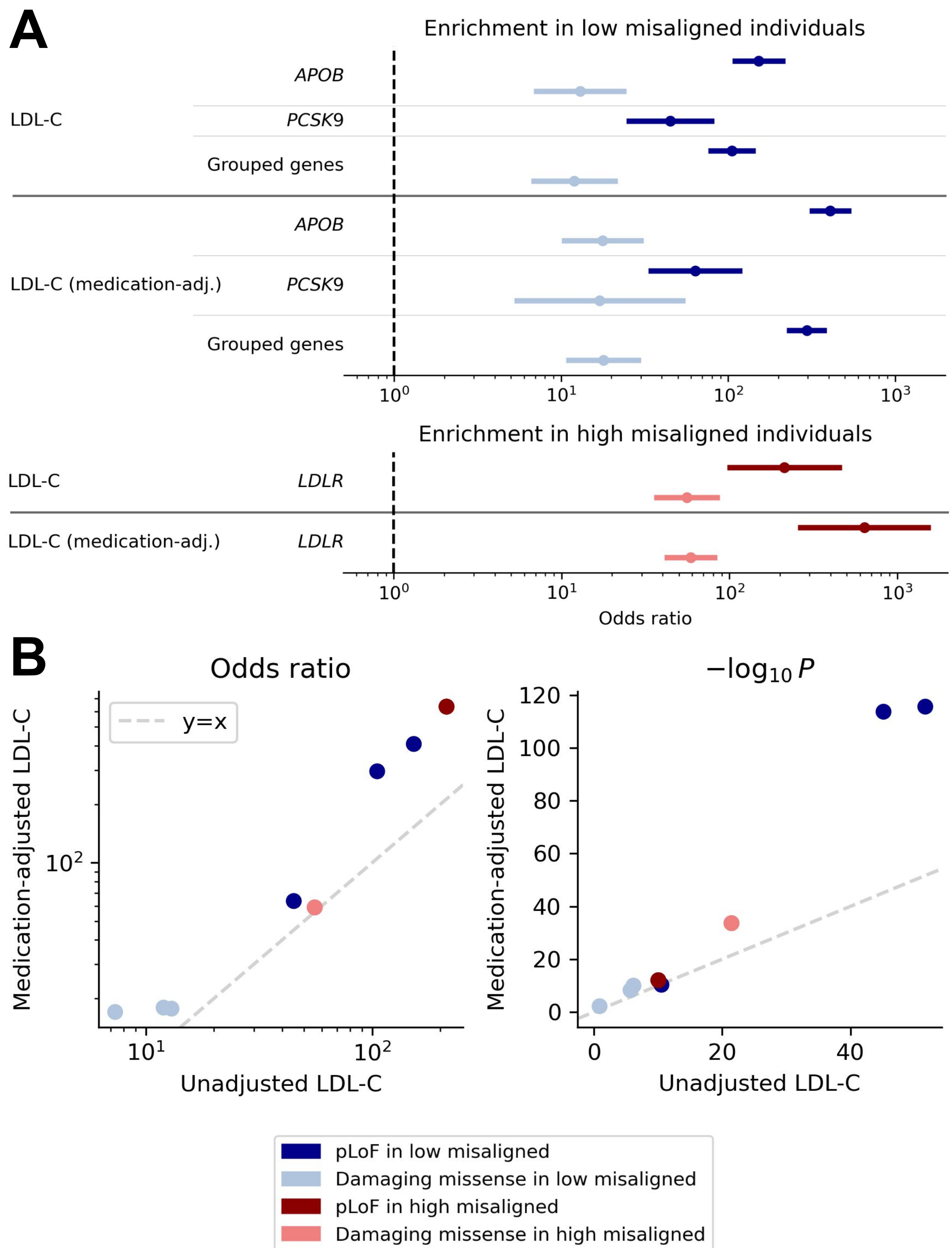

Figure S13: Comparing canonical gene enrichment results for unadjusted LDL-C to LDL-C adjusted for cholesterol-lowering medication. (**A**) Forest plot of canonical gene enrichment results. Error bars represent 90% confidence intervals. (**B**) Comparison of odds ratios and $-\log_{10} P$ for unadjusted (x-axis) and medication-adjusted (y-axis) LDL-C, where P is calculated from a one-sided Fisher's exact test. See Note S3 for description of cholesterol-lowering medication adjustment.

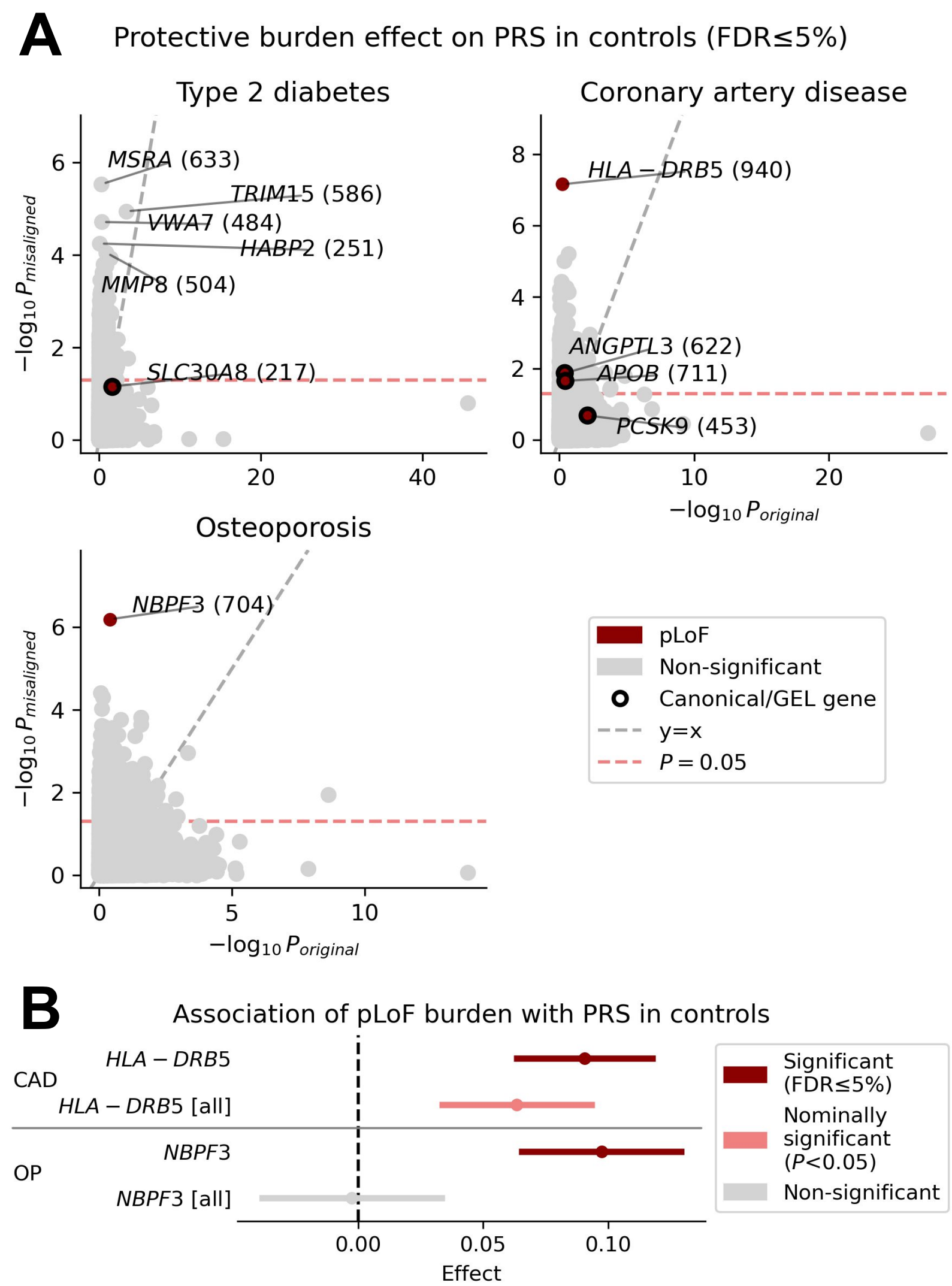

Figure S14: Strength of protective burden association with PRS in disease controls. (**A**) Comparison of strength of burden association between original trait (disease case/control status) and protective misaligned effect (effect of pLoF burden on PRS in controls). $P_{\text{misaligned}}$ is a one-sided P-value, with the alternate hypothesis that burden of pLoF variants is associated with higher PRS in disease controls. P_{original} is a two-sided P-value for the burden association with the original case-control disease trait. Significant genes are defined as having FDR-adjusted one-sided $P_{\text{misaligned}} \leq 0.05$ and are labeled with their gene symbol and, in parentheses, the total gene-level alternate allele count from REGENIE for variants included in the gene burden mask. Canonical and GEL genes are also labeled. Only genes with total alternate allele count ≥ 10 are shown. The total number of significant genes for each disease is shown in parentheses in the title of each plot. (**B**) Protective burden effects in FDR-significant genes when not conditioned or conditioned (indicated with "[all]" tag) on all common (MAF > 0.1%) QCed imputed variants in or within a 1 Mb radius of the gene tested (Methods). PRS, polygenic risk scores; pLoF, predicted loss-of-function; FDR, false discovery rate

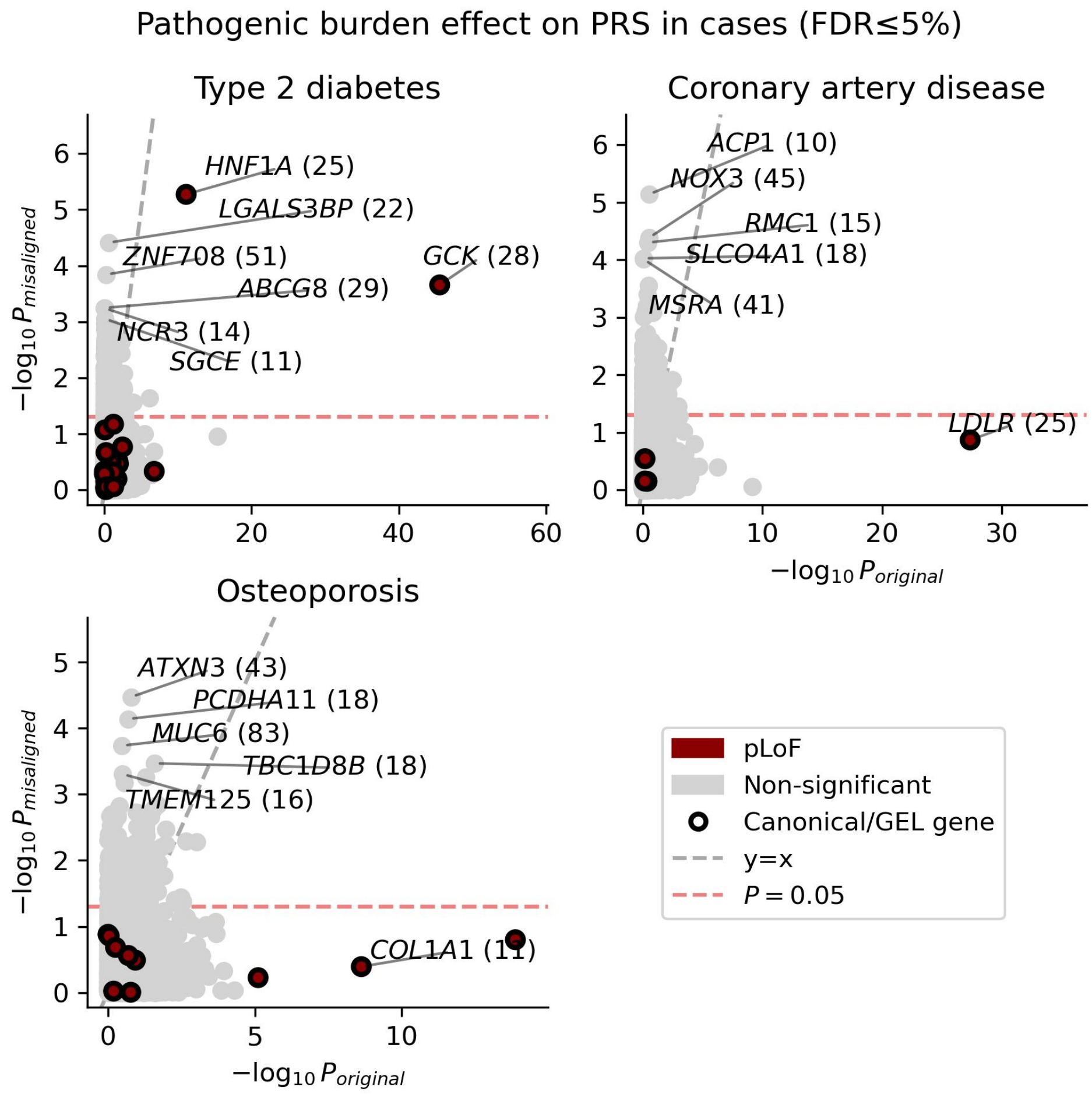

Figure S15: Strength of pathogenic burden association with PRS in disease cases. $P_{\text{misaligned}}$ is a one-sided P-value, with the alternate hypothesis that burden of pLoF variants is associated with lower PRS in disease controls. P_{original} is a two-sided P-value for the burden association with the original case-control disease trait. Significant genes, of which there were none for the pathogenic burden association testing shown here, are defined as having FDR-adjusted one-sided $P_{\text{misaligned}} \leq 0.05$. The top five genes for each trait with the strongest association strength (highest $-\log_{10} P_{\text{misaligned}}$) are labeled with their gene symbol and, in parentheses, the total gene-level alternate allele count from REGENIE for variants included in the gene burden mask. Canonical and GEL genes are also labeled. Only genes with total alternate allele count ≥ 10 are shown. PRS, polygenic risk scores; pLoF, predicted loss-of-function; FDR, false discovery rate

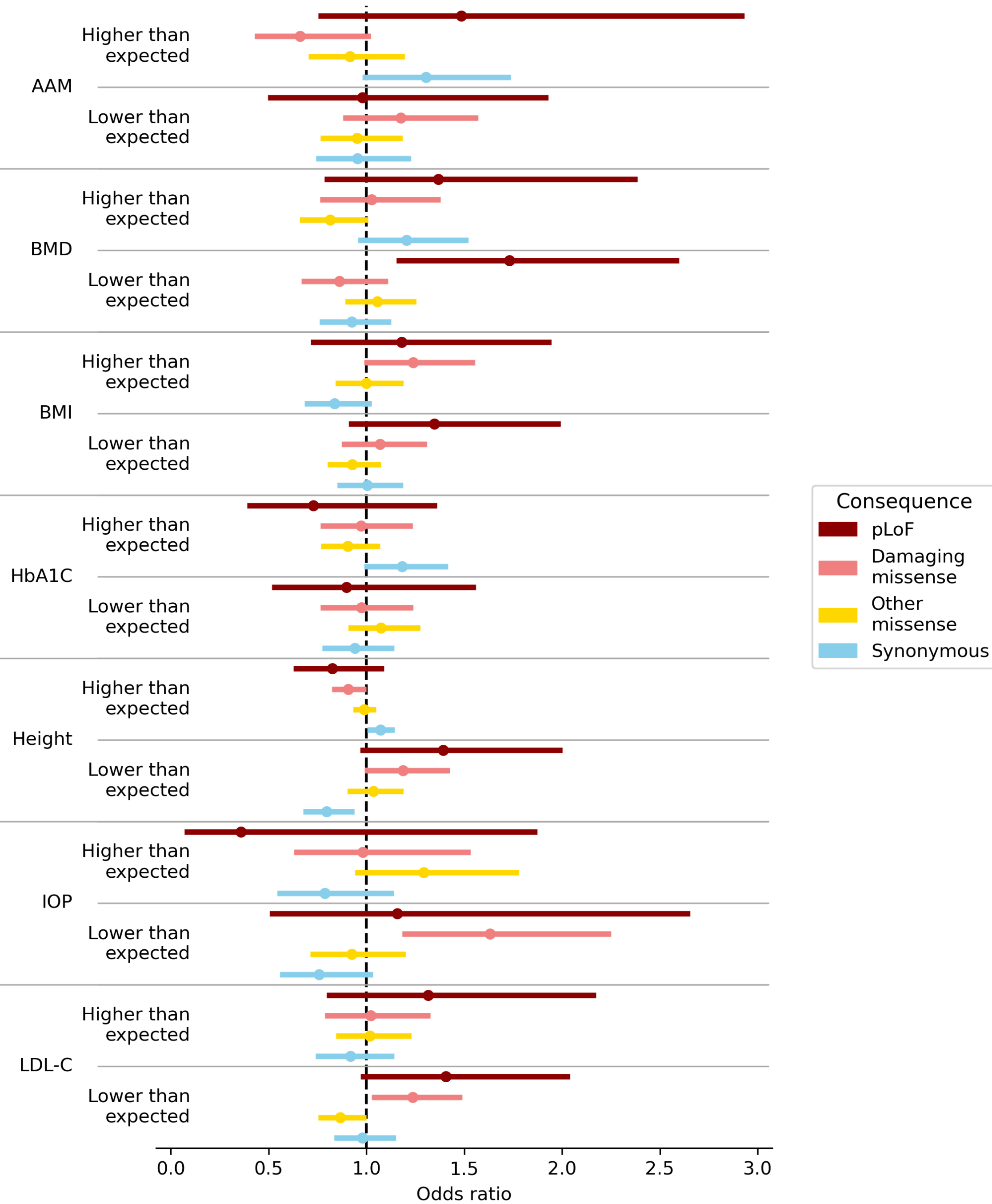

Figure S16: Enrichment of singleton burden in intolerant genes among misaligned individuals for continuous traits. Error bars are two-sided 90% CIs for OR, such that the lower error bar indicates the lower bound of the one-sided 95% CI of the OR from logistic regression of misaligned status against number of singletons, adjusted for total number of singletons (Methods).

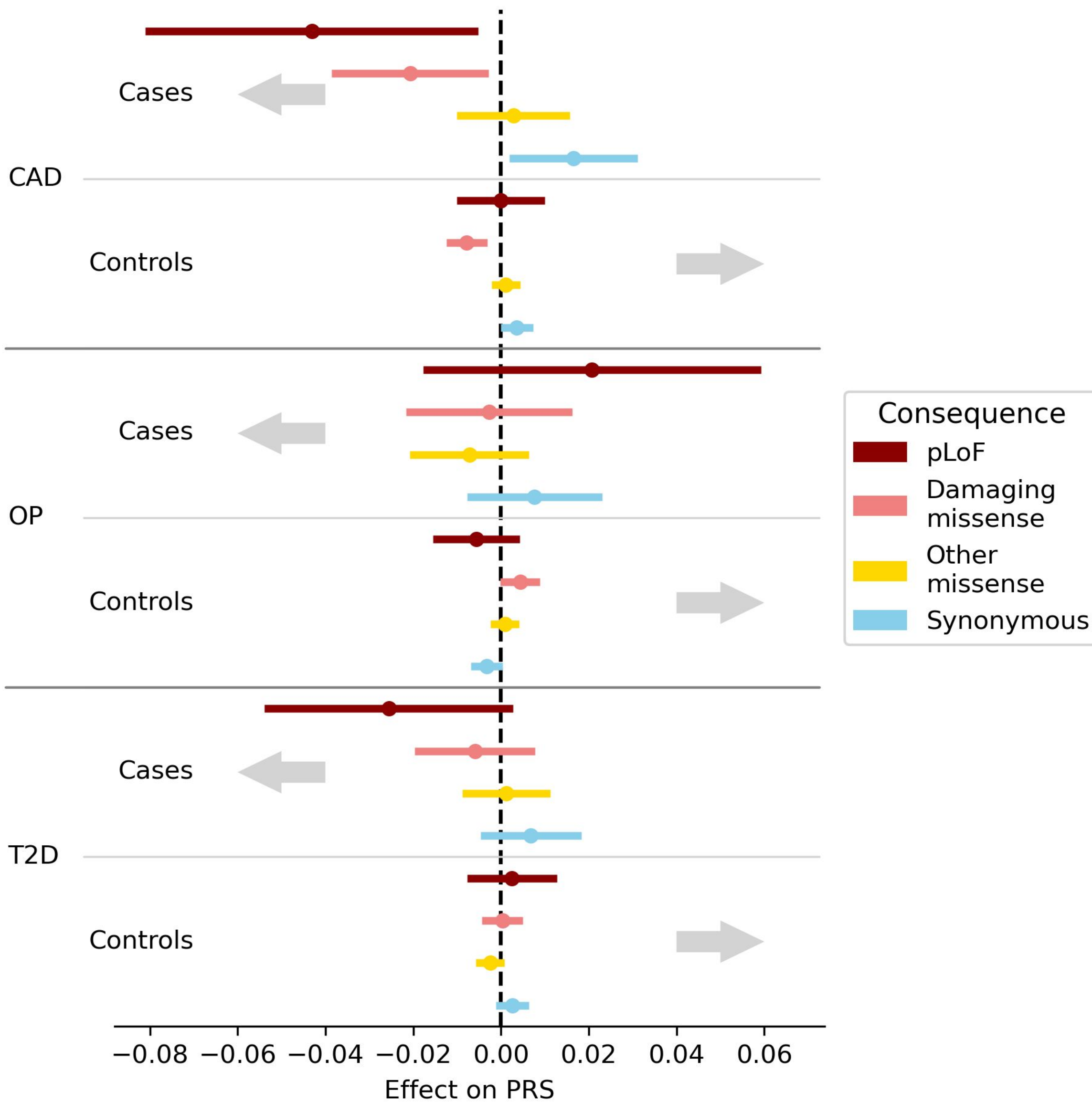

Figure S17: Enrichment of singleton burden in intolerant genes among misaligned individuals for disease traits. Error bars are two-sided 90% CIs for the effect of consequence-specific singleton burden on PRS (with total number of singletons as a covariate; see Methods), such that the upper bound (when testing cases) and lower bound (when testing controls) correspond to the upper and lower bounds, respectively, of the one-sided 95% CI. Grey arrows indicate the hypothesized direction of effect for the one-sided tests.

Supplemental tables

For supplemental tables 2-3 and 5-10, see attached supplemental material.

Phenotype (abbreviation)	pheno_id	Original phenotype	UKB data field(s) used	ICD10 code(s)	N total (subset to unrelated)	N misaligned			N aligned	N cases (subset to unrelated)	N controls (subset to unrelated)	Case prevalence	PGS	PGS UKB data field	R^2 ('PRS_only', from Thompson et al. 2024 Supp Table 4)	AUC ('PRS_only' from Thompson et al. 2024 Supp Table 3)	GEL disorder (PanelApp reviewed 'green' genes)
						Low	High	Total									
Standing height	height	Standing height	50	-	343025	185	318	503	235731	-	-	-	Standard PRS for height (HEIGHT)	26240	0.089	-	Monogenic short stature (66)<br>Beckwith-Wiedemann syndrome and other congenital overgrowth disorders (24)
LDL cholesterol (LDL-C)	ldl	LDL direct	30780	-	329391	175	109	284	225815	-	-	-	Standard PRS for low density lipoprotein cholesterol (LDL_SF)	26250	0.162	-	Familial hypercholesterolemia (9)
Body mass index (BMI)	bmi	BMI	21001	-	342697	170	111	281	234617	-	-	-	Standard PRS for body mass index (BMI)	26216	0.076	-	Severe early onset obesity (33)
Bone mineral density (BMD)	bmd	Heel BMD t-score automated	78	-	203733	122	86	208	139485	-	-	-	Standard PRS for estimated bone mineral density t-score (EBMDT)	26234	0.043	-	Osteogenesis imperfecta (34)
Intraocular pressure (IOP)	iopccmean	Intra-ocular pressure, corneal-compensated	5262, 5254	-	79598	36	32	68	54315	-	-	-	Standard PRS for intraocular pressure (IOP)	26246	0.014	-	Glaucoma, developmental (18)
Glycated haemoglobin (HbA1C)	hba1c	Glycated haemoglobin	30750	-	330025	127	111	238	225972	-	-	-	Standard PRS for glycated haemoglobin (HBA1C_DF)	26238	0.077	-	Monogenic diabetes (35)<br>Neonatal diabetes (34)
Age at menopause (AAM)	aam	Age at menopause (last menstrual period)	3581	-	117246	86	55	141	80964	-	-	-	Standard PRS for age at menopause (AAM)	26202	0.102	-	Primary ovarian insufficency (28)
Type 2 diabetes (T2D)	t2d	-	41270	E11	316377	-	-	-	-	31716	284661	0.1002	Standard PRS for type 2 diabetes (T2D)	26285	-	0.704	Monogenic diabetes (35)<br>Neonatal diabetes (34)
Coronary artery disease (CAD)	cad	-	41270	I25.1	314486	-	-	-	-	21149	293337	0.0672	Standard PRS for coronary artery disease (CAD)	26227	-	0.665	Familial hypercholesterolemia (9)<br>Extreme early-onset hypertension (15)<br>Pulmonary arterial hypertension (11)
Osteoporosis (OP)	osteoporosis	-	41270	M80+M81	313793	-	-	-	-	17650	296143	0.0562	Standard PRS for osteoporosis (OP)	26258	-	0.619	Osteogenesis imperfecta (34)

Table S1: Phenotype/PGS overview.

pheno_id	outlier_type	variable_tested	odds_ratio_90ci_fmt	pval_fisher	n_inliers	n_outliers	n_inliers_with_variable_true	n_outliers_with_variable_true	n	n_outlier	n_inlier	beta_90ci_fmt	pval_1sided_fmt
hba1c	low_outlier	atc_medication_code_prefix_A10B	1.24 [0.12, 12.73]	0.8807896528	225972	127	717	0					
bmd	high_outlier	atc_medication_code_prefix_M05B	0.79 [0.15, 4.14]	0.8158068538	139485	86	2044	1					
ldl	low_outlier	atc_medication_code_prefix_C10	11.67 [8.98, 15.17]	1.21E-53	225815	175	31844	115					
ldl	high_outlier	ever_smoked	1.52 [1.08, 2.14]	0.02625329098	224978	109	135626	76					
bmi	high_outlier	ever_smoked	0.7 [0.51, 0.96]	0.975007912	233706	110	143610	58					
bmd	low_outlier	ever_smoked	1.56 [1.13, 2.16]	0.01415429231	138977	121	83674	85					
iopccmean	high_outlier	ever_smoked	0.79 [0.44, 1.42]	0.8041299888	54065	32	33524	18					
hba1c	high_outlier	ever_smoked	1.43 [1.02, 1.99]	0.04831329822	225135	111	135911	76					
aam	low_outlier	ever_smoked	1.26 [0.88, 1.82]	0.1730638236	80713	86	46148	54					
height	low_outlier	tdi							235630	184	235446	0.174 [0.135, 0.212]	6.53E-14
ldl	high_outlier	tdi							225642	109	225533	0.0737 [0.0183, 0.129]	0.01434
bmi	high_outlier	tdi							234435	111	234324	0.187 [0.137, 0.236]	2.63E-10
bmd	low_outlier	tdi							139471	122	139349	0.0669 [0.0153, 0.118]	0.01648
iopccmean	high_outlier	tdi							54289	32	54257	0.118 [0.0127, 0.223]	0.03264
hba1c	high_outlier	tdi							225804	111	225693	0.17 [0.12, 0.22]	1.07E-08
aam	low_outlier	tdi							80952	86	80866	0.0904 [0.0275, 0.153]	9.00E-03
ldl	low_outlier	met_score							177506	132	177374	5.38e-06 [-4.78e-05, 5.86e-05]	0.4339
bmi	low_outlier	met_score							185280	127	185153	5.21e-05 [4.41e-06, 9.97e-05]	0.03615
bmd	high_outlier	met_score							108554	64	108490	4.38e-05 [-2.63e-05, 0.000114]	0.1521
iopccmean	low_outlier	met_score							43138	24	43114	4.91e-05 [-6.27e-05, 0.000161]	0.235
hba1c	low_outlier	met_score							177168	100	177068	-1.72e-06 [-6.42e-05, 6.07e-05]	0.5181
aam	high_outlier	met_score							60637	36	60601	5.11e-05 [-4.83e-05, 0.00015]	0.199
t2d	low_outlier	atc_medication_code_prefix_A10B										0.791 [0.737, 0.845]	2.12E-126
osteoporosis	low_outlier	atc_medication_code_prefix_M05B										0.281 [0.253, 0.309]	1.67E-61
cad	low_outlier	atc_medication_code_prefix_C10										0.181 [0.173, 0.188]	0.00E+00
t2d	high_outlier	tdi										-0.00349 [-0.00607, -0.000916]	0.01287
cad	high_outlier	tdi										-0.00109 [-0.0044, 0.00222]	0.2944
osteoporosis	high_outlier	tdi										-0.00469 [-0.00829, -0.00109]	0.01601
t2d	high_outlier	ever_smoked										-0.0391 [-0.0574, -0.0209]	2.12E-04
cad	high_outlier	ever_smoked										-0.00114 [-0.0251, 0.0228]	0.4687
osteoporosis	high_outlier	ever_smoked										-0.0117 [-0.0352, 0.0117]	0.2054
t2d	low_outlier	met_score										3.69e-06 [2.46e-06, 4.92e-06]	3.80E-07
cad	low_outlier	met_score										6.15e-07 [-6.08e-07, 1.84e-06]	0.204
osteoporosis	low_outlier	met_score										-2.02e-07 [-1.39e-06, 9.89e-07]	0.6099

Table S4: Association of non-genetic factors with phenotype misalignment.

pheno_id	csq_subset	max_maf	outlier_type	gene_symbol	n_total	n_inlier	n_outlier_type	n_inlier_w_variant	n_outlier_type_w_variant	n_alt	n_alt_in_subset	n_alt_in_inliers	n_alt_in_outlier_type	odds_ratio_90ci_fmt	pval_fisher_1sided_fmt
ldl_adj	pLoF	0.001	low_outlier	APOB	329391	226345	169	267	55	1107	572	516	56	409 [306-545]	2.20E-114
ldl_adj	damaging_missense	0.001	low_outlier	APOB	329391	226345	169	716	9	1143	725	716	9	17.7 [10.1-31.2]	5.31E-09
ldl_adj	pLoF	0.001	low_outlier	PCSK9	329391	226345	169	154	7	498	164	156	8	63.5 [33.2-121]	5.02E-11
ldl_adj	damaging_missense	0.001	low_outlier	PCSK9	329391	226345	169	159	2	273	161	159	2	17.0 [5.25-55.3]	6.59E-03
ldl_adj	pLoF	0.001	low_outlier	grouped:APOB-F	329391	226345	169	421	60	1605	736	672	64	295 [224-389]	2.64E-116
ldl_adj	damaging_missense	0.001	low_outlier	grouped:APOB-F	329391	226345	169	875	11	1416	886	875	11	17.9 [10.7-30.1]	1.02E-10
ldl_adj	pLoF	0.001	high_outlier	LDLR	329391	226345	183	10	5	93	15	10	5	636 [256-1580]	9.72E-13
ldl_adj	damaging_missense	0.001	high_outlier	LDLR	329391	226345	183	607	25	1248	634	609	25	58.8 [41.0-84.4]	2.39E-34
ldl	pLoF	0.001	low_outlier	APOB	329391	225815	175	258	26	1107	524	498	26	153 [106-220]	7.27E-46
ldl	damaging_missense	0.001	low_outlier	APOB	329391	225815	175	722	7	1143	729	722	7	13.0 [6.87-24.6]	1.95E-06
ldl	pLoF	0.001	low_outlier	PCSK9	329391	225815	175	240	8	498	252	244	8	45.0 [24.6-82.4]	2.97E-11
ldl	damaging_missense	0.001	low_outlier	PCSK9	329391	225815	175	177	1	273	178	177	1	7.33 [1.40-38.3]	0.129
ldl	pLoF	0.001	low_outlier	grouped:APOB-F	329391	225815	175	498	33	1605	776	742	34	105 [75.9-146]	2.25E-52
ldl	damaging_missense	0.001	low_outlier	grouped:APOB-F	329391	225815	175	899	8	1416	907	899	8	12.0 [6.59-21.8]	6.71E-07
ldl	pLoF	0.001	high_outlier	LDLR	329391	225815	109	51	5	93	57	52	5	213 [96.8-468]	8.92E-11
ldl	damaging_missense	0.001	high_outlier	LDLR	329391	225815	109	695	16	1248	713	697	16	55.7 [35.5-87.4]	3.56E-22

Table S11: LDL-C adjusted for cholesterol-lowering medication - Canonical genes associated with monogenic disorders.

pheno_id	outlier_type	csq_subset	n_inlier	n_outlier_type	n_alt	n_alt_in_subset	odds_ratio_90ci_fmt	pval_singletonburden_n_alt_1sided_fmt	qval_singletonburden_n_alt_1sided_fmt
aam	high_outlier	pLoF	80964	55	9131	6185	1.49 [0.753-2.93]	0.169	0.609
aam	high_outlier	damaging_missense	80964	55	50824	35379	0.661 [0.428-1.02]	0.941	0.969
aam	high_outlier	other_missense	80964	55	185646	129279	0.917 [0.703-1.20]	0.705	0.969
aam	high_outlier	synonymous	80964	55	92961	64948	1.31 [0.980-1.74]	0.063	0.373
aam	low_outlier	pLoF	80964	86	9131	6185	0.980 [0.497-1.93]	0.52	0.969
aam	low_outlier	damaging_missense	80964	86	50824	35402	1.18 [0.880-1.57]	0.179	0.609
aam	low_outlier	other_missense	80964	86	185646	129319	0.952 [0.765-1.18]	0.645	0.969
aam	low_outlier	synonymous	80964	86	92961	64957	0.954 [0.742-1.23]	0.62	0.969
bmd	high_outlier	pLoF	139485	86	16479	11177	1.37 [0.785-2.39]	0.177	0.609
bmd	high_outlier	damaging_missense	139485	86	90571	62018	1.03 [0.764-1.38]	0.443	0.956
bmd	high_outlier	other_missense	139485	86	325773	222913	0.815 [0.659-1.01]	0.943	0.969
bmd	high_outlier	synonymous	139485	86	162238	111165	1.21 [0.956-1.52]	0.0926	0.432
bmd	low_outlier	pLoF	139485	122	16479	11185	1.73 [1.15-2.60]	0.013	0.365
bmd	low_outlier	damaging_missense	139485	122	90571	62030	0.861 [0.669-1.11]	0.834	0.969
bmd	low_outlier	other_missense	139485	122	325773	223002	1.06 [0.890-1.26]	0.297	0.792
bmd	low_outlier	synonymous	139485	122	162238	111187	0.925 [0.760-1.13]	0.742	0.969
bmi	high_outlier	pLoF	234617	111	28165	18971	1.18 [0.716-1.95]	0.292	0.792
bmi	high_outlier	damaging_missense	234617	111	156722	106524	1.24 [0.988-1.56]	0.0598	0.373
bmi	high_outlier	other_missense	234617	111	565886	386034	1.00 [0.842-1.19]	0.498	0.969
bmi	high_outlier	synonymous	234617	111	283751	193683	0.838 [0.684-1.03]	0.925	0.969
bmi	low_outlier	pLoF	234617	170	28165	18978	1.35 [0.910-1.99]	0.106	0.455
bmi	low_outlier	damaging_missense	234617	170	156722	106539	1.07 [0.873-1.31]	0.295	0.792
bmi	low_outlier	other_missense	234617	170	565886	386098	0.928 [0.801-1.07]	0.798	0.969
bmi	low_outlier	synonymous	234617	170	283751	193731	1.00 [0.851-1.19]	0.481	0.969
hba1c	high_outlier	pLoF	225972	111	27031	18167	0.729 [0.390-1.36]	0.798	0.969
hba1c	high_outlier	damaging_missense	225972	111	150710	103003	0.973 [0.766-1.24]	0.574	0.969
hba1c	high_outlier	other_missense	225972	111	543893	374008	0.905 [0.767-1.07]	0.837	0.969
hba1c	high_outlier	synonymous	225972	111	272601	187555	1.18 [0.988-1.42]	0.062	0.373
hba1c	low_outlier	pLoF	225972	127	27031	18169	0.898 [0.517-1.56]	0.626	0.969
hba1c	low_outlier	damaging_missense	225972	127	150710	103003	0.974 [0.766-1.24]	0.571	0.969
hba1c	low_outlier	other_missense	225972	127	543893	374019	1.07 [0.906-1.27]	0.243	0.757
hba1c	low_outlier	synonymous	225972	127	272601	187532	0.940 [0.774-1.14]	0.7	0.969
height	high_outlier	pLoF	235731	318	28204	18957	0.826 [0.627-1.09]	0.872	0.969
height	high_outlier	damaging_missense	235731	318	156889	106309	0.908 [0.825-0.999]	0.951	0.969
height	high_outlier	other_missense	235731	318	566455	384251	0.989 [0.932-1.05]	0.616	0.969
height	high_outlier	synonymous	235731	318	284066	192493	1.07 [1.01-1.14]	0.0381	0.373
height	low_outlier	pLoF	235731	185	28204	18939	1.39 [0.969-2.00]	0.0667	0.373
height	low_outlier	damaging_missense	235731	185	156889	106021	1.19 [0.990-1.43]	0.0598	0.373
height	low_outlier	other_missense	235731	185	566455	382709	1.04 [0.903-1.19]	0.335	0.853
height	low_outlier	synonymous	235731	185	284066	191501	0.798 [0.677-0.939]	0.988	0.988
iopccmean	high_outlier	pLoF	54315	32	6567	4458	0.359 [0.0688-1.87]	0.846	0.969
iopccmean	high_outlier	damaging_missense	54315	32	37418	25742	0.982 [0.629-1.53]	0.527	0.969
iopccmean	high_outlier	other_missense	54315	32	137618	93565	1.29 [0.941-1.78]	0.0918	0.432
iopccmean	high_outlier	synonymous	54315	32	69953	47946	0.788 [0.545-1.14]	0.856	0.969
iopccmean	low_outlier	pLoF	54315	36	6567	4461	1.16 [0.504-2.65]	0.386	0.94
iopccmean	low_outlier	damaging_missense	54315	36	37418	25759	1.63 [1.18-2.25]	6.25E-03	0.35
iopccmean	low_outlier	other_missense	54315	36	137618	93580	0.925 [0.713-1.20]	0.688	0.969
iopccmean	low_outlier	synonymous	54315	36	69953	47957	0.759 [0.558-1.03]	0.929	0.969
ldl	high_outlier	pLoF	225815	109	27085	18450	1.32 [0.796-2.17]	0.185	0.609
ldl	high_outlier	damaging_missense	225815	109	150355	103468	1.02 [0.788-1.33]	0.444	0.956
ldl	high_outlier	other_missense	225815	109	542792	373745	1.02 [0.844-1.23]	0.435	0.956
ldl	high_outlier	synonymous	225815	109	272283	187994	0.919 [0.739-1.14]	0.737	0.969
ldl	low_outlier	pLoF	225815	175	27085	18459	1.41 [0.969-2.04]	0.0658	0.373
ldl	low_outlier	damaging_missense	225815	175	150355	103517	1.24 [1.03-1.49]	0.0294	0.373
ldl	low_outlier	other_missense	225815	175	542792	373850	0.867 [0.753-0.999]	0.951	0.969
ldl	low_outlier	synonymous	225815	175	272283	188061	0.981 [0.835-1.15]	0.58	0.969

Table S12: Singleton burden enrichment – Continuous traits.

pheno_id	outlier_type	csq_subset	n_case	n_ctrl	n_case_w_variant	n_ctrl_w_variant	beta_n_alt_90ci	pval_n_alt_1sided	qval_n_alt_1sided	q_low	q_high	odds_ratio_90ci	pval_fisher_1sided	qval_fisher_1sided
t2d	low_outlier	pLoF	31716	284661	2788	22147	2.46e-03 [-7.75e-03, 0.0127]	0.346	0.757	0.5	0.5	1.01 [0.989, 1.04]	0.190	0.880
t2d	low_outlier	pLoF	31716	284661	2788	22147	2.46e-03 [-7.75e-03, 0.0127]	0.346	0.757	0.25	0.75	1.000 [0.967, 1.03]	0.510	0.998
t2d	low_outlier	pLoF	31716	284661	2788	22147	2.46e-03 [-7.75e-03, 0.0127]	0.346	0.757	0.1	0.9	1.01 [0.955, 1.06]	0.417	0.998
t2d	high_outlier	pLoF	31716	284661	2788	22147	-0.0256 [-0.0539, 2.74e-03]	0.0687	0.757	0.5	0.5	1.06 [0.986, 1.14]	0.0959	0.780
t2d	high_outlier	pLoF	31716	284661	2788	22147	-0.0256 [-0.0539, 2.74e-03]	0.0687	0.757	0.25	0.75	1.06 [0.948, 1.20]	0.196	0.880
t2d	high_outlier	pLoF	31716	284661	2788	22147	-0.0256 [-0.0539, 2.74e-03]	0.0687	0.757	0.1	0.9	0.962 [0.769, 1.20]	0.632	0.998
t2d	low_outlier	damaging_misse	31716	284661	11081	99868	3.58e-04 [-4.31e-03, 5.03e-03]	0.450	0.757	0.5	0.5	1.00 [0.992, 1.02]	0.278	0.978
t2d	low_outlier	damaging_misse	31716	284661	11081	99868	3.58e-04 [-4.31e-03, 5.03e-03]	0.450	0.757	0.25	0.75	0.994 [0.976, 1.01]	0.715	0.998
t2d	low_outlier	damaging_misse	31716	284661	11081	99868	3.58e-04 [-4.31e-03, 5.03e-03]	0.450	0.757	0.1	0.9	0.975 [0.947, 1.00]	0.918	0.998
t2d	high_outlier	damaging_misse	31716	284661	11081	99868	-5.94e-03 [-0.0197, 7.77e-03]	0.238	0.757	0.5	0.5	1.03 [0.984, 1.07]	0.156	0.880
t2d	high_outlier	damaging_misse	31716	284661	11081	99868	-5.94e-03 [-0.0197, 7.77e-03]	0.238	0.757	0.25	0.75	1.02 [0.955, 1.10]	0.299	0.978
t2d	high_outlier	damaging_misse	31716	284661	11081	99868	-5.94e-03 [-0.0197, 7.77e-03]	0.238	0.757	0.1	0.9	1.02 [0.898, 1.15]	0.423	0.998
t2d	low_outlier	other_missense	31716	284661	23329	213522	-2.46e-03 [-5.79e-03, 8.66e-04]	0.888	0.757	0.5	0.5	0.995 [0.981, 1.01]	0.735	0.998
t2d	low_outlier	other_missense	31716	284661	23329	213522	-2.46e-03 [-5.79e-03, 8.66e-04]	0.888	0.757	0.25	0.75	0.988 [0.968, 1.01]	0.837	0.998
t2d	low_outlier	other_missense	31716	284661	23329	213522	-2.46e-03 [-5.79e-03, 8.66e-04]	0.888	0.757	0.1	0.9	0.995 [0.963, 1.03]	0.609	0.998
t2d	high_outlier	other_missense	31716	284661	23329	213522	1.19e-03 [-8.81e-03, 0.0112]	0.578	0.757	0.5	0.5	0.999 [0.953, 1.05]	0.515	0.998
t2d	high_outlier	other_missense	31716	284661	23329	213522	1.19e-03 [-8.81e-03, 0.0112]	0.578	0.757	0.25	0.75	1.05 [0.970, 1.13]	0.169	0.880
t2d	high_outlier	other_missense	31716	284661	23329	213522	1.19e-03 [-8.81e-03, 0.0112]	0.578	0.757	0.1	0.9	0.880 [0.770, 1.01]	0.947	0.998
t2d	low_outlier	synonymous	31716	284661	15303	145110	2.56e-03 [-1.19e-03, 6.31e-03]	0.131	0.757	0.5	0.5	0.993 [0.981, 1.01]	0.822	0.998
t2d	low_outlier	synonymous	31716	284661	15303	145110	2.56e-03 [-1.19e-03, 6.31e-03]	0.131	0.757	0.25	0.75	1.00 [0.984, 1.02]	0.431	0.998
t2d	low_outlier	synonymous	31716	284661	15303	145110	2.56e-03 [-1.19e-03, 6.31e-03]	0.131	0.757	0.1	0.9	0.988 [0.960, 1.02]	0.760	0.998
t2d	high_outlier	synonymous	31716	284661	15303	145110	6.81e-03 [-4.68e-03, 0.0183]	0.835	0.757	0.5	0.5	1.01 [0.964, 1.05]	0.422	0.998
t2d	high_outlier	synonymous	31716	284661	15303	145110	6.81e-03 [-4.68e-03, 0.0183]	0.835	0.757	0.25	0.75	0.971 [0.908, 1.04]	0.775	0.998
t2d	high_outlier	synonymous	31716	284661	15303	145110	6.81e-03 [-4.68e-03, 0.0183]	0.835	0.757	0.1	0.9	1.00 [0.888, 1.13]	0.510	0.998
cad	low_outlier	pLoF	21149	293337	1672	23070	-7.65e-05 [-0.0101, 9.98e-03]	0.505	0.757	0.5	0.5	0.993 [0.971, 1.02]	0.703	0.998
cad	low_outlier	pLoF	21149	293337	1672	23070	-7.65e-05 [-0.0101, 9.98e-03]	0.505	0.757	0.25	0.75	0.998 [0.966, 1.03]	0.544	0.998
cad	low_outlier	pLoF	21149	293337	1672	23070	-7.65e-05 [-0.0101, 9.98e-03]	0.505	0.757	0.1	0.9	0.960 [0.912, 1.01]	0.909	0.998
cad	high_outlier	pLoF	21149	293337	1672	23070	-0.0431 [-0.0810, -5.20e-03]	0.0307	0.757	0.5	0.5	1.07 [0.980, 1.17]	0.108	0.780
cad	high_outlier	pLoF	21149	293337	1672	23070	-0.0431 [-0.0810, -5.20e-03]	0.0307	0.757	0.25	0.75	1.20 [1.06, 1.36]	0.0116	0.279
cad	high_outlier	pLoF	21149	293337	1672	23070	-0.0431 [-0.0810, -5.20e-03]	0.0307	0.757	0.1	0.9	1.18 [0.959, 1.46]	0.106	0.780
cad	low_outlier	damaging_misse	21149	293337	7156	103237	-7.77e-03 [-0.0124, -3.16e-03]	0.997	0.757	0.5	0.5	0.978 [0.965, 0.990]	0.998	0.998
cad	low_outlier	damaging_misse	21149	293337	7156	103237	-7.77e-03 [-0.0124, -3.16e-03]	0.997	0.757	0.25	0.75	0.972 [0.955, 0.990]	0.995	0.998
cad	low_outlier	damaging_misse	21149	293337	7156	103237	-7.77e-03 [-0.0124, -3.16e-03]	0.997	0.757	0.1	0.9	0.955 [0.928, 0.983]	0.996	0.998
cad	high_outlier	damaging_misse	21149	293337	7156	103237	-0.0207 [-0.0386, -2.80e-03]	0.0285	0.757	0.5	0.5	1.03 [0.977, 1.08]	0.194	0.880
cad	high_outlier	damaging_misse	21149	293337	7156	103237	-0.0207 [-0.0386, -2.80e-03]	0.0285	0.757	0.25	0.75	1.06 [0.987, 1.15]	0.0901	0.780
cad	high_outlier	damaging_misse	21149	293337	7156	103237	-0.0207 [-0.0386, -2.80e-03]	0.0285	0.757	0.1	0.9	1.12 [0.986, 1.26]	0.0773	0.780
cad	low_outlier	other_missense	21149	293337	15328	220347	1.11e-03 [-2.19e-03, 4.40e-03]	0.290	0.757	0.5	0.5	0.987 [0.974, 1.00]	0.930	0.998
cad	low_outlier	other_missense	21149	293337	15328	220347	1.11e-03 [-2.19e-03, 4.40e-03]	0.290	0.757	0.25	0.75	0.989 [0.969, 1.01]	0.826	0.998
cad	low_outlier	other_missense	21149	293337	15328	220347	1.11e-03 [-2.19e-03, 4.40e-03]	0.290	0.757	0.1	0.9	0.970 [0.940, 1.00]	0.943	0.998
cad	high_outlier	other_missense	21149	293337	15328	220347	2.86e-03 [-0.0100, 0.0157]	0.642	0.757	0.5	0.5	0.976 [0.924, 1.03]	0.780	0.998
cad	high_outlier	other_missense	21149	293337	15328	220347	2.86e-03 [-0.0100, 0.0157]	0.642	0.757	0.25	0.75	0.974 [0.899, 1.06]	0.713	0.998
cad	high_outlier	other_missense	21149	293337	15328	220347	2.86e-03 [-0.0100, 0.0157]	0.642	0.757	0.1	0.9	0.959 [0.839, 1.09]	0.715	0.998
cad	low_outlier	synonymous	21149	293337	10185	149546	3.64e-03 [-7.58e-05, 7.35e-03]	0.0536	0.757	0.5	0.5	0.997 [0.985, 1.01]	0.657	0.998
cad	low_outlier	synonymous	21149	293337	10185	149546	3.64e-03 [-7.58e-05, 7.35e-03]	0.0536	0.757	0.25	0.75	0.993 [0.976, 1.01]	0.752	0.998
cad	low_outlier	synonymous	21149	293337	10185	149546	3.64e-03 [-7.58e-05, 7.35e-03]	0.0536	0.757	0.1	0.9	0.990 [0.963, 1.02]	0.734	0.998
cad	high_outlier	synonymous	21149	293337	10185	149546	0.0166 [1.95e-03, 0.0312]	0.969	0.757	0.5	0.5	0.954 [0.909, 1.00]	0.946	0.998
cad	high_outlier	synonymous	21149	293337	10185	149546	0.0166 [1.95e-03, 0.0312]	0.969	0.757	0.25	0.75	0.980 [0.912, 1.05]	0.690	0.998
cad	high_outlier	synonymous	21149	293337	10185	149546	0.0166 [1.95e-03, 0.0312]	0.969	0.757	0.1	0.9	1.01 [0.899, 1.14]	0.448	0.998
osteoporosis	low_outlier	pLoF	17650	296143	1478	23268	-5.61e-03 [-0.0155, 4.26e-03]	0.825	0.825	0.5	0.5	0.989 [0.967, 1.01]	0.793	0.998
osteoporosis	low_outlier	pLoF	17650	296143	1478	23268	-5.61e-03 [-0.0155, 4.26e-03]	0.825	0.825	0.25	0.75	0.992 [0.960, 1.02]	0.673	0.998
osteoporosis	low_outlier	pLoF	17650	296143	1478	23268	-5.61e-03 [-0.0155, 4.26e-03]	0.825	0.825	0.1	0.9	0.963 [0.916, 1.01]	0.893	0.998
osteoporosis	high_outlier	pLoF	17650	296143	1478	23268	0.0207 [-0.0177, 0.0592]	0.812	0.825	0.5	0.5	0.932 [0.849, 1.02]	0.900	0.998
osteoporosis	high_outlier	pLoF	17650	296143	1478	23268	0.0207 [-0.0177, 0.0592]	0.812	0.825	0.25	0.75	0.953 [0.831, 1.09]	0.731	0.998
osteoporosis	high_outlier	pLoF	17650	296143	1478	23268	0.0207 [-0.0177, 0.0592]	0.812	0.825	0.1	0.9	0.782 [0.620, 0.986]	0.967	0.998
osteoporosis	low_outlier	damaging_misse	17650	296143	6086	104160	4.41e-03 [-1.10e-04, 8.92e-03]	0.0543	0.825	0.5	0.5	1.02 [1.01, 1.03]	6.09e-03	0.279
osteoporosis	low_outlier	damaging_misse	17650	296143	6086	104160	4.41e-03 [-1.10e-04, 8.92e-03]	0.0543	0.825	0.25	0.75	1.03 [1.01, 1.05]	8.44e-03	0.279
osteoporosis	low_outlier	damaging_misse	17650	296143	6086	104160	4.41e-03 [-1.10e-04, 8.92e-03]	0.0543	0.825	0.1	0.9	1.02 [0.991, 1.05]	0.132	0.865
osteoporosis	high_outlier	damaging_misse	17650	296143	6086	104160	-2.68e-03 [-0.0216, 0.0163]	0.408	0.825	0.5	0.5	1.03 [0.973, 1.08]	0.213	0.895
osteoporosis	high_outlier	damaging_misse	17650	296143	6086	104160	-2.68e-03 [-0.0216, 0.0163]	0.408	0.825	0.25	0.75	0.973 [0.899, 1.05]	0.727	0.998
osteoporosis	high_outlier	damaging_misse	17650	296143	6086	104160	-2.68e-03 [-0.0216, 0.0163]	0.408	0.825	0.1	0.9	0.935 [0.820, 1.07]	0.810	0.998
osteoporosis	low_outlier	other_missense	17650	296143	12846	222313	8.70e-04 [-2.36e-03, 4.10e-03]	0.329	0.825	0.5	0.5	1.02 [1.00, 1.03]	0.0360	0.518
osteoporosis	low_outlier	other_missense	17650	296143	12846	222313	8.70e-04 [-2.36e-03, 4.10e-03]	0.329	0.825	0.25	0.75	1.01 [0.988, 1.03]	0.264	0.978
osteoporosis	low_outlier	other_missense	17650	296143	12846	222313	8.70e-04 [-2.36e-03, 4.10e-03]	0.329	0.825	0.1	0.9	1.04 [1.01, 1.07]	0.0222	0.400
osteoporosis	high_outlier	other_missense	17650	296143	12846	222313	-7.20e-03 [-0.0208, 6.40e-03]	0.192	0.825	0.5	0.5	1.01 [0.952, 1.07]	0.417	0.998
osteoporosis	high_outlier	other_missense	17650	296143	12846	222313	-7.20e-03 [-0.0208, 6.40e-03]	0.192	0.825	0.25	0.75	0.931 [0.856, 1.01]	0.925	0.998
osteoporosis	high_outlier	other_missense	17650	296143	12846	222313	-7.20e-03 [-0.0208, 6.40e-03]	0.192	0.825	0.1	0.9	0.922 [0.804, 1.06]	0.845	0.998
osteoporosis	low_outlier	synonymous	17650	296143	8609	150840	-3.21e-03 [-6.85e-03, 4.34e-04]	0.926	0.825	0.5	0.5	0.998 [0.986, 1.01]	0.589	0.998
osteoporosis	low_outlier	synonymous	17650	296143	8609	150840	-3.21e-03 [-6.85e-03, 4.34e-04]	0.926	0.825	0.25	0.75	1.01 [0.989, 1.02]	0.288	0.978
osteoporosis	low_outlier	synonymous	17650	296143	8609	150840	-3.21e-03 [-6.85e-03, 4.34e-04]	0.926	0.825	0.1	0.9	1.01 [0.986, 1.04]	0.224	0.895
osteoporosis	high_outlier	synonymous	17650	296143	8609	150840	7.61e-03 [-7.73e-03, 0.0230]	0.793	0.825	0.5	0.5	0.971 [0.923, 1.02]	0.832	0.998
osteoporosis	high_outlier	synonymous	17650	296143	8609	150840	7.61e-03 [-7.73e-03, 0.0230]	0.793	0.825	0.25	0.75	0.910 [0.844, 0.980]	0.982	0.998
osteoporosis	high_outlier	synonymous	17650	296143	8609	150840	7.61e-03 [-7.73e-03, 0.0230]	0.793	0.825	0.1	0.9	0.885 [0.783, 1.00]	0.953	0.998

Table S13: Singleton burden enrichment – Dichotomous traits.

Supplemental references

1. Hawkes, G., Yengo, L., Vedantam, S., Marouli, E., Beaumont, R.N., GIANT Consortium, Tyrrell, J., Weedon, M.N., Hirschhorn, J., Frayling, T.M., et al. (2023). Identification and analysis of individuals who deviate from their genetically-predicted phenotype. PLoS Genet. *19*, e1010934. 10.1371/journal.pgen.1010934.

2. Wu, Y., Byrne, E.M., Zheng, Z., Kemper, K.E., Yengo, L., Mallett, A.J., Yang, J., Visscher, P.M., Wray, N.R. (2019). Genome-wide association study of medication-use and associated disease in the UK Biobank. Nat. Commun. *10*, 1891. 10.1038/s41467-019-09572-5.

3. Benjamini, Y., Hochberg, Y. (1995). Controlling the False Discovery Rate: A Practical and Powerful Approach to Multiple Testing. J. R. Stat. Soc. Ser. B Methodol. *57*, 289–300.

4. Tieber, V.L., Abruzzini, L.F., Didier, D.K., Schwartz, B.D., Rotwein, P. (1986). Complete characterization and sequence of an HLA class II DR beta chain cDNA from the DR5 haplotype. J. Biol. Chem. *261*, 2738–2742. 10.1016/S0021-9258(17)35847-7.

5. Ajoolabady, A., Pratico, D., Lin, L., Mantzoros, C.S., Bahijri, S., Tuomilehto, J., Ren, J. (2024). Inflammation in atherosclerosis: pathophysiology and mechanisms. Cell Death Dis. *15*, 817. 10.1038/s41419-024-07228-3.

6. Patel, A.P., Wang, M., Pirruccello, J.P., Ellinor, P.T., Ng, K., Kathiresan, S., Khera, A.V. (2021). Lipoprotein(a) concentrations and incident atherosclerotic cardiovascular disease: new insights from a large national biobank. Arterioscler. Thromb. Vasc. Biol. *41*, 465–474. 10.1161/ATVBAHA.120.315291.

7. Purcell, S., Neale, B., Todd-Brown, K., Thomas, L., Ferreira, M.A.R., Bender, D., Maller, J., Sklar, P., Bakker, P.I.W. de, Daly, M.J., et al. (2007). PLINK: a tool set for whole-genome association and population-based linkage analyses. Am. J. Hum. Genet. *81*, 559–575. 10.1086/519795.

8. Karczewski, K.J., Francioli, L.C., Tiao, G., Cummings, B.B., Alföldi, J., Wang, Q., Collins, R.L., Laricchia, K.M., Ganna, A., Birnbaum, D.P., et al. (2020). The mutational constraint spectrum quantified from variation in 141,456 humans. Nature *581*, 434–443. 10.1038/s41586-020-2308-7.

9. Genovese, G., Fromer, M., Stahl, E.A., Ruderfer, D.M., Chambert, K., Landén, M., Moran, J.L., Purcell, S.M., Sklar, P., Sullivan, P.F., et al. (2016). Increased burden of ultra-rare protein-altering variants among 4,877 individuals with schizophrenia. Nat. Neurosci. *19*, 1433–1441. 10.1038/nn.4402.

10. Backman, J.D., Li, A.H., Marcketta, A., Sun, D., Mbatchou, J., Kessler, M.D., Benner, C., Liu, D., Locke, A.E., Balasubramanian, S., et al. (2021). Exome sequencing and analysis of 454,787 UK Biobank participants. Nature *599*, 628–634. 10.1038/s41586-021-04103-z.

11. Hawkes, G., Beaumont, R.N., Li, Z., Mandla, R., Li, X., Albert, C.M., Arnett, D.K., Ashley-Koch, A.E., Ashrani, A.A., Barnes, K.C., et al. (2024). Whole-genome sequencing in 333,100 individuals reveals rare non-coding single variant and aggregate associations with height. Nat. Commun. *15*, 8549. 10.1038/s41467-024-52579-w.

12. Landrum, M.J., Lee, J.M., Riley, G.R., Jang, W., Rubinstein, W.S., Church, D.M., Maglott, D.R. (2014). ClinVar: public archive of relationships among sequence variation and human phenotype. Nucleic Acids Res. *42*, D980–5. 10.1093/nar/gkt1113.

13. Buniello, A., Suveges, D., Cruz-Castillo, C., Llinares, M.B., Cornu, H., Lopez, I., Tsukanov, K., Roldán-Romero, J.M., Mehta, C., Fumis, L., et al. (2025). Open Targets Platform: facilitating therapeutic hypotheses building in drug discovery. Nucleic Acids Res. *53*, D1467–D1475. 10.1093/nar/gkae1128.
